# Exploring the anticancer potential of Actinidia chinensis Planch root extracts (*acRoots*) on hepatocellular carcinoma: A molecular mechanism study

**DOI:** 10.1016/j.heliyon.2023.e21851

**Published:** 2023-11-02

**Authors:** Kaijie Qiu, Qing Xia, Hao Chen, Qiong Ye, Haixiang Mao, Mei Tian, Yichao Gan, Qinyuan Huang, Haibiao Wang, Shiwei Duan

**Affiliations:** a Department of Hepatobiliary and Pancreatic Surgery, Ningbo Medical Center Lihuili Hospital, Ningbo, Zhejiang, 315048, China; b College of Pharmacy, Zhejiang University of Technology, Hangzhou, Zhejiang, 310014, China; c Department of Orthopaedic Surgery, Ningbo Medical Center Lihuili Hospital, Ningbo, Zhejiang, 315048, China; d Key Laboratory of Novel Targets and Drug Study for Neural Repair of Zhejiang Province, School of Medicine, Hangzhou City University, Hangzhou, Zhejiang, 310014, China

**Keywords:** *Actinidia chinensis planch Root extracts* (*acRoots*), Hepatocellular carcinoma, Anticancer efficacy, AKT/mTOR signaling, Apoptosis, Autophagy

## Abstract

Hepatocellular carcinoma (HCC), ranking as the seventh most prevalent cancer worldwide, poses a significant health challenge. *Actinidia chinensis Planch Root extracts* (*acRoots*), a traditional Chinese medicine, has exhibited promising inhibitory effects on the proliferation, invasion, and migration of various cancer cell types. Nevertheless, its specific impact and underlying mechanisms concerning HCC remain unclear. This research aimed to elucidate the anticancer properties and potential molecular mechanisms of *acRoots* in the HepG2 and LM3 cell lines. Our findings demonstrate that *acRoots* effectively hampers the in vitro proliferation, migration, and invasion of HCC cells. Furthermore, *acRoots* induces apoptosis and autophagy by impeding the AKT/mTOR signaling pathway, with its inhibitory effects on cells being restored under AKT activator induction. This study, for the first time, elucidates that *acRoots* can suppress HepG2 and LM3 cell proliferation by blocking the Akt/mTOR pathway, thereby activating apoptosis and autophagy. These results underscore the potential of *acRoots* as a promising antitumor agent for HCC.

## Abbreviations

**HCC**: hepatocellular carcinoma
**acRoots**: Actinidia chinensis Planch Root extracts
**DLX2**: distal-less homeobox 2
**TARBP2**: RISC loading complex RNA binding subunit
**AKT**: RAC-alpha serine RAC-alpha serine
**P-AKT**: Phosphorylation
**mTOR**: mammalian target of Rapamycin
**P-mTOR**: Phosphorylation mammalian target of Rapamycin
**DMEM**: Dulbecco's modified eagle medium
**CCK-8**: Cell Counting Kit-8
**SDS-PAGE**: sodium dodecyl sulfate-polyacrylamide gel electrophoresis
**EMT**: Epithelial-Mesenchymal Transition
**FBS**: Fetal bovine serum
**PBS**: phosphate-buffered saline
**NS**: No significant difference

## 1 Introduction

Hepatocellular carcinoma (HCC) stands as a prevalent primary liver cancer, accounting for approximately 75 % of all liver cancer cases worldwide [1]. It's a sobering reality that HCC ranks as the second leading cause of cancer-related deaths globally, responsible for about 9.1 % of all cancer-related fatalities [2]. Conventional treatment options for HCC predominantly revolve around surgical interventions and chemotherapy [3]. Sorafenib, Lenvatinib, and Nivolumab take the frontline in HCC treatment [[4], [5], [6]]. However, these medications often come with a host of dose-limiting toxicities, encompassing discomfort in the gastrointestinal tract, loss of appetite, weight decline, and elevated blood pressure [[7], [8], [9], [10]]. Consequently, there exists a compelling imperative to develop innovative therapeutics that not only enhance efficacy but also minimize side effects, thereby leading to improved survival rates for individuals grappling with HCC.

In recent years, traditional Chinese medicine has garnered substantial attention due to its potential to mitigate the adverse effects of chemotherapy and reduce the development of drug resistance [[11], [12], [13]]. Among these traditional remedies, *Actinidia chinensis Planch Root extracts (acRoots)*, derived from the kiwi tree's root, has surfaced as a noteworthy contender [14]. *acRoots*'s primary constituents encompass triterpenes and flavonoids [15]. Research has underscored *acRoots*'s capacity to combat gastric cancer by orchestrating processes like apoptosis, ferroptosis, and modulating mesenchymal phenotype [16]. Furthermore, *acRoots* exhibits promise in curbing HCC proliferation and metastasis through the inhibition of the DLX2/TARBP2/JNK/AKT pathway [17]. While numerous studies have elucidated the anti-tumor effects of vine pear root, only a handful have delved into the specific anti-tumor mechanisms. Hence, an urgent need persists to investigate the precise biomolecular mechanisms and therapeutic targets associated with *acRoots*' anti-tumor effects. This exploration is paramount for the development of natural anti-cancer drugs and novel treatment avenues for the prevention and management of HCC.

Notably, AKT, a serine-threonine kinase, plays a pivotal role in mediating diverse biological functions encompassing cell proliferation, glucose metabolism, protein synthesis, genome stability, and suppression of apoptosis [18], all in response to growth factors and extracellular cues [18,19]. Dysregulated AKT signaling is a hallmark of various human cancers [20], fueling tumor aggressiveness and rendering resistance to therapies [20]. Notably, aberrant activation of the AKT/mTOR pathway afflicts nearly half of HCC patients [21,22]. However, it remains an enigma whether the AKT/mTOR signaling cascade interplays with *acRoots*'s role in suppressing HCC growth, warranting comprehensive exploration.

This study is undertaken with the explicit objective of dissecting the antitumor properties and molecular intricacies of *acRoots* within HCC cell lines. Our aim is to pave the way for the development of potential anticancer drugs, thereby revolutionizing the landscape of liver cancer prevention and treatment. The findings herein contribute to a deeper comprehension of *acRoots*'s anticancer mechanism, shedding light on its potential as an efficacious therapeutic agent for HCC treatment.

## 2 Materials and methods

### 2.1 Preparation of *acRoots* concentrated solution

To prepare the concentrated acRoots solution, 100g of acRoots were soaked in 300 ml of water, boiled for approximately 45 min, and then concentrated to 50 ml using a 60-degree constant temperature water bath, resulting in a concentrated solution with a concentration of 2 g/ml. This concentrated solution underwent filtration, followed by storage in a −80 °C refrigerator for one day. Subsequently, it was subjected to freeze-drying (Martin Christ Freeze Dryers, Osterode, Germany), ultimately transforming it into a lyophilized powder. This powder was securely stored in a −20 °C refrigerator for future use. To create a mother solution, the *acRoots* lyophilized powder was dissolved in double-distilled water, yielding a 100 mg/ml solution. This solution was further diluted with culture medium as needed.

In this study, ultra performance liquid chromatography-Triple time of flight mass spectrometry (UPLC-Triple-TOF/MS) was used to identify the chemical components in the roots of P. truncatula. Reverse-phase analysis was performed on a AcquityTM ARC system (Waters, USA) using an ACQUITY UPLC HSS T3 column (1.8 μm, 3.0 mm × 150 mm) (Waters, USA) containing a binary pump, a column oven, and an ESI ionization source. The flow rate was 0.3 mL/min, with mobile phase A composed of 0.1 % formic acid and mobile phase B composed of acetonitrile. A gradient elution achieved sample separation: 0–20 min, 2–15 % mobile phase B formic acid acetonitrile; 30–40 min, 50–95 % mobile phase B formic acid acetonitrile.

The column temperature was set to 50 °C, and each analysis utilized an injection volume of 3 μL. Prior to injection, the samples underwent filtration through a 0.22-μm ultrafiltration membrane.

Mass spectrometric analysis was conducted using a Triple TOF® 5600 System (AB SCIEX, USA) in both positive and negative ion modes. The source conditions were as follows: a spray voltage of 5500 V in ESI (+) and − 4500 V in ESI (−), nebulizing gas at 35 psi, heating gas at 55 psi, curtain gas™ at 35 psi, and heater temperature at 600 °C. The declustering potential was set to 100 V. Mass spectrometry was performed with a scan range of 100–1500 (*m*/*z*).

### 2.2 Cell culture and procurement of HepG2 and LM3 cells

Human hepatocellular carcinoma cells, namely HepG2 and LM3, were procured from iCell Bioscience Inc., located in Shanghai, China. HepG2 cells were cultivated in MEM (Nanjing BioChannel Biotechnology Co., Ltd, Nanjing, China), supplemented with 10 % fetal bovine serum obtained from the same source. As for LM3 cells, they were cultured in DMEM along with 10 % fetal bovine serum. Passage of the cells was conducted every two days using trypsin acquired from Nanjing BioChannel Biotechnology Co., Ltd in Nanjing, China.

### 2.3 Cell viability assessment using CCK-8 assay

HepG2 and LM3 cells were meticulously seeded in 96-well plates (Guangzhou Yike Biotechnology Co., Ltd, Guangzhou, China) at a density of 5 × 10^3^ cells per well. Subsequently, hepatocellular carcinoma (HCC) cells were exposed to varying concentrations of acRoots solutions (0 μg/ml, 200 μg/ml, 450 μg/ml, and 600 μg/ml), with each well containing 200ul of the solution. After 24 h, 10 μl of CCK-8 solution (MedChemExpress, Shanghai, China) was introduced into each well. Subsequently, the cells underwent incubation at 37 °C in a humidified atmosphere enriched with 5 % CO_2_ for a duration of 2 h. To gauge cell viability, the optical density at 450 nm was meticulously gauged employing a microplate reader sourced from Thermo Fisher Scientific, based in Massachusetts, USA.

### 2.4 Colony formation observation after *acRoots* treatment in HepG2 and LM3 cells

HepG2 and LM3 cells were meticulously seeded into six-well plates, each well initially containing 500 cells. These cells were then treated with 1 ml of acRoots solution (0 μg/ml, 200 μg/ml, 450 μg/ml, and 600 μg/ml) for a 24-h period. After a span of 14 days, the cells were fixed with methanol for a duration of 20 min. Subsequently, they were stained with a 0.05 % crystal violet solution, facilitating the examination and observation of colony formation.

### 2.5 Assessment of cell proliferation using the EdU cell proliferation kit

To evaluate cell proliferation, the EdU Cell Proliferation Kit sourced from Beyotime in Shanghai, China was employed. In this protocol, appropriate cells were initially seeded into 6-well plates and treated for 24 h with 1 ml of acRoots solution (0 μg/ml, 200 μg/ml, 450 μg/ml, and 600 μg/ml). Subsequently, EdU (20 μM) was introduced into each well and allowed to incubate for 2 h. Following this, the cells were fixed using a 4 % paraformaldehyde solution and permeabilized with phosphate-buffered saline (PBS) containing 0.3 % Triton X-100. Next, a 0.5 mL aliquot of Click additive solution was applied to the cells and left to incubate for 30 min. Finally, the nuclei were stained with Hoechst (1:1000) for 10 min in the absence of light. The resultant fluorescence was visualized using a fluorescence microscope.

### 2.6 Assessment of cell invasion using transwell chambers after *acRoots* treatment

Following a 24-h treatment, the cells were exposed to 500 μl of acRoots solution (0 μg/ml, 200 μg/ml, 450 μg/ml, and 600 μg/ml), HepG2 and LM3 cells were transferred to the upper chamber of Transwell inserts (Beijing Lanjieko Technology Co., Ltd, Beijing, China). Simultaneously, the lower chamber received culture medium containing 10 % fetal bovine serum (FBS) and was incubated at 37 °C for an additional 24 h. Subsequently, any matrix or cells that did not traverse the membrane surface were meticulously removed and washed three times with phosphate-buffered saline (PBS). Following this, the cells were fixed using methanol sourced from Shanghai Linen Technology Development Co., Ltd, Shanghai, China, for a duration of 20 min. A subsequent triple rinse with double-distilled water ensued. After drying, the cells were stained with a 0.1 % crystal violet staining solution for 20 min. The cell invasion was ultimately observed under a microscope.

### 2.7 Autophagy detection using MDC staining after *acRoots* treatment

To detect autophagy, MDC (monodansylcadaverine) staining was employed as a marker for autophagic vesicles. After a 24-h treatment with 1 ml of acRoots solution (0 μg/ml, 200 μg/ml, 450 μg/ml, and 600 μg/ml), both HepG2 and LM3 cells underwent subsequent procedures. HepG2 and LM3 cells were subjected to MDC staining, following the manufacturer's instructions (Beyotime, Shanghai, China). The procedure involved aspirating the medium, rinsing the cells once with PBS, and subsequently adding 1 ml of MDC staining solution to each well. The cells were then incubated in the dark at 37 °C, within a humidified atmosphere containing 5 % CO_2_, for a period of 30 min. Subsequently, the cells were placed under a fluorescent microscope and excited with ultraviolet excitation light to visualize the green fluorescence, which was captured in photographs.

### 2.8 Apoptosis assessment in HepG2 and LM3 cells after *acRoots* treatment

After a 24-h treatment with 1 ml acRoots solution (0 μg/ml, 200 μg/ml, 450 μg/ml, 600 μg/ml), both HepG2 and LM3 cells were subjected to subsequent procedures. The medium was first removed, and the cells were diligently collected. They were then sequentially stained with 5 μL Annexin V-FITC and 5 μL propidium iodide, with reagents sourced from Beyotime in Shanghai, China. This staining process took place at 4 °C for a duration of 30 min, conducted in complete darkness. Finally, the number of apoptotic cells was quantified utilizing flow cytometry.

### 2.9 Immunofluorescence analysis of cell cultures following *acRoots* treatment

Upon reaching a cellular density of 60%–80 % confluence on coverslips, the cells were subjected to a 24-h treatment with 1 ml of acRoots solution (0 μg/ml, 200 μg/ml, 450 μg/ml, and 600 μg/ml). Subsequently, the cells were fixed using 4 % paraformaldehyde for a duration of 15 min, followed by a blocking step using goat serum for 30 min at room temperature. The coverslips were then subjected to an overnight incubation at 4 °C with the primary antibody. Following three washes with phosphate-buffered saline (PBS), the cells were further incubated with secondary antibodies, and their nuclei were counterstained with DAPI. All images were captured using an inverted fluorescence microscope sourced from Beijing Lanjieko Technology Co., Ltd, Beijing, China.

### 2.10 Protein extraction and Western blot analysis procedure

For protein extraction, RIPA lysis buffer from Beyotime in Shanghai, China was employed. The total protein concentration within the extracts was quantified using a BCA kit, also from Beyotime, Shanghai, China. Subsequently, proteins within the samples were separated through SDS-PAGE (Sodium Dodecyl Sulfate-Polyacrylamide Gel Electrophoresis), using gels of varying concentrations (8 %, 10 %, or 12 %) depending on their molecular weights. These separated proteins were then transferred onto PVDF membranes sourced from MILLIPORE, located in Massachusetts, USA. To minimize non-specific binding, the PVDF membranes underwent a blocking step with skim milk for 2 h. Following this, they were incubated overnight at 4 °C with primary antibodies obtained from Proteintech Group Inc., based in Chicago, USA. Afterward, the PVDF membranes were thoroughly washed three times with PBST (Phosphate-Buffered Saline with Tween-20), a solution procured from Nanjing BioChannel Biotechnology Co., Ltd, NanJing, China. Subsequently, a secondary antibody was employed for a 2-h incubation, and a final round of washing followed.

### 2.11 Statistical analysis and software utilized

Statistical analyses were conducted employing SPSS version 22.0 (SPSS). Variations in the expression levels derived from the invasion assay were assessed via the Mann-Whitney *U* test. In vitro experiments were meticulously conducted in triplicate and independently repeated at least three times to ensure robustness and reliability. All data are expressed as mean ± standard error of the mean (SEM). Statistical analyses were additionally performed using GraphPad Prism 9, with statistical significance defined as a P-value less than 0.05.

## 3 Results

### 3.1 Assessment of *acRoots*' impact on HCC cell viability and proliferation

In this study, a total of 24 phytochemicals were identified using UPLC-Triple-TOF/MS technology. Among them, Fraxin, loganic acid, chlorogenic acid, 9,12,13-Trihydroxy-10-trans-octadecenoic acid, citric acid, and 9(S),12(S),13(S)-trihydroxyoctadeca-10(E),15(Z)-dienoic acid were identified as the most abundant compounds (Table 1).

**Table 1:** Chemical composition of acRoots.

Number	Compound	Formula
1	fraxin	C16H18O10
2	D-catechin	C15H14O6
3	procyanidin dimer B2	C30H26O12
4	loganic acid	C16H24O10
5	chlorogenic acid	C16H18O9
6	9,12,13-Trihydroxy-10-trans-octadecensaeure	C18H34O5
7	citric acid	C6H8O7
8	vanillate glucoside	C14H18O9
9	9(S),12(S),13(S)-trihydroxyoctadeca-10(E),15(Z)-dienoic acid	C18H32O5
10	glucosyringic acid	C15H20O10
11	N-Acetyl-2,3-didehydro-2-deoxyneuraminic Acid	C11H17NO8
12	protocatechuic acid-*O*-hexoside	C13H16O9
13	akebia saponin D	C47H76O18
14	5-carboxystrictosidine	C28H34N2O11
15	isochlorogenic acid C	C25H24O12
16	(6*R*,3*E*)-1-*O*-β-d-glucopyranosyl-6,7-dihydroxy-3,7-dimethyl-2-octenoate	C16H28O9
17	4-coumaroylquinic acid	C16H18O8
18	sweroside	C16H22O9
19	*trans*-3-*p*-coumaroylquinic acid	C16H18O8
20	procyanidin C1	C45H38O18
21	3-*O*-β-d-glucopyranosyl platycodigenin	C36H58O12
22	hesperidin	C28H34O15
23	nigaichigoside F1	C36H58O11
24	apigenin 7-glucuronide	C21H18O11

**Fig. 1:**
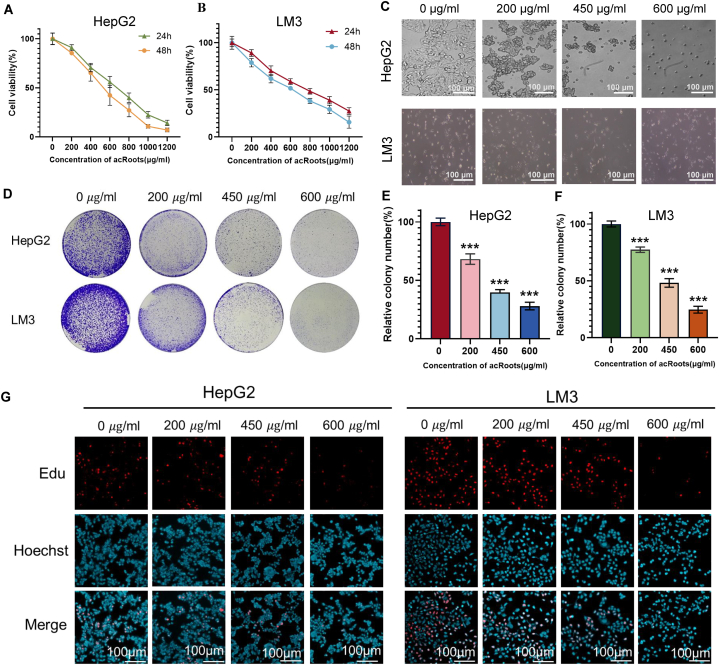
Effects of *acRoots* on HCC cell viability, morphology, colony formation, and proliferation **A-B.** The CCK-8 assay demonstrates a significant reduction in cell viability induced by *acRoots*. **C.** The influence of *acRoots* on cell morphology is readily apparent. **D-F.***acRoots* hinders the colony formation ability of HCC cells. **G.***acRoots* effectively restrains the proliferation of HCC cells. * means P-value <0.05, ** means P-value <0.001, *** means P-value <0.0001.

In an initial evaluation of Hepatocellular Carcinoma (HCC) cell tolerance to *acRoots*, HepG2 and LM3 cells were exposed to varying concentrations of *acRoots* solutions (0, 50, 100, 200, 400, 600, 800, and 1200 μg/ml). Cell viability was assessed after 24 and 48 h using the Cell Counting Kit-8 (CCK-8). The results revealed that low concentrations of *acRoots* had no significant impact on the viability of HepG2 and LM3 cells. As the concentration increased, the inhibitory effect gradually intensified, with high-concentration *acRoots* solutions exerting a potent toxic influence on the cells (Fig. 1A–B). Consequently, we opted for four concentration groups (0, 200, 450, and 600 μg/ml) for subsequent experiments. Furthermore, HCC cells were treated with these concentrations for 24 h to observe the toxic effect of *acRoots* on hepatocellular carcinoma. The findings indicated distinct morphological changes in HepG2 cells, especially within the 600 μg/ml group, where some cells displayed fragmentation. In contrast, LM3 cell morphology remained largely unchanged (Fig. 1C). To further substantiate *acRoots*' tumor-suppressing potential, we assessed the cells' colony-forming ability, which was notably inhibited by *acRoots* (Fig. 1D–F). Additionally, the EdU proliferation assay confirmed *acRoots*' inhibitory effect on HCC cell proliferation. Collectively, these results underscored *acRoots*' dose-dependent inhibition of HCC cell proliferation (Fig. 1G).

### 3.2 Effect of *acRoots* on HCC cell migration and Epithelial-mesenchymal Transition (EMT)-related pathways

**Fig. 2:**
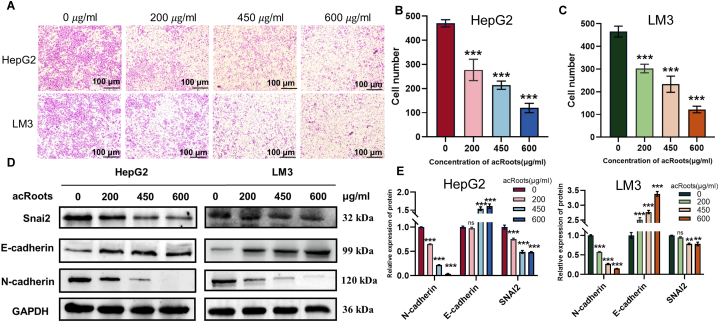
Impact of *acRoots* on HCC cell migration and molecular pathways **A.** Transwell experiments conclusively affirm *acRoots*' capacity to inhibit the migratory ability of HCC cells. **B–C.** Quantification of cells that successfully traversed the transwell membrane, presented through a histogram. **D.** Western blot experiments offer insights into *acRoots*' potential to hinder the migration of HCC cells by modulating N-cadherin and Vimentin expression while promoting *E*-cadherin expression. Complete, unadjusted images are included in the supplemental material. **E.** Compilation and analysis of Western blot results. * means P-value <0.05, ** means P-value <0.001, *** means P-value <0.0001.

To investigate the impact of *acRoots* on the migratory capability of HCC cells, we conducted a transwell assay. As depicted in Fig. 2A, the migration ability of both LM3 and HepG2 cells was notably diminished following a 24-h treatment with various concentrations of *acRoots* solutions. Moreover, we quantified the number of cells that successfully traversed the transwell membrane (Fig. 2B and C). In addition to this, Western blot experiments shed light on *acRoots*' role in inhibiting EMT-related pathways. This inhibition was reflected in the downregulation of N-cadherin (N-cad) and Snail2 (Snai2) and the concomitant upregulation of *E*-cadherin (E-cad) expression (Fig. 2D and E). These findings collectively underscore the potential of *acRoots* in curtailing HCC cell migration and modulating EMT-related signaling pathways.

### 3.3 Impact of *acRoots* on autophagy and apoptosis in HCC cells

**Fig. 3:**
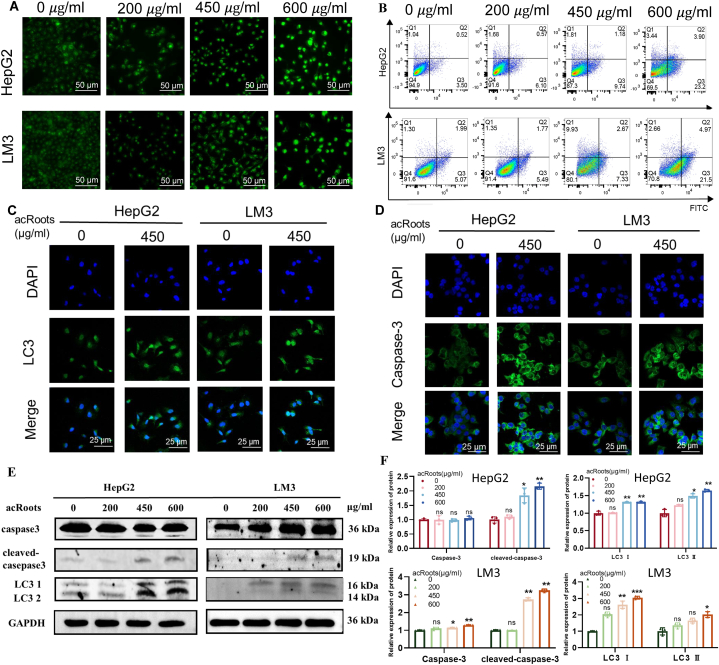
*AcRoots*-induced autophagy and apoptosis in HCC cells: evidence from immunofluorescence and Western blot analyses **A.***acRoots* demonstrates the capability to induce autophagy in HCC cells. **B.***acRoots* exhibits the potential to induce apoptosis in HCC cells. **C-D.** A comparative analysis of LC3 and Caspase-3 levels conducted via immunofluorescence. ***E*-F.** A quantitative evaluation of Caspase-3, cleaved Caspase-3, LC3Ⅰ, and LC3Ⅱ levels through Western blotting. Complete, unadjusted images are included in the ssupplemental material. * means P-value <0.05, ** means P-value <0.001, *** means P-value <0.0001.

To investigate the influence of *acRoots* on autophagy and apoptosis in HCC cells, we initially exposed HepG2 and LM3 cells to various concentrations of *acRoots* solutions for 24 h. Subsequently, we evaluated autophagy using MDC staining. As depicted in Fig. 3A, an increase in *acRoots* concentration correlated with a gradual rise in fluorescence intensity, indicative of enhanced cellular autophagy. Following this, we employed flow cytometry to assess cell apoptosis. The results demonstrated that *acRoots* significantly induced apoptosis in HCC cells in a dose-dependent manner. Specifically, the proportion of apoptotic HepG2 cells increased by 19.7 %, while that of LM3 cells increased by 16.43 % (Fig. 3B). Confocal analysis revealed intracellular accumulation of LC3 (an autophagy marker) and Caspase-3 (an apoptosis marker) upon treatment with *acRoots* solution in HepG2 and LM3 cells (Fig. 3C–D). Western blot results further corroborated these findings, illustrating dose-dependent upregulation of LC3Ⅰ, LC3Ⅱ, Caspase-3, and cleaved Caspase-3 in *acRoots*-treated cells (Fig. 3*E*–F). Collectively, these observations highlight *acRoots*' role in modulating autophagy and apoptosis pathways in HCC cells.

### 3.4 *AcRoots* modulates HCC cell apoptosis and autophagy via the AKT/mTOR pathway

**Fig. 4:**
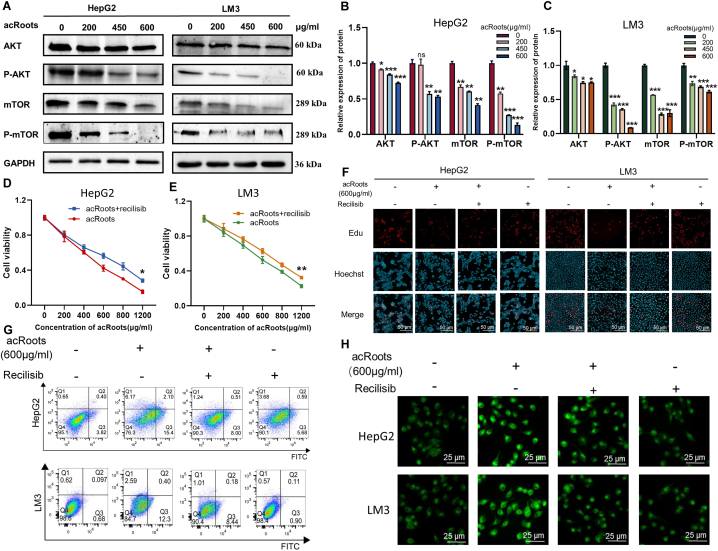
*AcRoots* promotes cancer progression by blocking the AKT/mTOR pathway in HCC cells **A-C.***AcRoots* down-regulates AKT, *p*-AKT, mTOR, and *p*-mTOR in HCC Cells. Complete, unadjusted images are included in the ssupplemental material. **D-E.** Recilisib reverses HCC cell viability after 24-h pretreatment. **F.** Recilisib restores HCC cell proliferation. **G.** Recilisib decreases HCC cell apoptosis. **H.** Recilisib reduces autophagy in HCC cells. * means P-value <0.05, ** means P-value <0.001, *** means P-value <0.0001.

The AKT/mTOR pathway is well-established as a critical signal transduction cascade that exerts inhibitory effects on cellular autophagy and apoptosis [23]. Therefore, we sought to elucidate *acRoots*' influence on the AKT/mTOR pathway. Western blot results demonstrated that *acRoots* dose-dependently downregulated phosphorylated AKT, mTOR, and phosphorylated mTOR levels in both HepG2 and LM3 cells. Notably, it did not impact AKT expression in LM3 cells (Fig. 4A–C).

Furthermore, to investigate whether *acRoots* regulates apoptosis and autophagy in HCC cells by suppressing the AKT/mTOR pathway, we pre-treated cells with Recilisib, an AKT activator, for 24 h prior to *acRoots* treatment. Subsequently, we assessed cell viability. As depicted in Fig. 4D–E, the AKT activator restored cell viability. This effect was further corroborated by colony formation and EdU proliferation assays, confirming the capacity of AKT activators to rescue cell proliferation (Fig. 4F). Additionally, we evaluated cell apoptosis and autophagy, revealing that AKT activators attenuated cell apoptosis and autophagy to varying degrees following their administration (Fig. 4G–H). In summary, AKT activators exhibited the ability to reverse the antitumor effects of *acRoots*. These findings collectively underscore *acRoots*'s capacity to induce apoptosis and autophagy in HCC cells through the regulation of the Akt/mTOR pathway.

## 4 Discussion

*AcRoots*, a traditional Chinese medicine, has garnered attention for its reported efficacy in inhibiting breast cancer cell proliferation, migration, and invasion, while promoting apoptosis in these cells. This action has been attributed to its ability to impede the AKT/GSK-3β pathway, evidenced by reductions in *p*-AKT, *p*-GSK-3β, and β-catenin levels, leading to suppression of breast cancer cell proliferation, migration, invasion, and induction of apoptosis [24]. *AcRoots* has also demonstrated its tumor-suppressing potential in nasopharyngeal carcinoma by inhibiting the E2F1-mediated MNX1-AS1 expression, thus hindering cell proliferation and metastasis [25]. Furthermore, the ethanol extract of *acRoots* has shown promise in inhibiting tumor angiogenesis, promoting colon cancer cell apoptosis, and inhibiting tumor growth [26,27]. In our study, we elucidate that *acRoots* exerts its anticancer effects by dose-dependently inhibiting the proliferation, migration, and invasion of hepatocellular carcinoma (HCC) cells. In a groundbreaking revelation, this study demonstrates, for the very first time, that vine pear root induces cell apoptosis and autophagy in liver cancer cells by inhibiting the AKT/mTOR signaling pathway. Moreover, our conclusions have been rigorously validated through a comprehensive array of experiments, including EDU cell proliferation assays, immunofluorescence studies, and flow cytometry analyses. This multifaceted approach strengthens the robustness of our findings and underscores the significance of our research from various angles.

Apoptosis serves as a pivotal indicator of cell death and holds relevance in tumor development [[28], [29], [30]]. Excessive autophagy can lead to apoptosis and subsequent cell demise, representing a mechanism of autophagic cell death in malignancies and drug-resistant tumor cells [31,32]. In our investigations, we observed that *acRoots* induced both autophagy and apoptosis in HepG2 and LM3 cells, significantly elevating the levels of Caspase-3, cleaved Caspase-3, and LC3.

The AKT/mTOR signaling pathway is a key regulator of apoptosis and autophagy in cancer cells [[33], [34], [35]]. Targeting this pathway presents a crucial therapeutic strategy across various tumors, enhancing tumor cell chemosensitivity while mitigating drug resistance [35,36]. Our study further explored the interplay between *acRoots*-induced apoptosis and autophagy within this signaling pathway, revealing that *acRoots* downregulated *p*-AKT and *p*-mTOR levels. Notably, AKT activators effectively restored HCC cell proliferation while concurrently reducing apoptosis and autophagy.

In summary, *acRoots* emerges as a natural compound with potential anticancer properties, acting through modulation of the AKT/mTOR signaling pathway. This research may provide a novel avenue for hepatocellular carcinoma treatment. Nonetheless, future investigations should delve into the specific interactions between *acRoots* and genes within the AKT signaling pathway.

## Funding

This study was supported by the Medicine and Health Science and Technology Plan Projects in Zhejiang Province (NO:2020RC108) and 10.13039/100007834Ningbo Natural Science Foundation (NO: 202003N4211).

## Inclusion and diversity

We support inclusive, diverse, and equitable conduct of research.

## Data availability statement

Data will be made available on request.

## Ethics declarations

Informed consent was not required for this study because animal and human experiments were not involved in this study.

## CRediT authorship contribution statement

**Kaijie Qiu:** Writing – original draft, Conceptualization. **Qing Xia:** Writing – review & editing, Writing – original draft, Conceptualization. **Hao Chen:** Writing – original draft, Formal analysis, Data curation. **Qiong Ye:** Writing – original draft, Formal analysis, Data curation. **Haixiang Mao:** Writing – original draft, Formal analysis, Data curation. **Mei Tian:** Methodology. **Yichao Gan:** Methodology. **Qinyuan Huang:** Methodology. **Haibiao Wang:** Software, Methodology, Formal analysis, Data curation. **Shiwei Duan:** Writing – review & editing, Software, Formal analysis, Data curation.

## Declaration of competing interest

The authors declare no competing interests.
