# Supplementary material for: Exploring the anticancer potential of Actinidia chinensis Planch root extracts (acRoots) on hepatocellular carcinoma: A molecular mechanism study
Source: Heliyon. 2023 Nov 2;9(11):e21851. doi: 10.1016/j.heliyon.2023.e21851 (PMC10656260; doi:10.1016/j.heliyon.2023.e21851)
Supplement: Multimedia component 1 [file mmc1.docx]

**supplementary material**

**Figure 2D**

| HepG2 - E-Cad | Page 2 |
| --- | --- |
| HepG2 - GAPDH | Page 2 |
| HepG2 - N-Cad | Page 3 |
| HepG2 - SNAI2 | Page 4 |
| LM3 - E-Cad | Page 4 |
| LM3 - GAPDH | Page 5 |
| LM3 - SNAI2 | Page 5 |
| LM3 - N-Cad | Page 6 |

**Figure 3E**

| HepG2 - GAPDH | Page 6 |
| --- | --- |
| HepG2 - cleaved caspase-3 and caspase-3 | Page 7 |
| HepG2 - LC3Ⅰand LC3Ⅱ | Page 7 |
| LM3 - GAPDH | Page 8 |
| LM3 - cleaved caspase-3 and caspase-3 | Page 8 |
| LM3 - LC3Ⅰand LC3 Ⅱ | Page 9 |

**Figure 4A**

| HepG2 - AKT | Page 9 |
| --- | --- |
| HepG2 - GAPDH | Page 10 |
| HepG2 - mTOR | Page 10 |
| HepG2 - P-AKT | Page 11 |
| HepG2 - P-mTOR | Page 11 |
| LM3 - GAPDH | Page 12 |
| LM3 - AKT | Page 12 |
| LM3 - mTOR | Page 13 |
| LM3 - P-AKT | Page 13 |
| LM3 - mTOR | Page 14 |

**Figure 2D**

**HepG2**

E-Cad


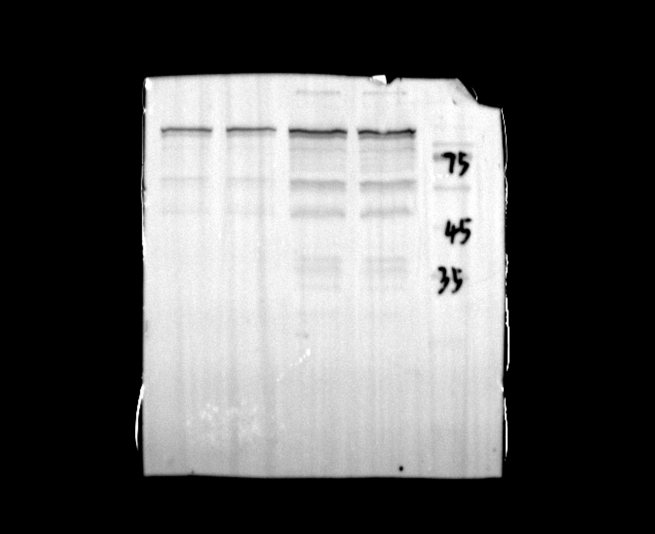


GAPDH


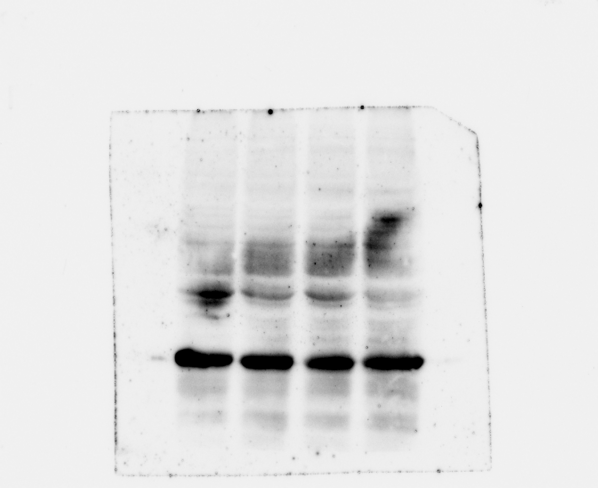


GAPDH_marker


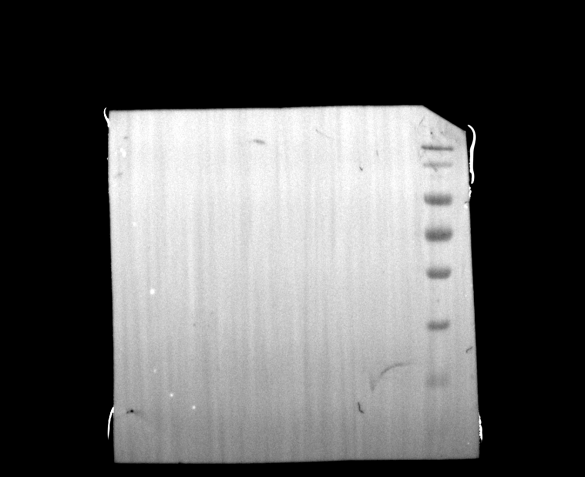


N-Cad


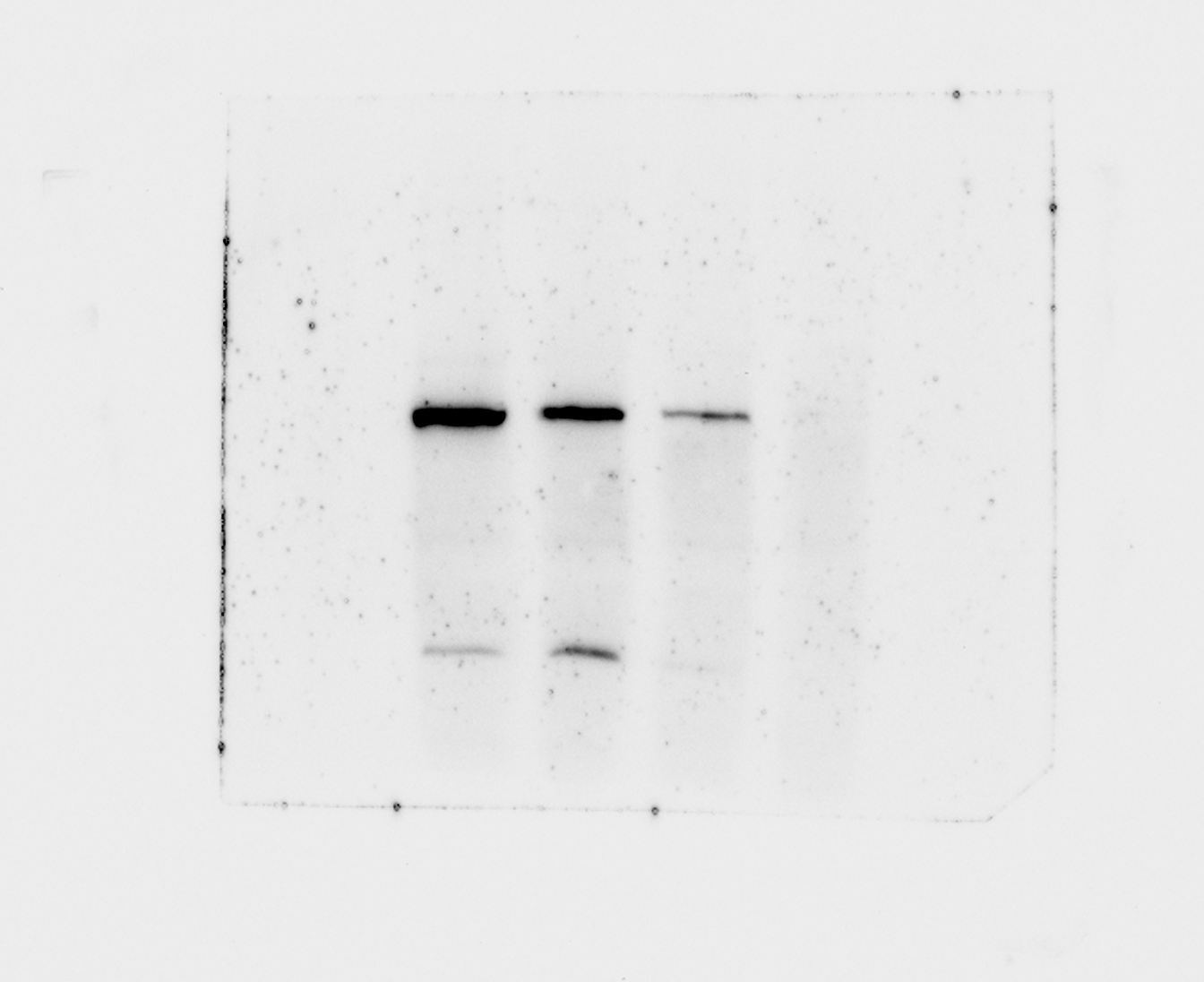


N-Cad_marker


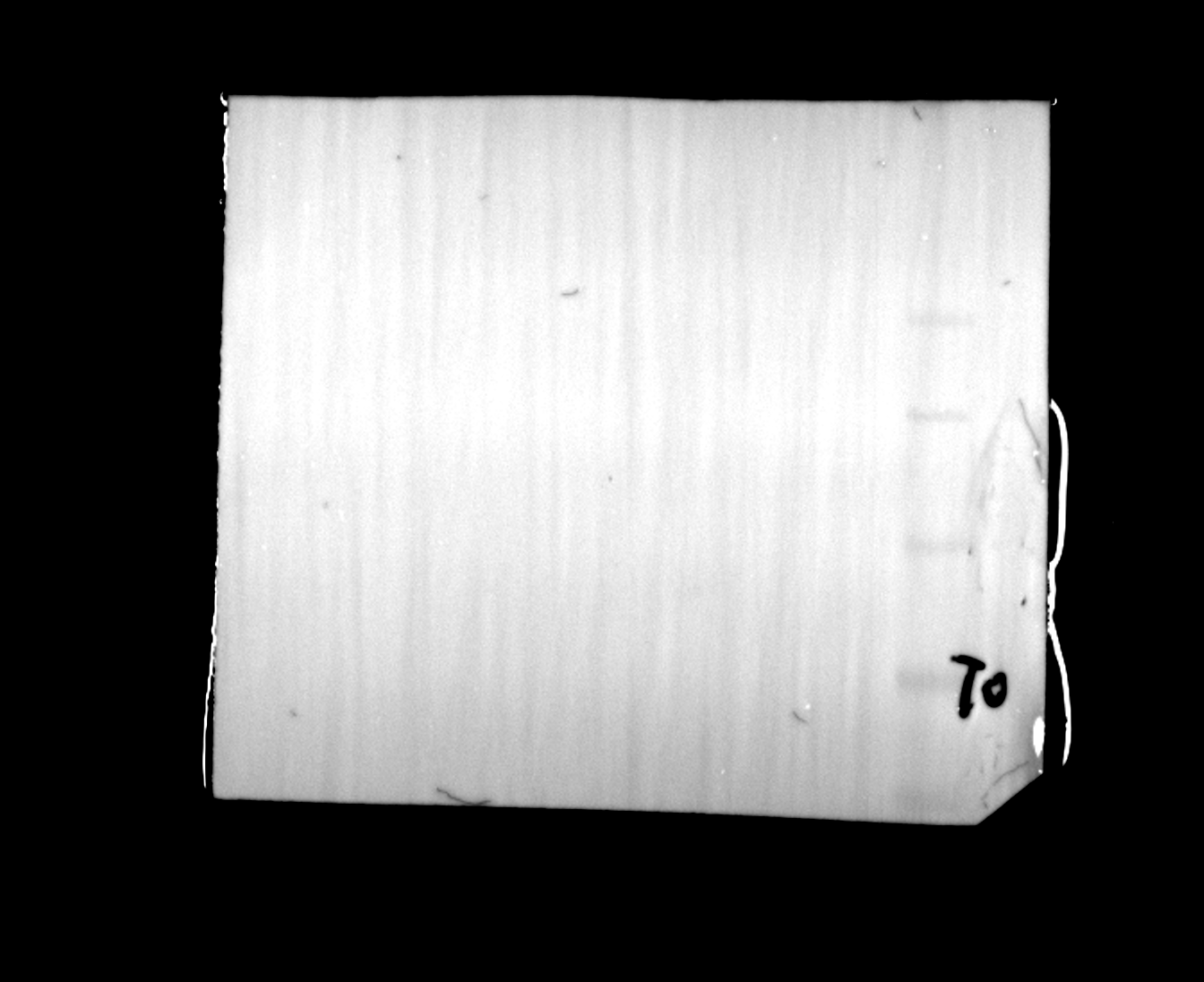


SNAI2


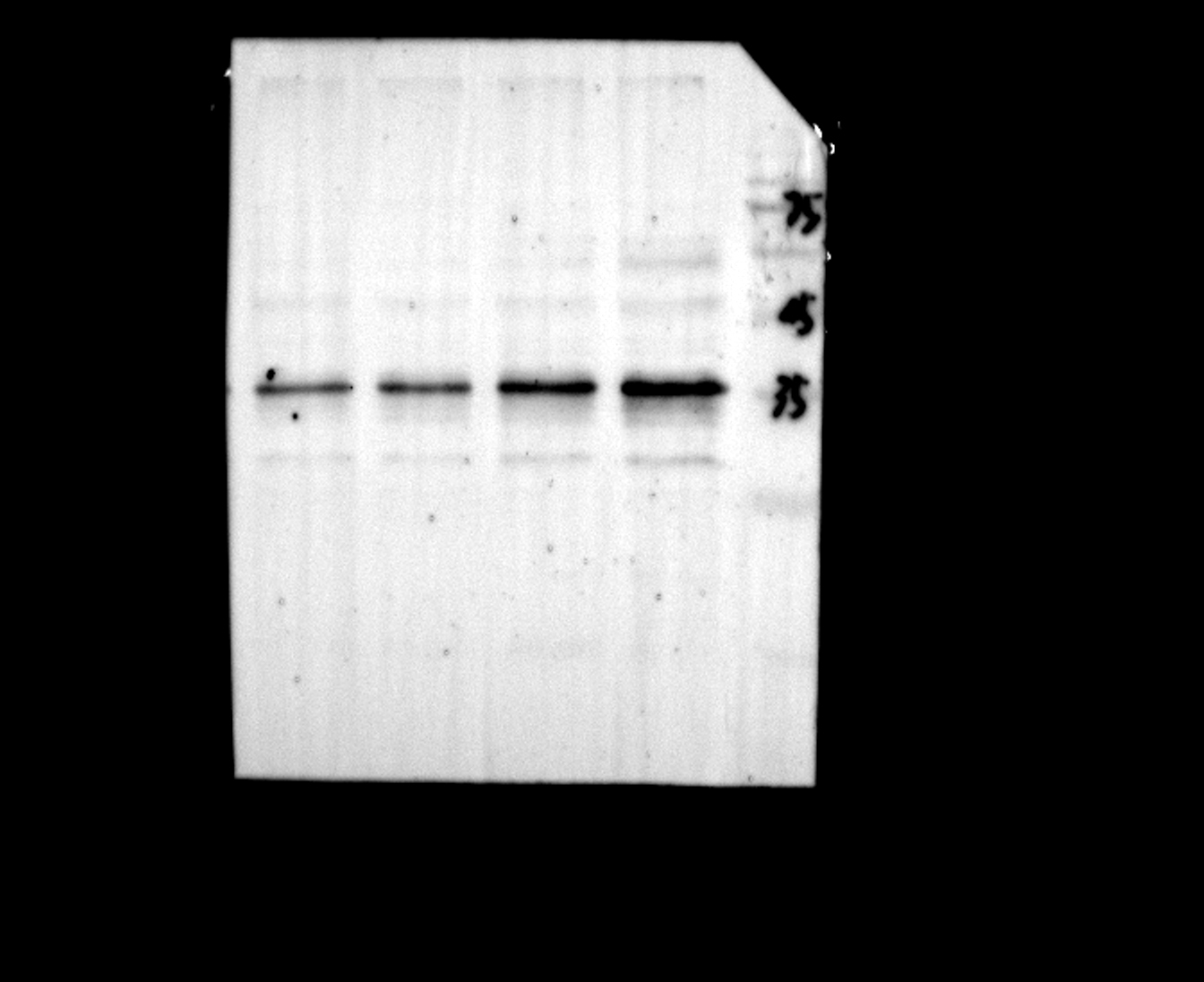


**LM3**

E-cad


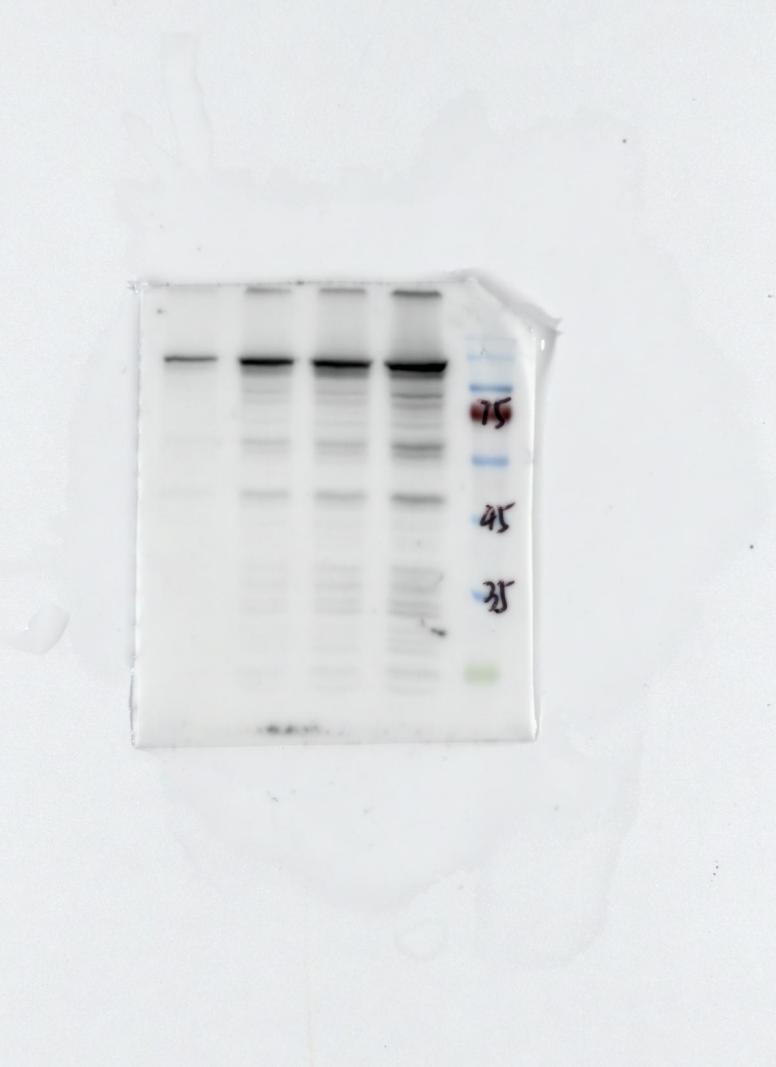


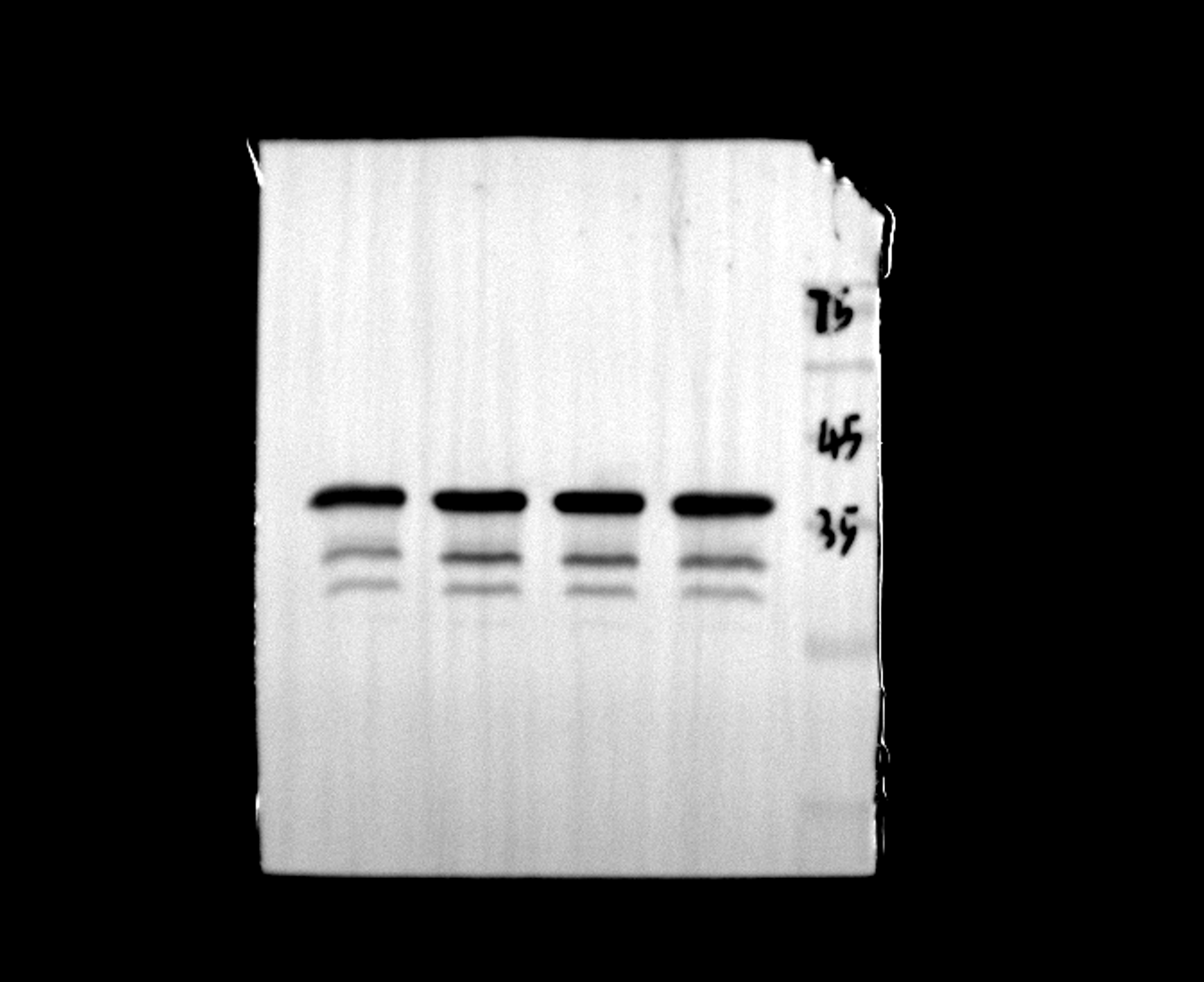
GAPDH

SNAI2


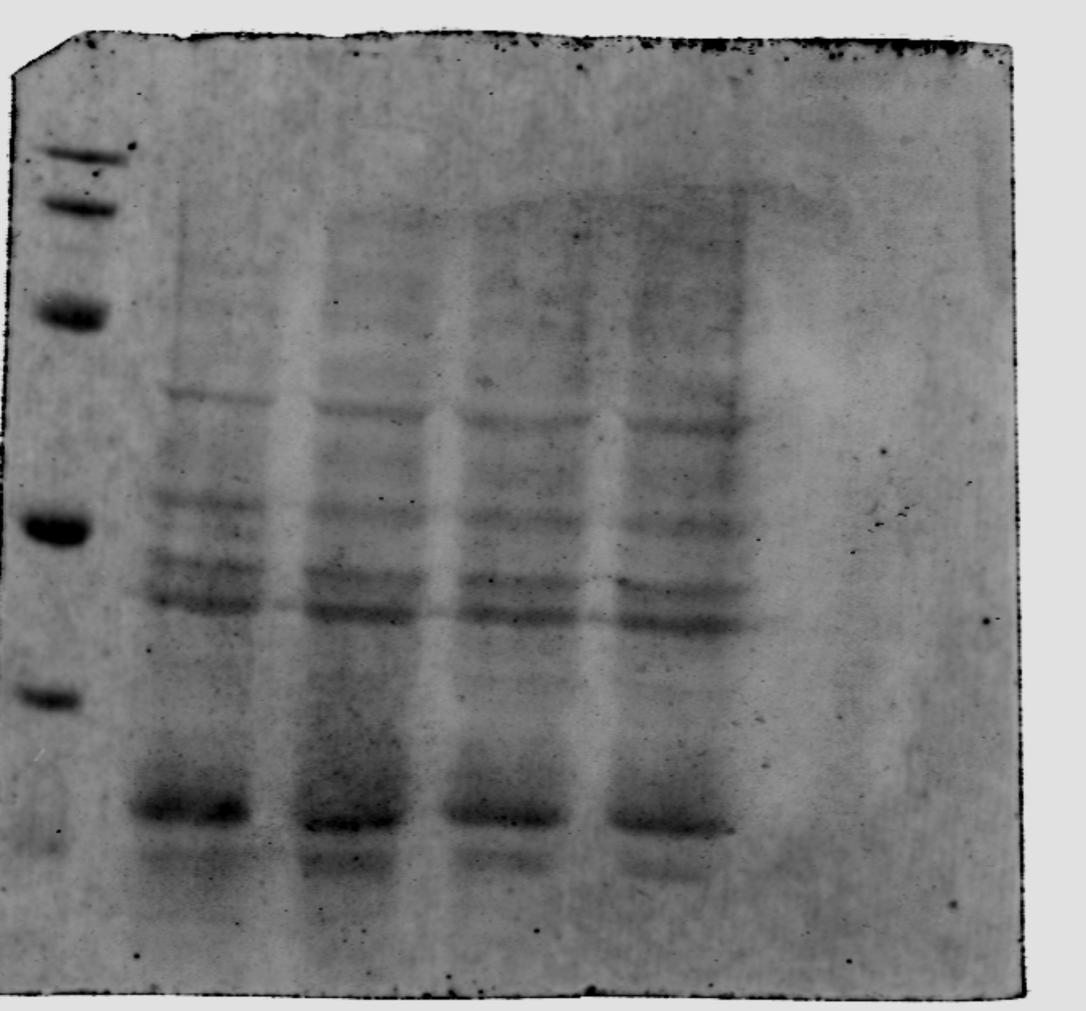


N-cad


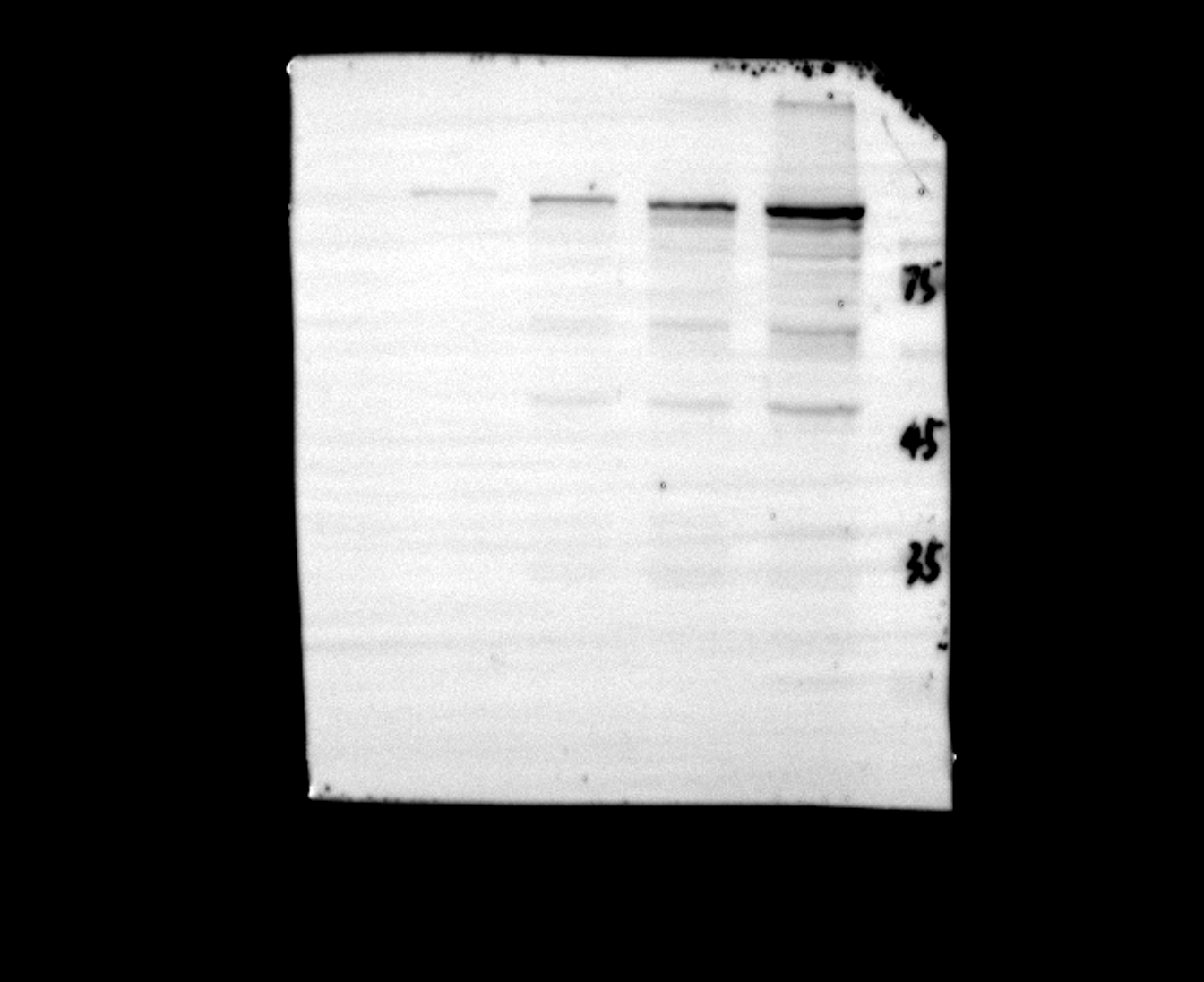
**Figure 3E**

**HepG2**

GAPDH


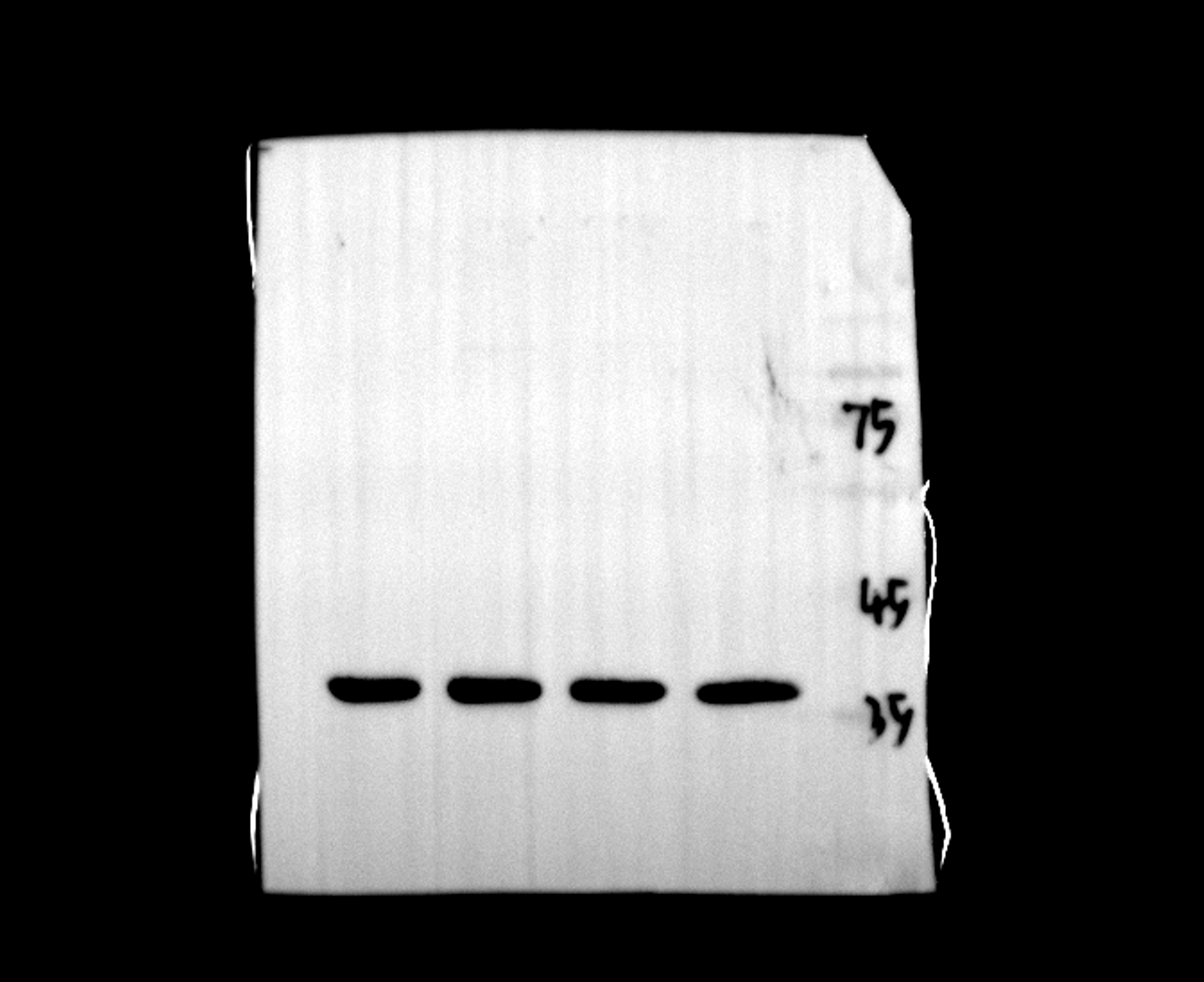


cleaved caspase-3 and caspase-3


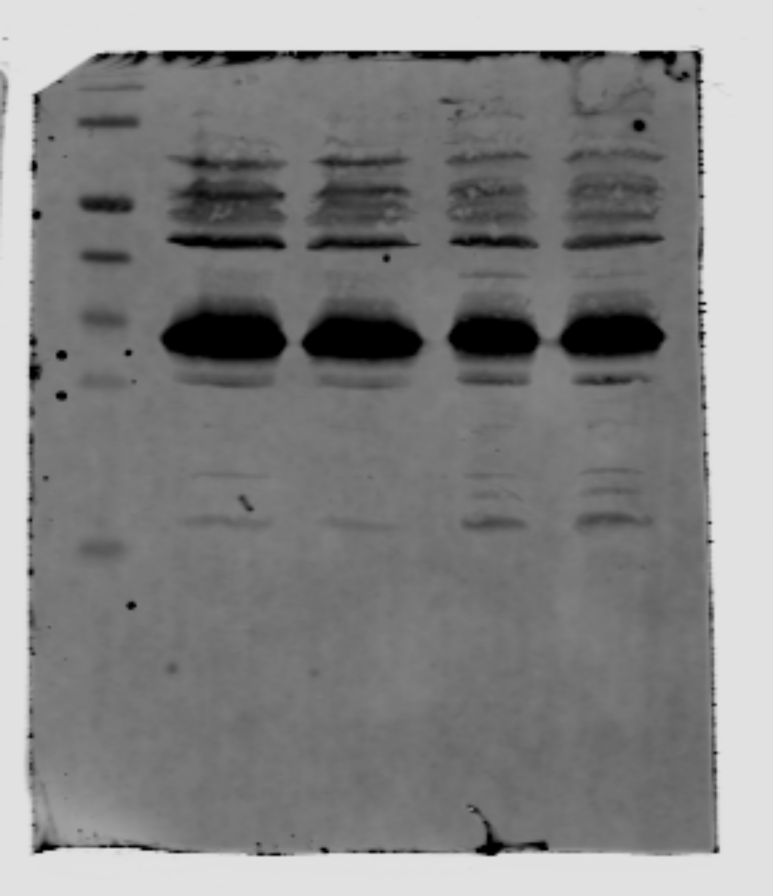


LC3 I and LC3 II


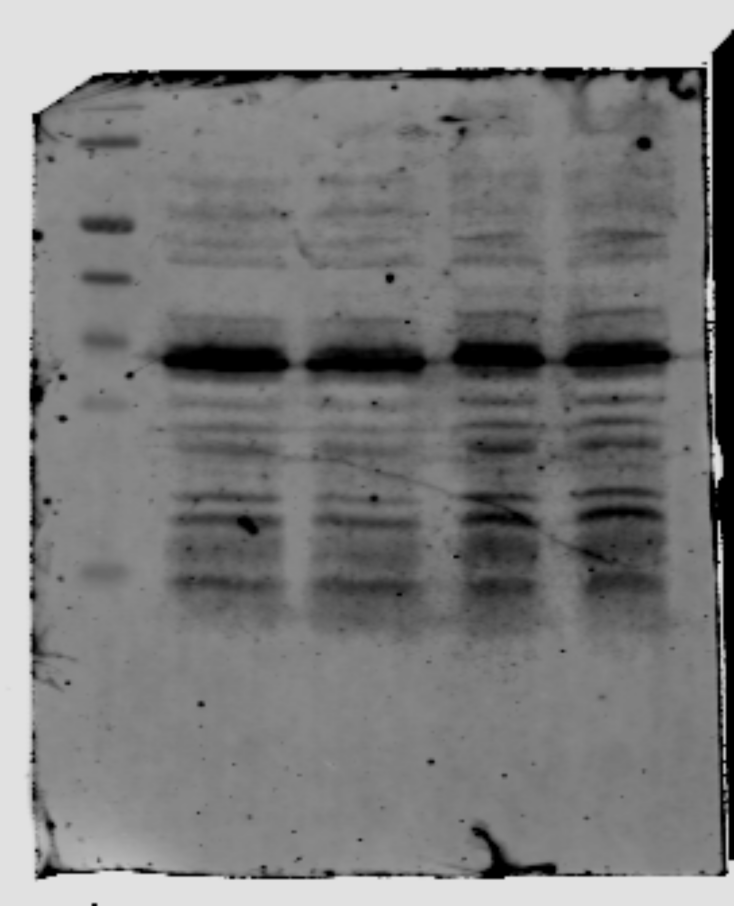


**LM3**

GAPDH

**
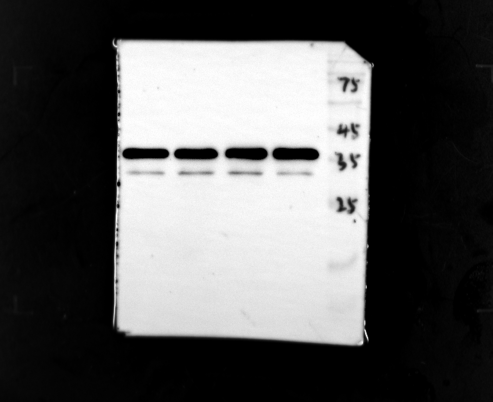
**

cleaved caspase-3 and caspase-3

**
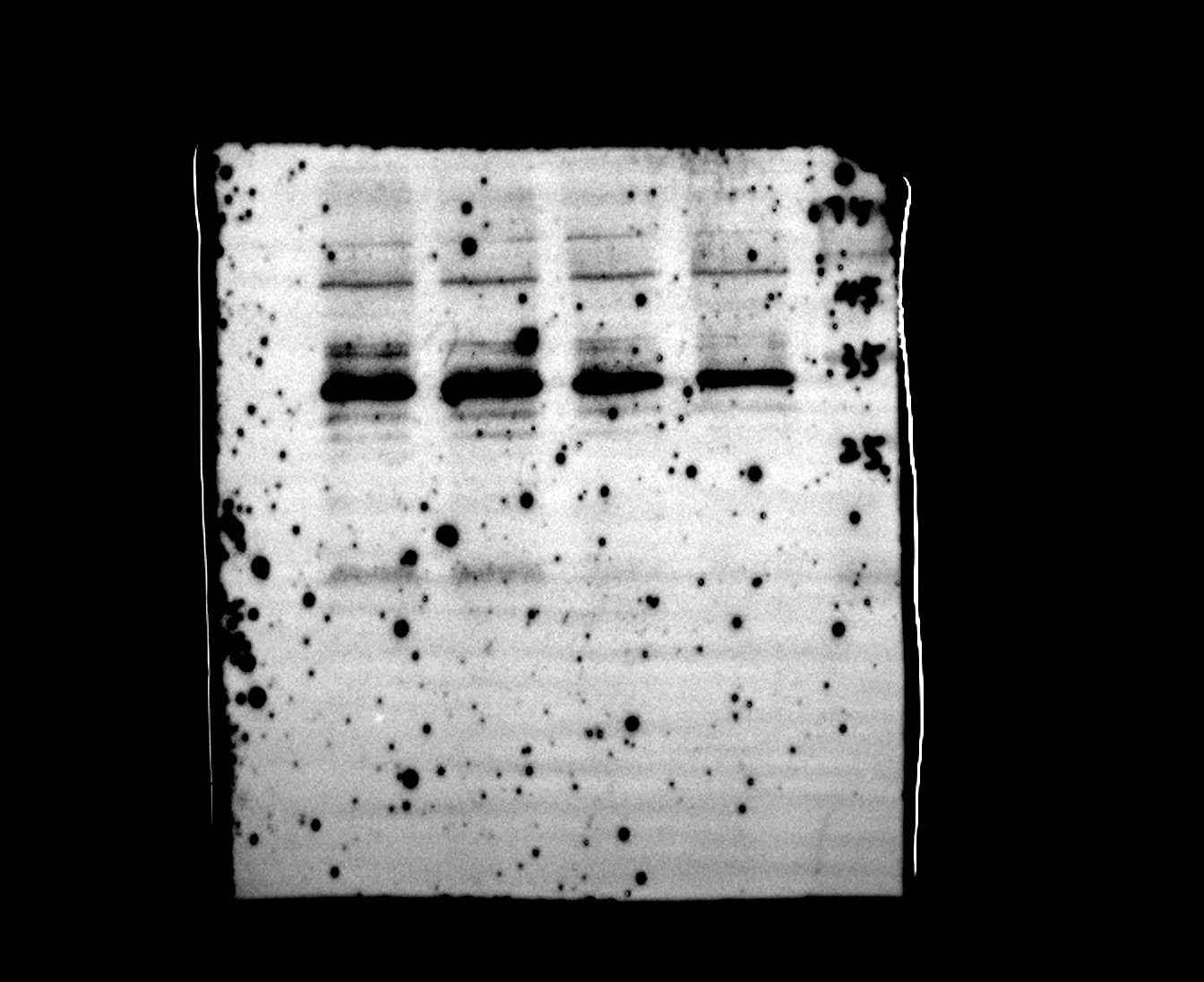
**

LC3I and LC3 II

**
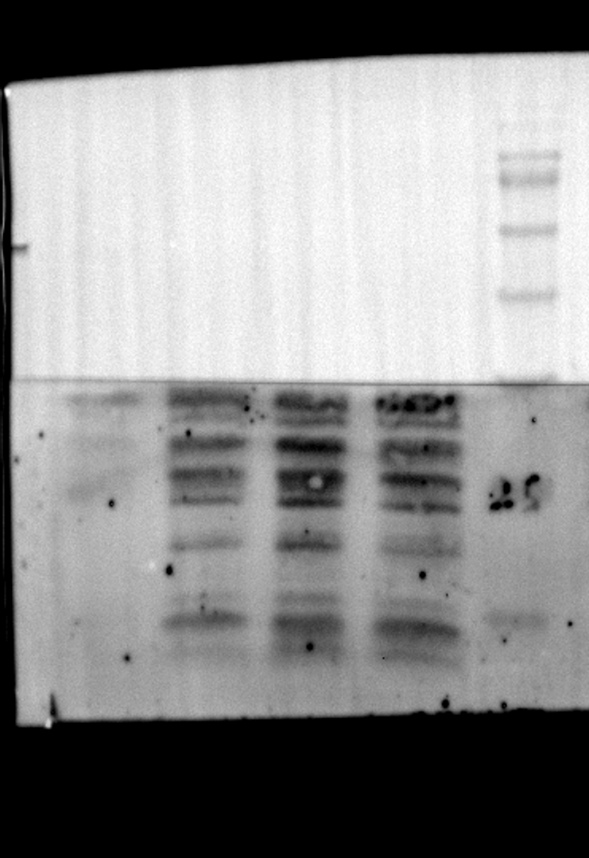
**

**Figure 4A**

**HepG2**

AKT

**
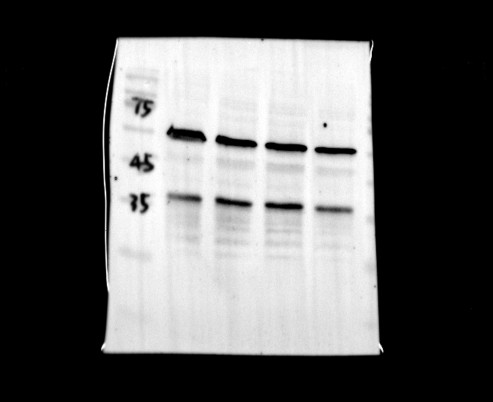
**

GAPDH


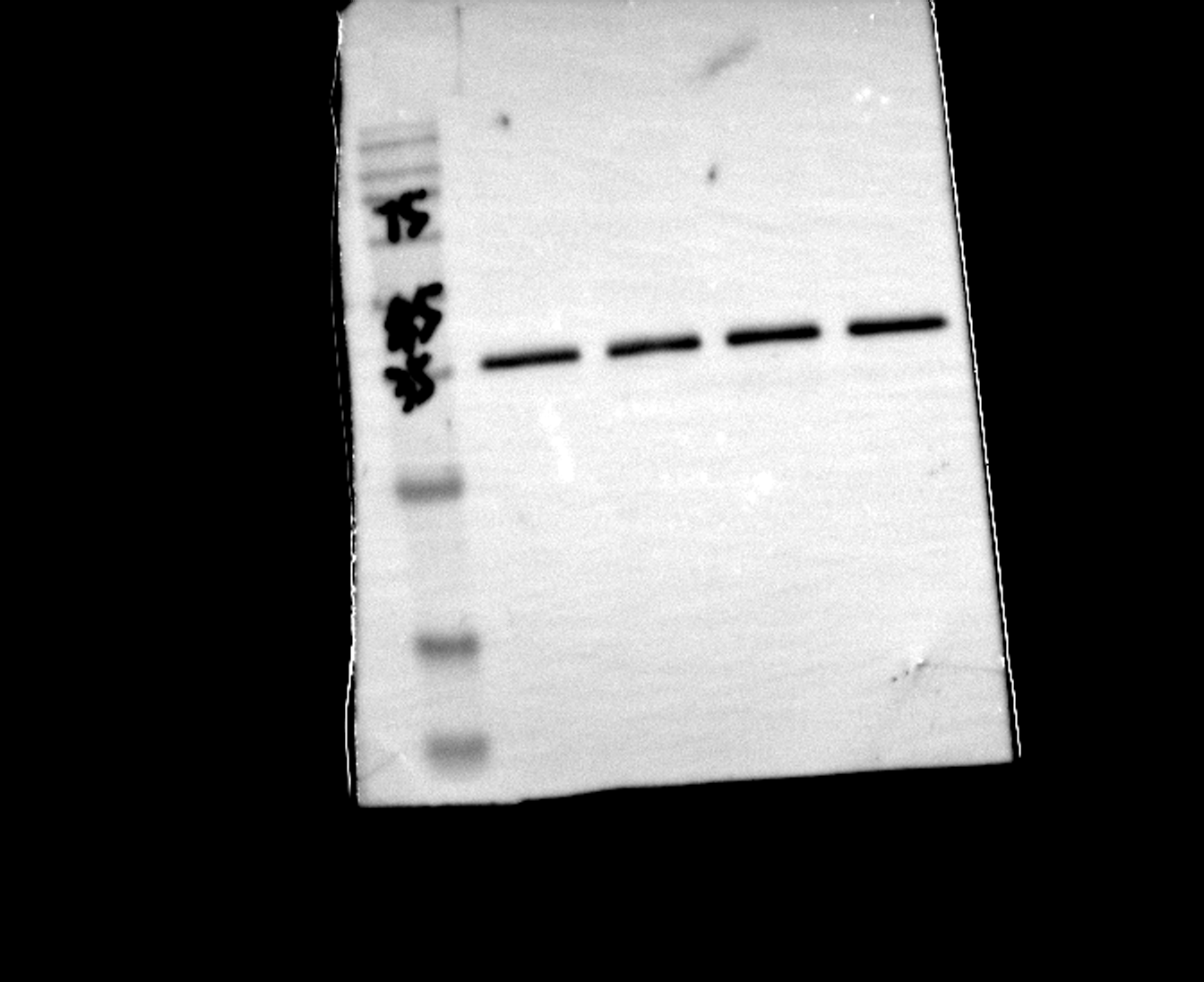


mTOR

**
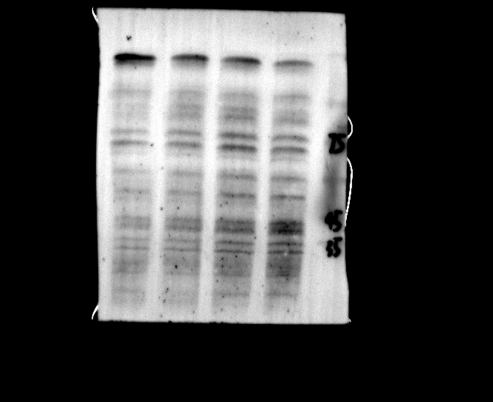
**

P-AKT

**
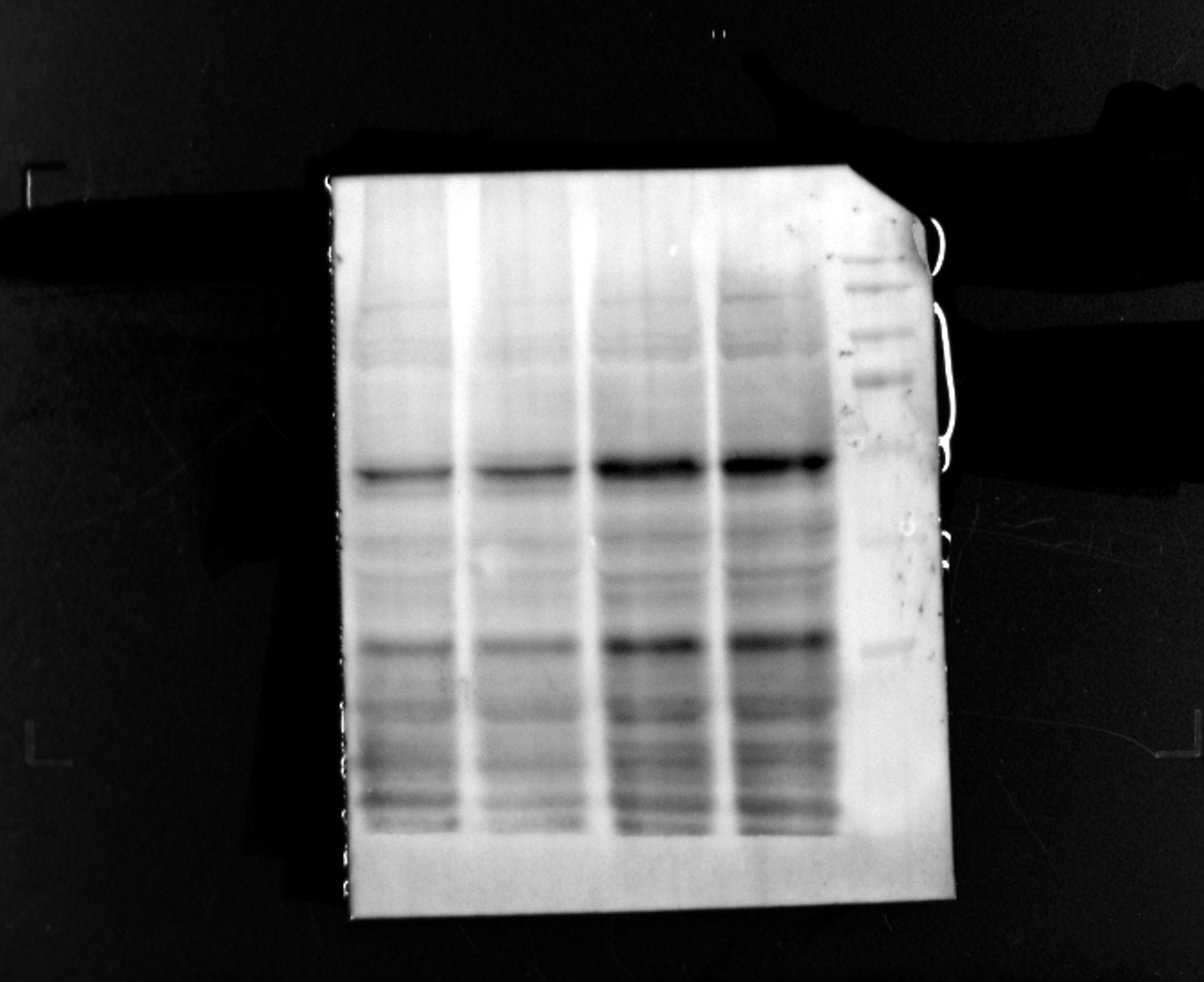
**

p-mTOR(right)

**
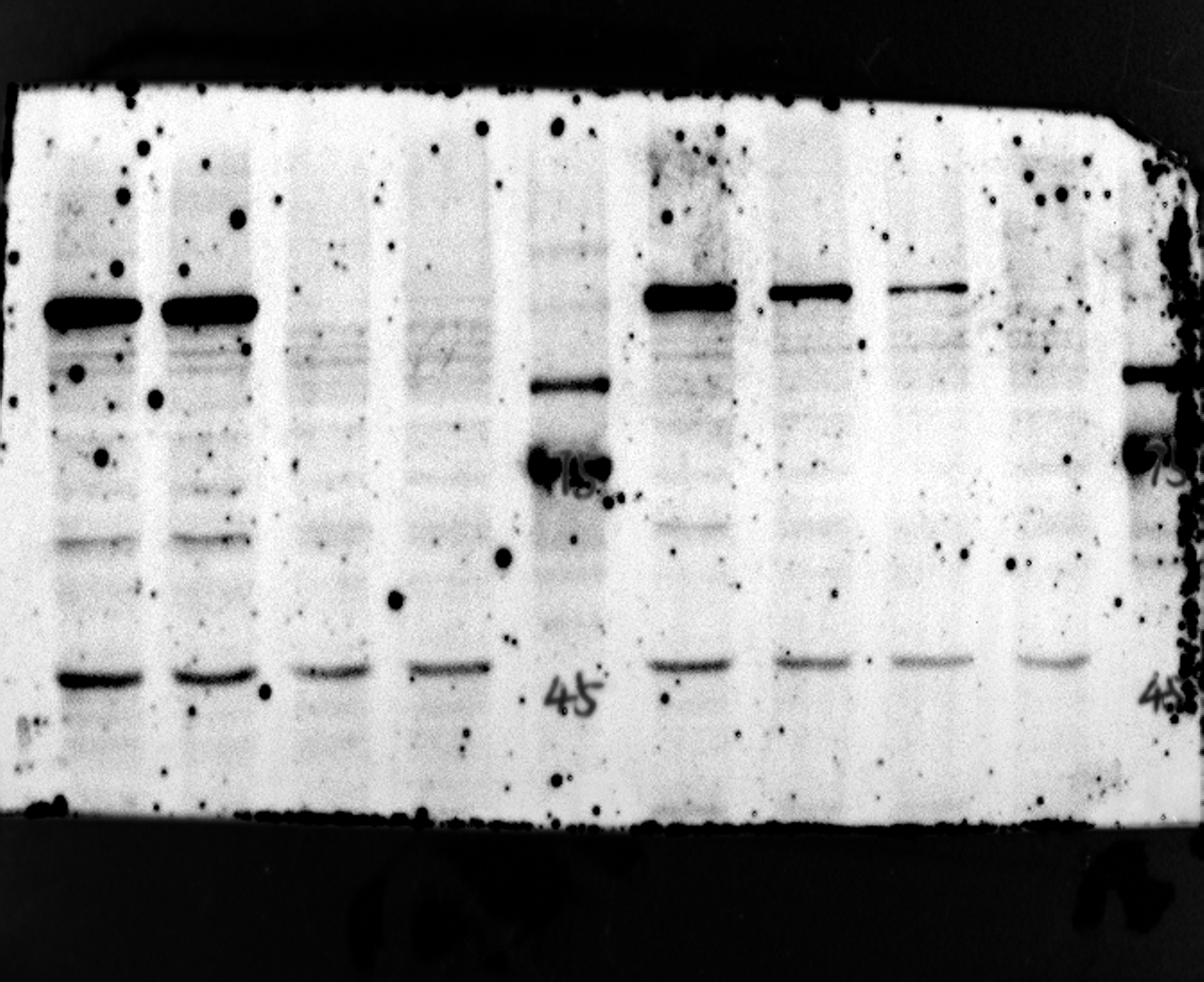
**

**LM3**

GAPDH

**
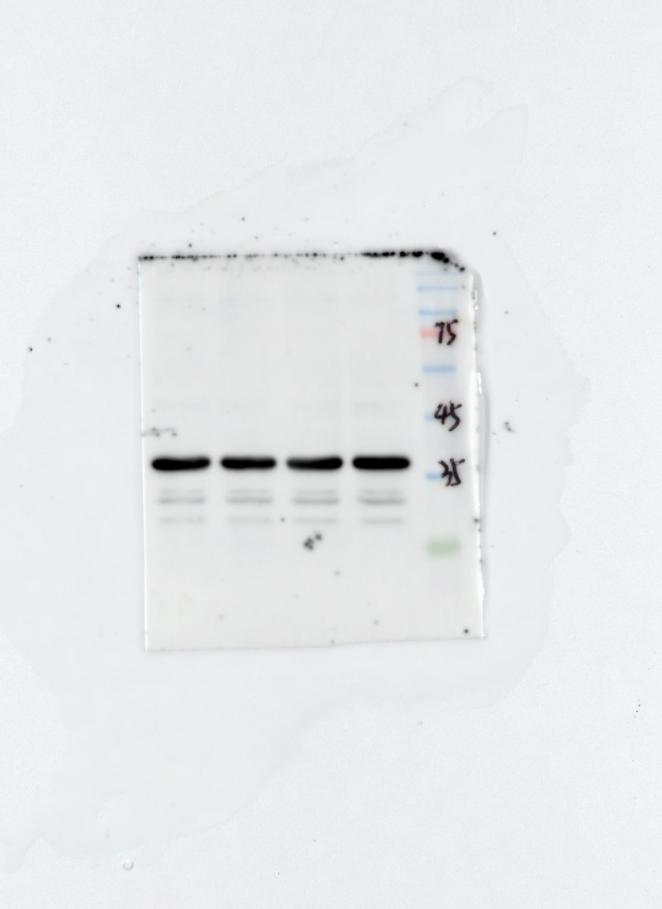
**

AKT

**
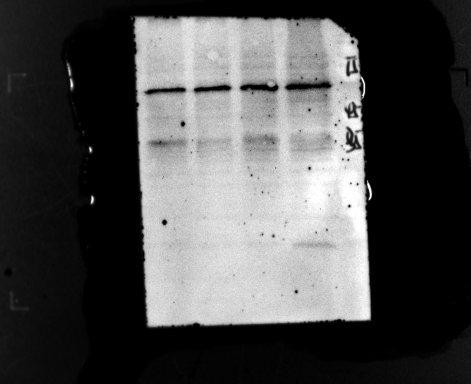
**

mTOR

**
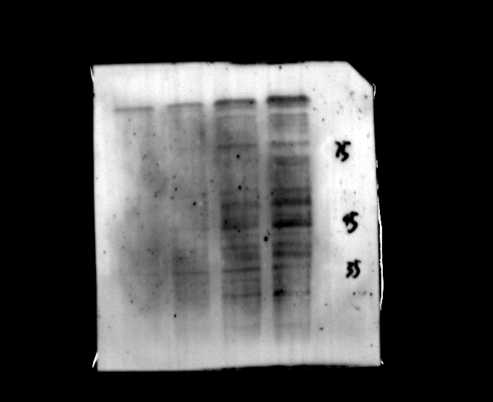
**

P-AKT

**
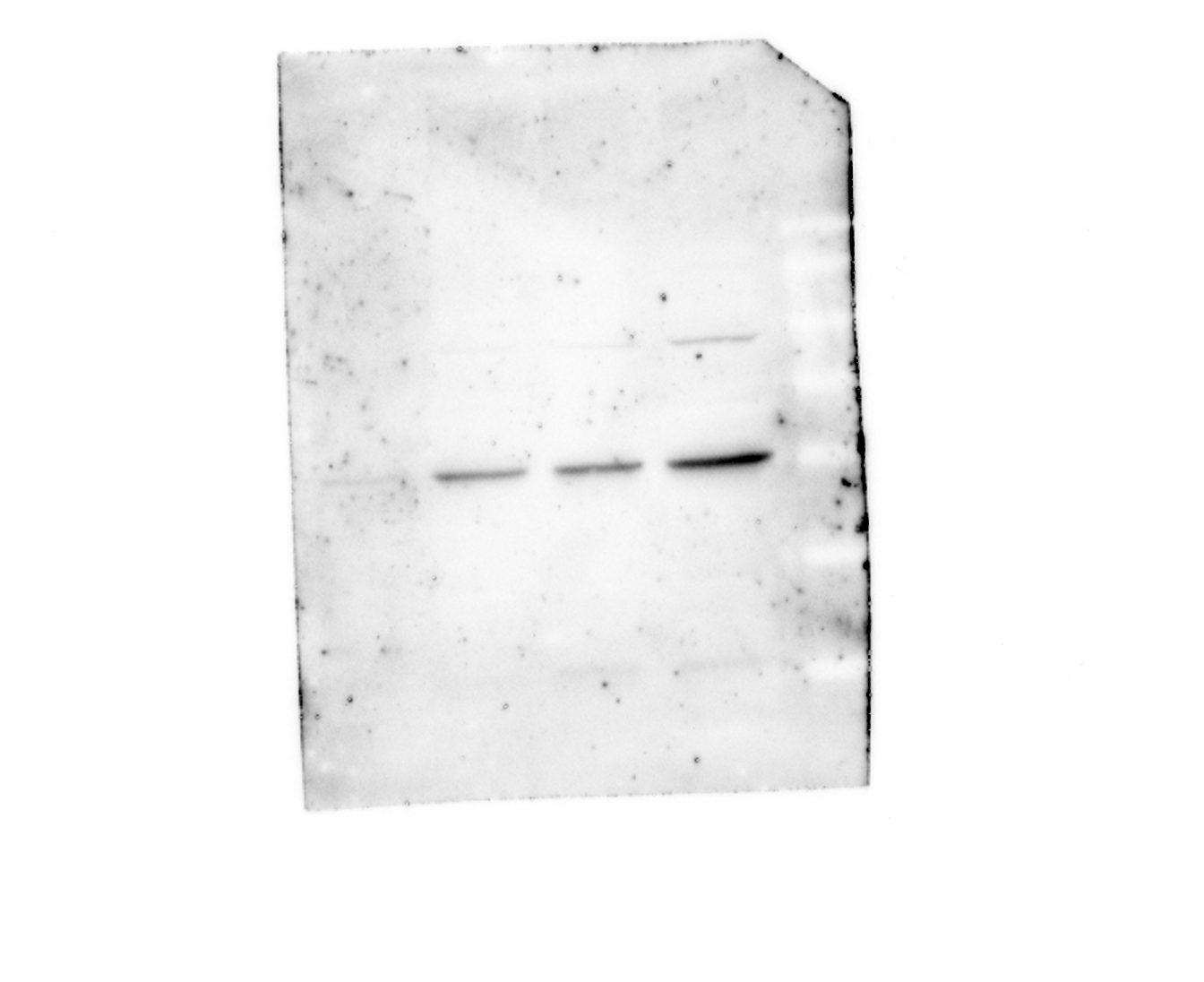
**

P-AKT_marker

**
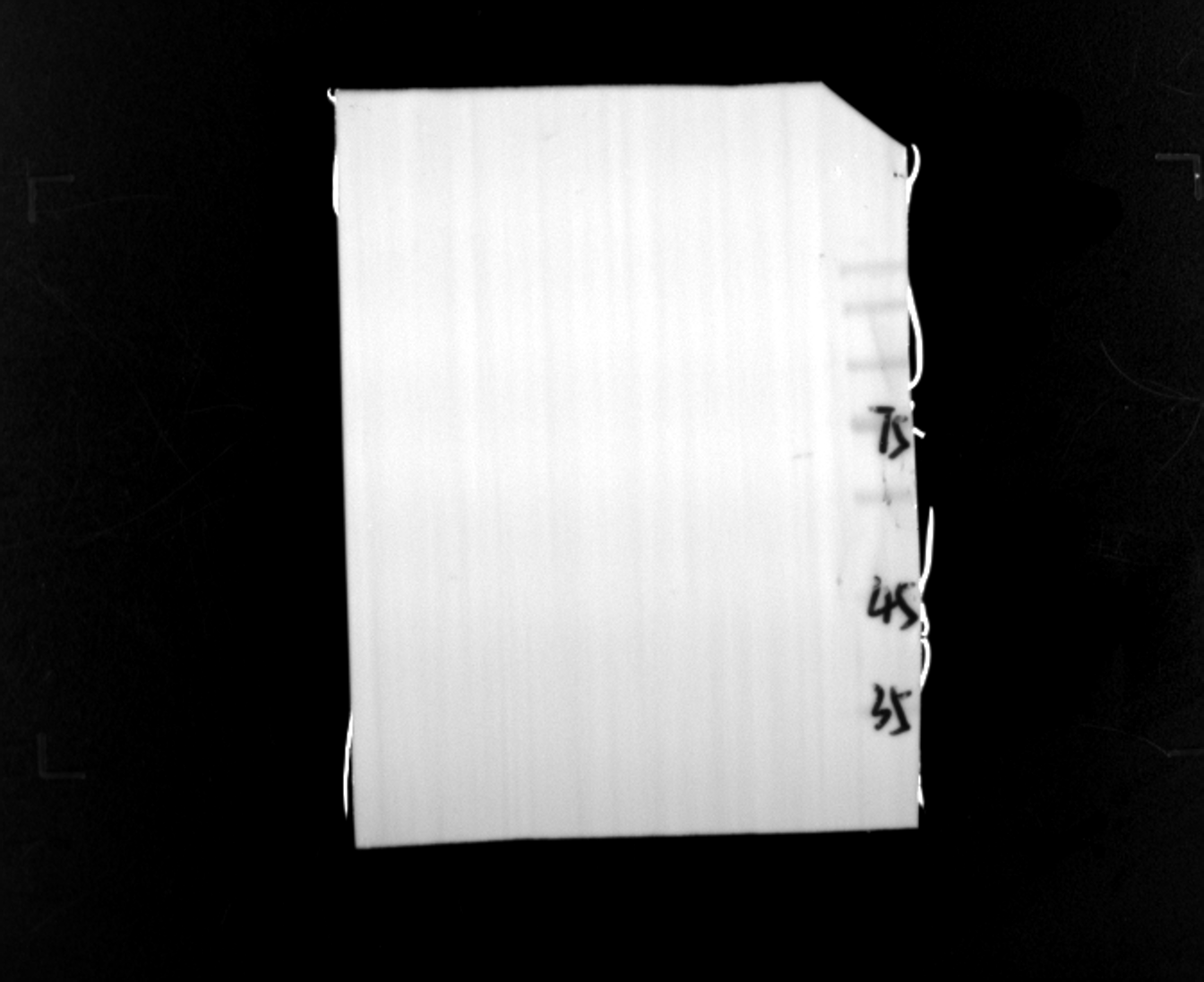
**

P-mTOR

**
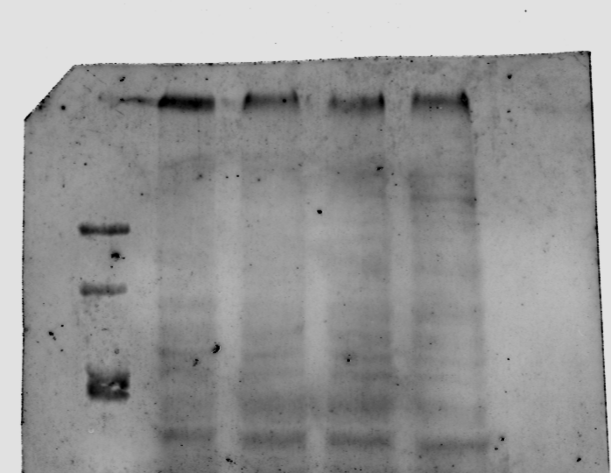
**
