# Supplementary figures and images for: Exploring the anticancer potential of Actinidia chinensis Planch root extracts (acRoots) on hepatocellular carcinoma: A molecular mechanism study (part 2 of 2)
Source: Heliyon. 2023 Nov 2;9(11):e21851. doi: 10.1016/j.heliyon.2023.e21851 (PMC10656260; doi:10.1016/j.heliyon.2023.e21851)

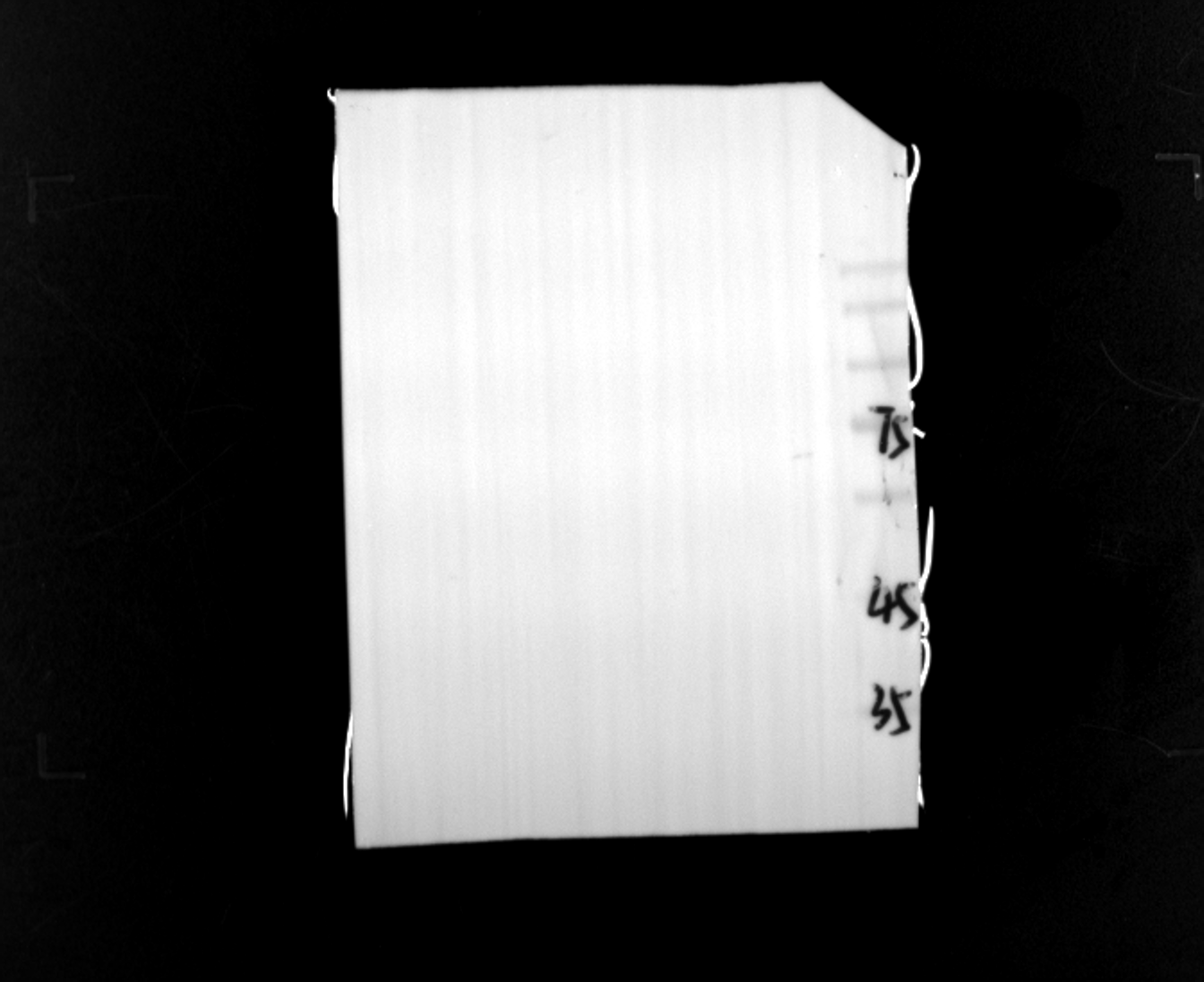

Supplement: Multimedia component 2 [file mmc2.zip › Supplemental_files/Figure 4/Figure 4A/LM3/p-akt_marker.Tif]

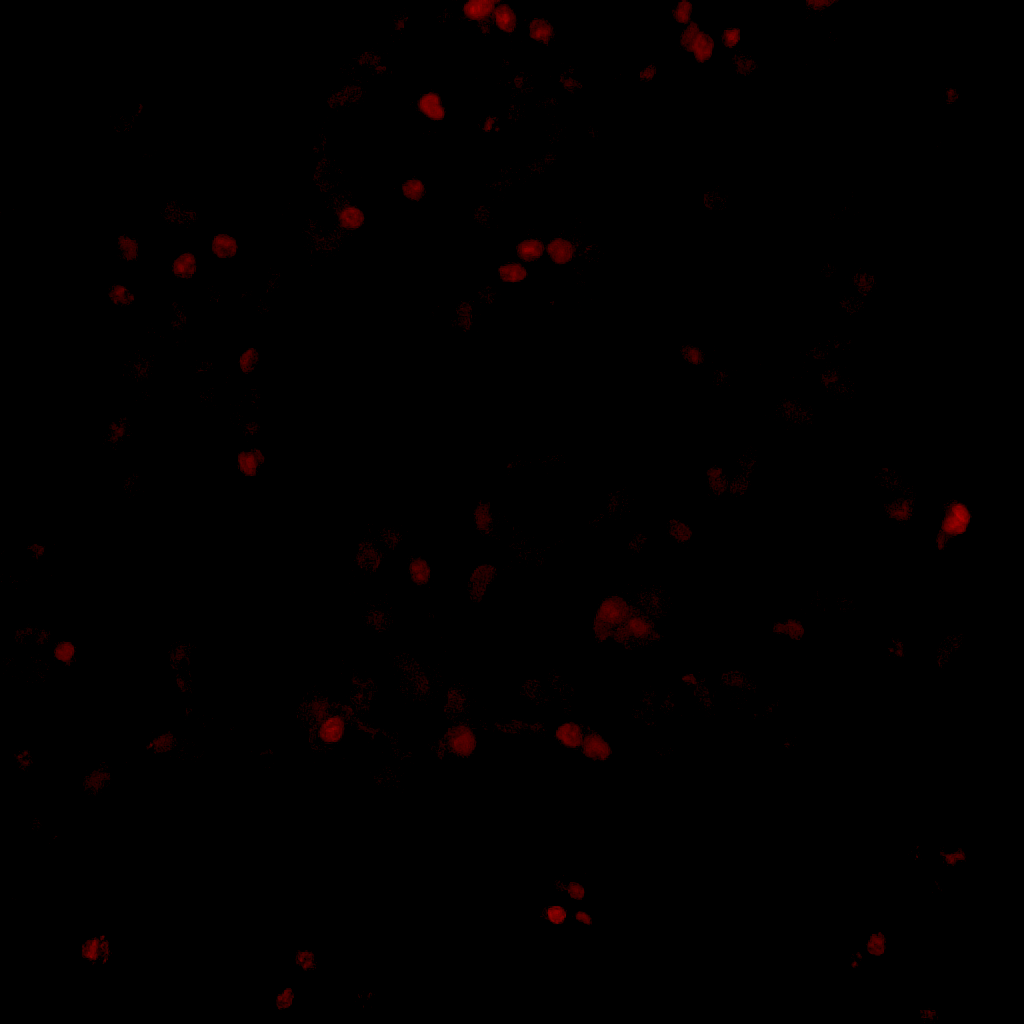

Supplement: Multimedia component 2 [file mmc2.zip › Supplemental_files/Figure 4/Figure 4F/HepG2/acRoots+Reci+_Edu.tif]

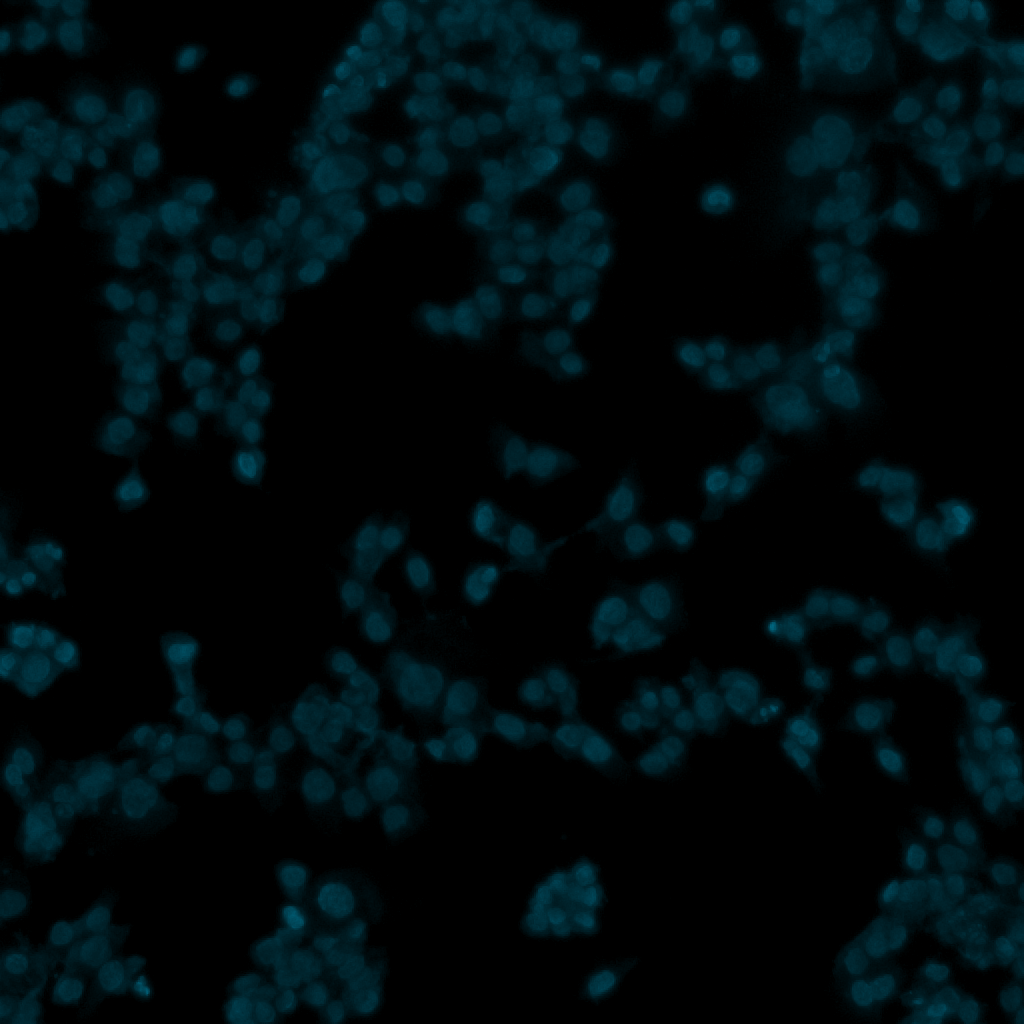

Supplement: Multimedia component 2 [file mmc2.zip › Supplemental_files/Figure 4/Figure 4F/HepG2/acRoots+Reci+_Hon.tif]

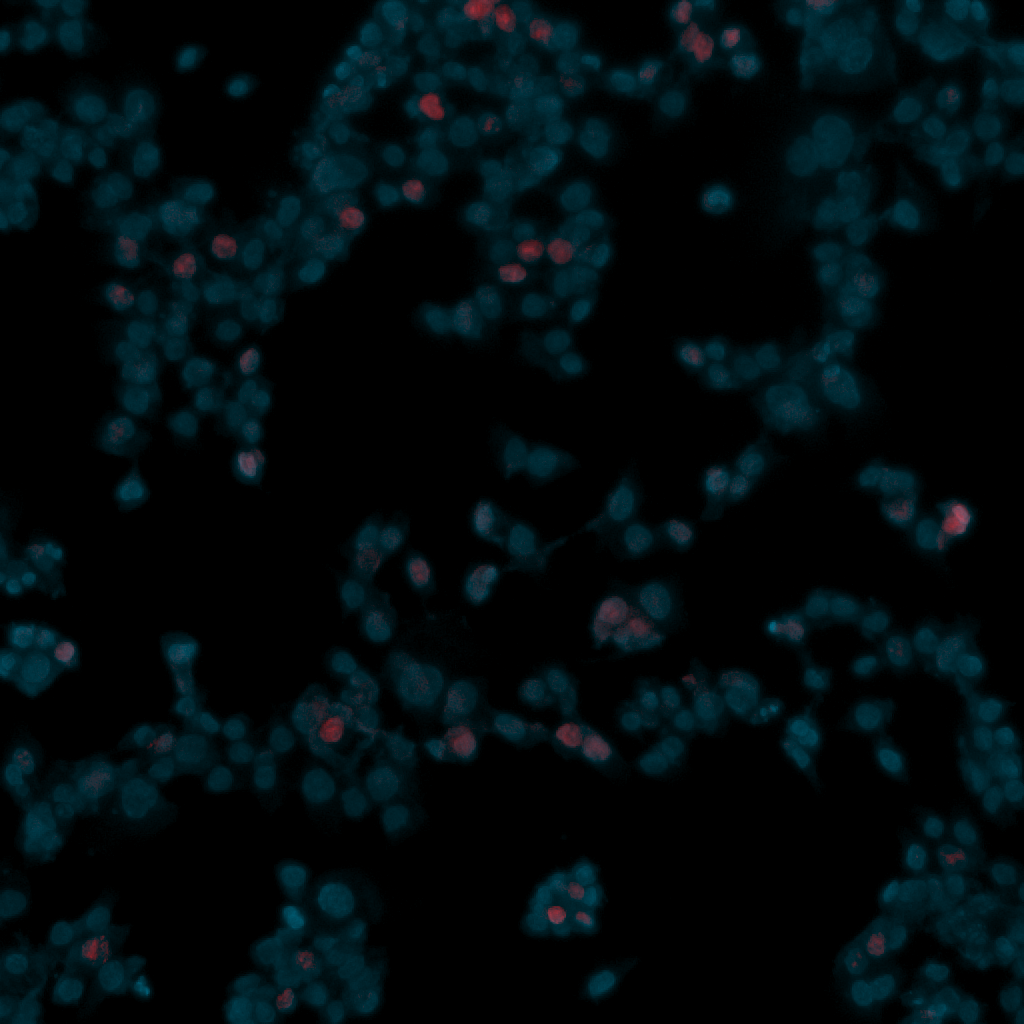

Supplement: Multimedia component 2 [file mmc2.zip › Supplemental_files/Figure 4/Figure 4F/HepG2/acRoots+Reci+_merge.tif]

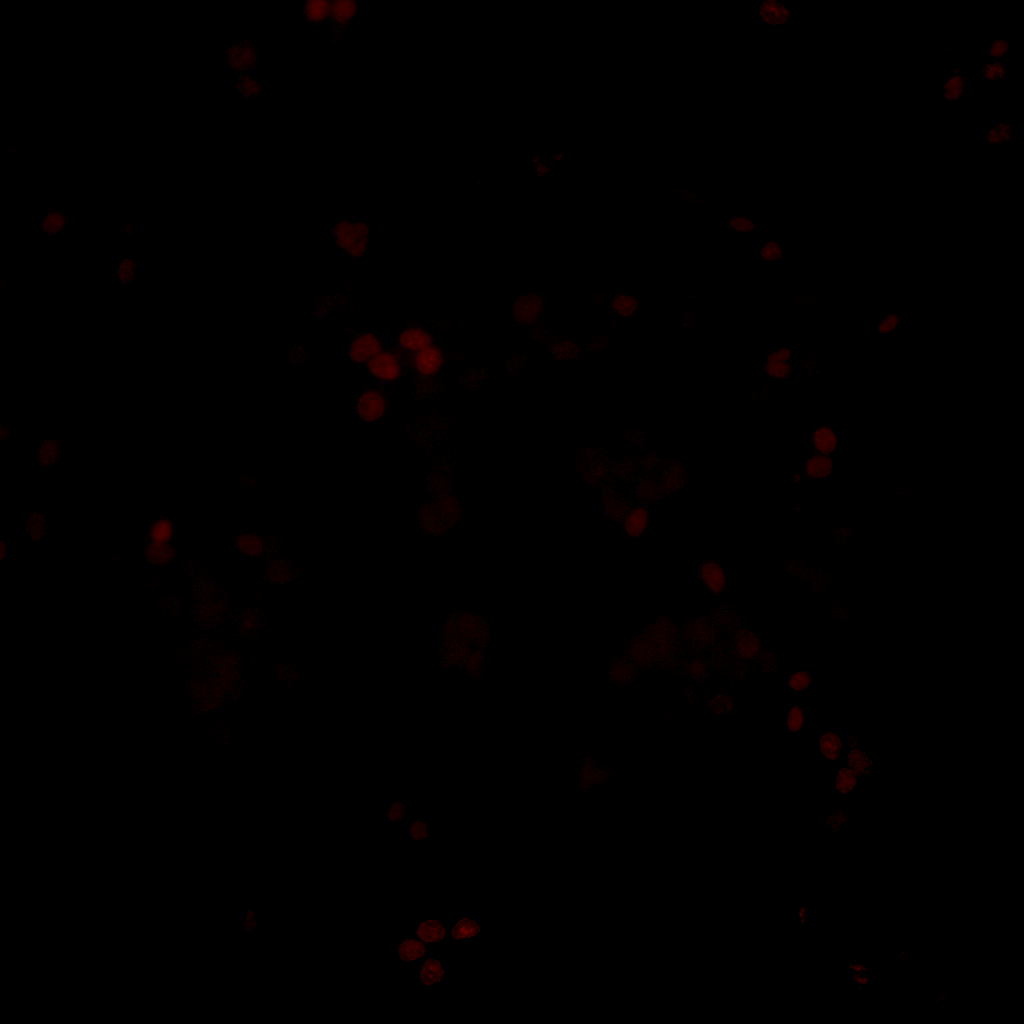

Supplement: Multimedia component 2 [file mmc2.zip › Supplemental_files/Figure 4/Figure 4F/HepG2/acRoots+Reci-_Edu.tif]

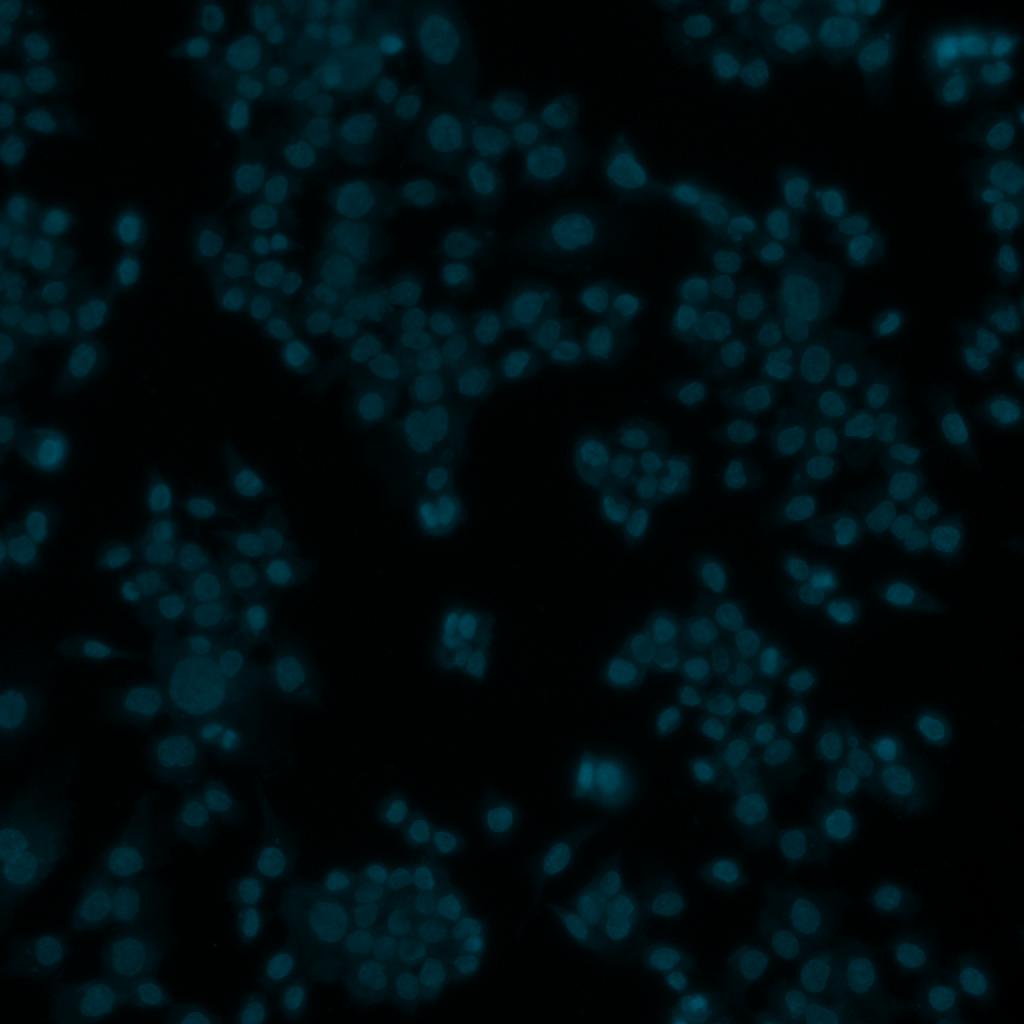

Supplement: Multimedia component 2 [file mmc2.zip › Supplemental_files/Figure 4/Figure 4F/HepG2/acRoots+Reci-_Hon.tif]

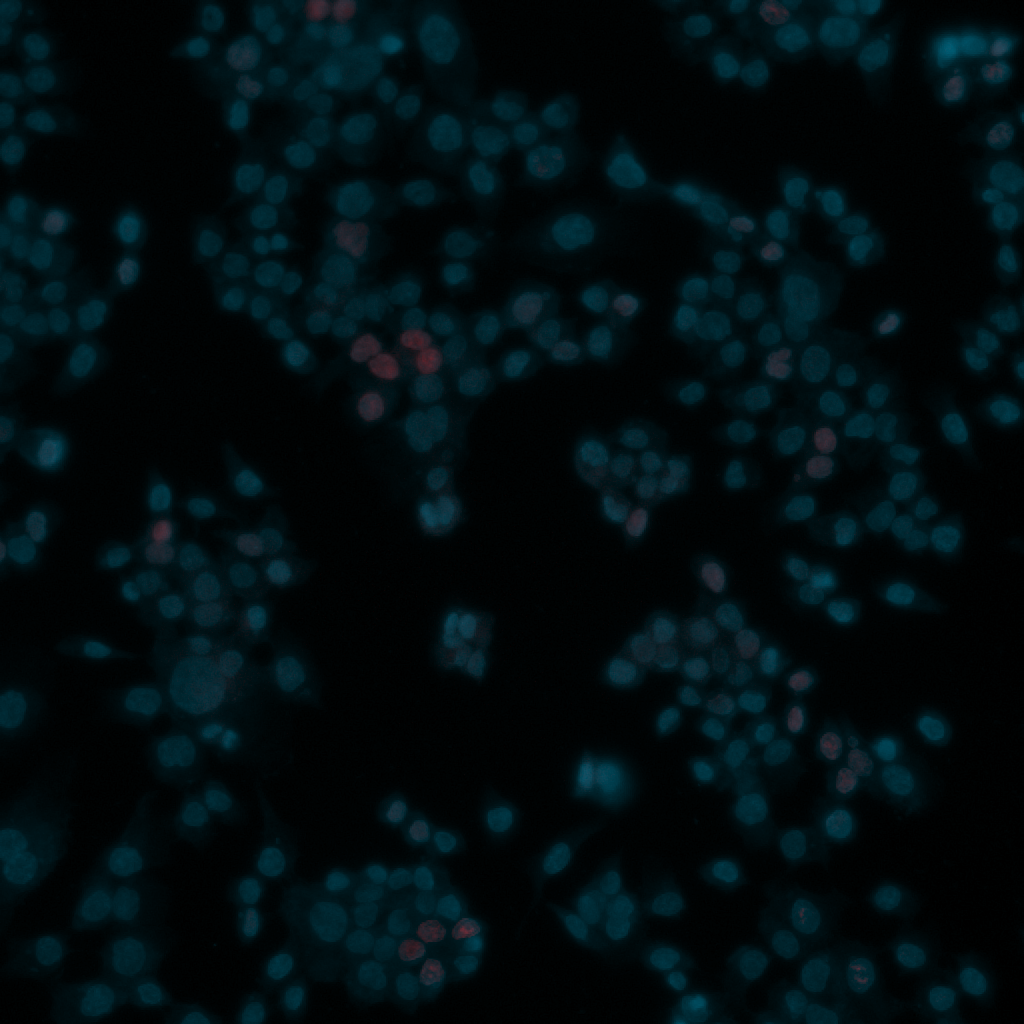

Supplement: Multimedia component 2 [file mmc2.zip › Supplemental_files/Figure 4/Figure 4F/HepG2/acRoots+Reci-_merge.tif]

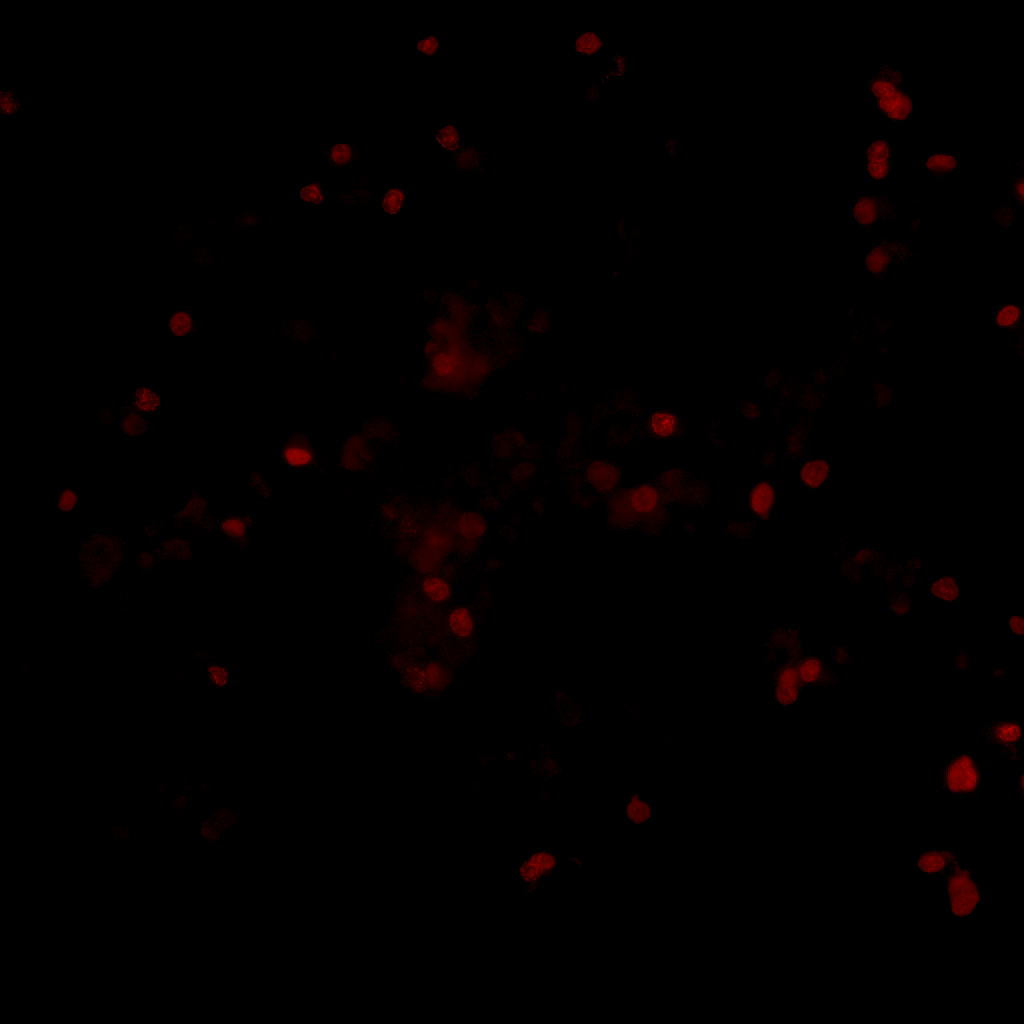

Supplement: Multimedia component 2 [file mmc2.zip › Supplemental_files/Figure 4/Figure 4F/HepG2/acRoots-Reci+_Edu.tif]

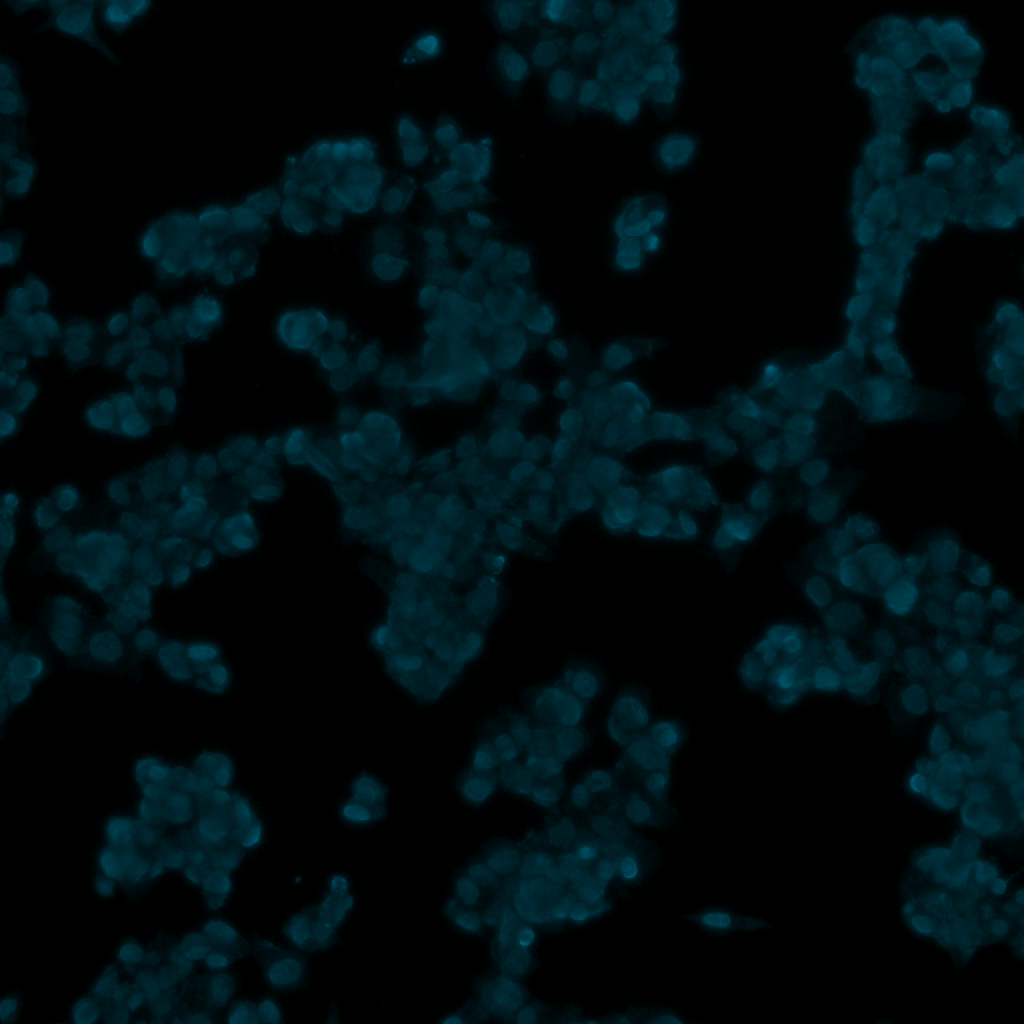

Supplement: Multimedia component 2 [file mmc2.zip › Supplemental_files/Figure 4/Figure 4F/HepG2/acRoots-Reci+_Hon.tif]

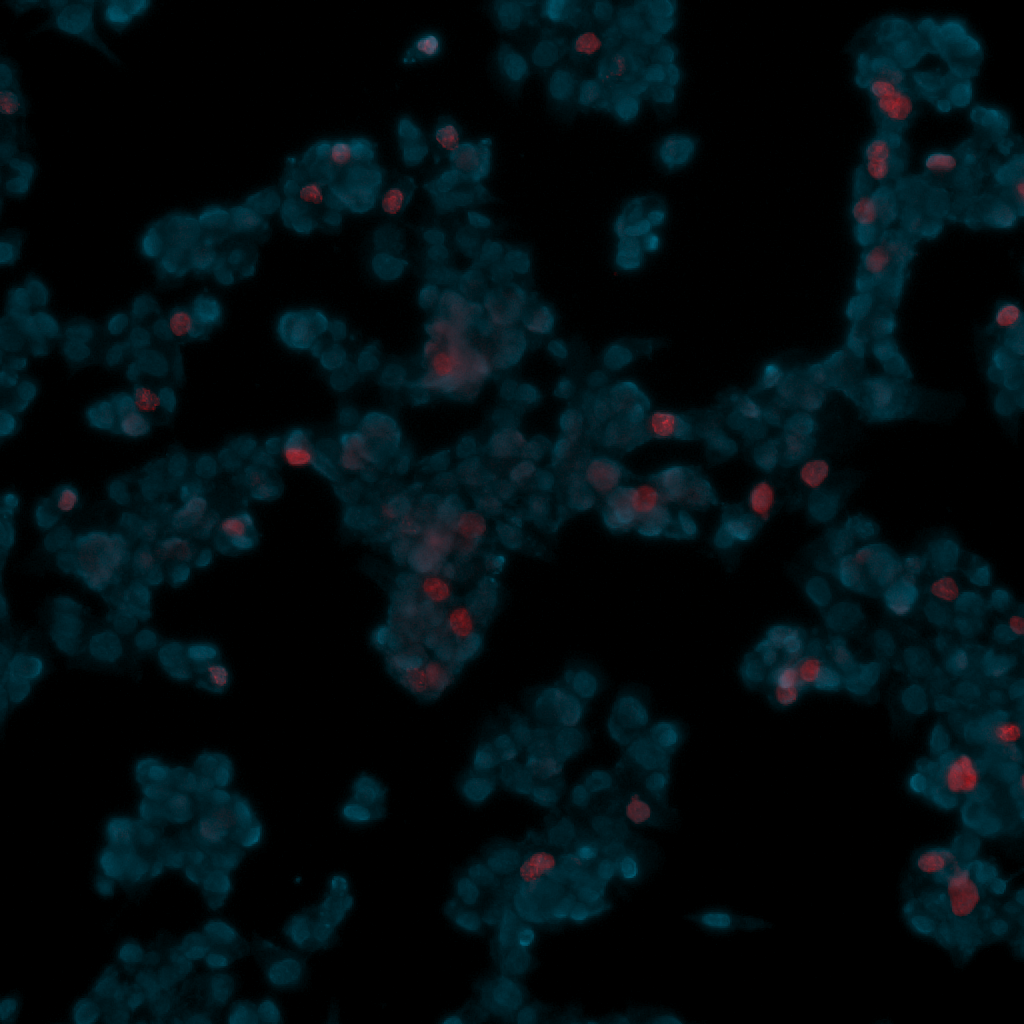

Supplement: Multimedia component 2 [file mmc2.zip › Supplemental_files/Figure 4/Figure 4F/HepG2/acRoots-Reci+_merge.tif]

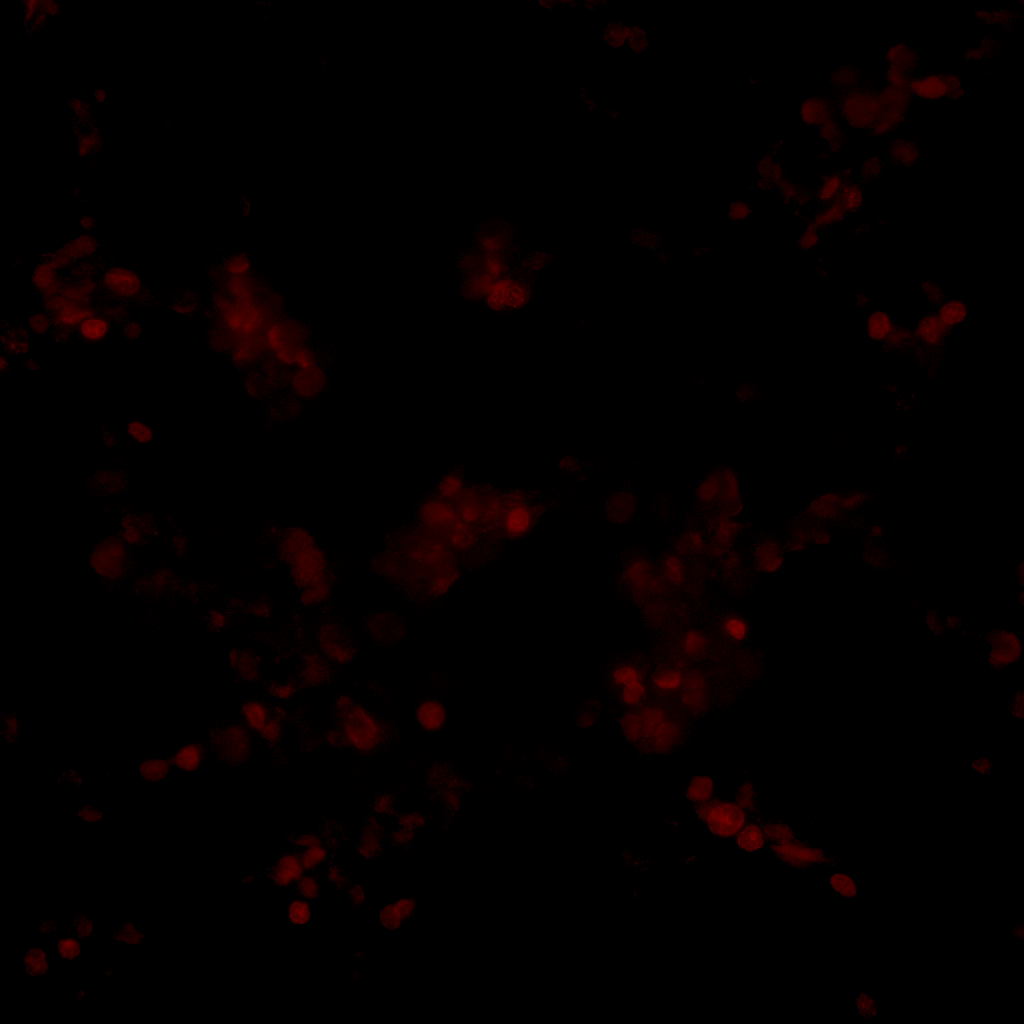

Supplement: Multimedia component 2 [file mmc2.zip › Supplemental_files/Figure 4/Figure 4F/HepG2/acRoots-Reci-_Edu.tif]

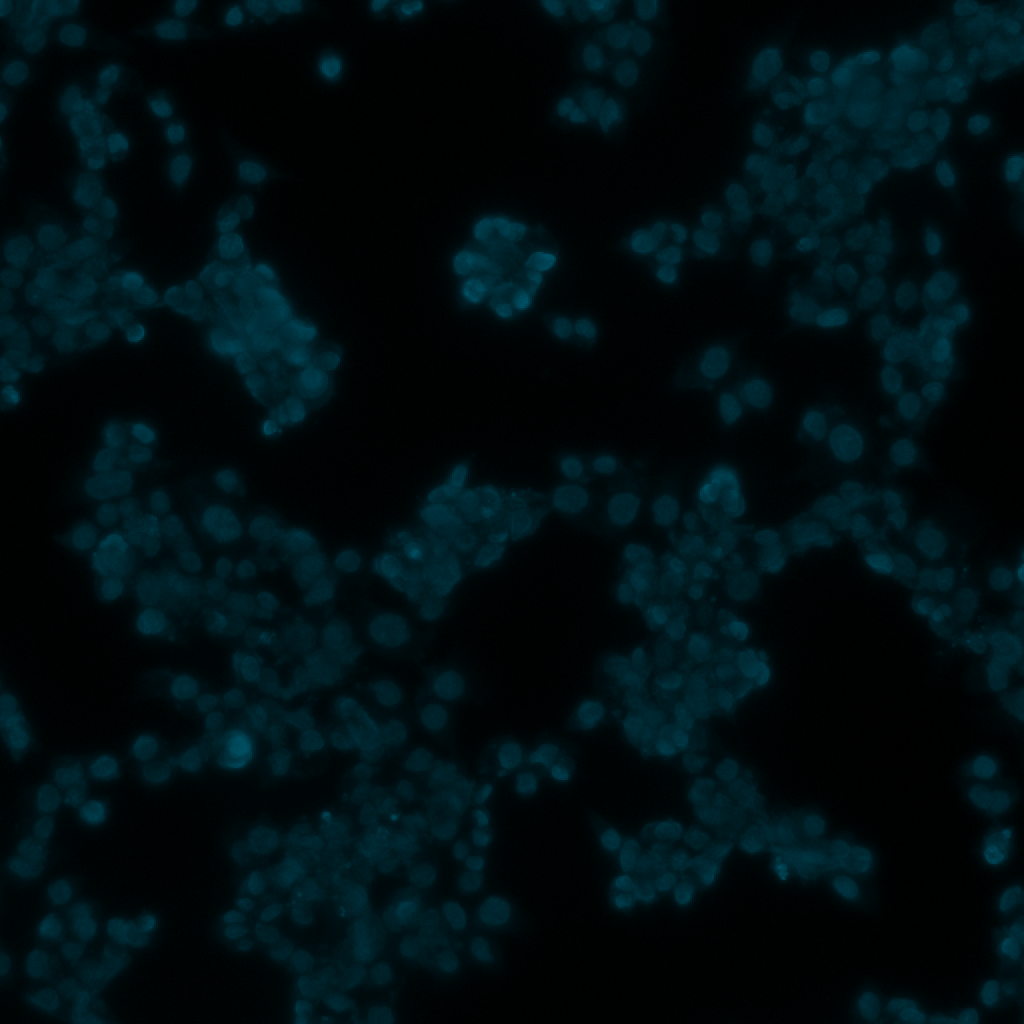

Supplement: Multimedia component 2 [file mmc2.zip › Supplemental_files/Figure 4/Figure 4F/HepG2/acRoots-Reci-_Hon.tif]

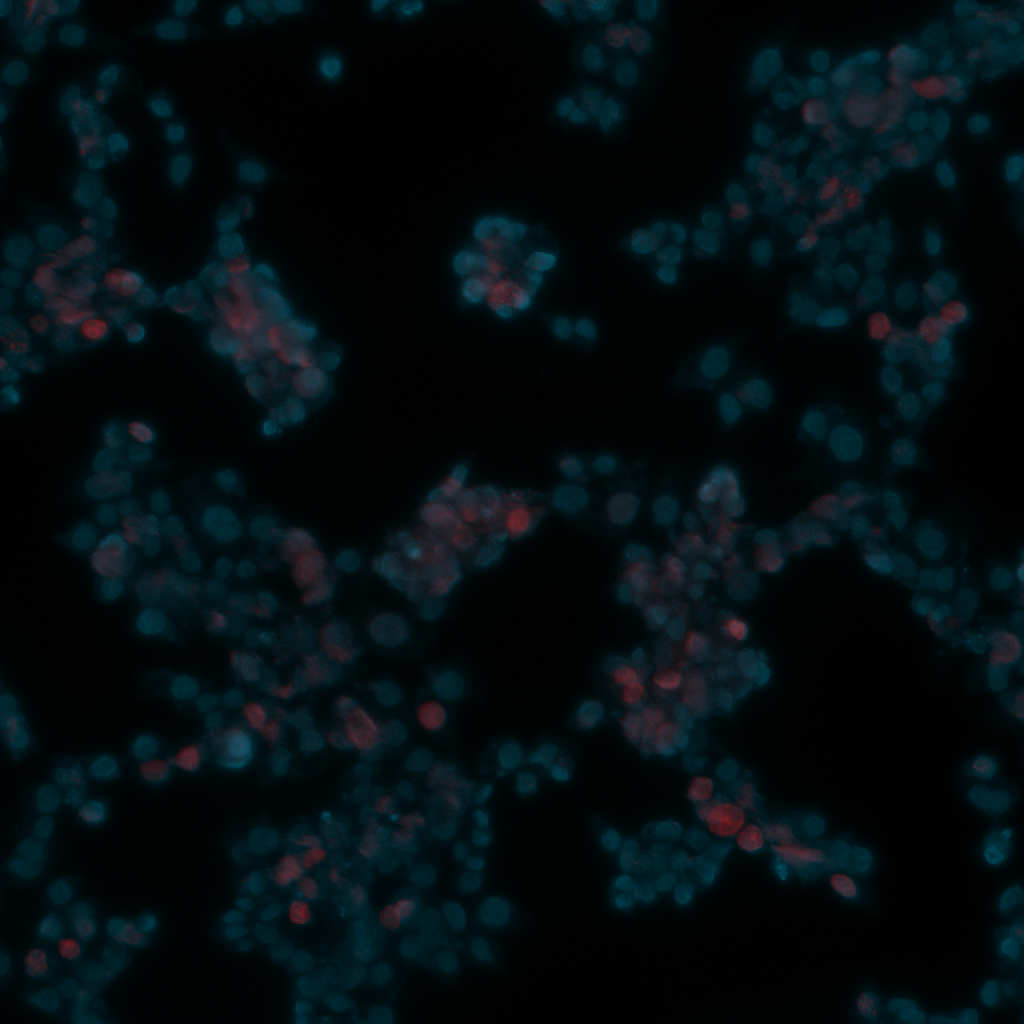

Supplement: Multimedia component 2 [file mmc2.zip › Supplemental_files/Figure 4/Figure 4F/HepG2/acRoots-Reci-_merge.tif]

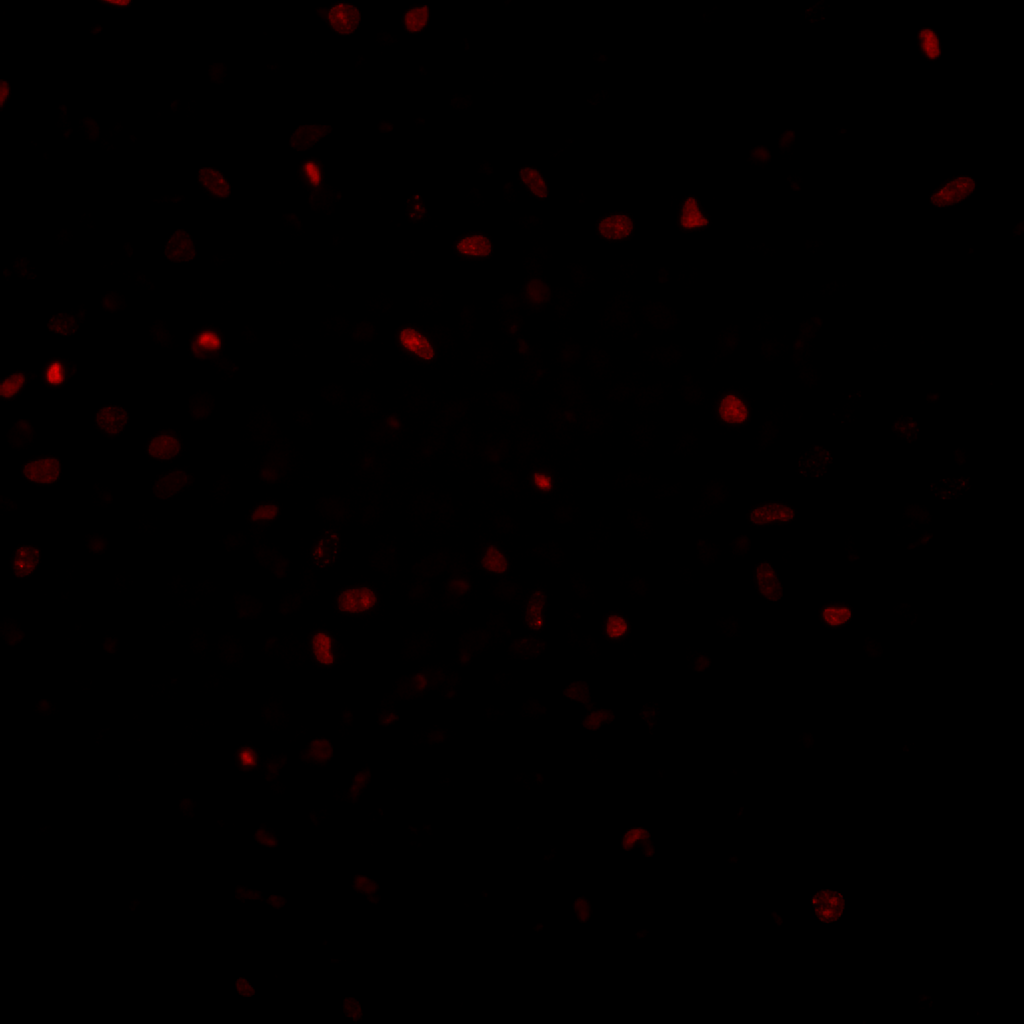

Supplement: Multimedia component 2 [file mmc2.zip › Supplemental_files/Figure 4/Figure 4F/LM3/acRoots+Reci+_Edu.tif]

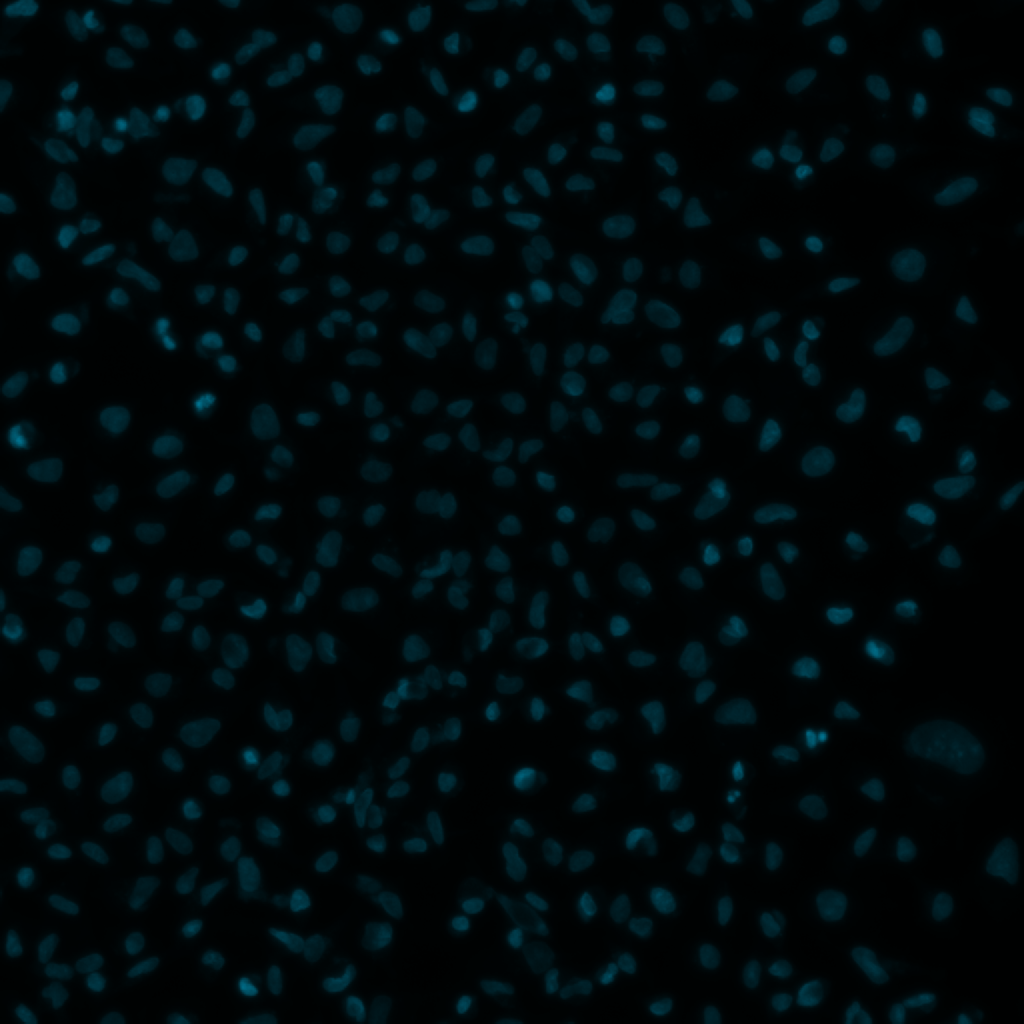

Supplement: Multimedia component 2 [file mmc2.zip › Supplemental_files/Figure 4/Figure 4F/LM3/acRoots+Reci+_Hon.tif]

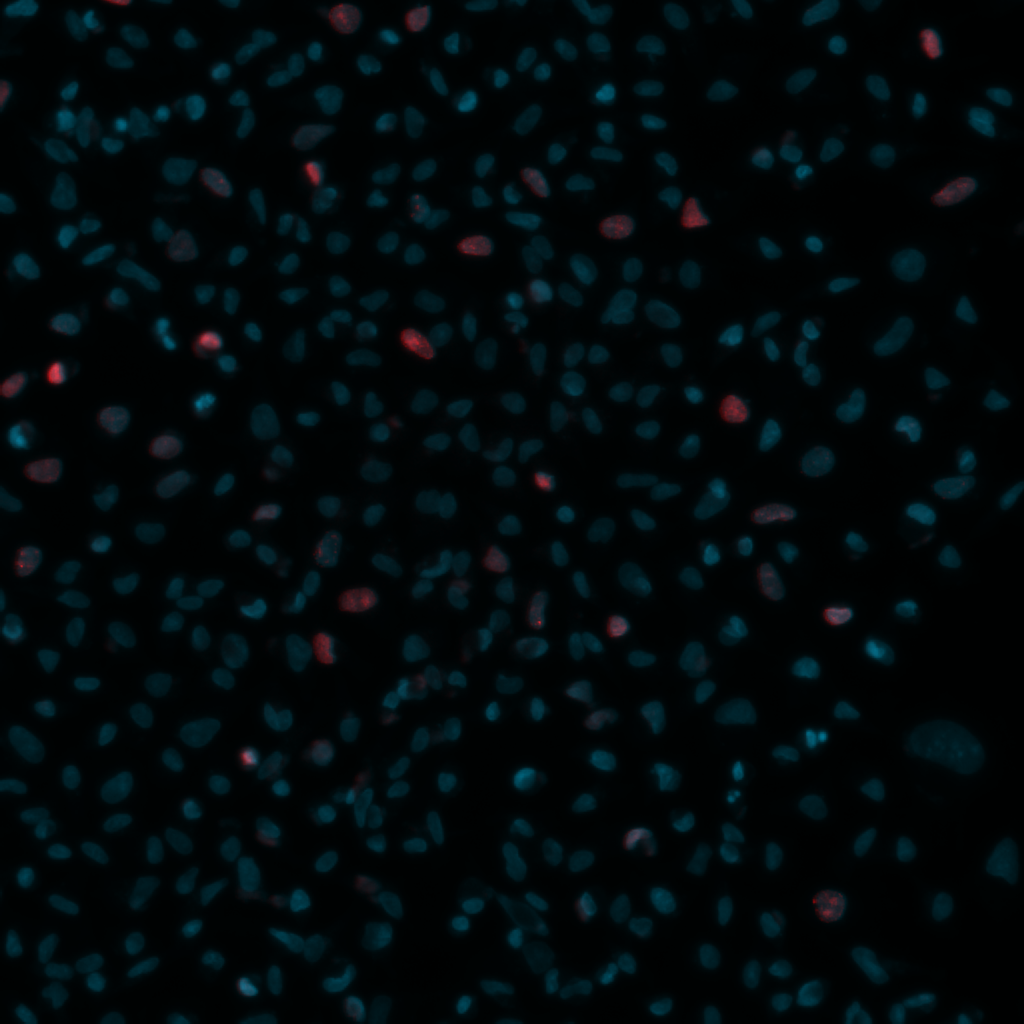

Supplement: Multimedia component 2 [file mmc2.zip › Supplemental_files/Figure 4/Figure 4F/LM3/acRoots+Reci+_merge.tif]

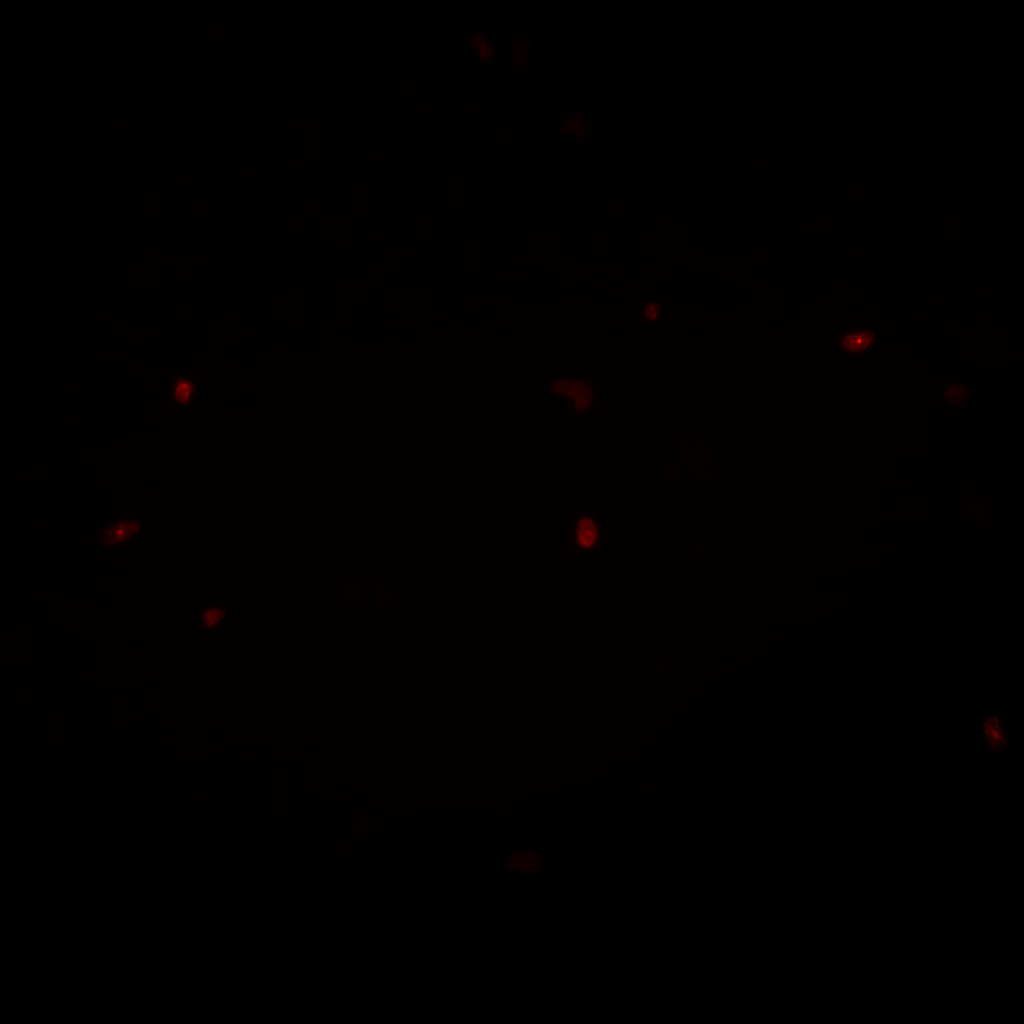

Supplement: Multimedia component 2 [file mmc2.zip › Supplemental_files/Figure 4/Figure 4F/LM3/acRoots+Reci-_Edu.tif]

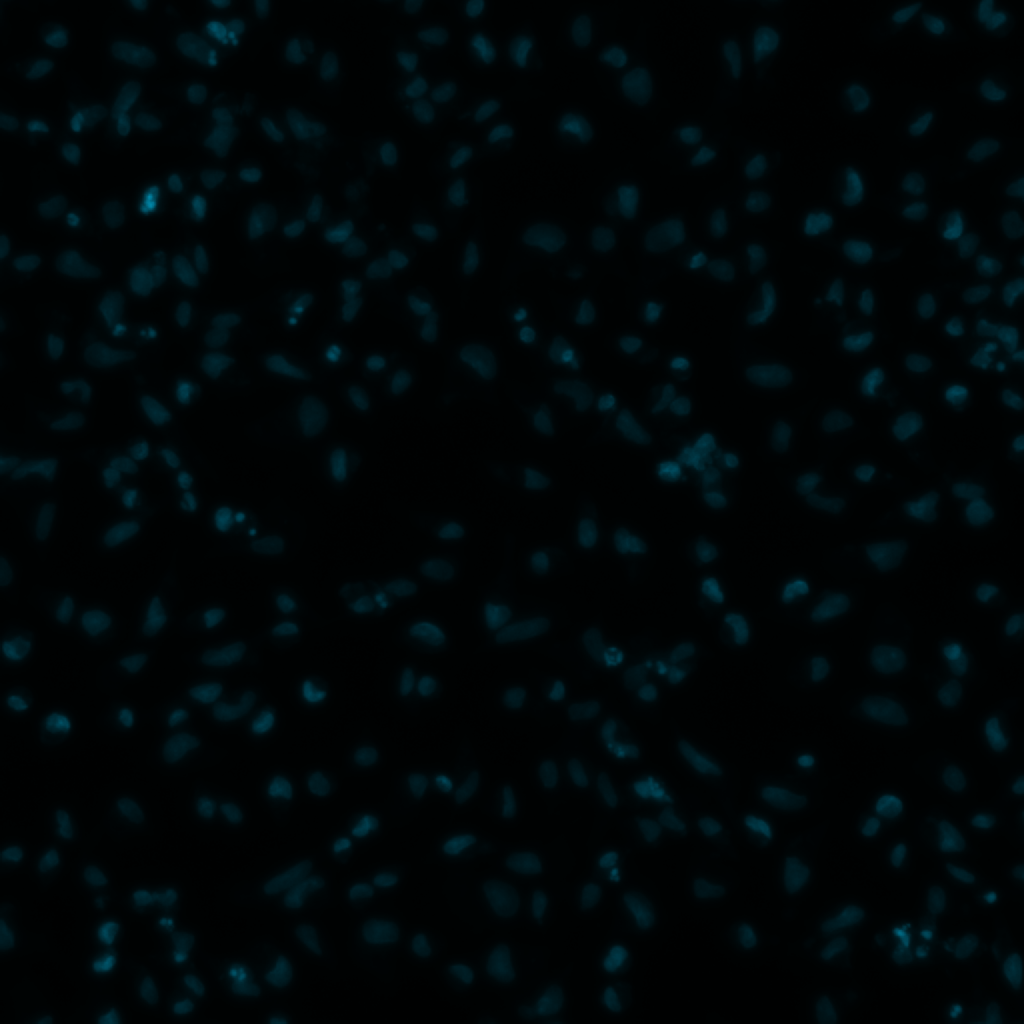

Supplement: Multimedia component 2 [file mmc2.zip › Supplemental_files/Figure 4/Figure 4F/LM3/acRoots+Reci-_Hon.tif]

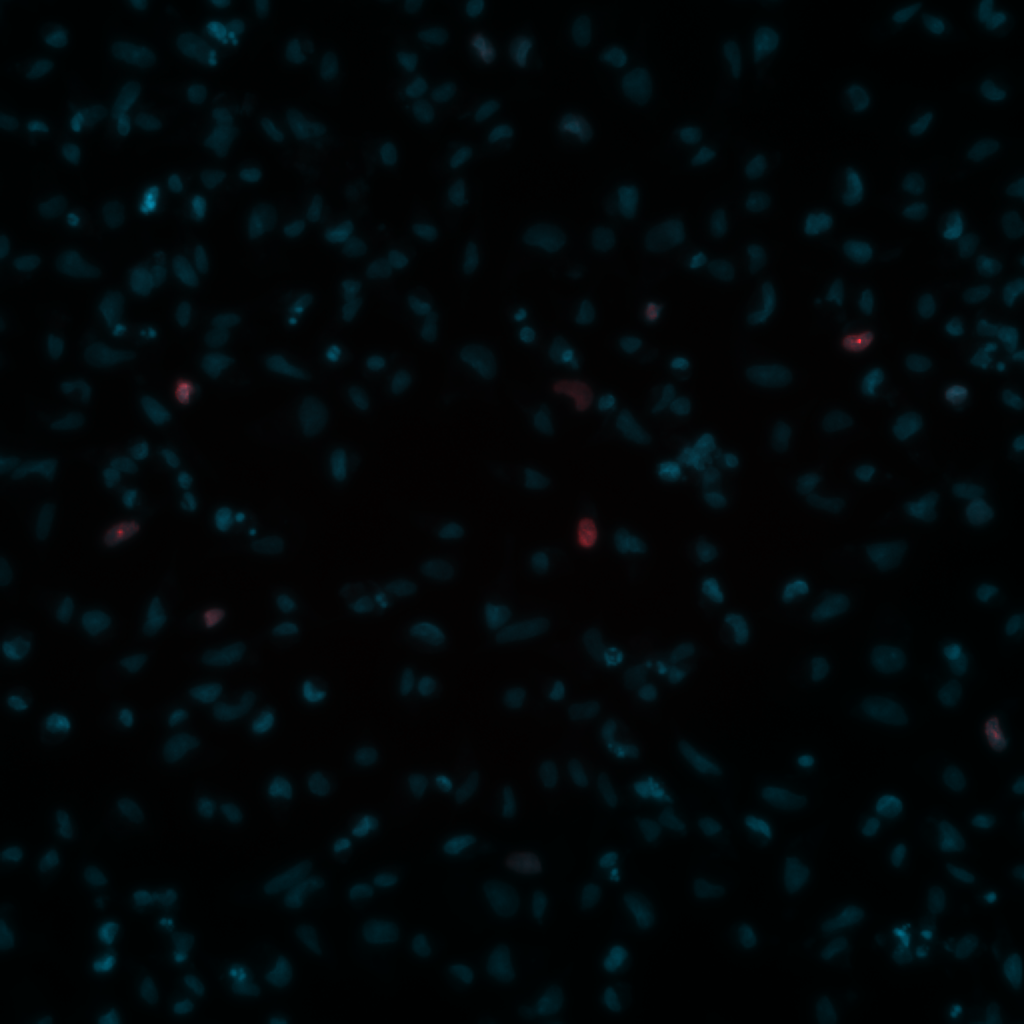

Supplement: Multimedia component 2 [file mmc2.zip › Supplemental_files/Figure 4/Figure 4F/LM3/acRoots+Reci-_merge.tif]

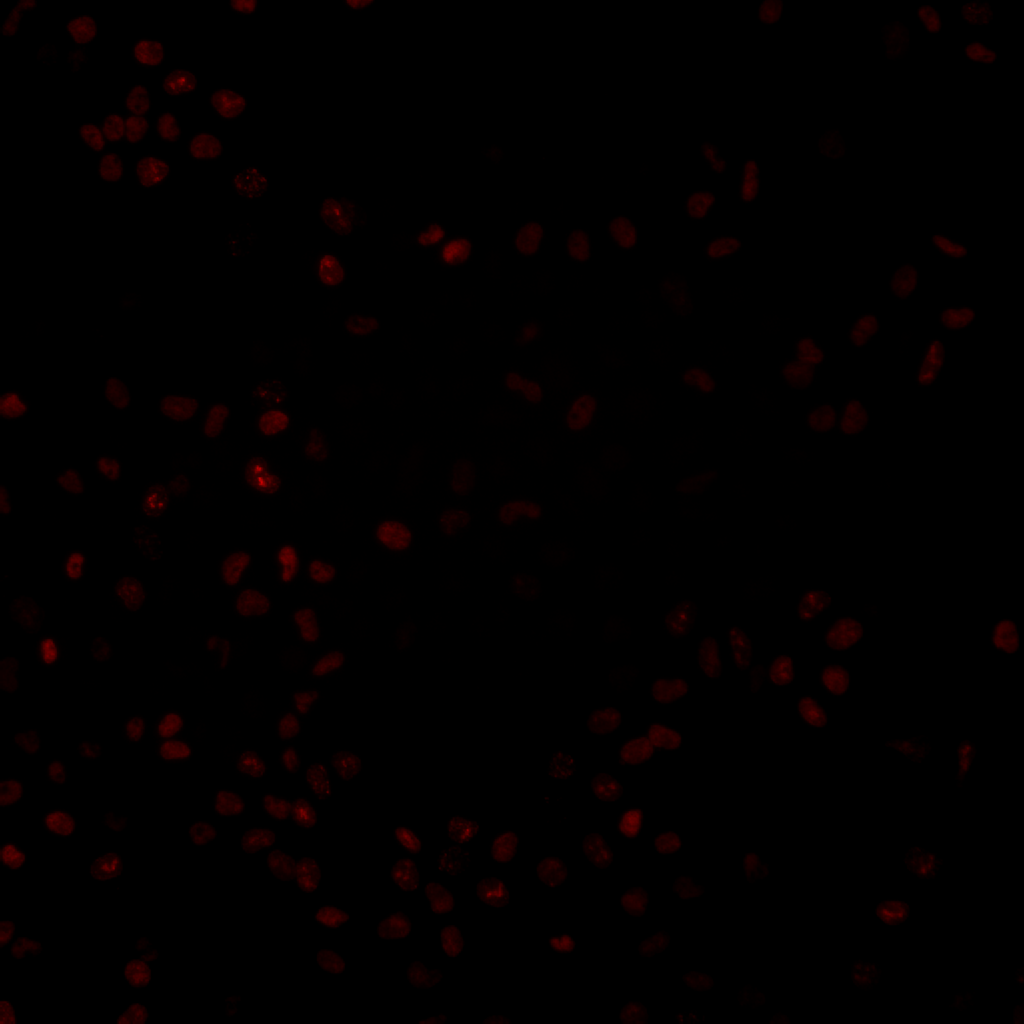

Supplement: Multimedia component 2 [file mmc2.zip › Supplemental_files/Figure 4/Figure 4F/LM3/acRoots-Reci+_Edu.tif]

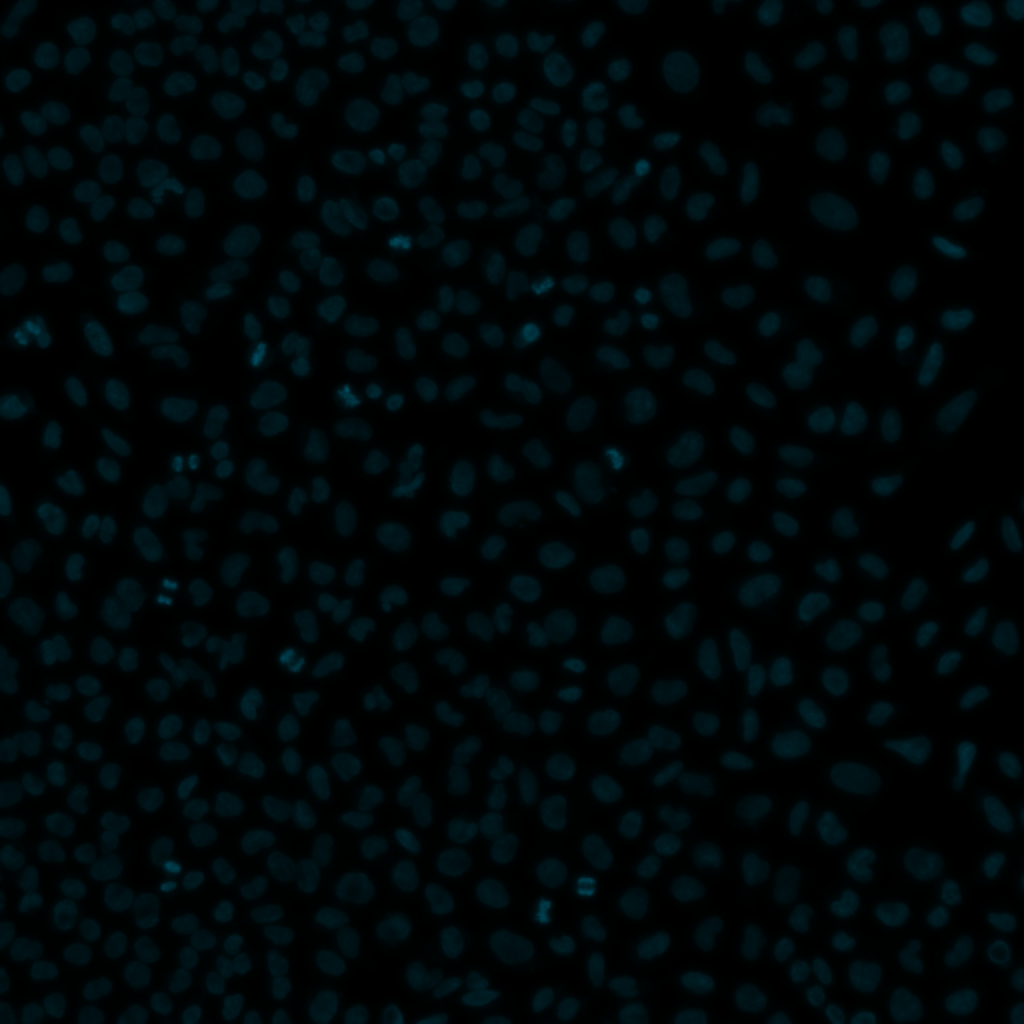

Supplement: Multimedia component 2 [file mmc2.zip › Supplemental_files/Figure 4/Figure 4F/LM3/acRoots-Reci+_Hon.tif]

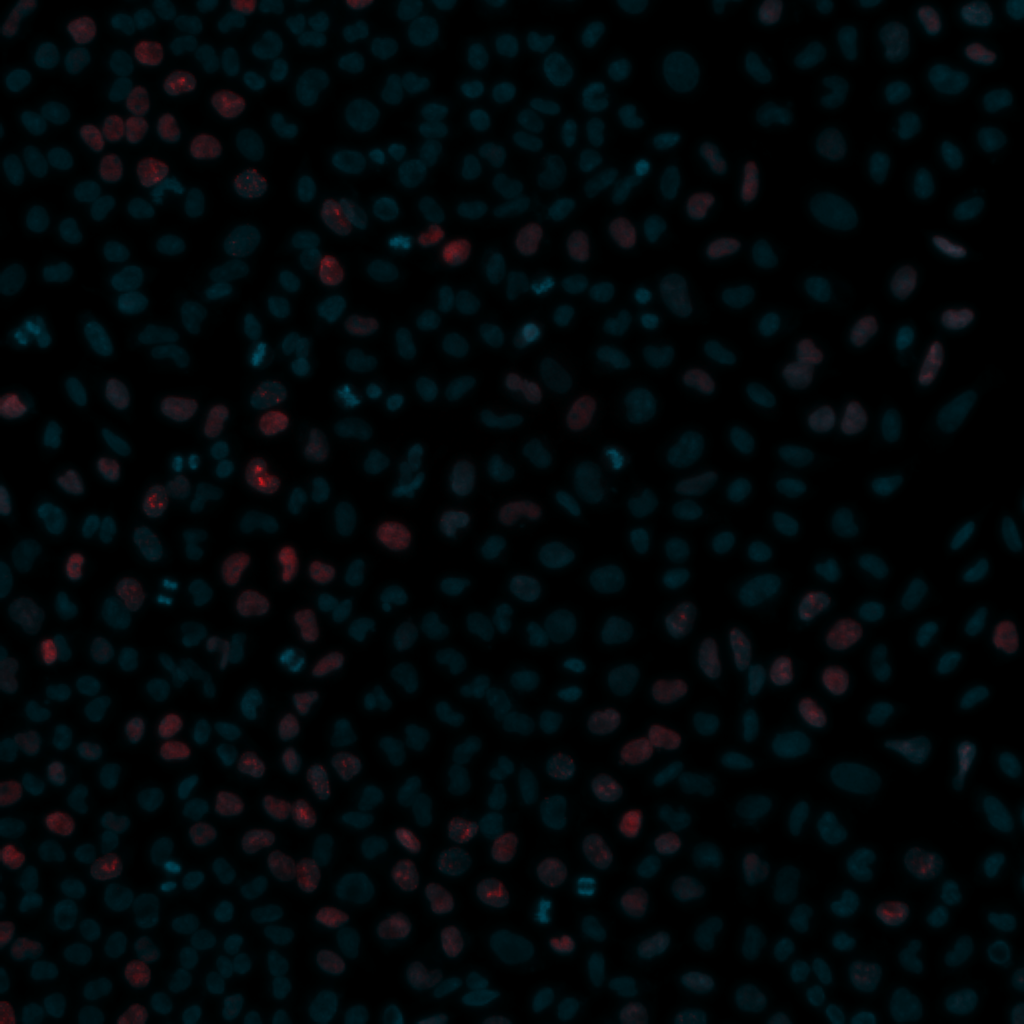

Supplement: Multimedia component 2 [file mmc2.zip › Supplemental_files/Figure 4/Figure 4F/LM3/acRoots-Reci+_merge.tif]

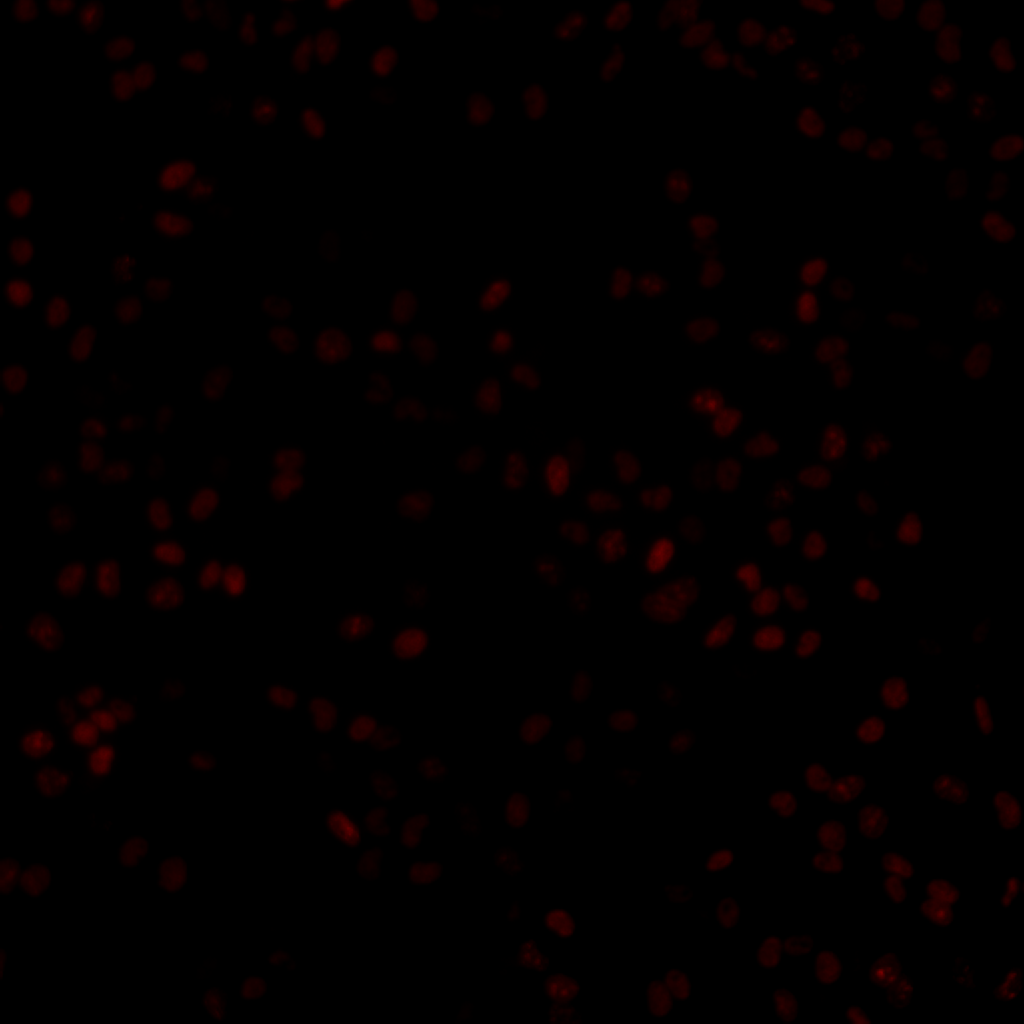

Supplement: Multimedia component 2 [file mmc2.zip › Supplemental_files/Figure 4/Figure 4F/LM3/acRoots-Reci-_Edu.tif]

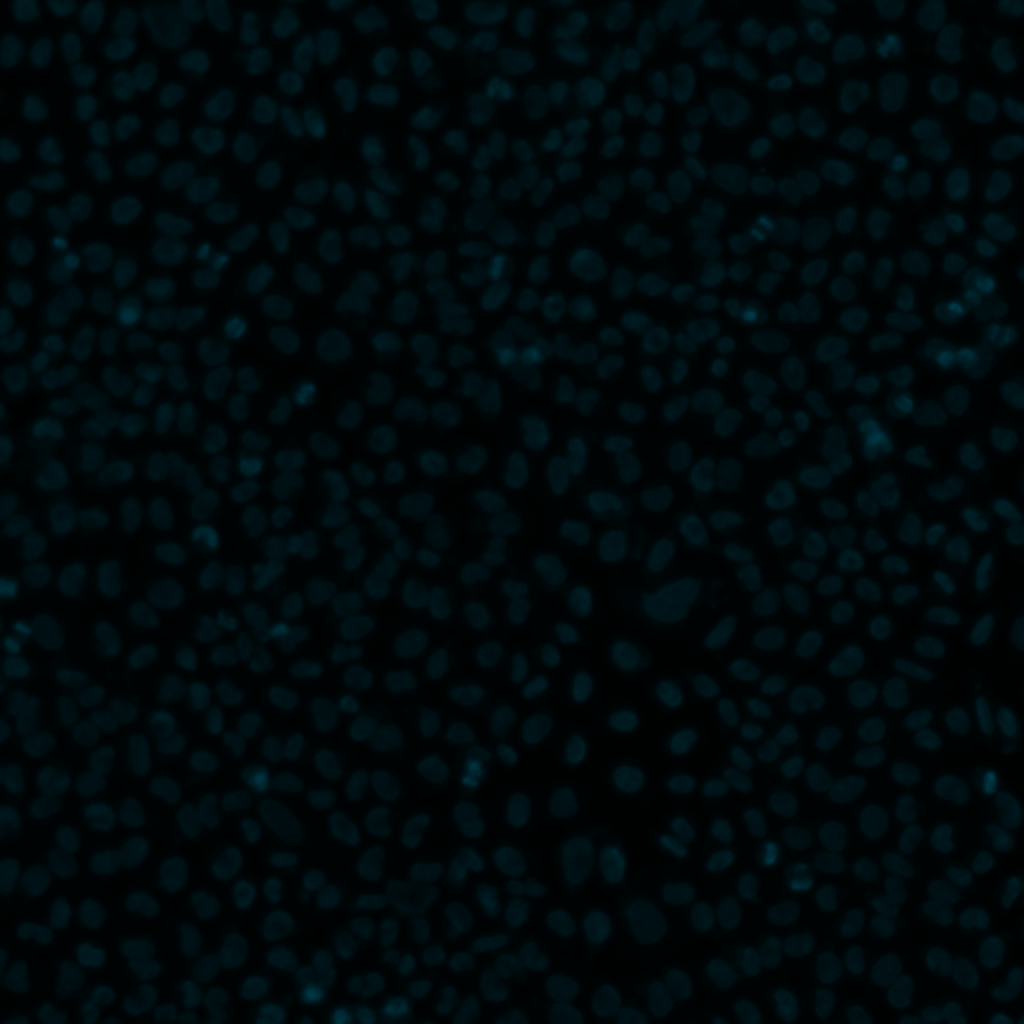

Supplement: Multimedia component 2 [file mmc2.zip › Supplemental_files/Figure 4/Figure 4F/LM3/acRoots-Reci-_Hon.tif]

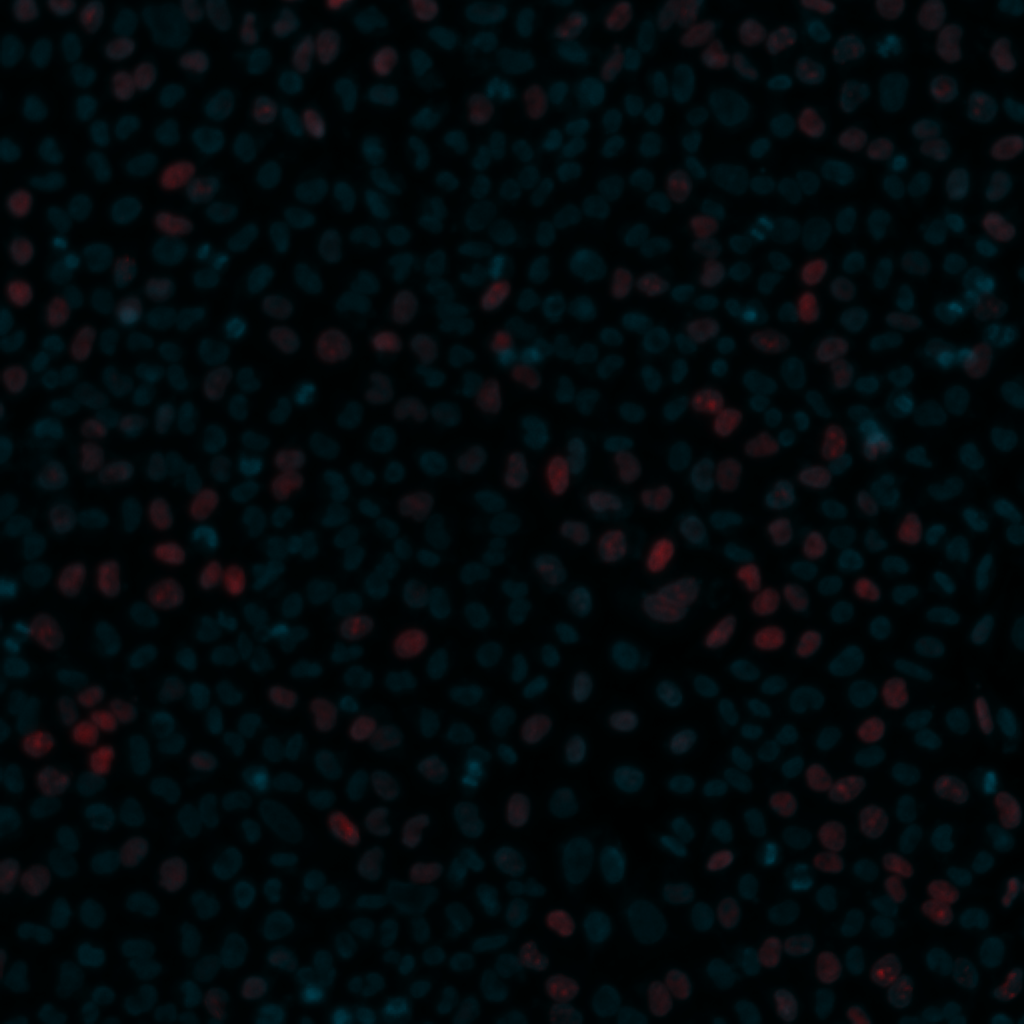

Supplement: Multimedia component 2 [file mmc2.zip › Supplemental_files/Figure 4/Figure 4F/LM3/acRoots-Reci-_merge.tif]

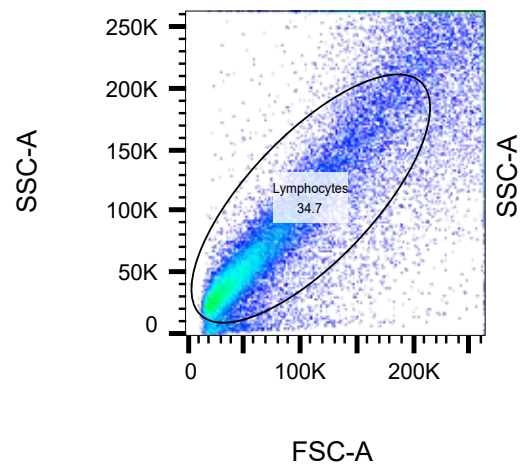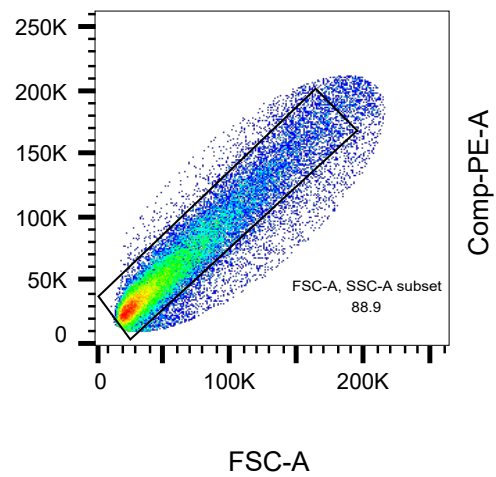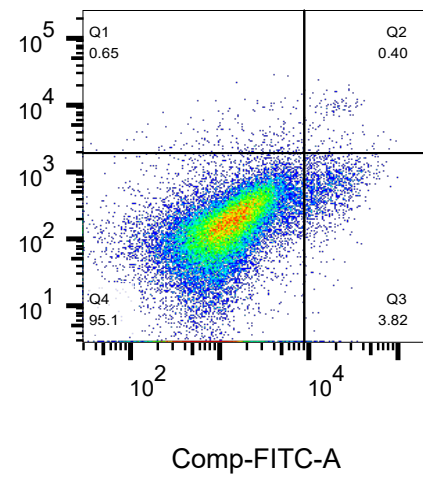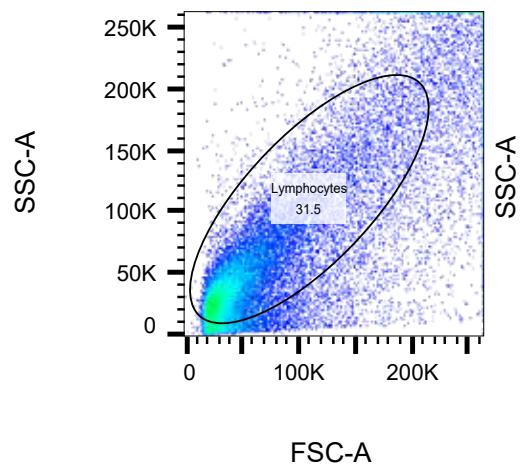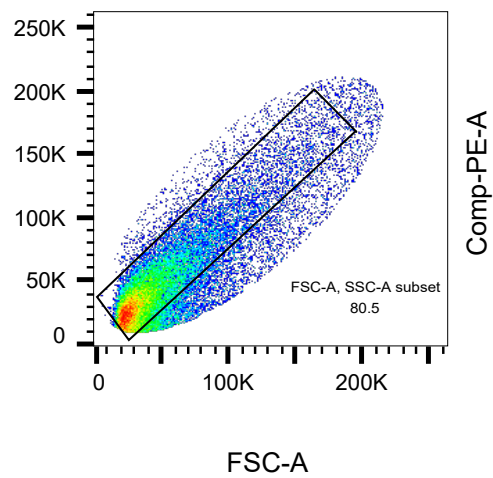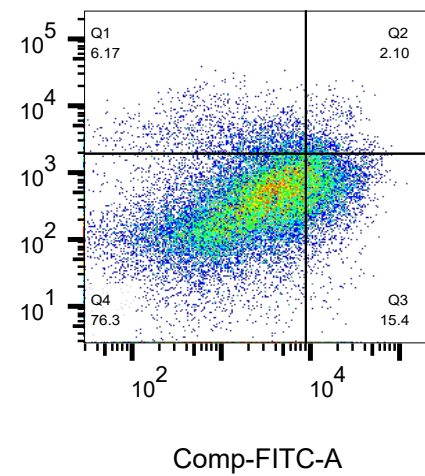

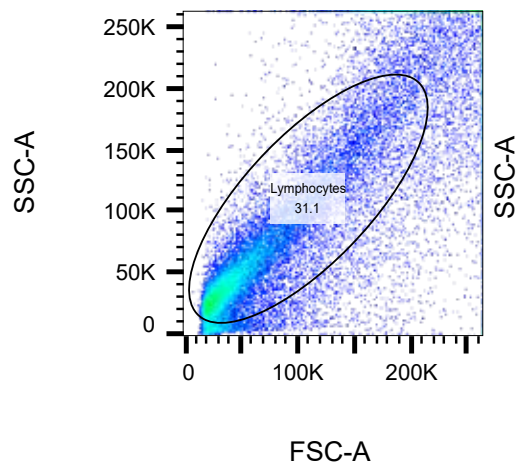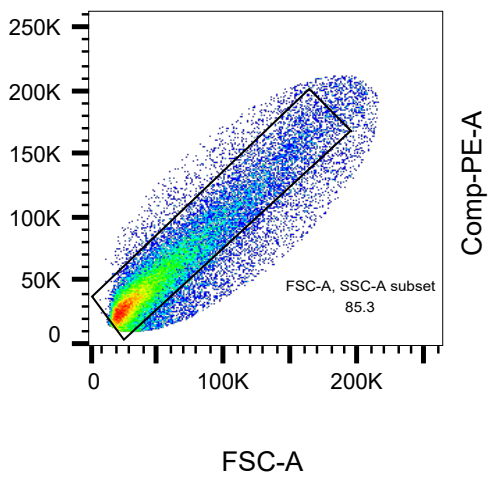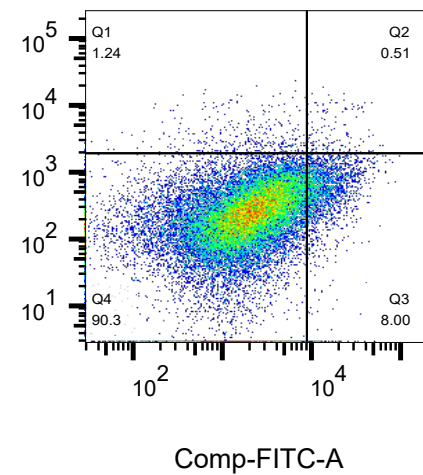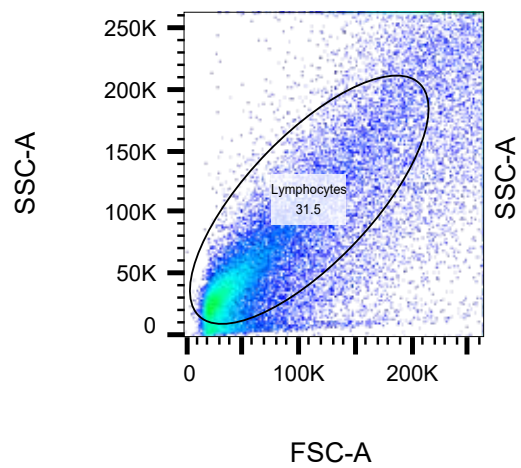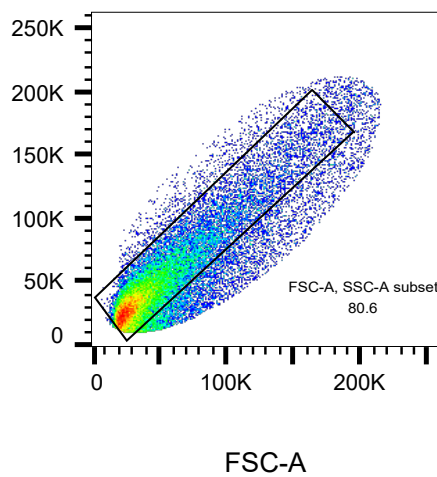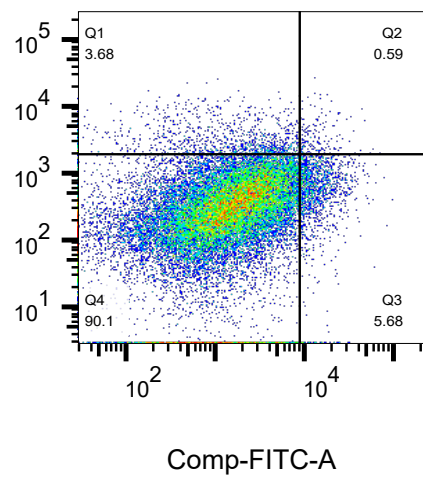

Supplement: Multimedia component 2 [file mmc2.zip › Supplemental_files/Figure 4/Figure 4G/HepG2.pdf]

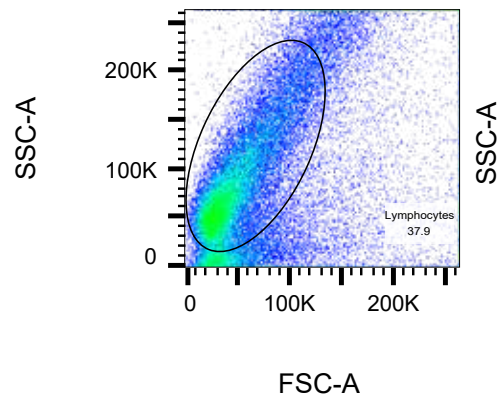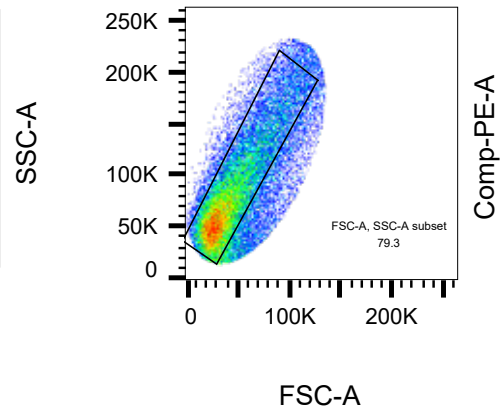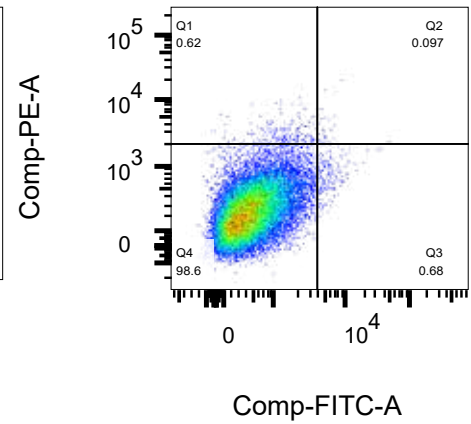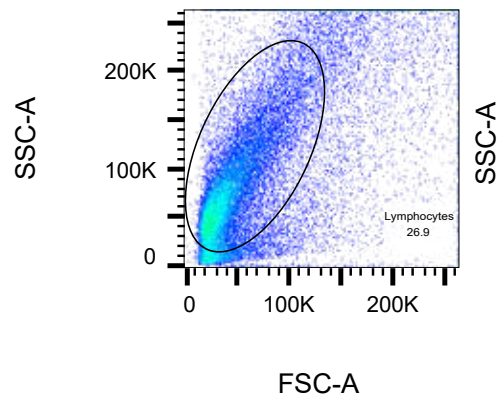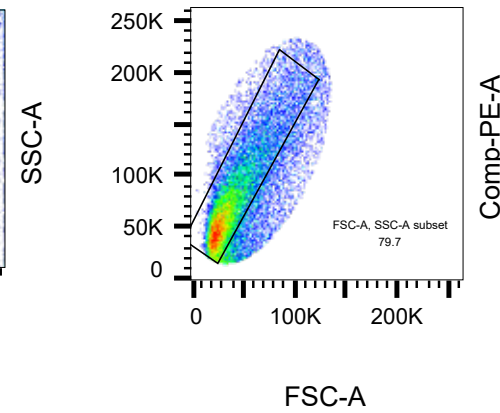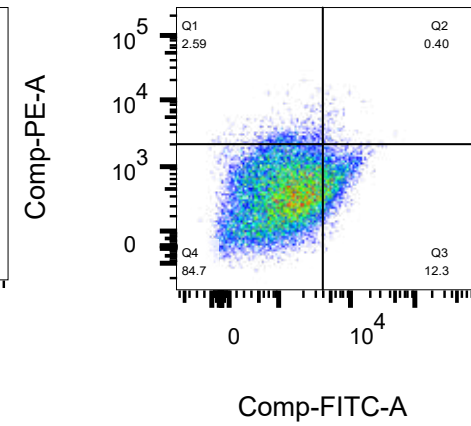

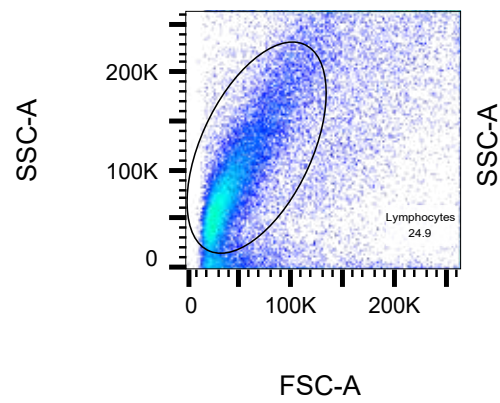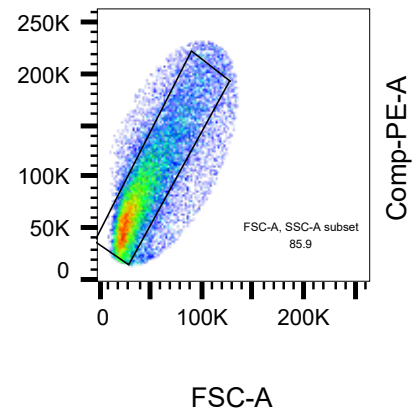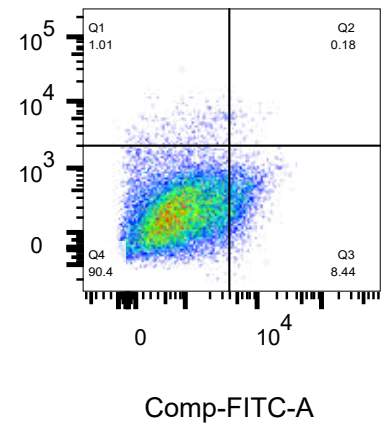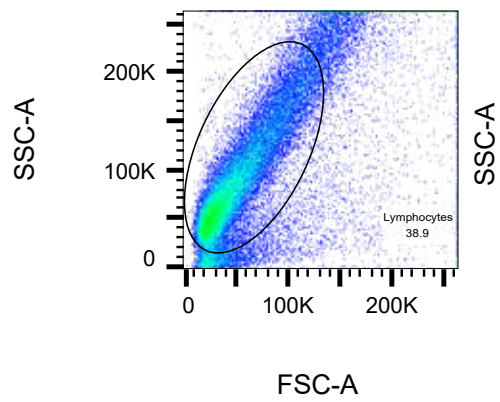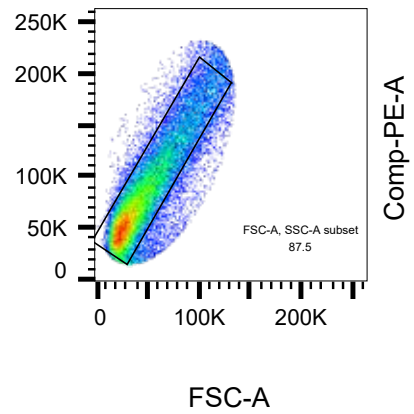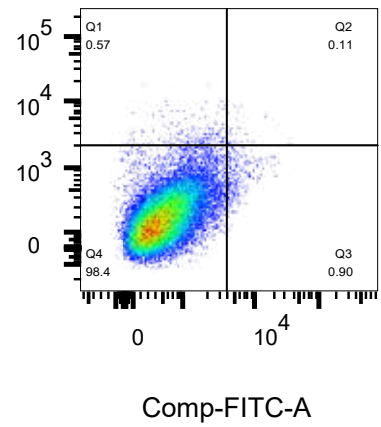

Supplement: Multimedia component 2 [file mmc2.zip › Supplemental_files/Figure 4/Figure 4G/LM3.pdf]

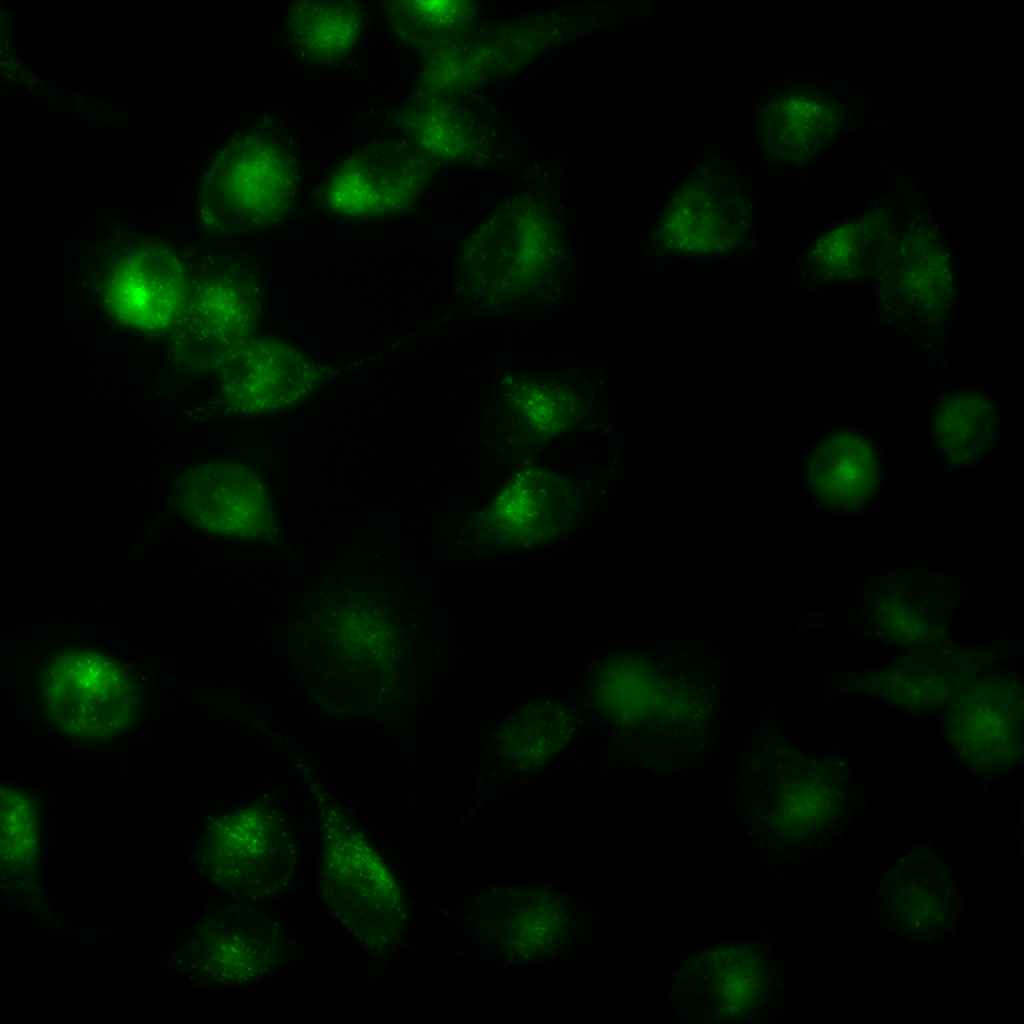

Supplement: Multimedia component 2 [file mmc2.zip › Supplemental_files/Figure 4/Figure 4H/HepG2/acR+_rec+.tif]

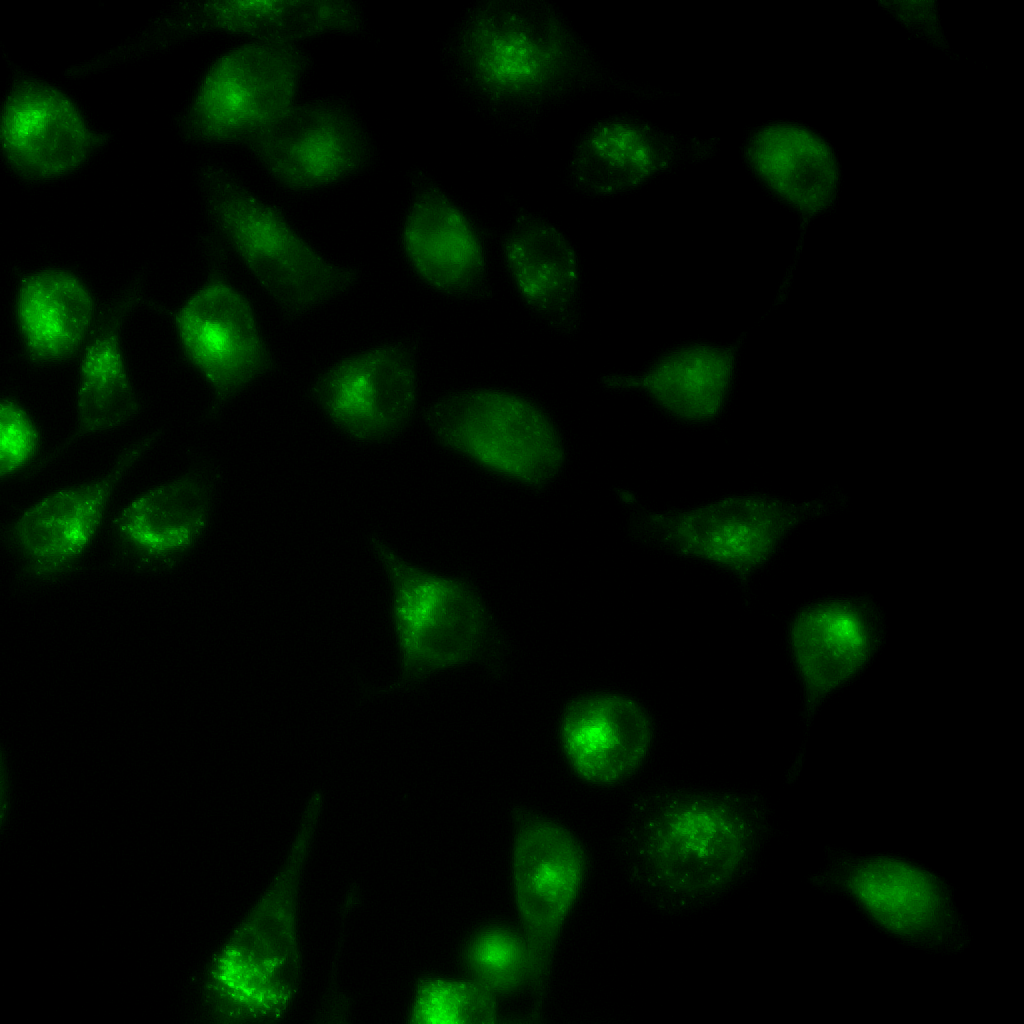

Supplement: Multimedia component 2 [file mmc2.zip › Supplemental_files/Figure 4/Figure 4H/HepG2/acR+_rec-.tif]

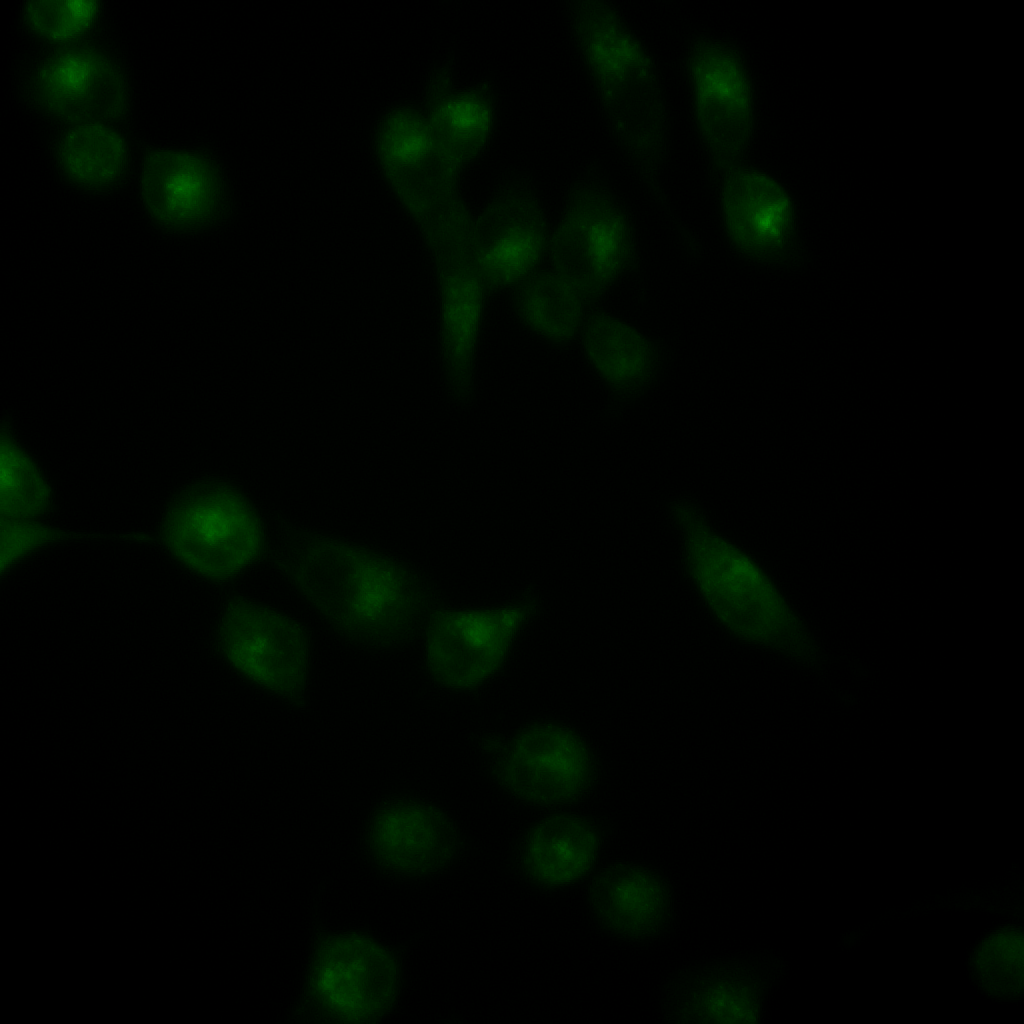

Supplement: Multimedia component 2 [file mmc2.zip › Supplemental_files/Figure 4/Figure 4H/HepG2/acR-_rec+.tif]

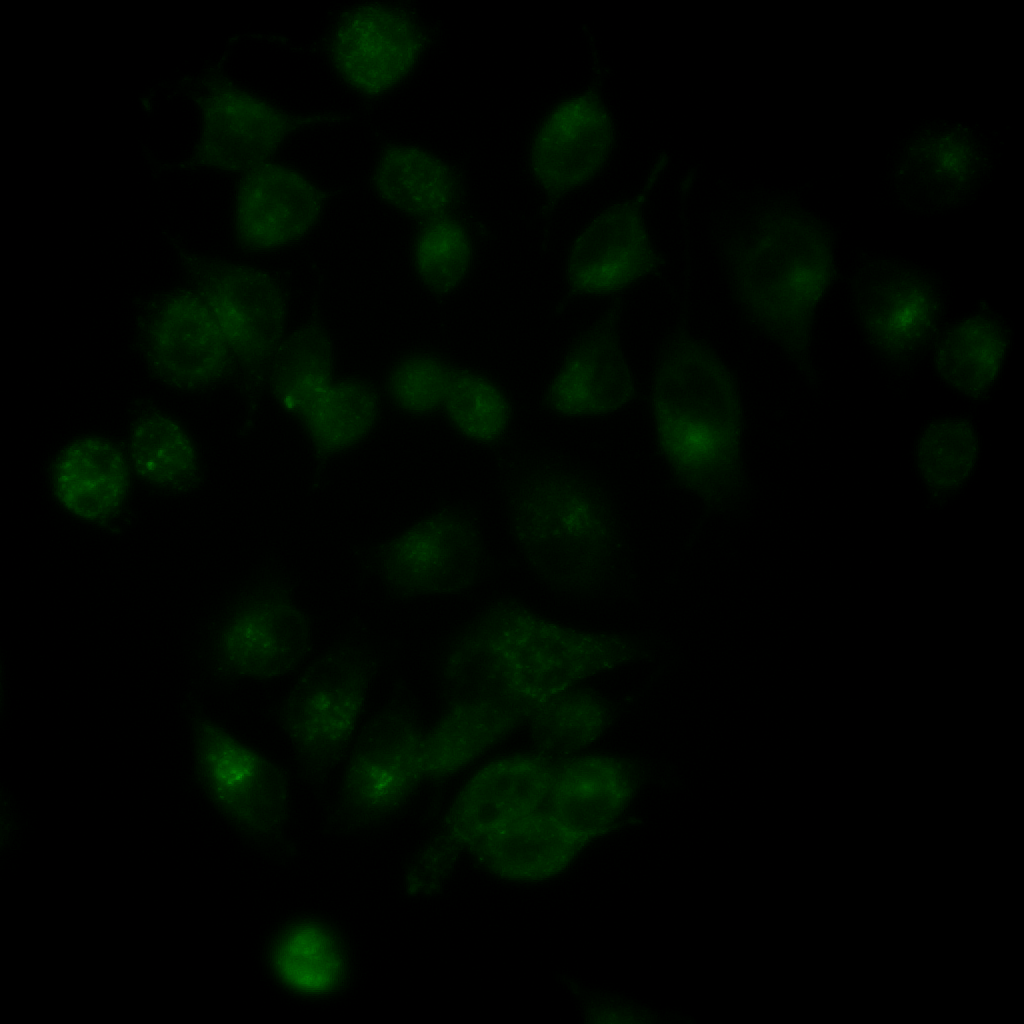

Supplement: Multimedia component 2 [file mmc2.zip › Supplemental_files/Figure 4/Figure 4H/HepG2/acR-_rec-.tif]

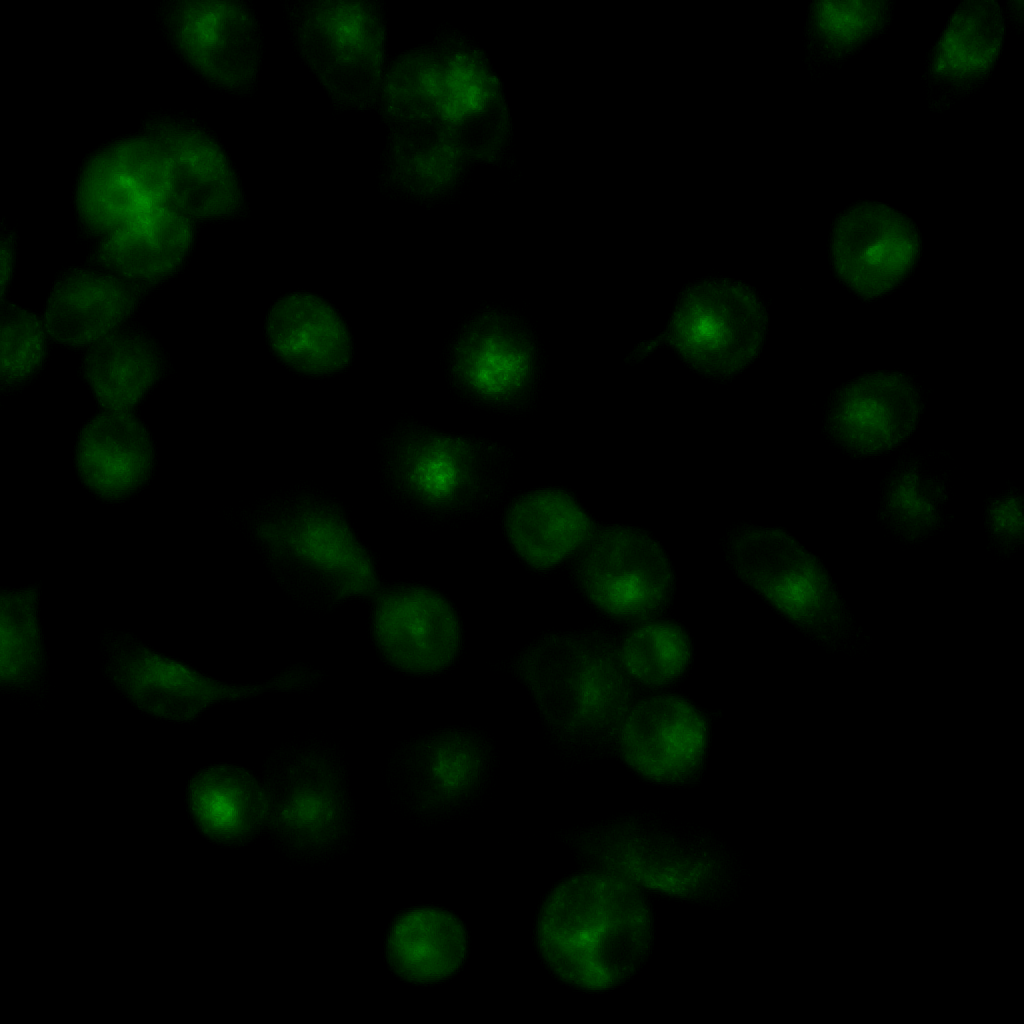

Supplement: Multimedia component 2 [file mmc2.zip › Supplemental_files/Figure 4/Figure 4H/LM3/acR+_Rec+.tif]

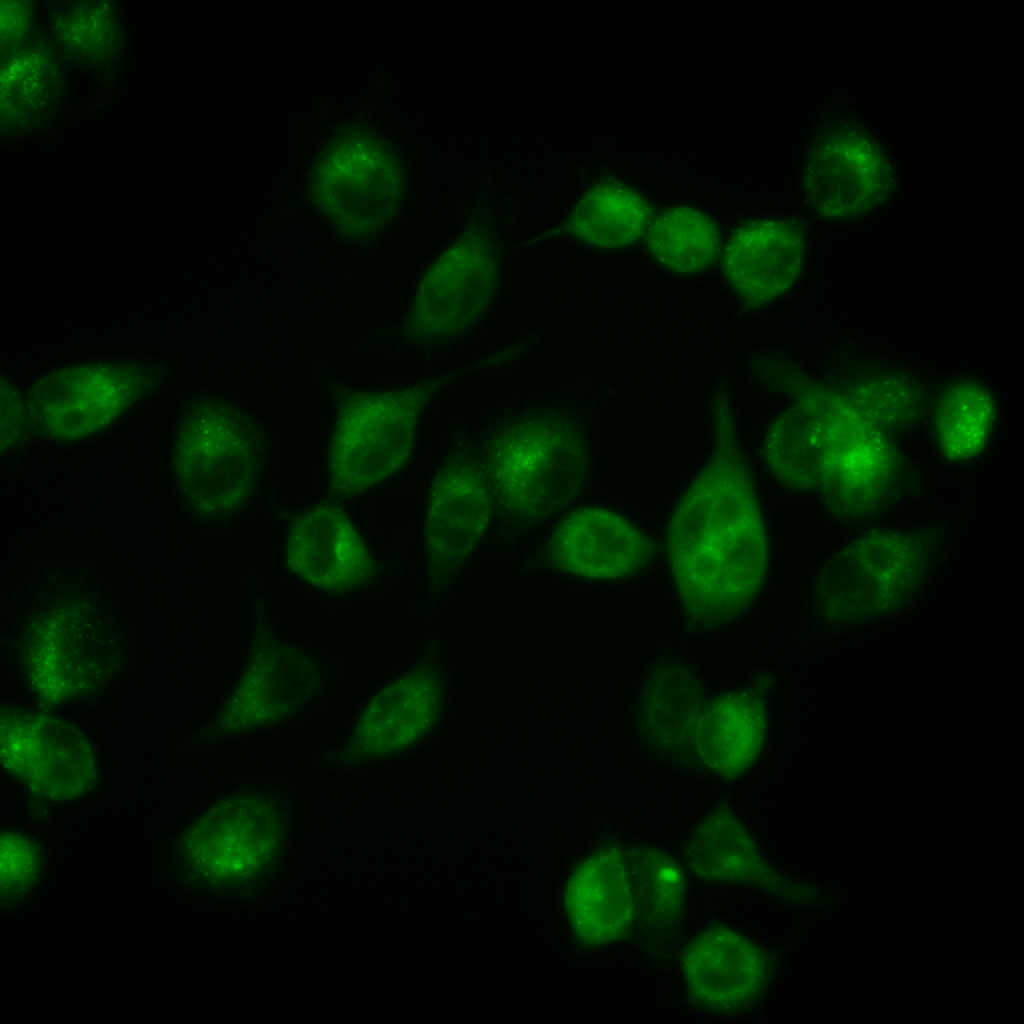

Supplement: Multimedia component 2 [file mmc2.zip › Supplemental_files/Figure 4/Figure 4H/LM3/acR+_rec-.tif]

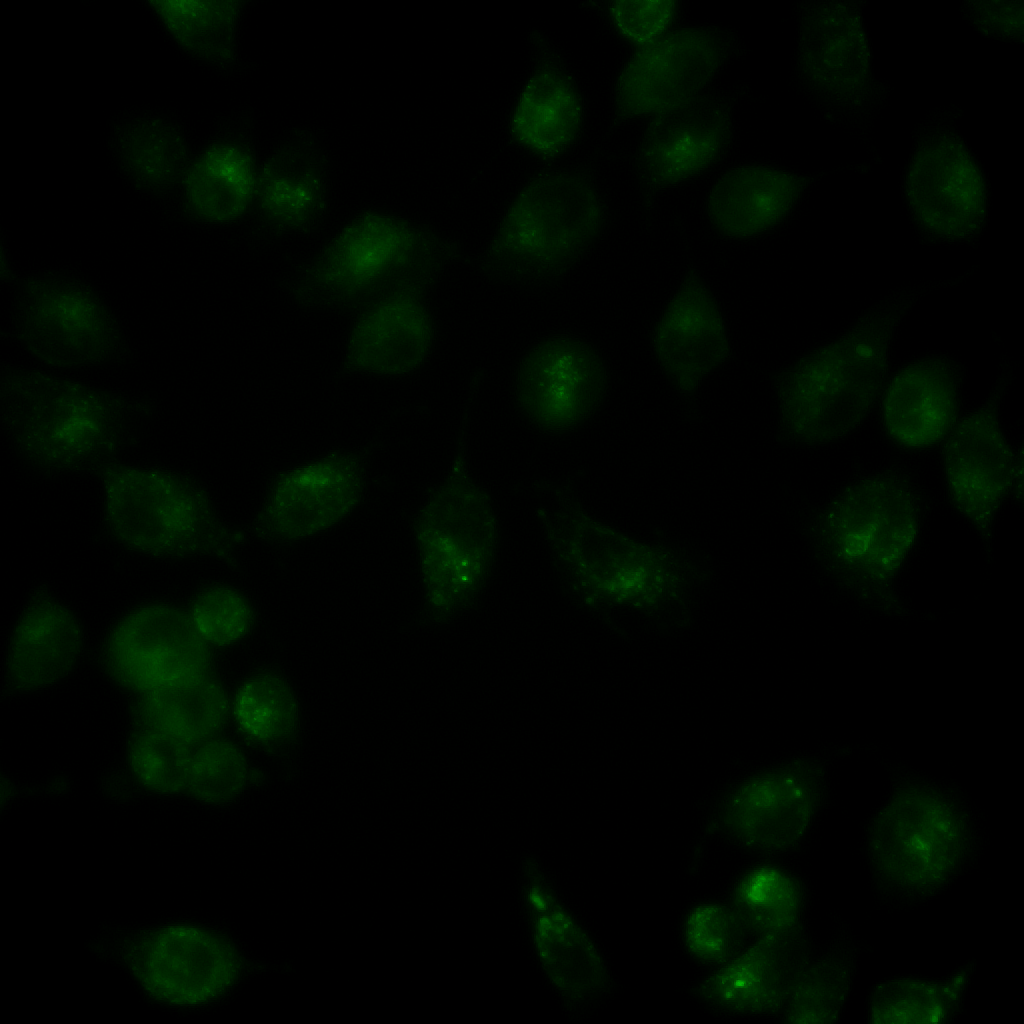

Supplement: Multimedia component 2 [file mmc2.zip › Supplemental_files/Figure 4/Figure 4H/LM3/acR-_Rec-.tif]

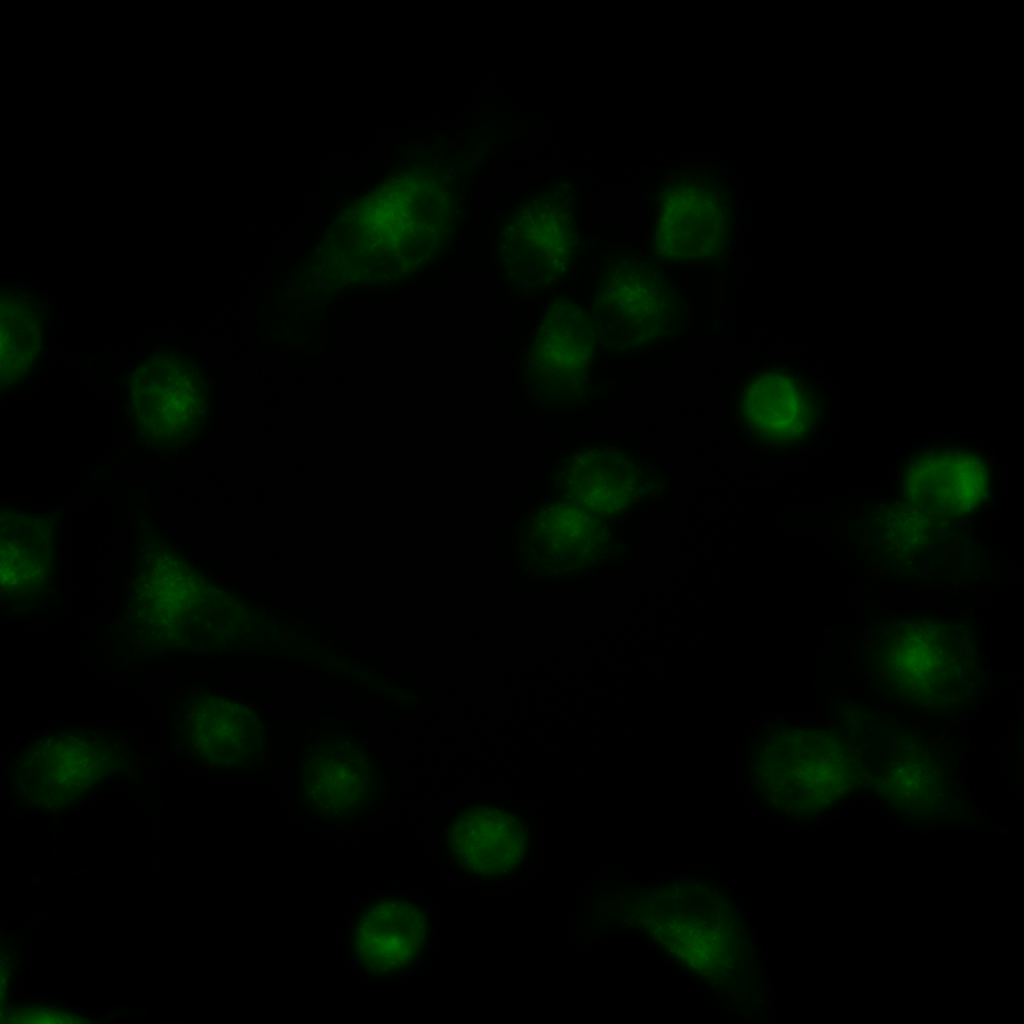

Supplement: Multimedia component 2 [file mmc2.zip › Supplemental_files/Figure 4/Figure 4H/LM3/acR-_rec+.tif]
