# Supplementary figures and images for: Exploring the anticancer potential of Actinidia chinensis Planch root extracts (acRoots) on hepatocellular carcinoma: A molecular mechanism study (part 1 of 2)
Source: Heliyon. 2023 Nov 2;9(11):e21851. doi: 10.1016/j.heliyon.2023.e21851 (PMC10656260; doi:10.1016/j.heliyon.2023.e21851)

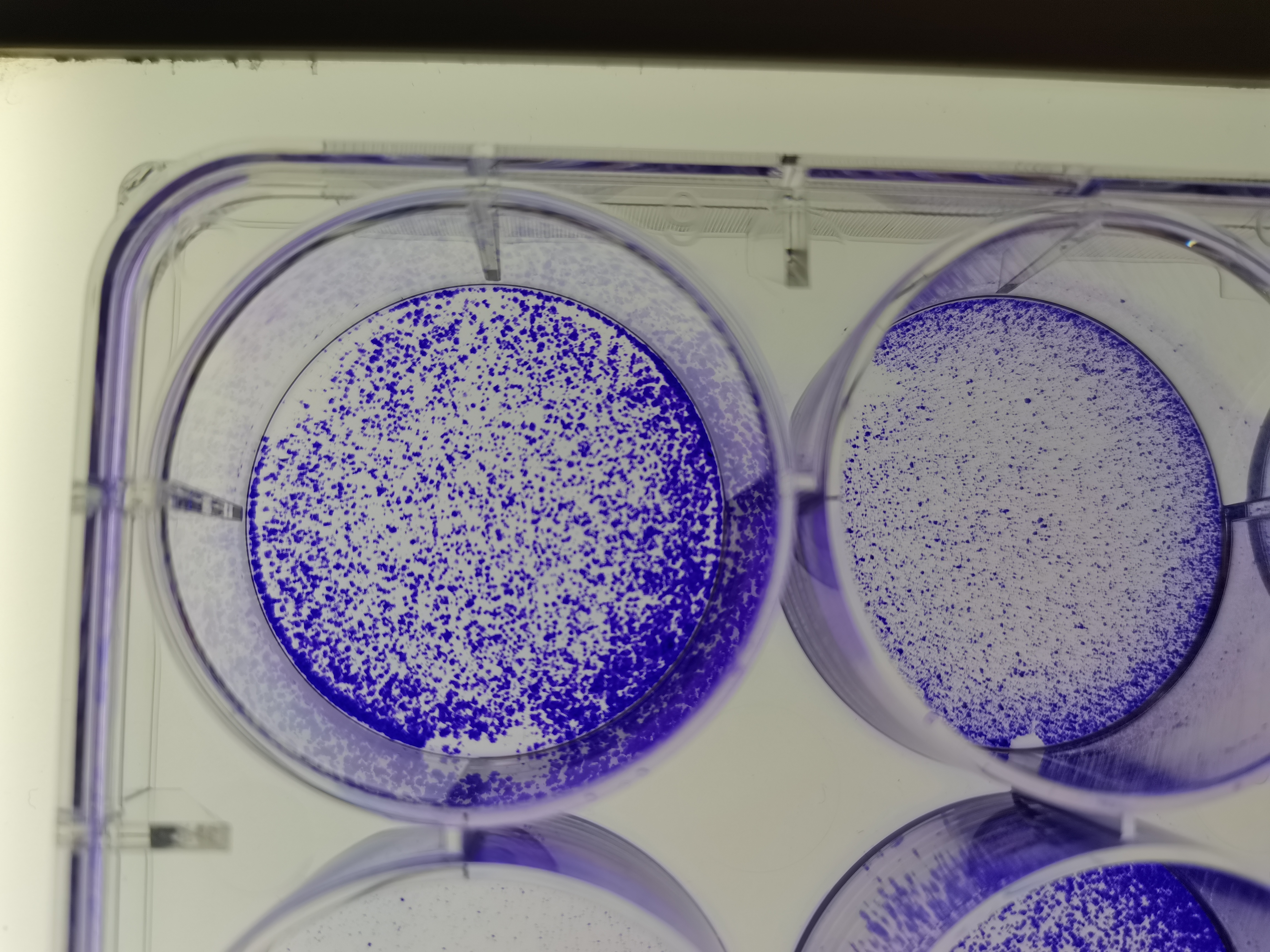

Supplement: Multimedia component 2 [file mmc2.zip › Supplemental_files/Figure 1/Figure 1D/HepG2/0.jpg]

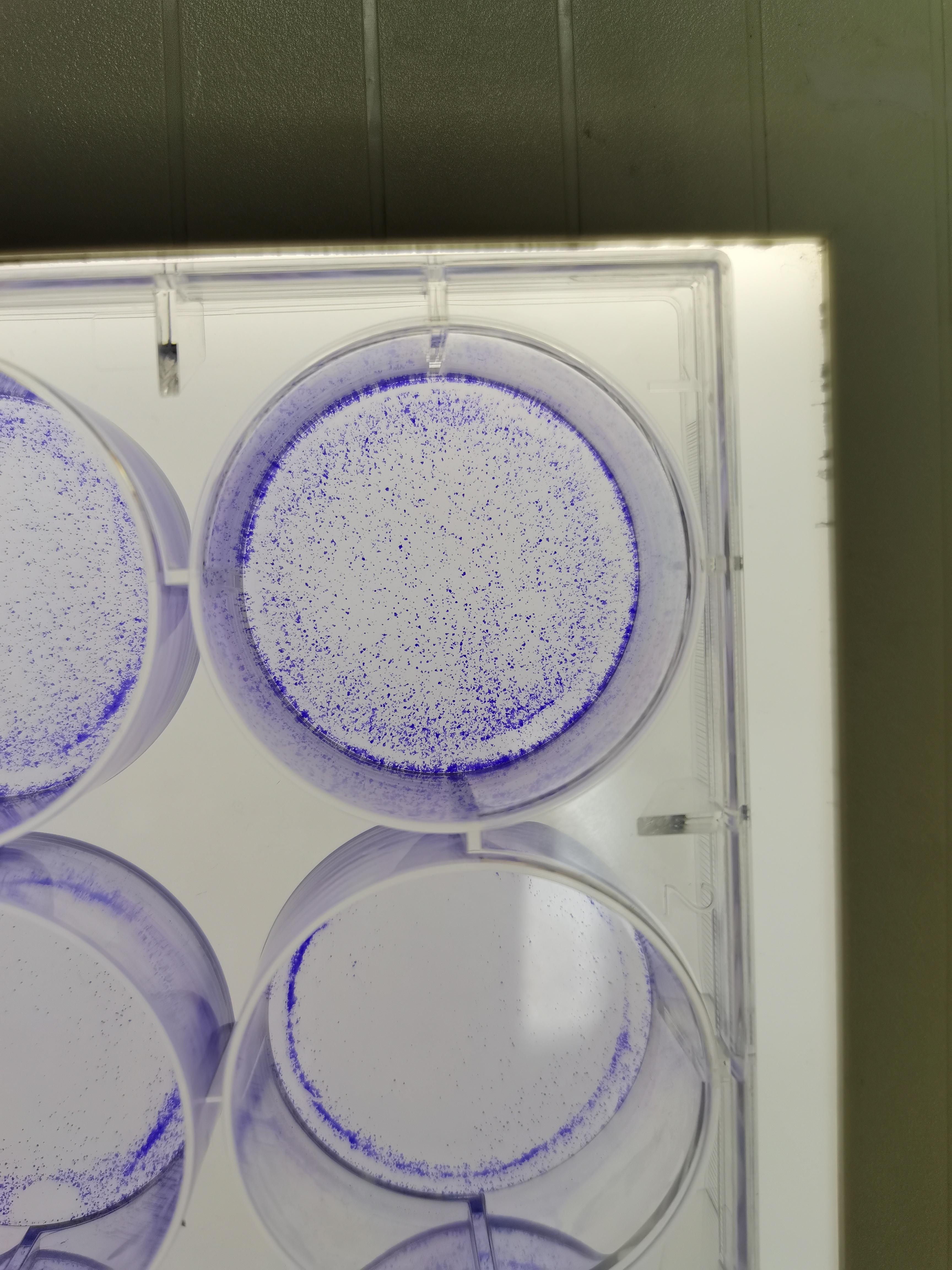

Supplement: Multimedia component 2 [file mmc2.zip › Supplemental_files/Figure 1/Figure 1D/HepG2/200.jpg]

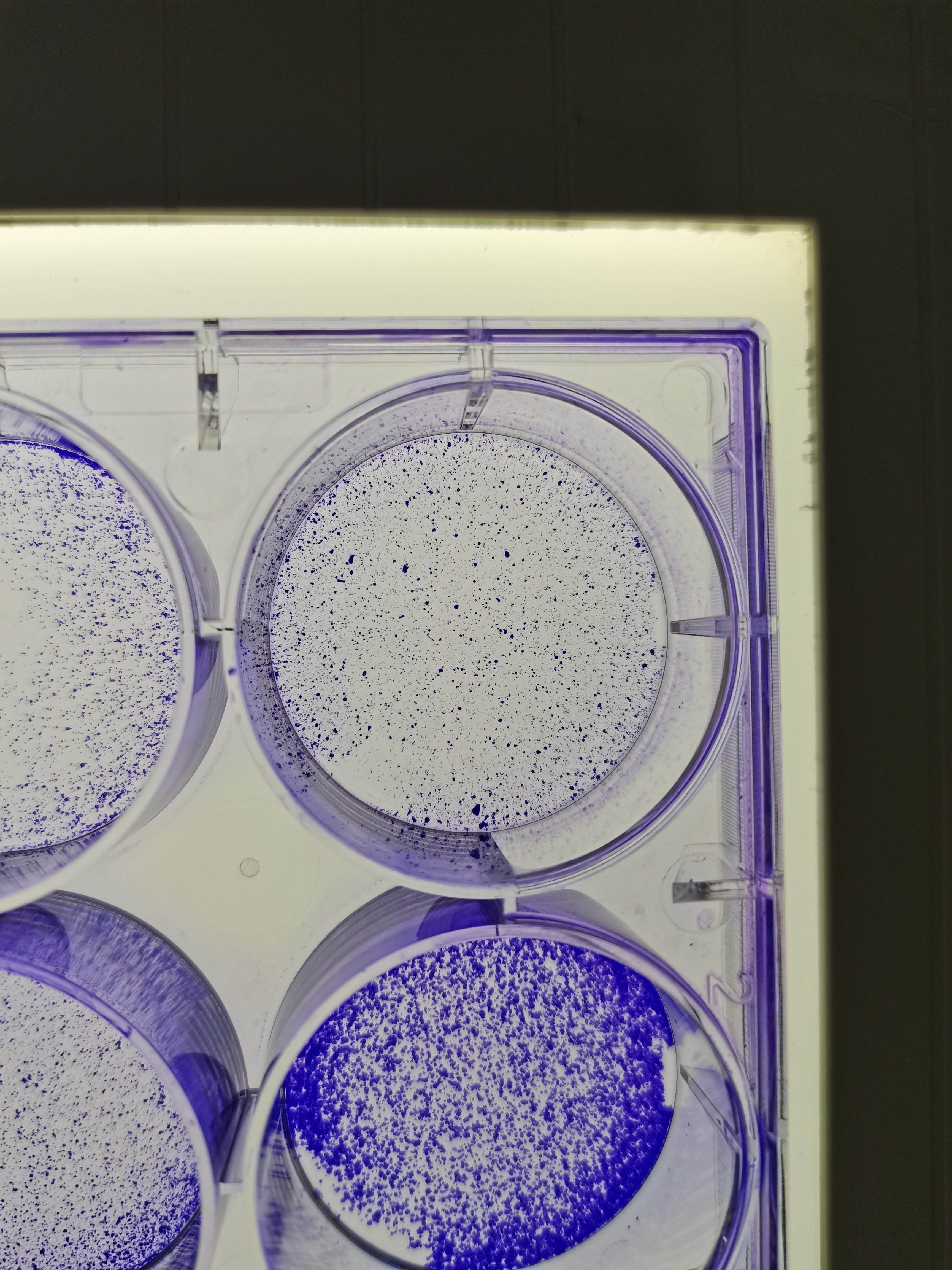

Supplement: Multimedia component 2 [file mmc2.zip › Supplemental_files/Figure 1/Figure 1D/HepG2/450.jpg]

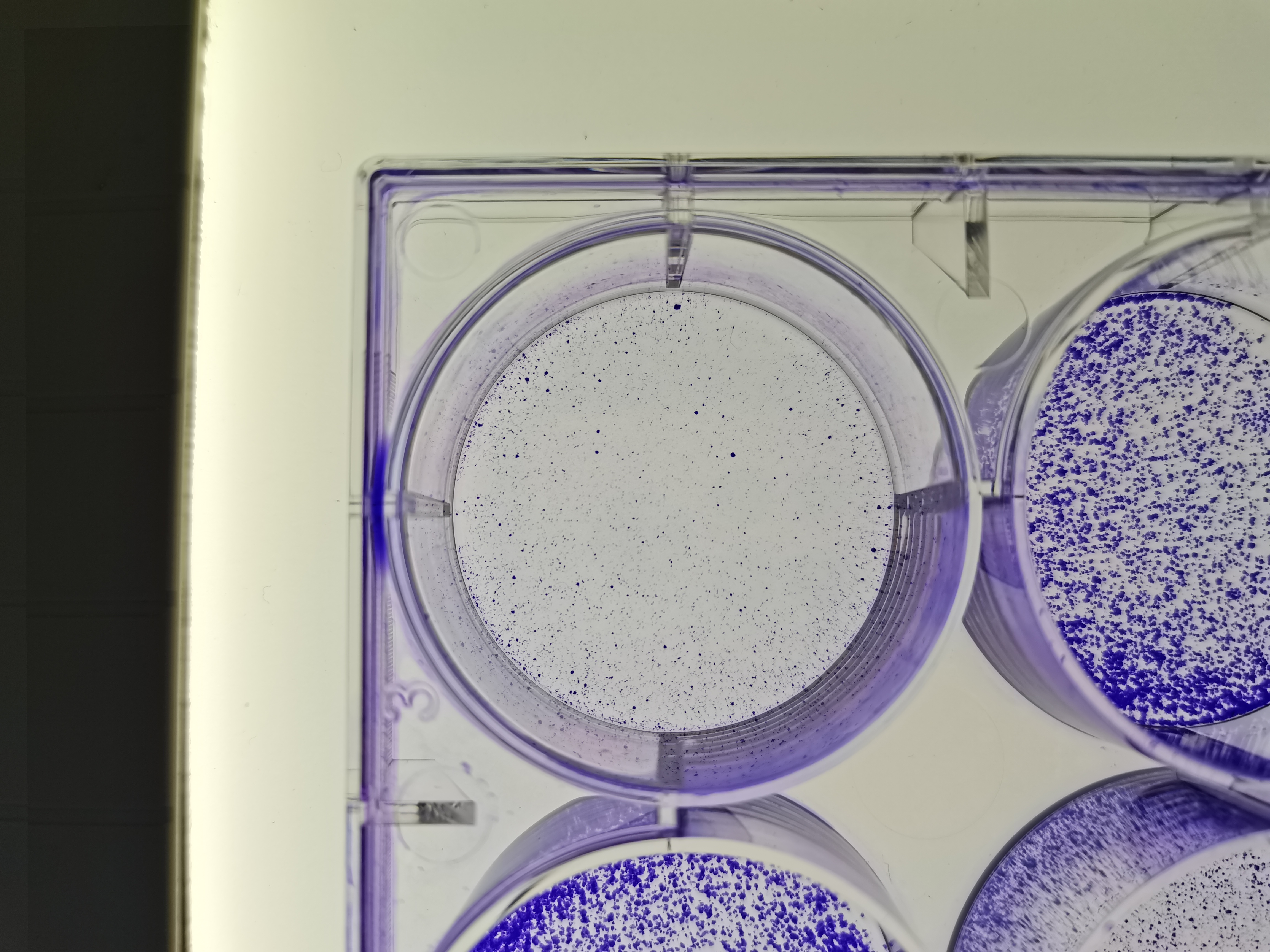

Supplement: Multimedia component 2 [file mmc2.zip › Supplemental_files/Figure 1/Figure 1D/HepG2/600.jpg]

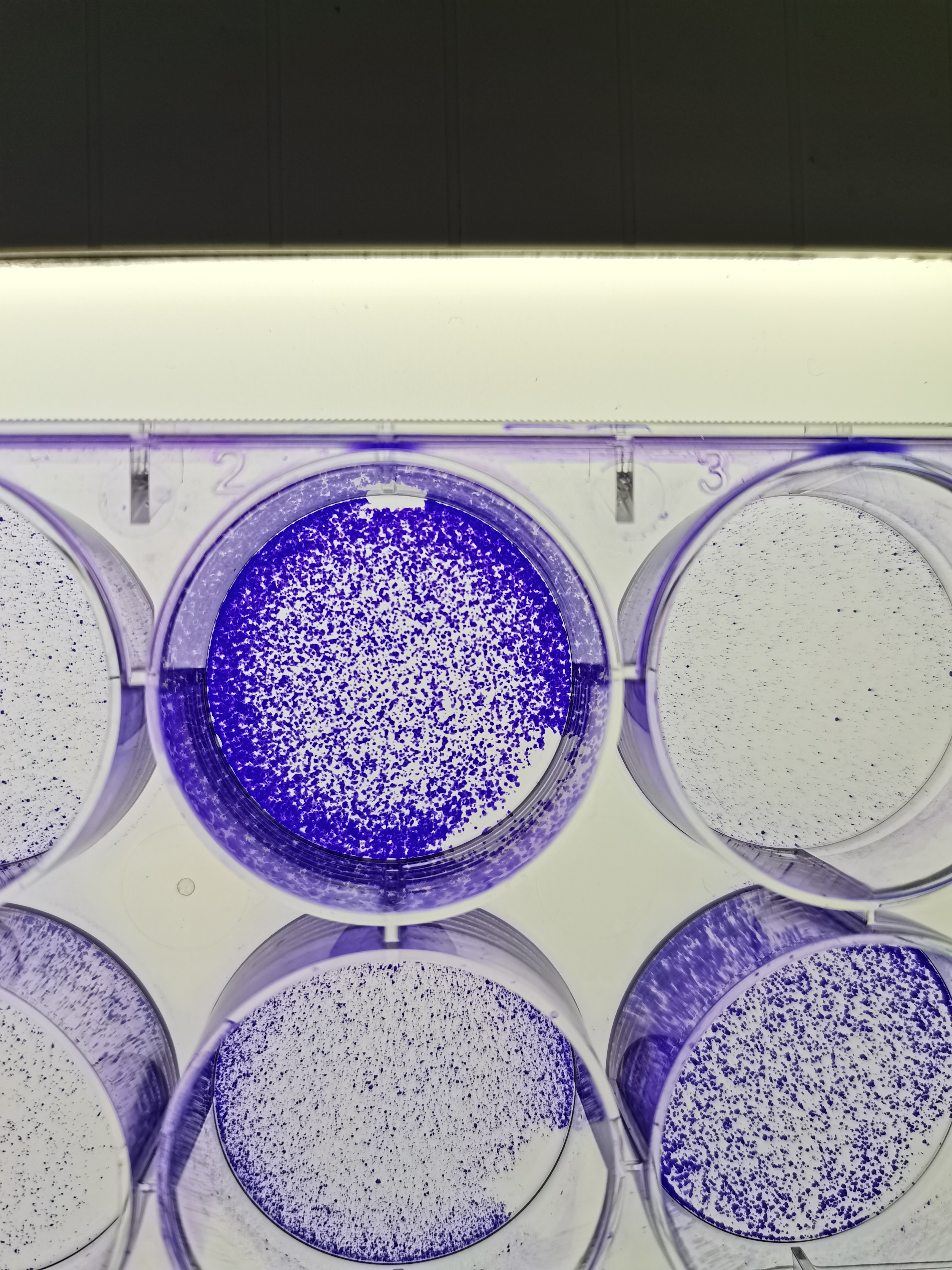

Supplement: Multimedia component 2 [file mmc2.zip › Supplemental_files/Figure 1/Figure 1D/LM3/0.jpg]

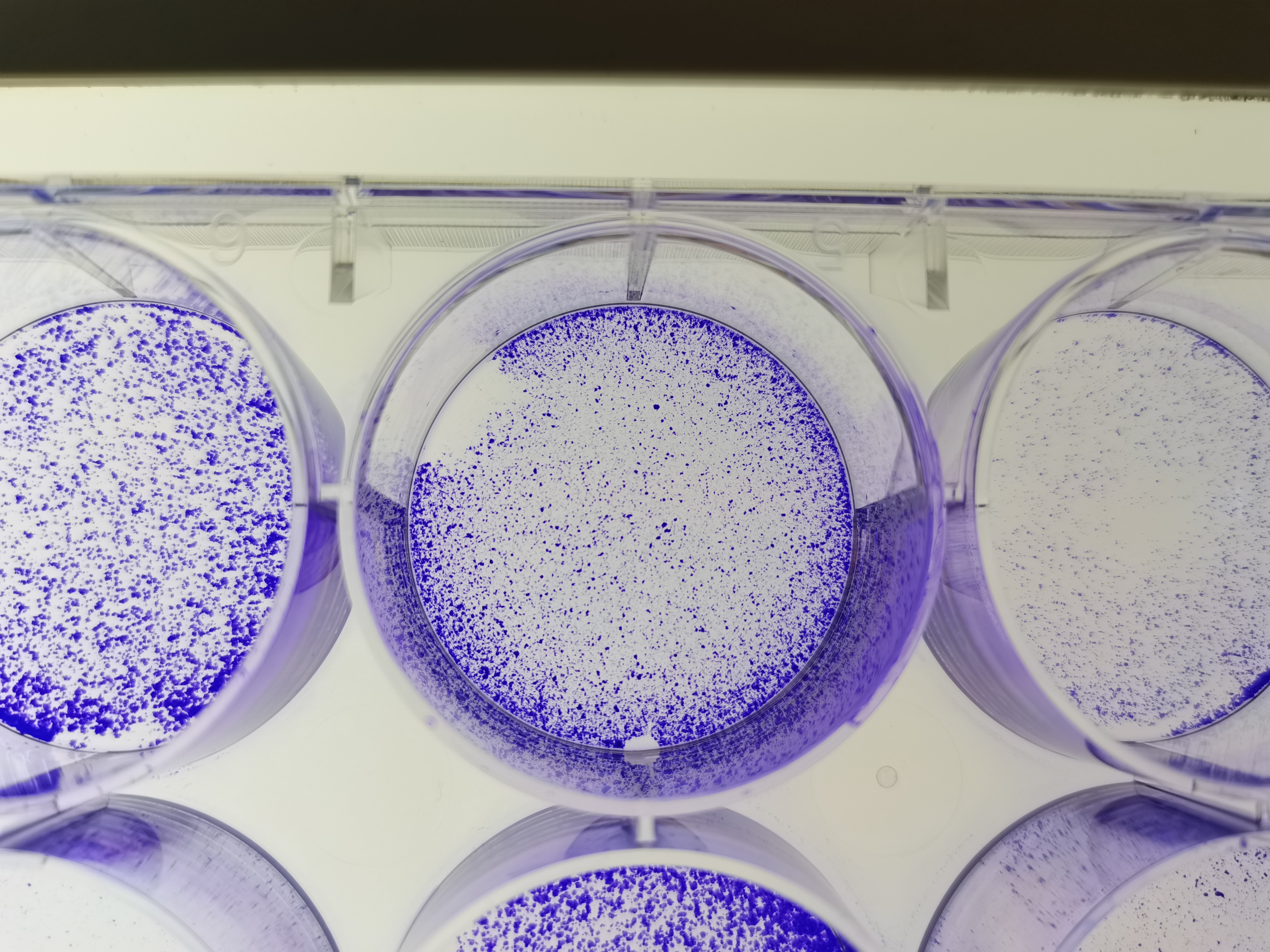

Supplement: Multimedia component 2 [file mmc2.zip › Supplemental_files/Figure 1/Figure 1D/LM3/200.jpg]

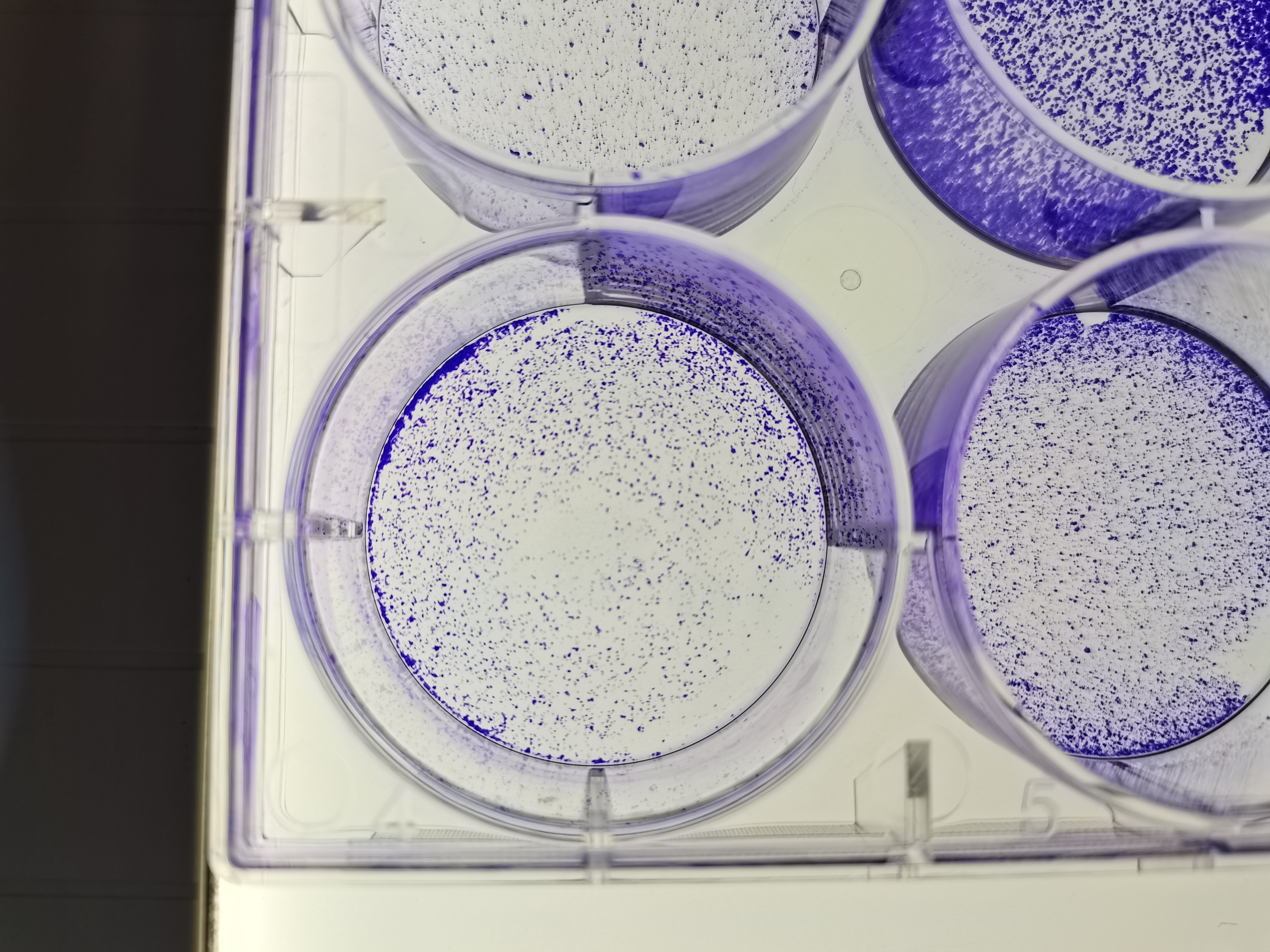

Supplement: Multimedia component 2 [file mmc2.zip › Supplemental_files/Figure 1/Figure 1D/LM3/450 .jpg]

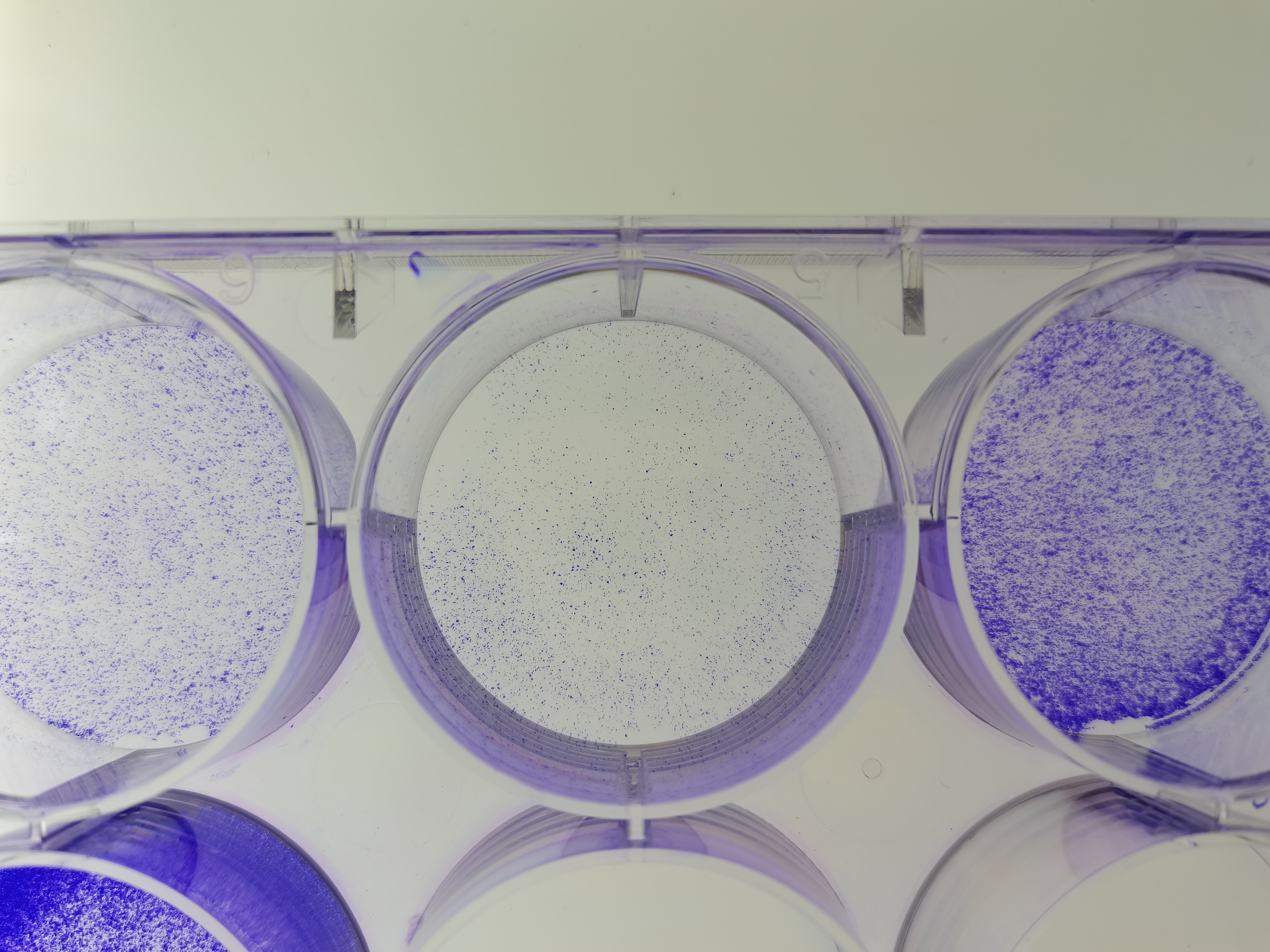

Supplement: Multimedia component 2 [file mmc2.zip › Supplemental_files/Figure 1/Figure 1D/LM3/600.jpg]

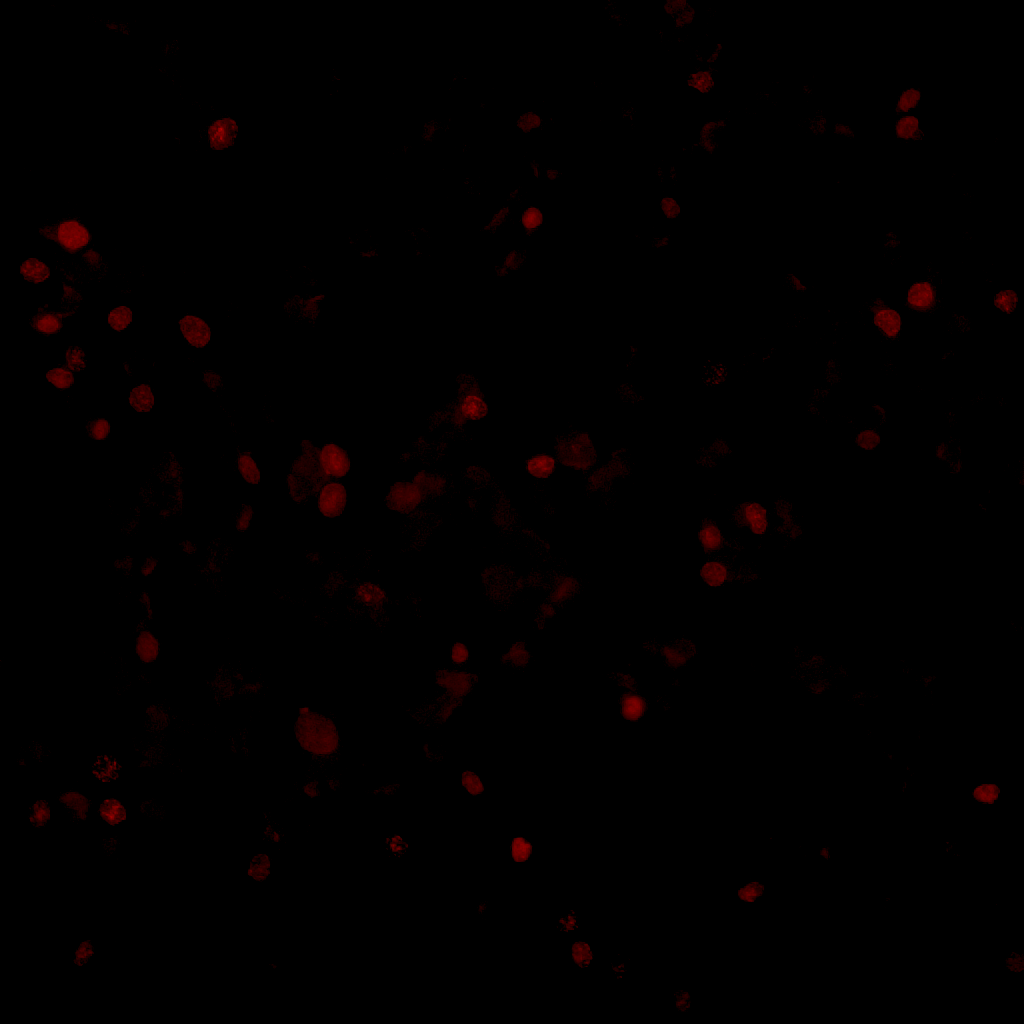

Supplement: Multimedia component 2 [file mmc2.zip › Supplemental_files/Figure 1/Figure 1G/HepG2/0_Edu.tif]

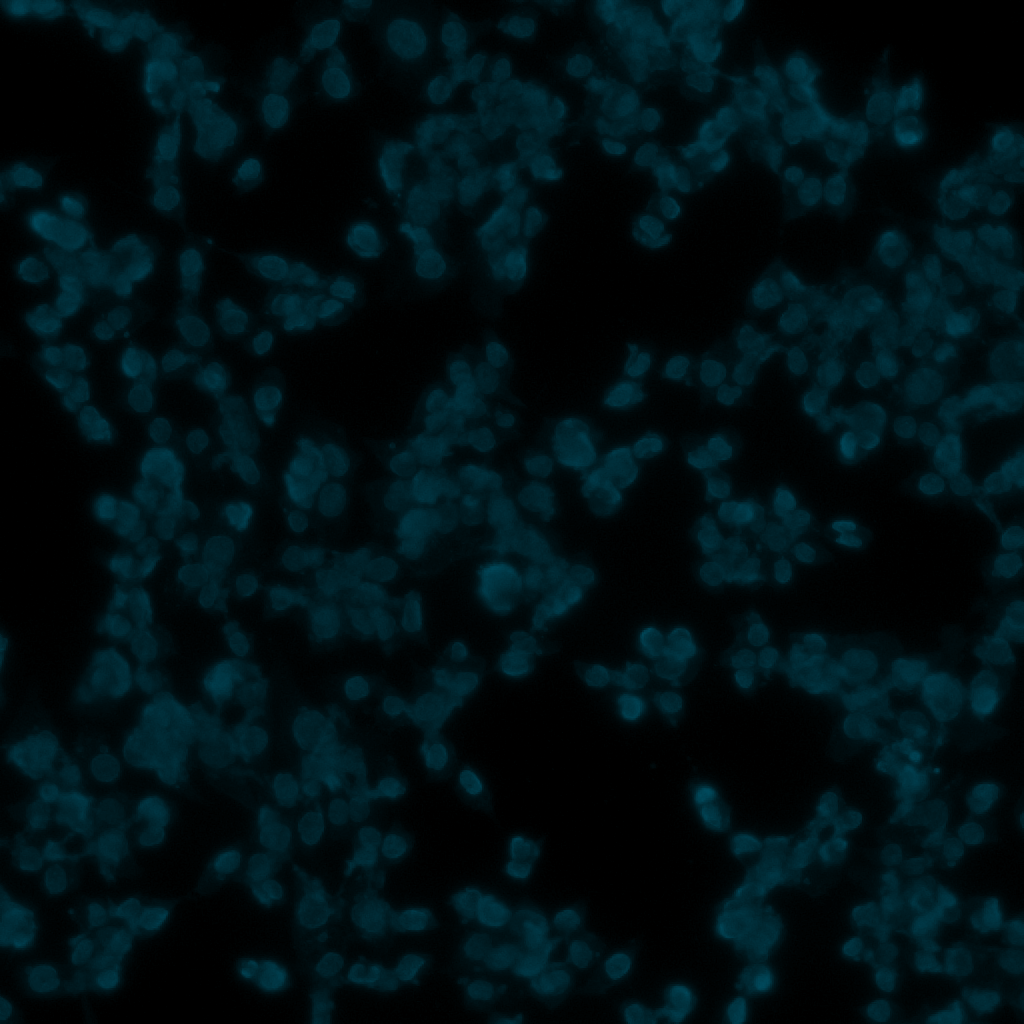

Supplement: Multimedia component 2 [file mmc2.zip › Supplemental_files/Figure 1/Figure 1G/HepG2/0_Hon.tif]

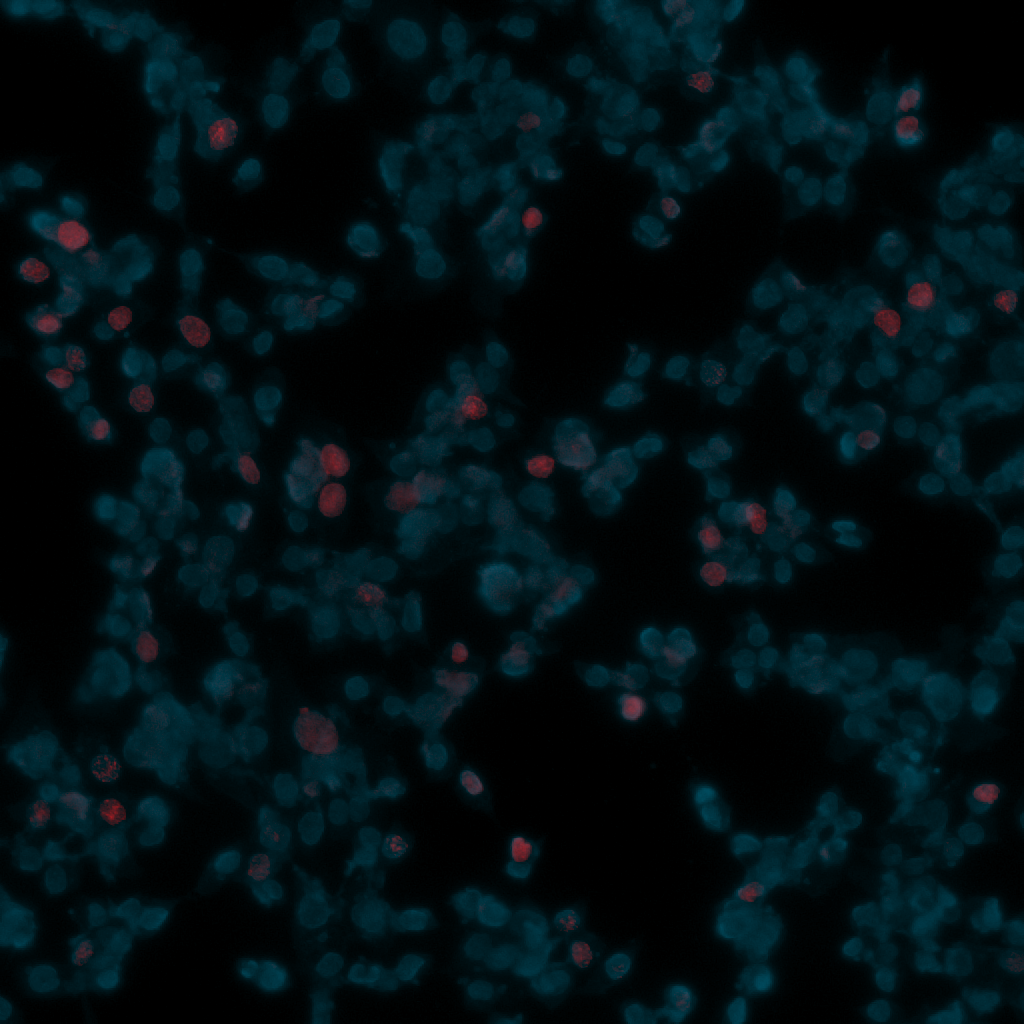

Supplement: Multimedia component 2 [file mmc2.zip › Supplemental_files/Figure 1/Figure 1G/HepG2/0_merge.tif]

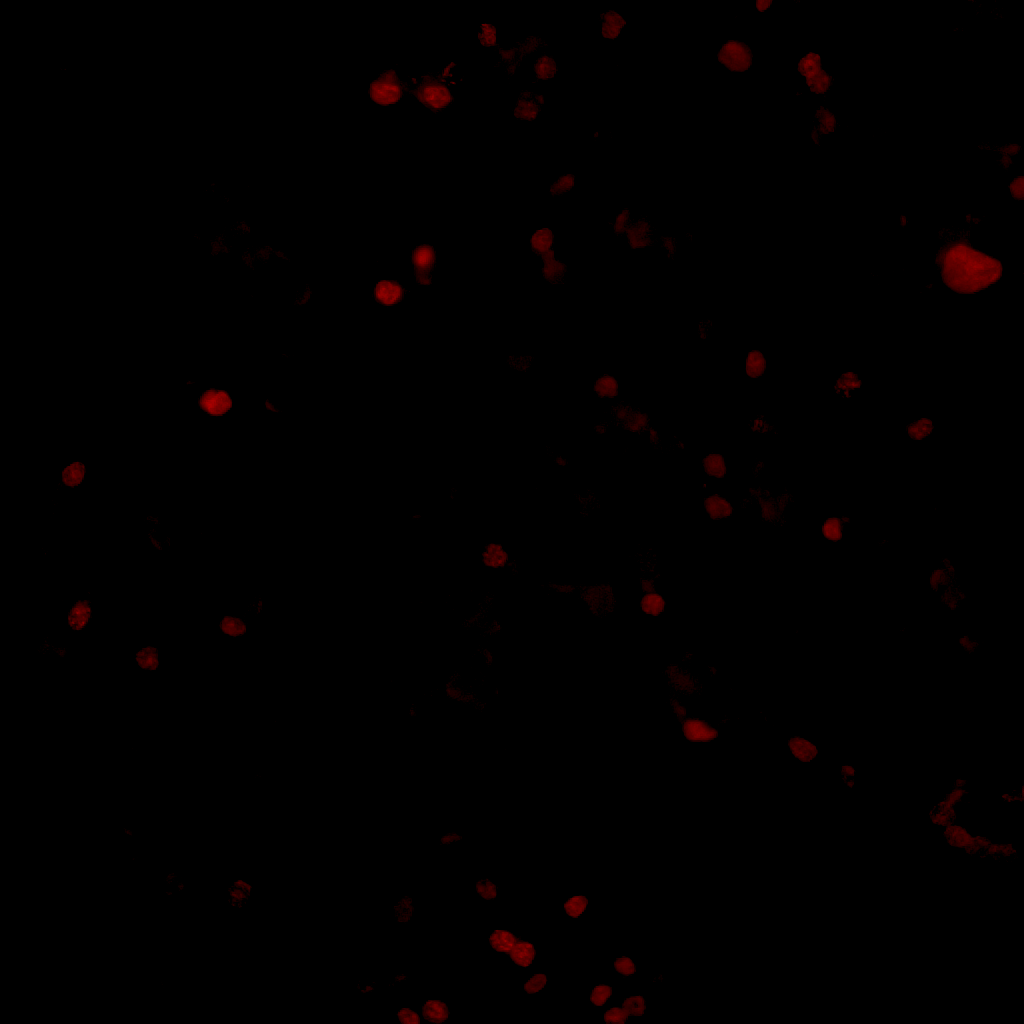

Supplement: Multimedia component 2 [file mmc2.zip › Supplemental_files/Figure 1/Figure 1G/HepG2/200_Edu.tif]

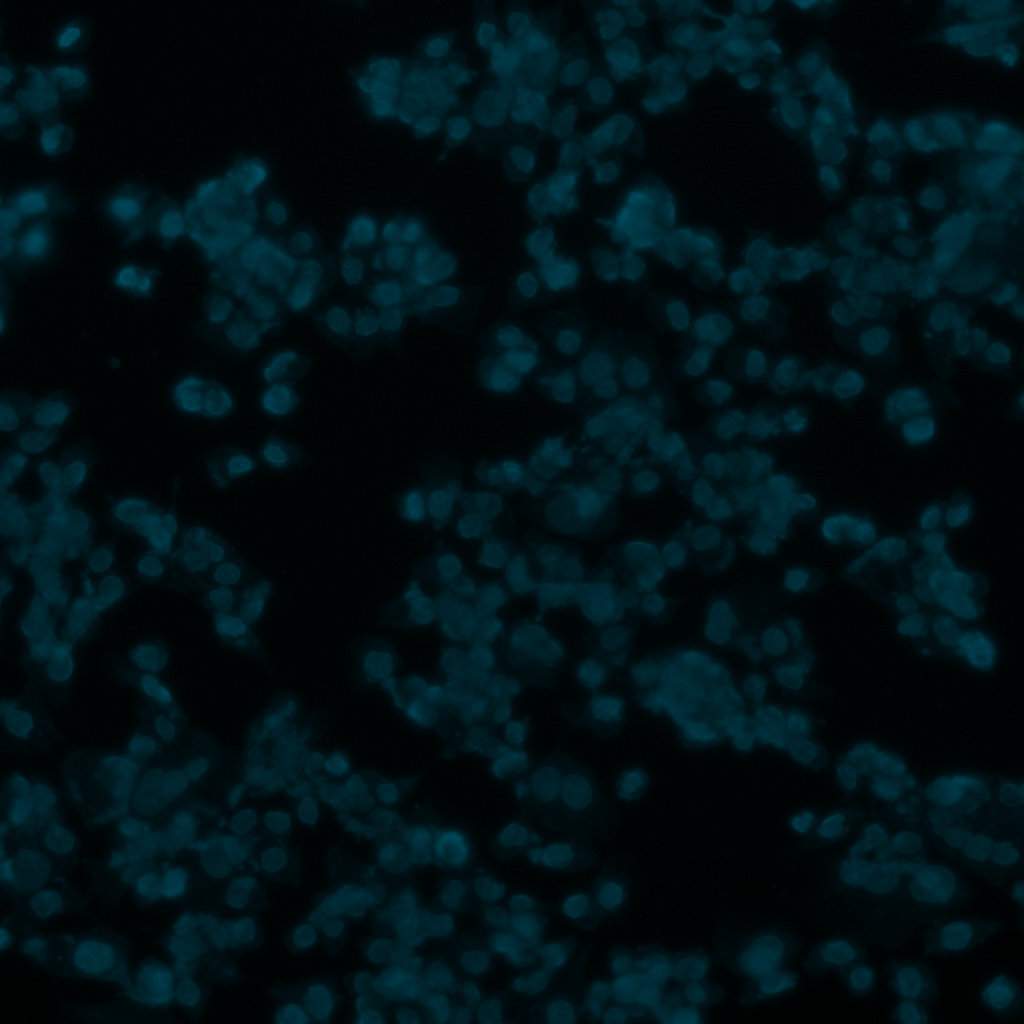

Supplement: Multimedia component 2 [file mmc2.zip › Supplemental_files/Figure 1/Figure 1G/HepG2/200_Hon.tif]

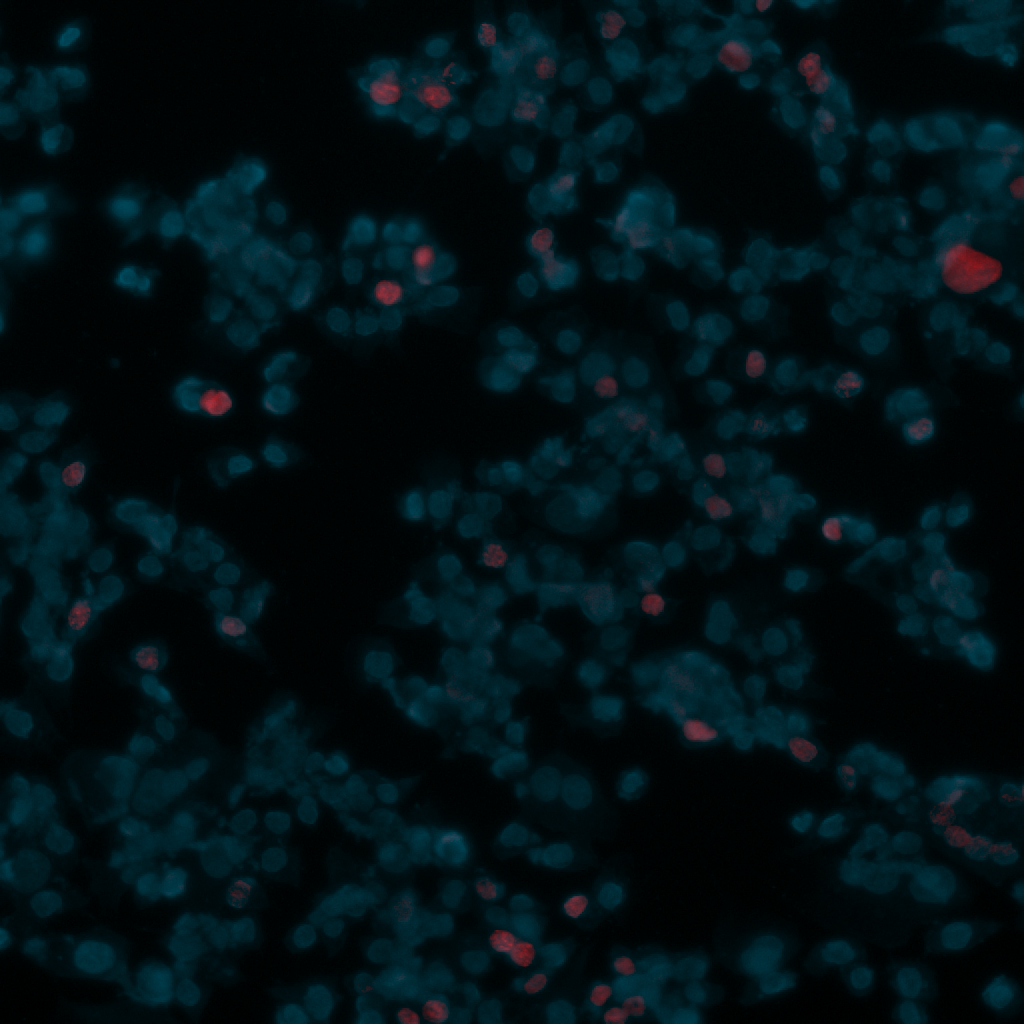

Supplement: Multimedia component 2 [file mmc2.zip › Supplemental_files/Figure 1/Figure 1G/HepG2/200_merge.tif]

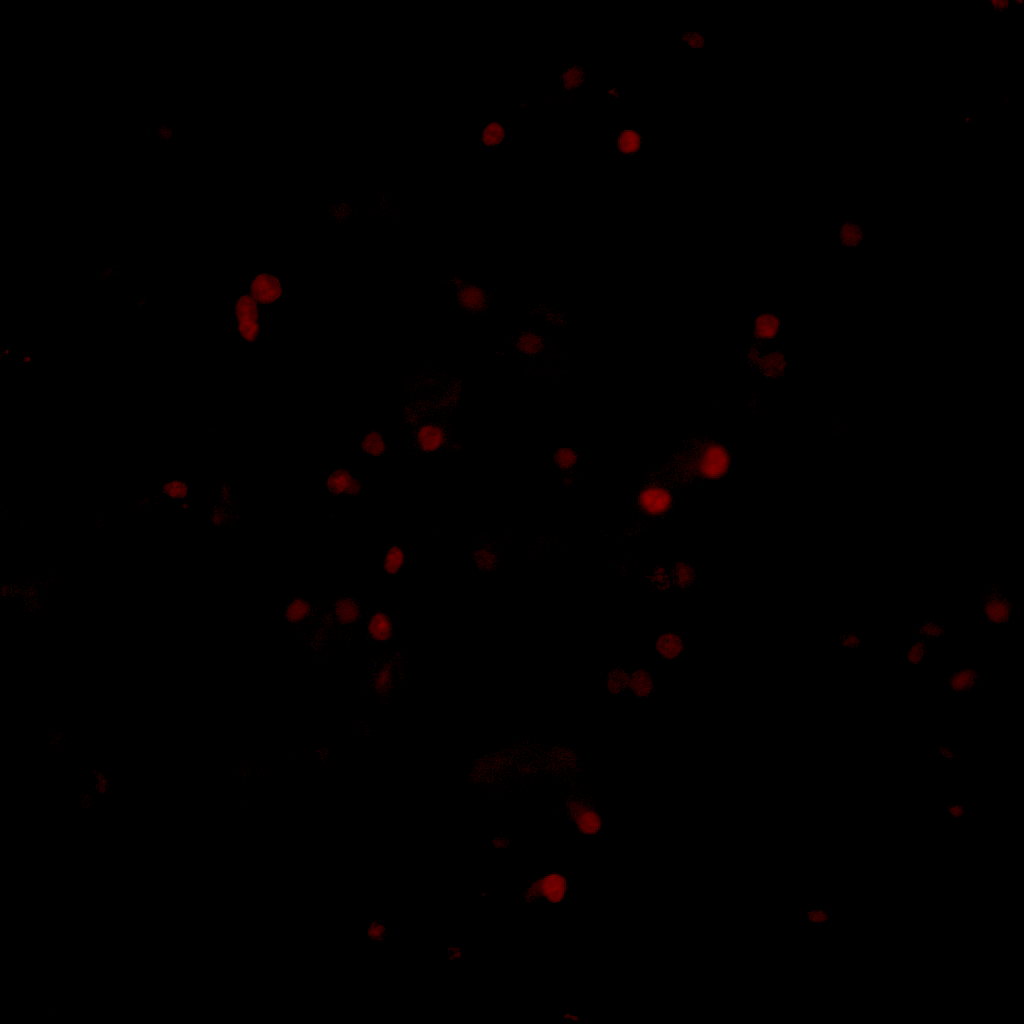

Supplement: Multimedia component 2 [file mmc2.zip › Supplemental_files/Figure 1/Figure 1G/HepG2/450_Edu.tif]

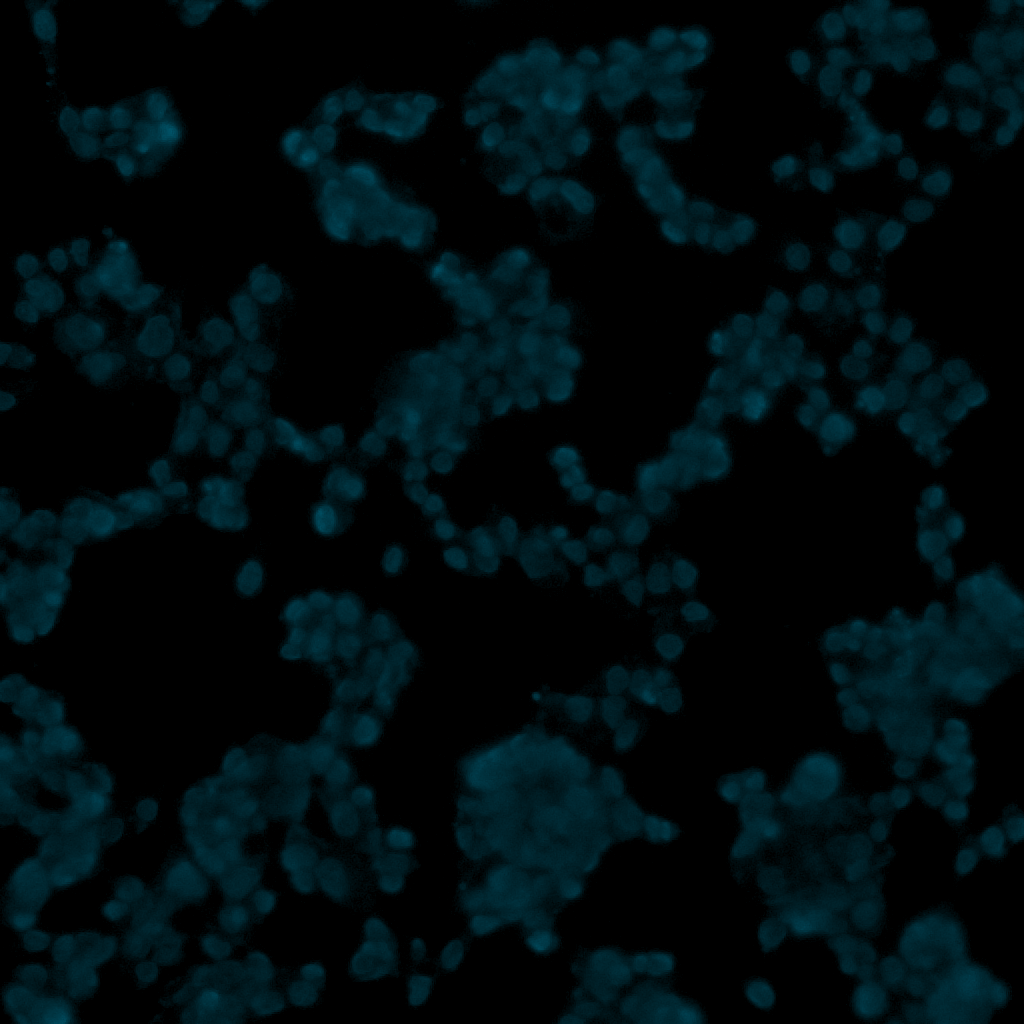

Supplement: Multimedia component 2 [file mmc2.zip › Supplemental_files/Figure 1/Figure 1G/HepG2/450_Hon.tif]

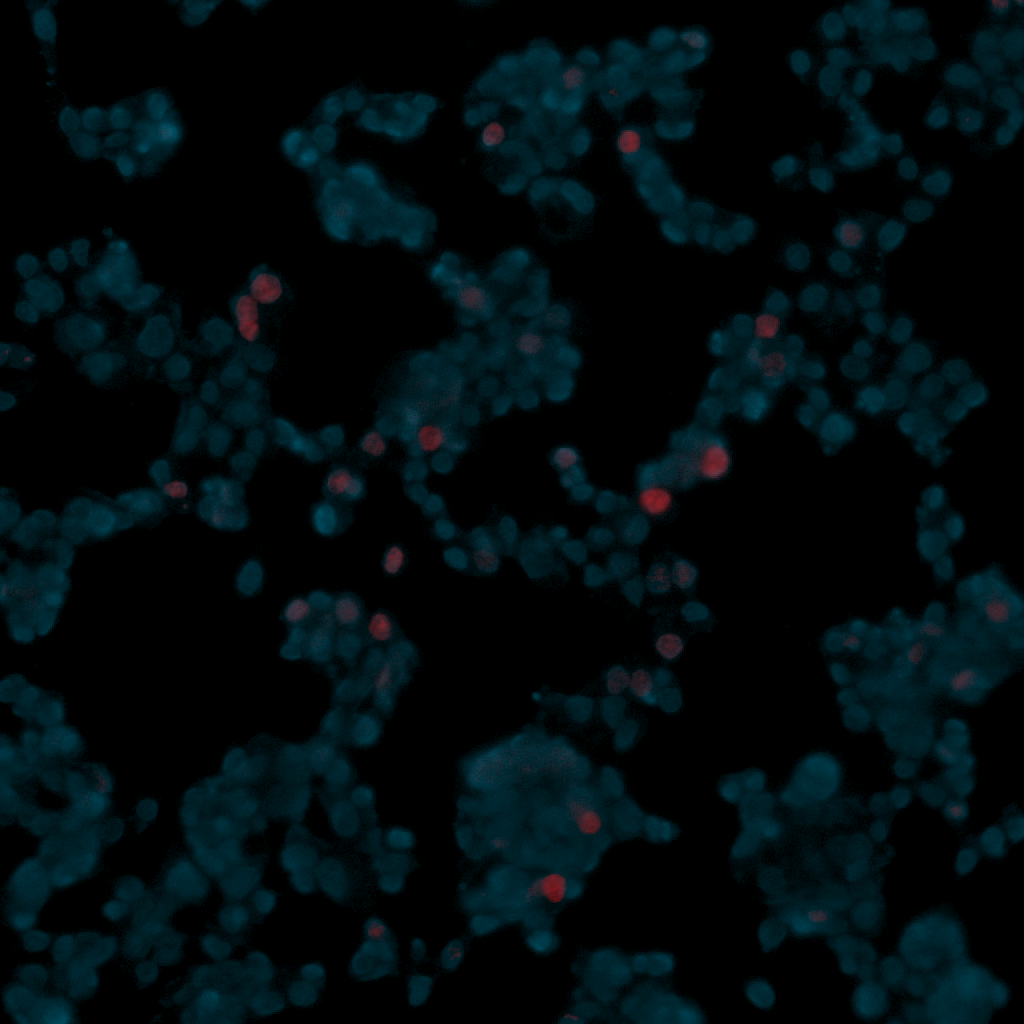

Supplement: Multimedia component 2 [file mmc2.zip › Supplemental_files/Figure 1/Figure 1G/HepG2/450_merge.tif]

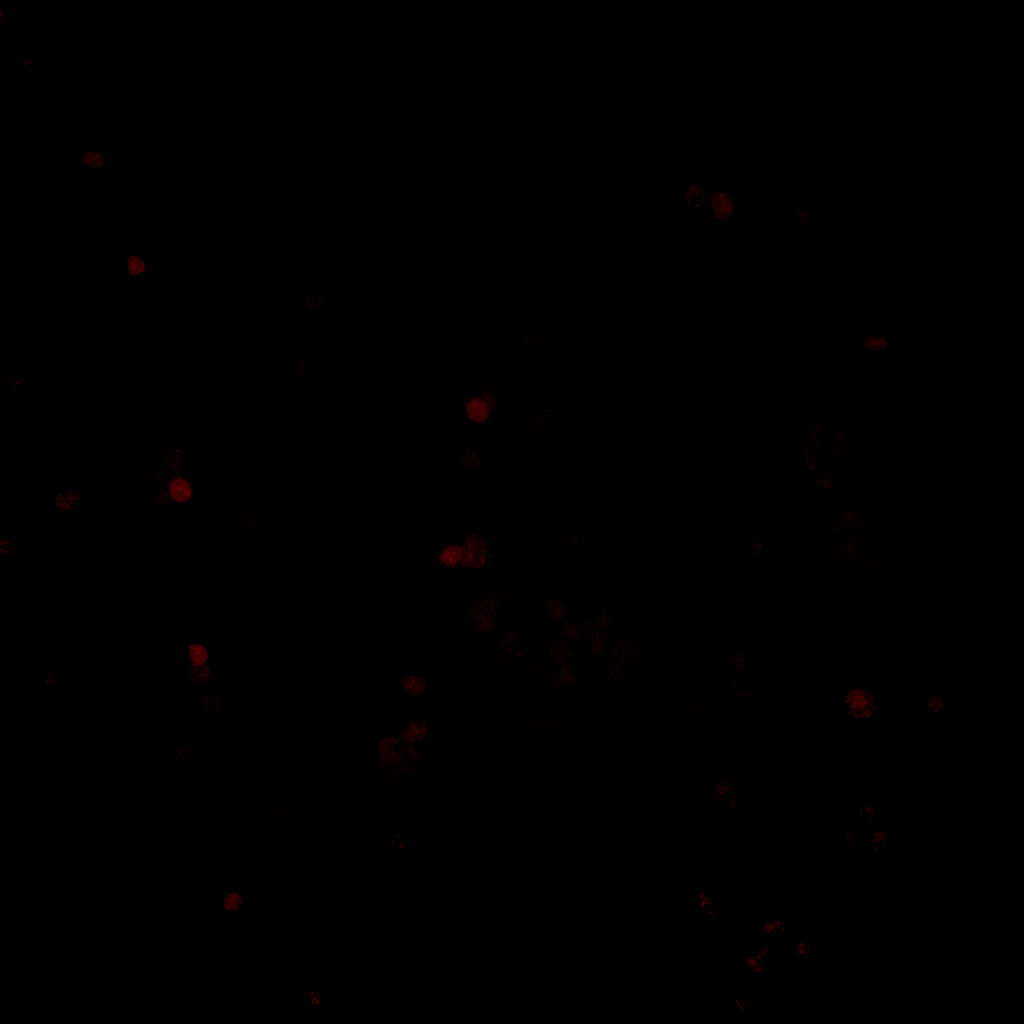

Supplement: Multimedia component 2 [file mmc2.zip › Supplemental_files/Figure 1/Figure 1G/HepG2/600_Edu.tif]

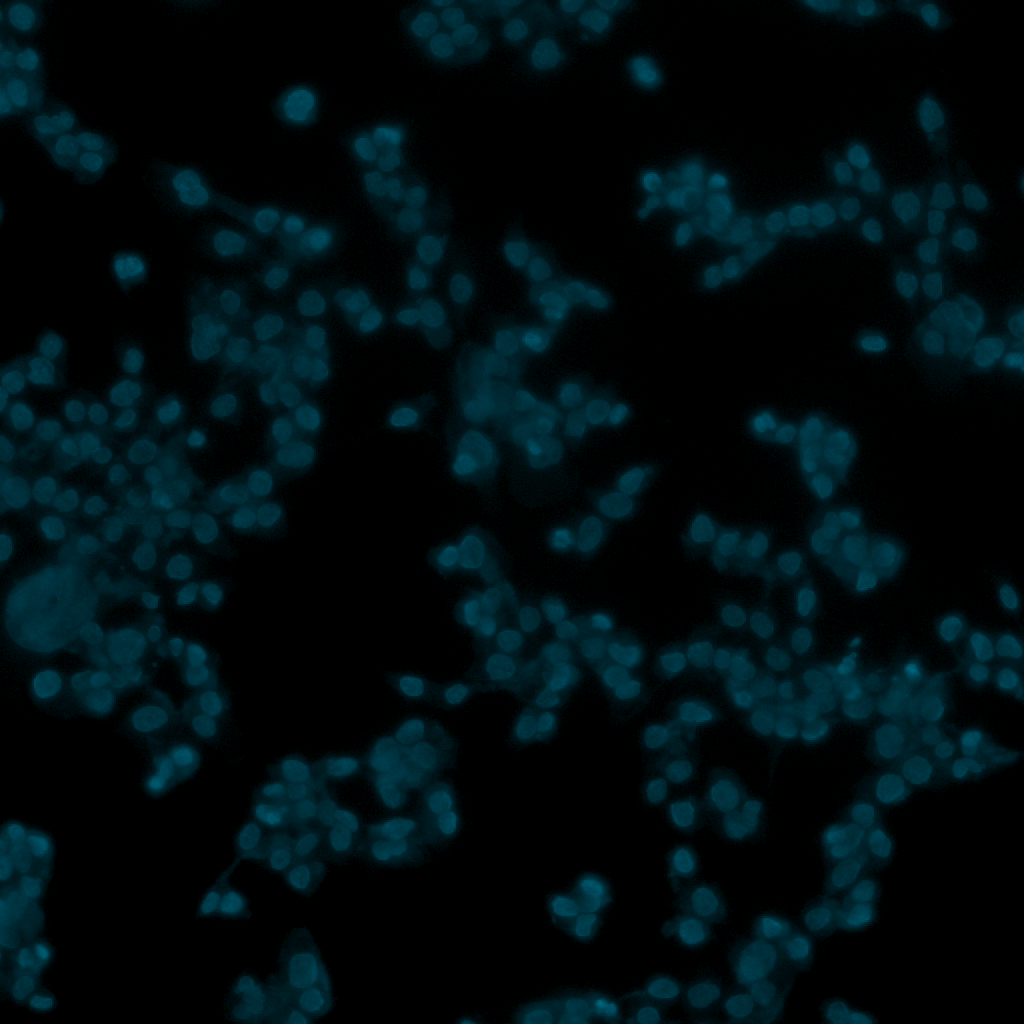

Supplement: Multimedia component 2 [file mmc2.zip › Supplemental_files/Figure 1/Figure 1G/HepG2/600_Hon.tif]

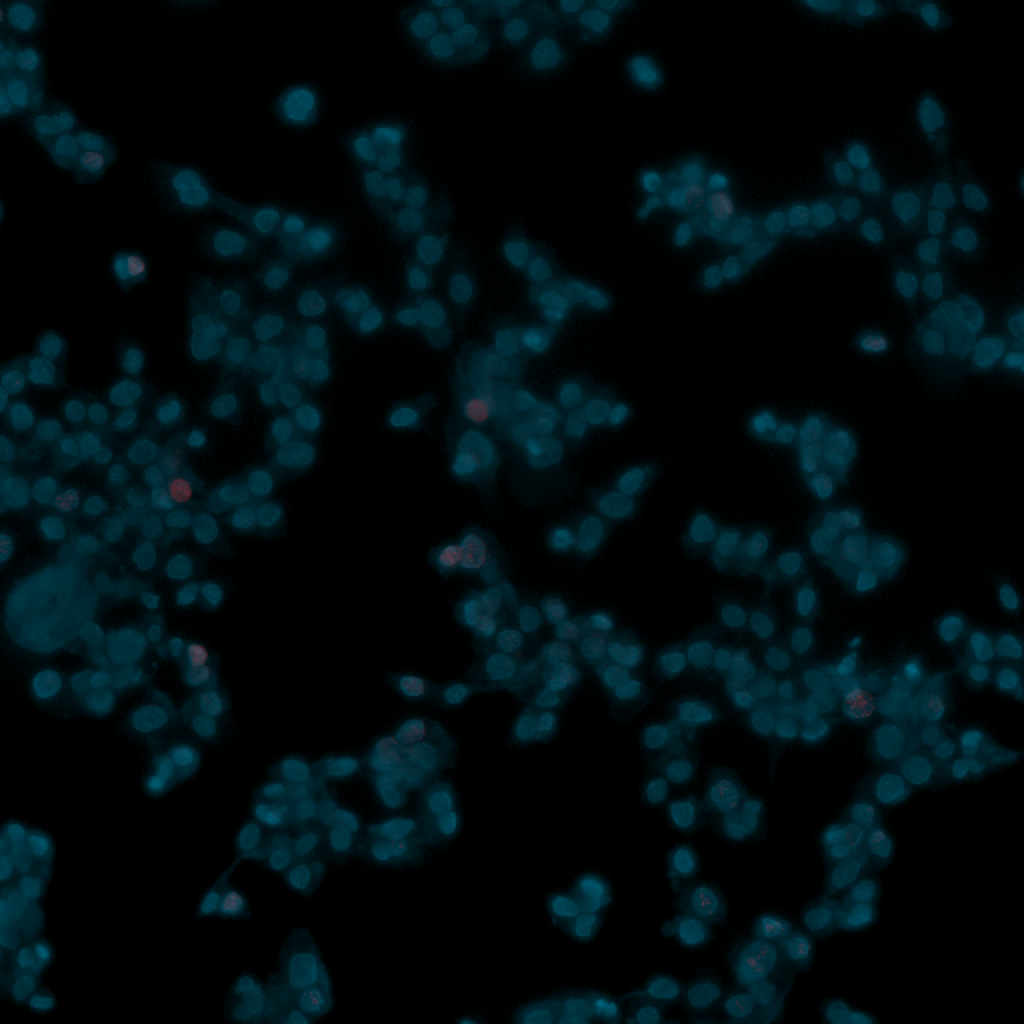

Supplement: Multimedia component 2 [file mmc2.zip › Supplemental_files/Figure 1/Figure 1G/HepG2/600_merge.tif]

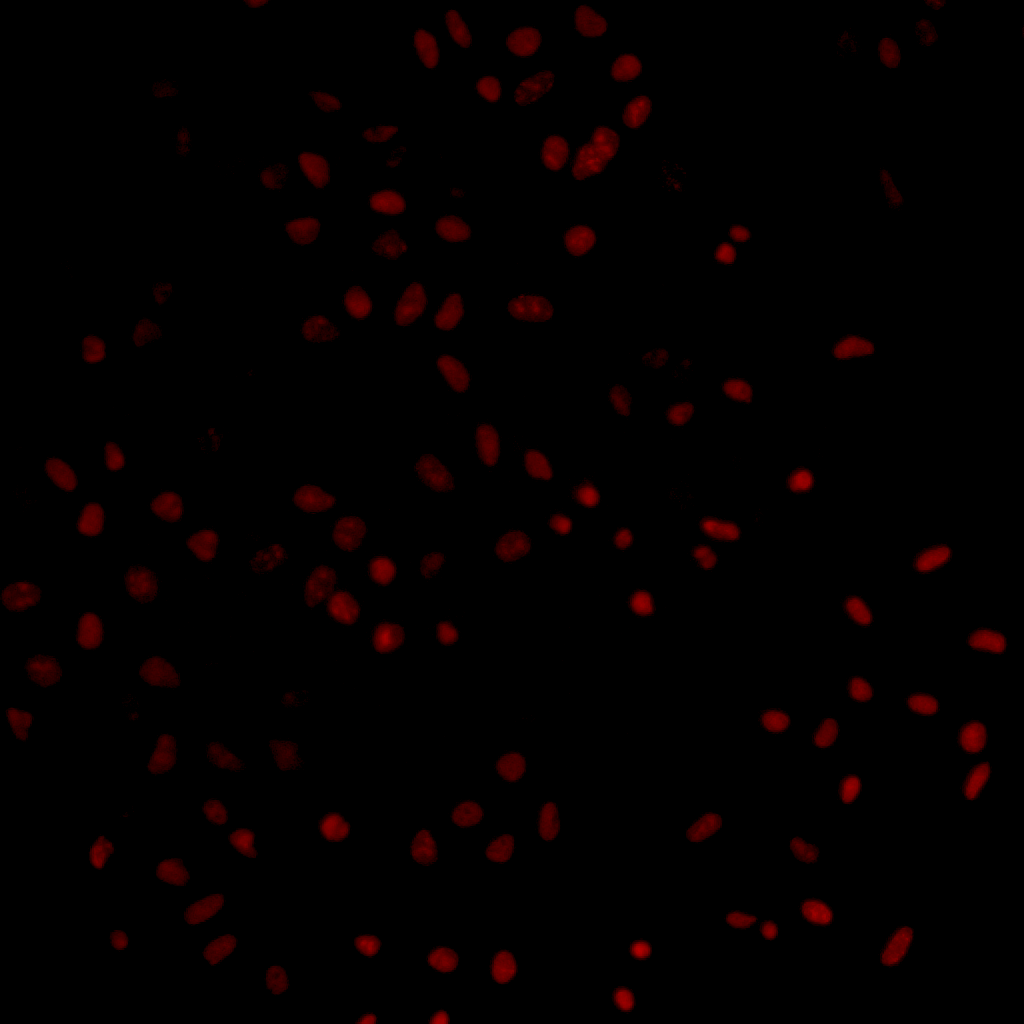

Supplement: Multimedia component 2 [file mmc2.zip › Supplemental_files/Figure 1/Figure 1G/LM3/0_Edu.tif]

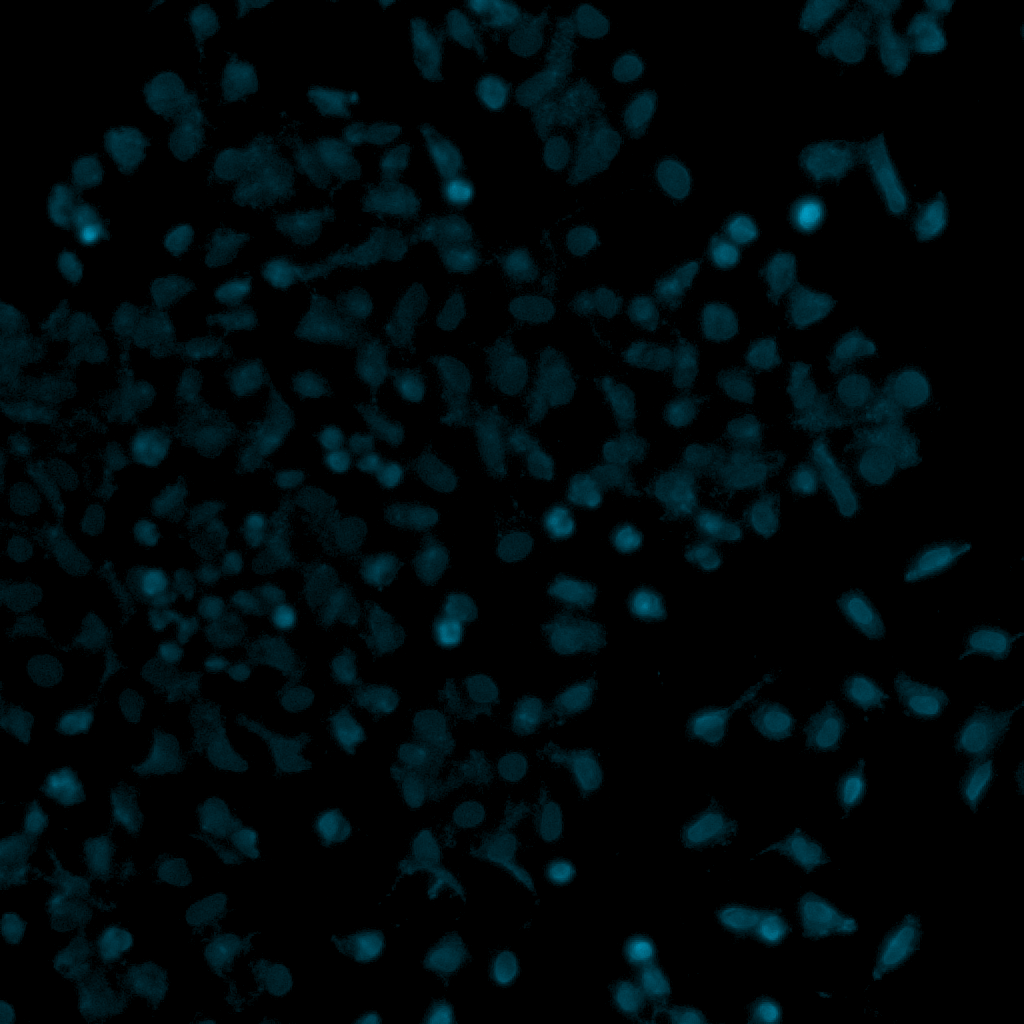

Supplement: Multimedia component 2 [file mmc2.zip › Supplemental_files/Figure 1/Figure 1G/LM3/0_Hon.tif]

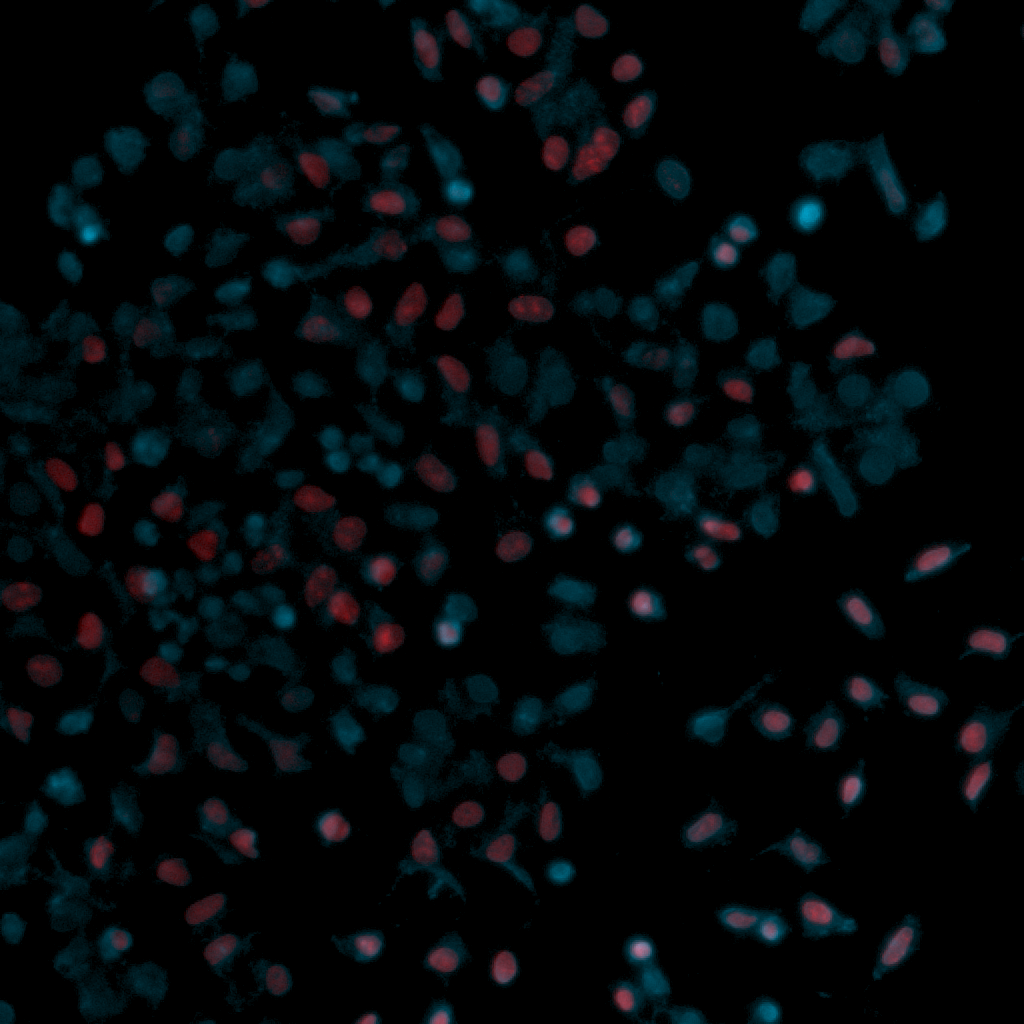

Supplement: Multimedia component 2 [file mmc2.zip › Supplemental_files/Figure 1/Figure 1G/LM3/0_merge.tif]

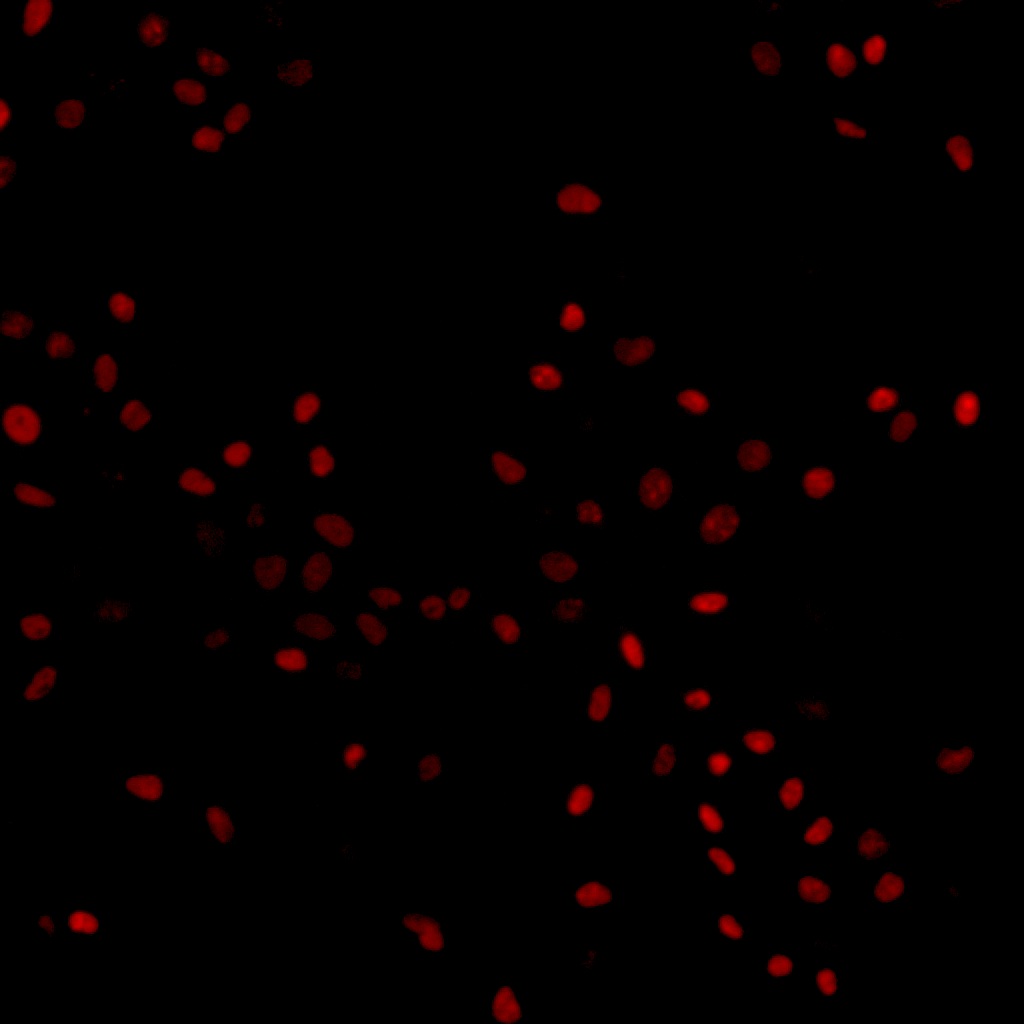

Supplement: Multimedia component 2 [file mmc2.zip › Supplemental_files/Figure 1/Figure 1G/LM3/200_Edu.tif]

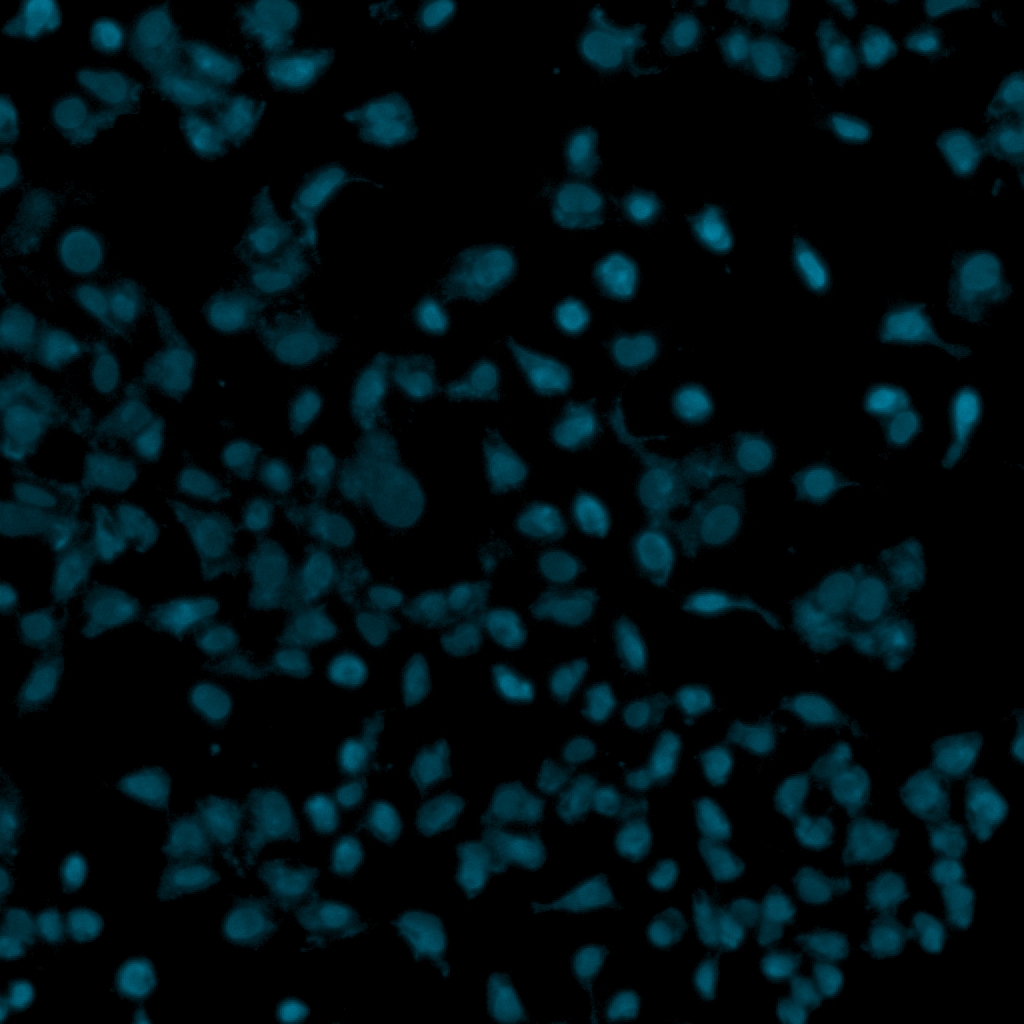

Supplement: Multimedia component 2 [file mmc2.zip › Supplemental_files/Figure 1/Figure 1G/LM3/200_Hon.tif]

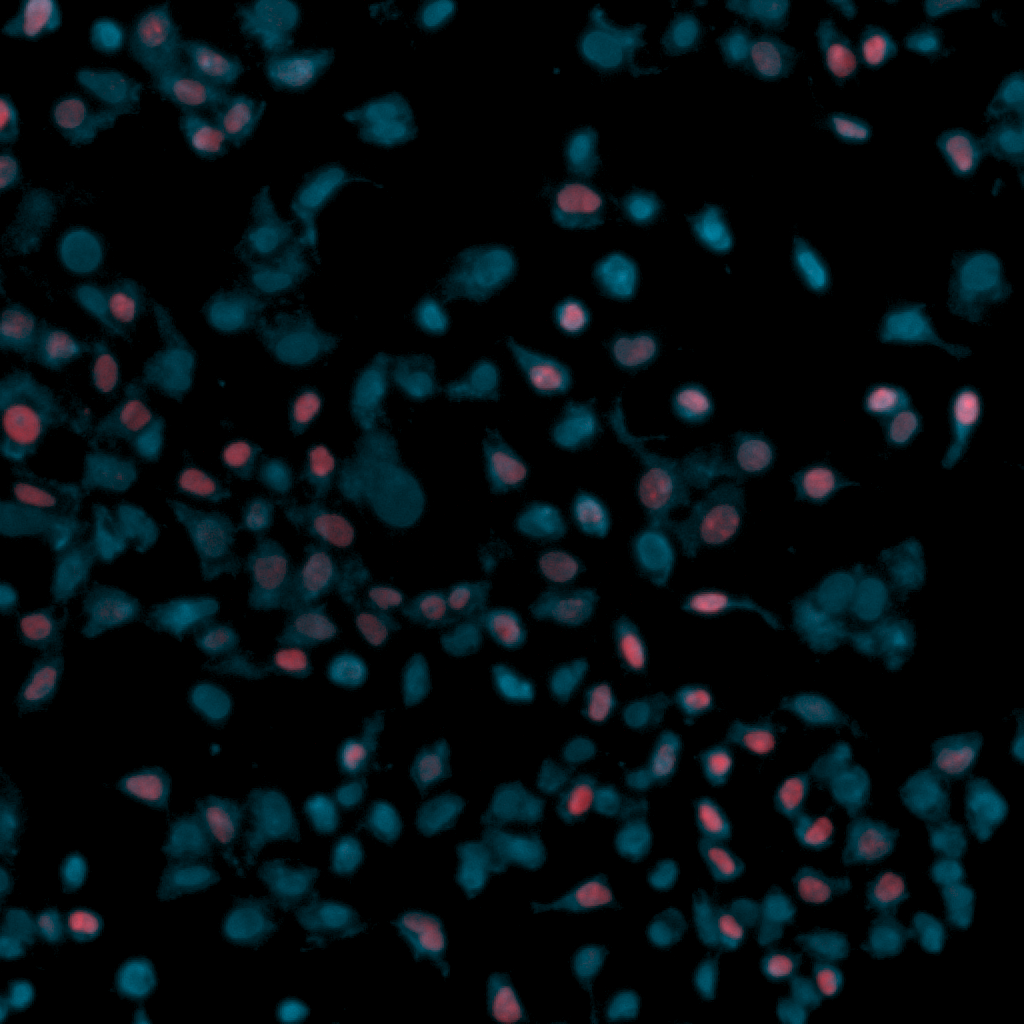

Supplement: Multimedia component 2 [file mmc2.zip › Supplemental_files/Figure 1/Figure 1G/LM3/200_merge.tif]

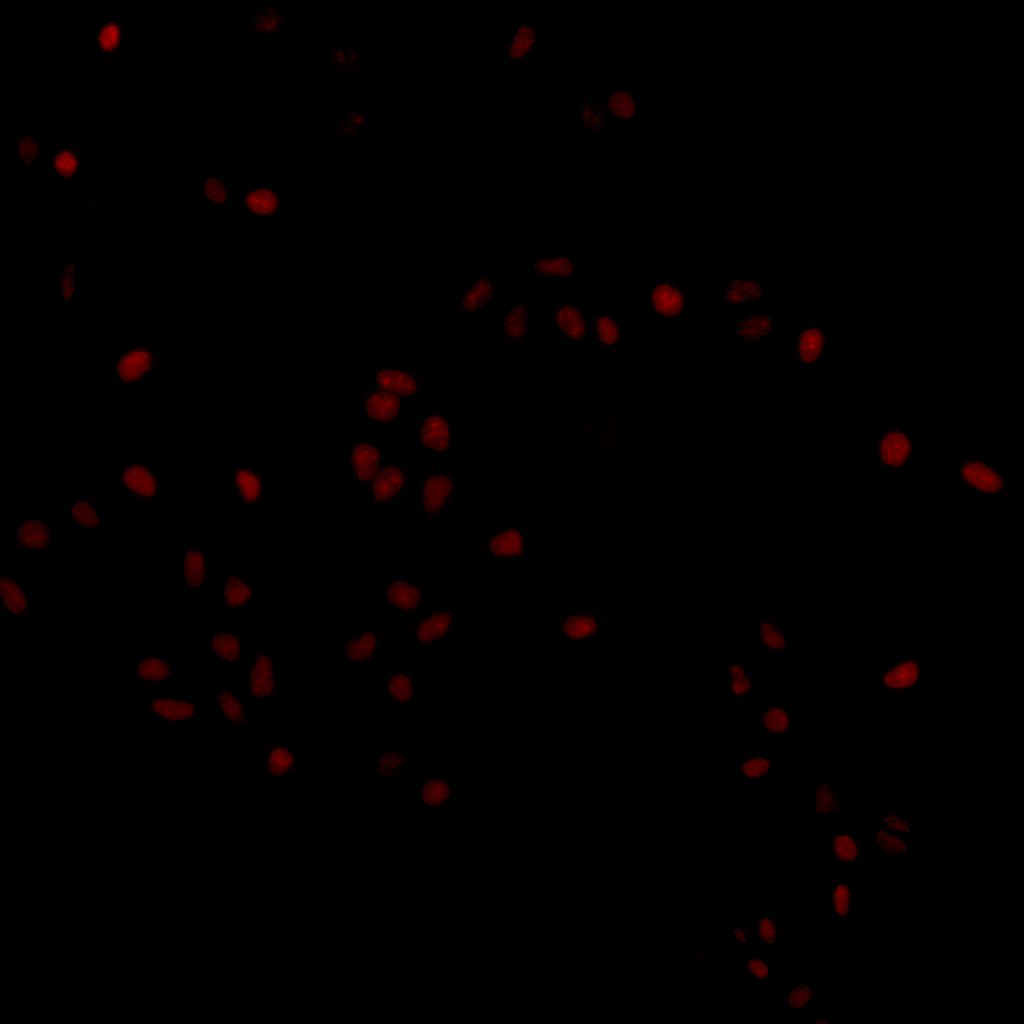

Supplement: Multimedia component 2 [file mmc2.zip › Supplemental_files/Figure 1/Figure 1G/LM3/450_Edu.tif]

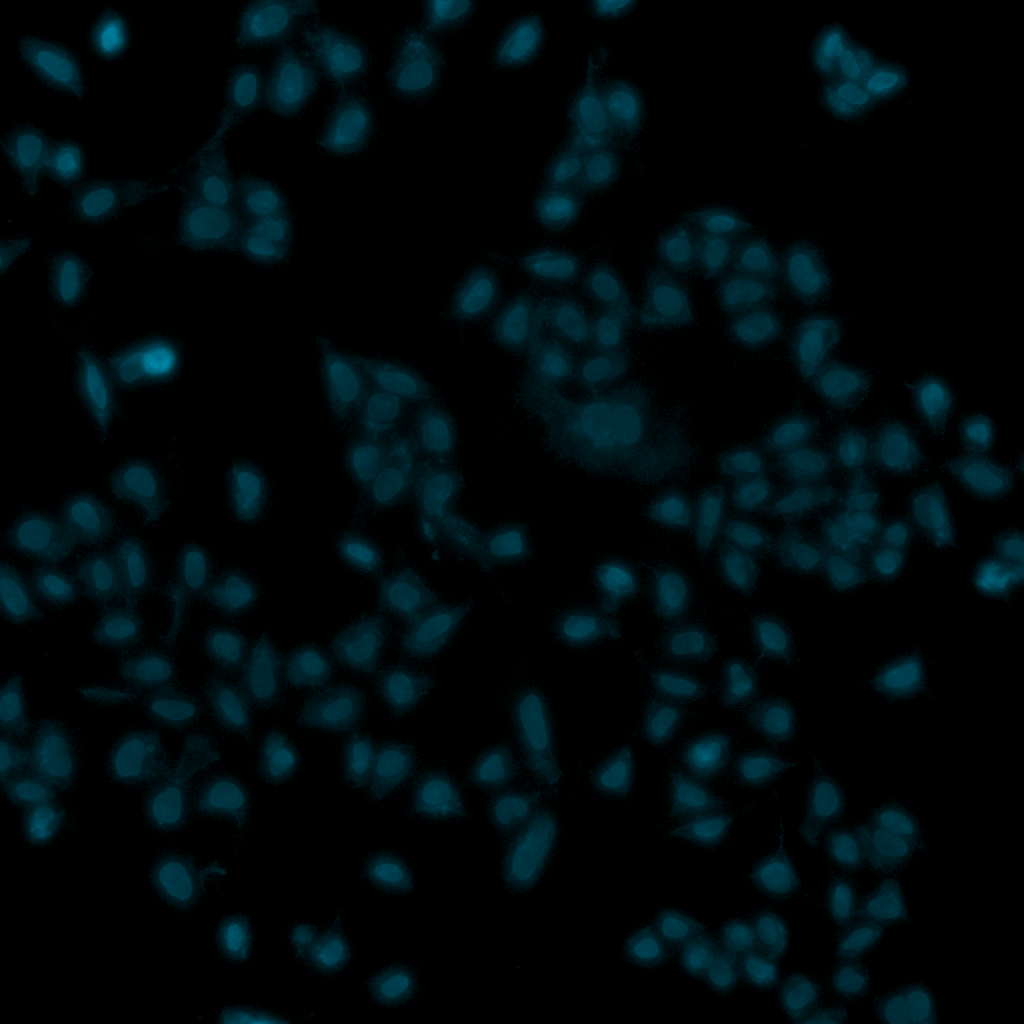

Supplement: Multimedia component 2 [file mmc2.zip › Supplemental_files/Figure 1/Figure 1G/LM3/450_Hon.tif]

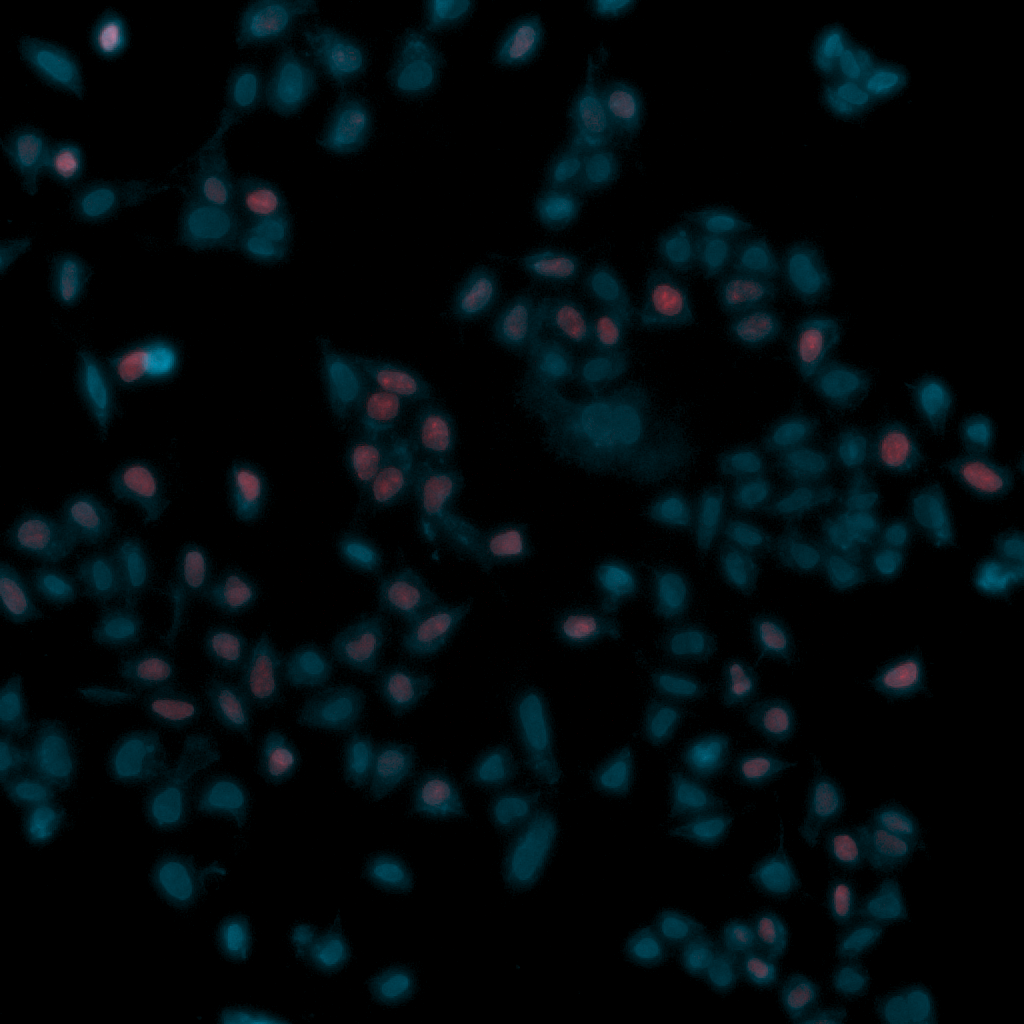

Supplement: Multimedia component 2 [file mmc2.zip › Supplemental_files/Figure 1/Figure 1G/LM3/450_merge.tif]

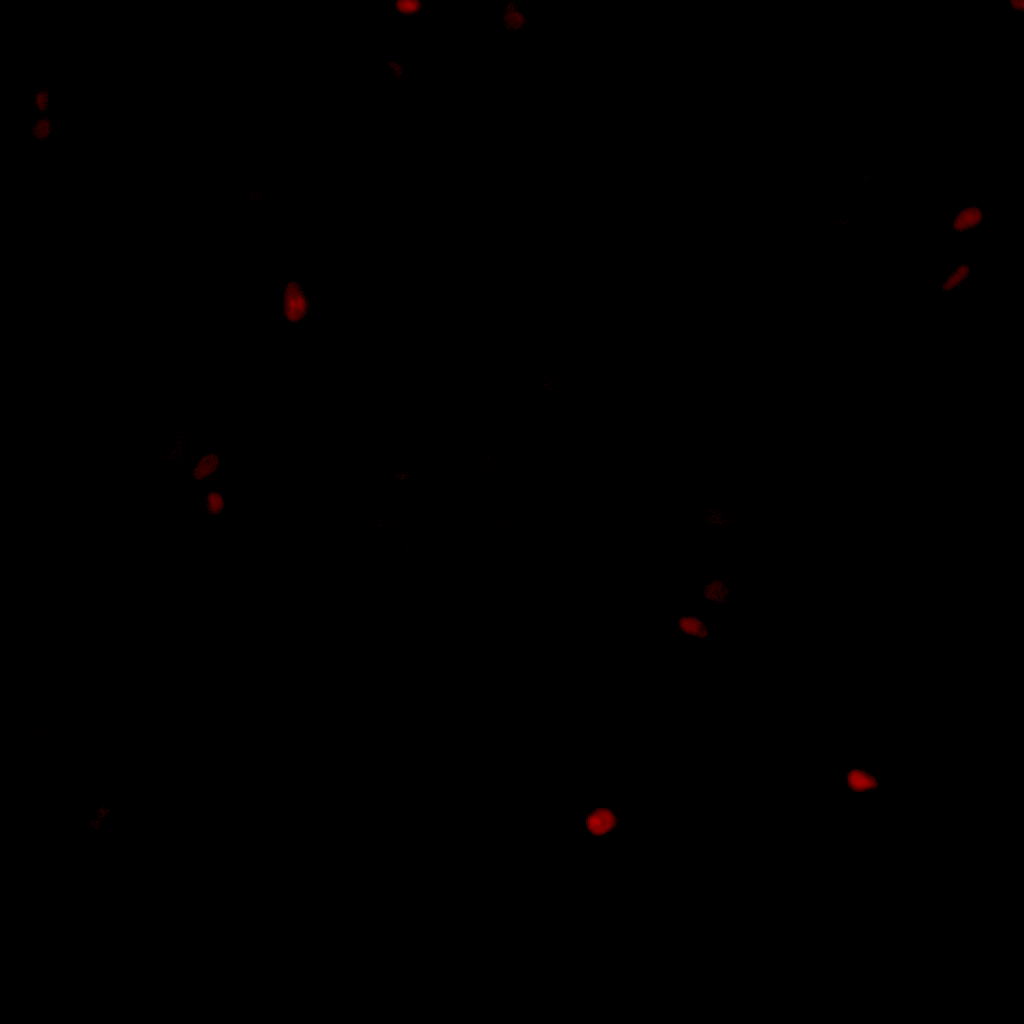

Supplement: Multimedia component 2 [file mmc2.zip › Supplemental_files/Figure 1/Figure 1G/LM3/600_Edu.tif]

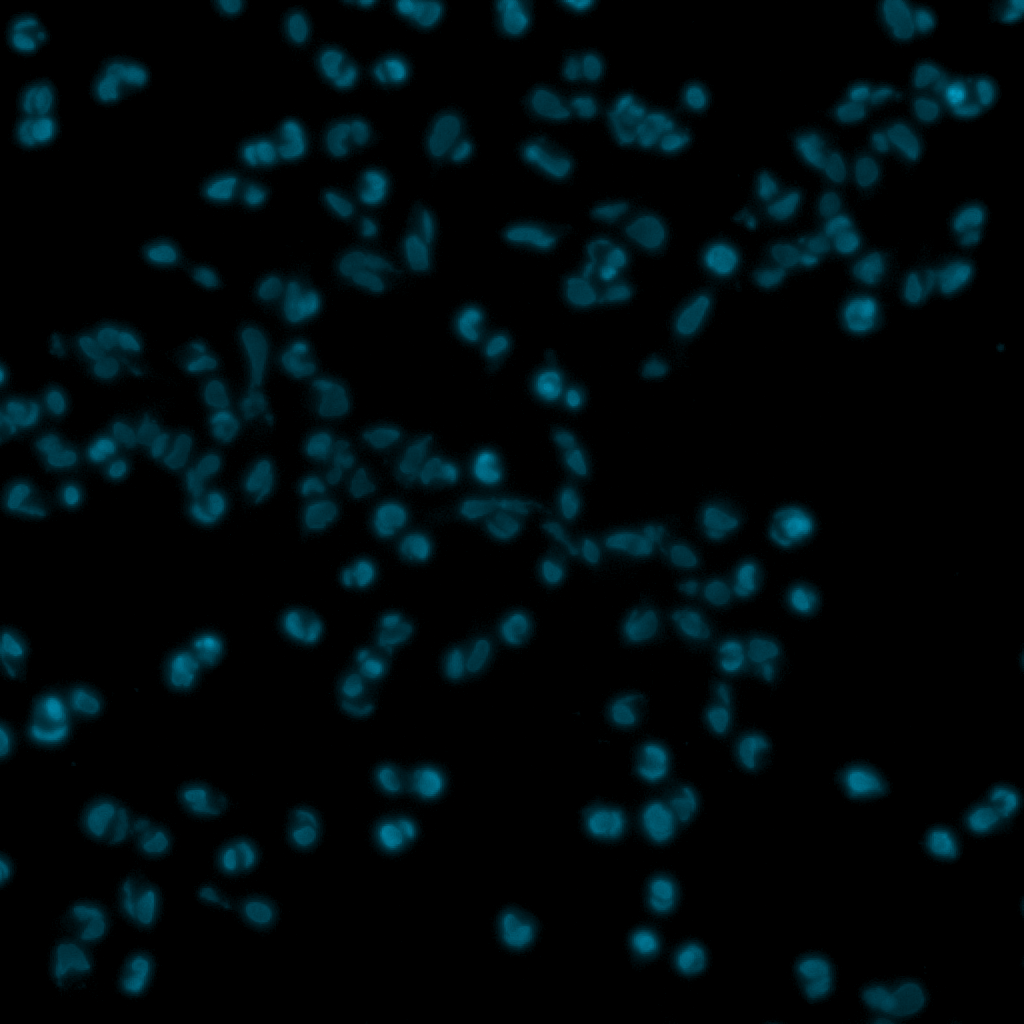

Supplement: Multimedia component 2 [file mmc2.zip › Supplemental_files/Figure 1/Figure 1G/LM3/600_Hon.tif]

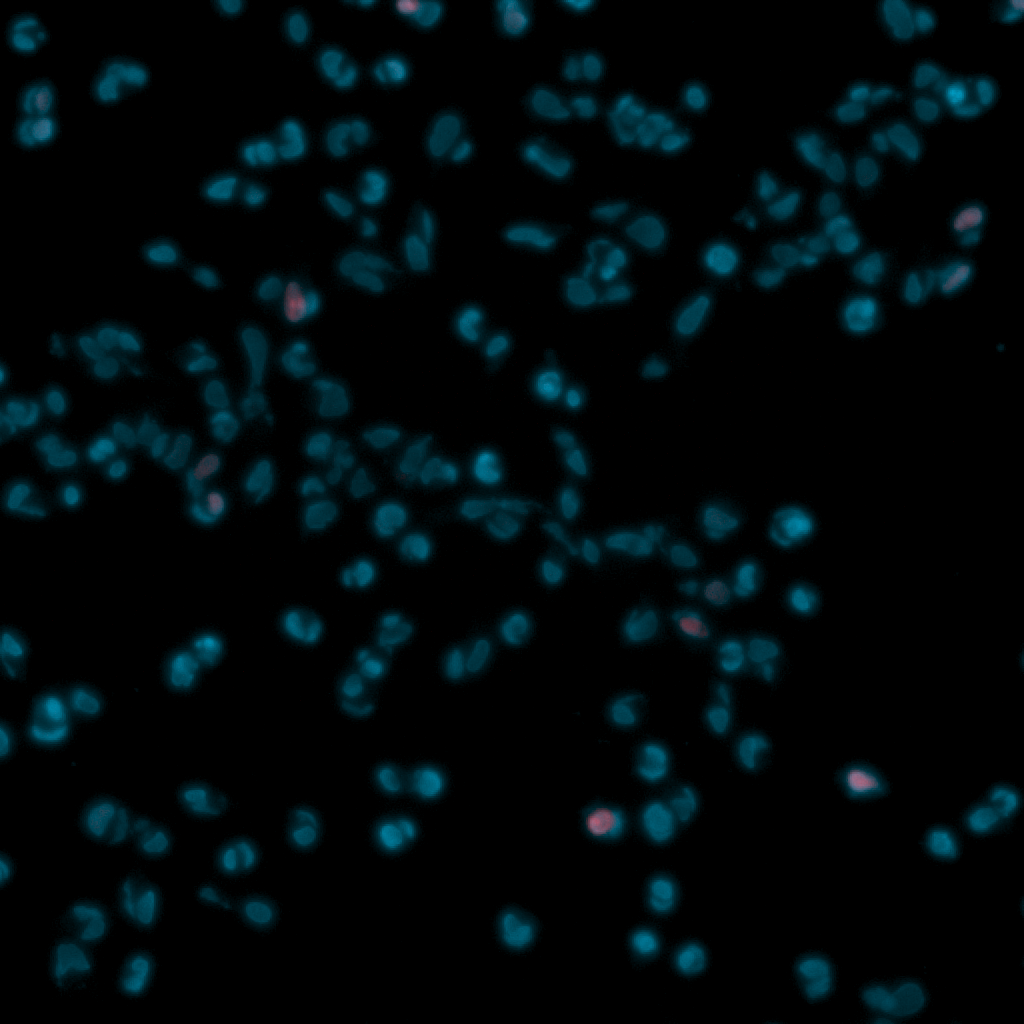

Supplement: Multimedia component 2 [file mmc2.zip › Supplemental_files/Figure 1/Figure 1G/LM3/600_merge.tif]

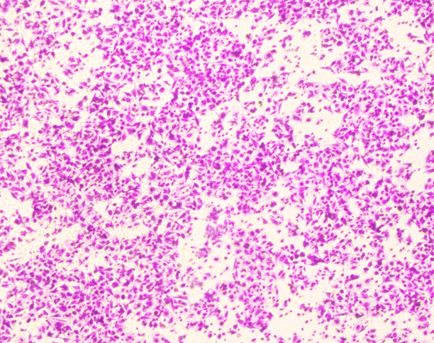

Supplement: Multimedia component 2 [file mmc2.zip › Supplemental_files/Figure 2/Figure 2A/HepG2/0.tif]

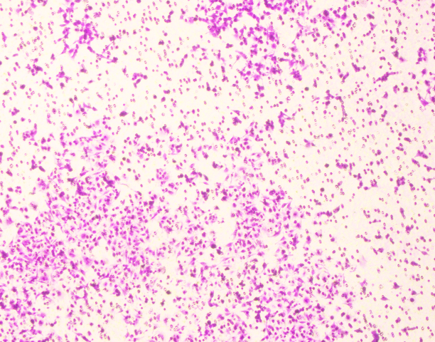

Supplement: Multimedia component 2 [file mmc2.zip › Supplemental_files/Figure 2/Figure 2A/HepG2/200.tif]

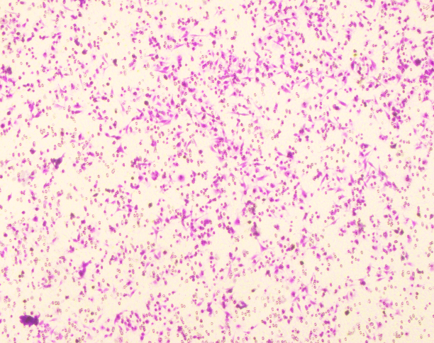

Supplement: Multimedia component 2 [file mmc2.zip › Supplemental_files/Figure 2/Figure 2A/HepG2/450.tif]

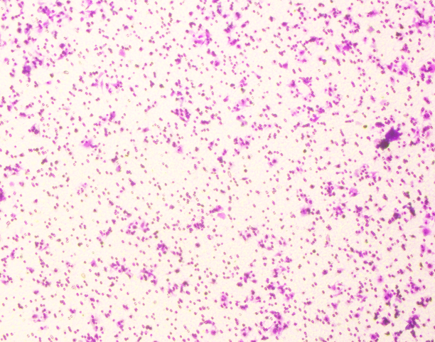

Supplement: Multimedia component 2 [file mmc2.zip › Supplemental_files/Figure 2/Figure 2A/HepG2/600.tif]

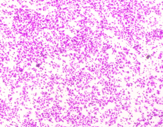

Supplement: Multimedia component 2 [file mmc2.zip › Supplemental_files/Figure 2/Figure 2A/LM3/0.tif]

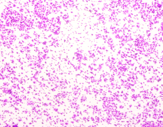

Supplement: Multimedia component 2 [file mmc2.zip › Supplemental_files/Figure 2/Figure 2A/LM3/200.tif]

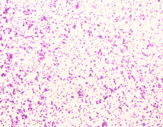

Supplement: Multimedia component 2 [file mmc2.zip › Supplemental_files/Figure 2/Figure 2A/LM3/450.tif]

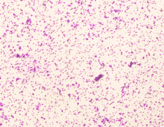

Supplement: Multimedia component 2 [file mmc2.zip › Supplemental_files/Figure 2/Figure 2A/LM3/600.tif]

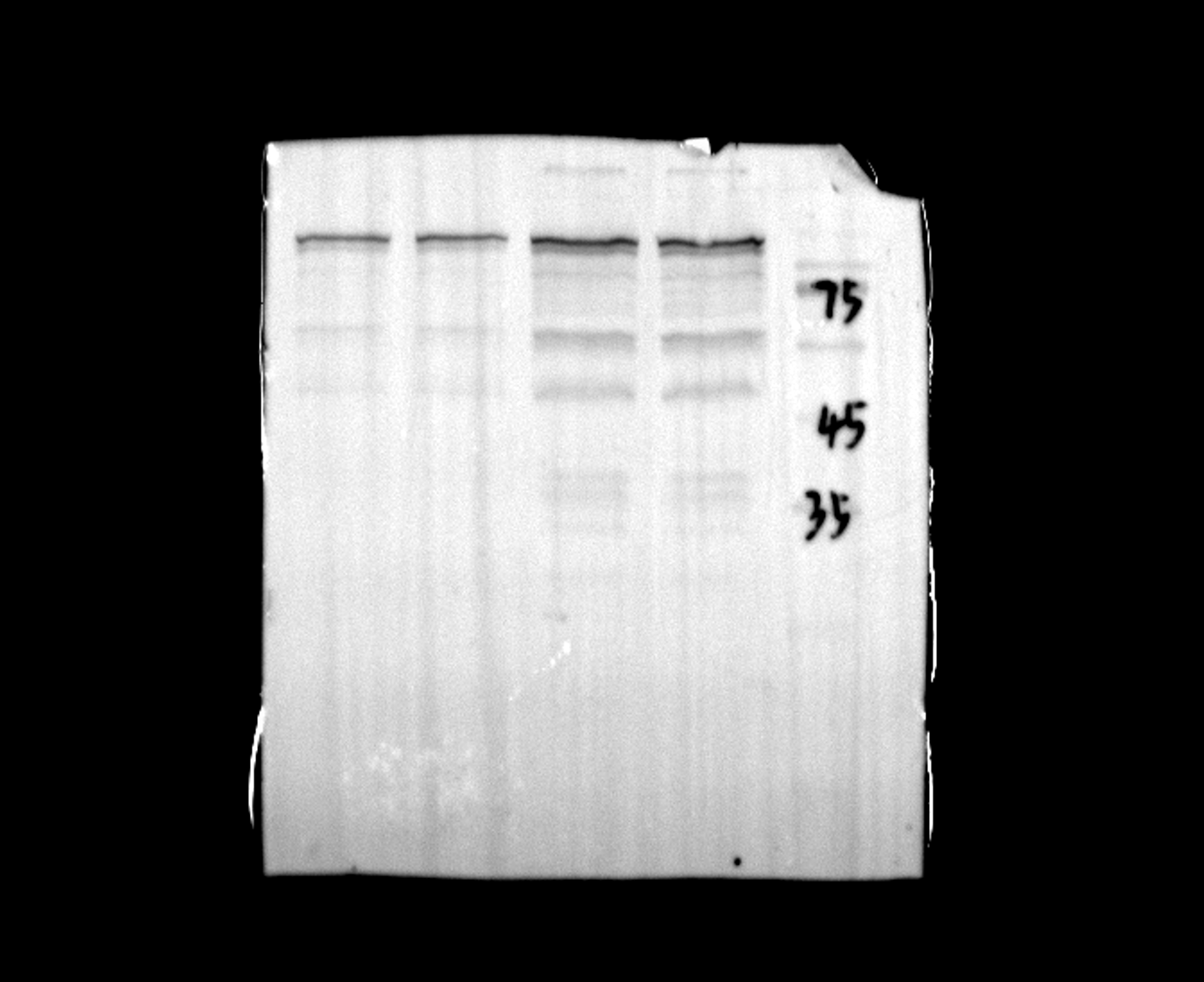

Supplement: Multimedia component 2 [file mmc2.zip › Supplemental_files/Figure 2/Figure 2D/HepG2/E-Cad.Tif]

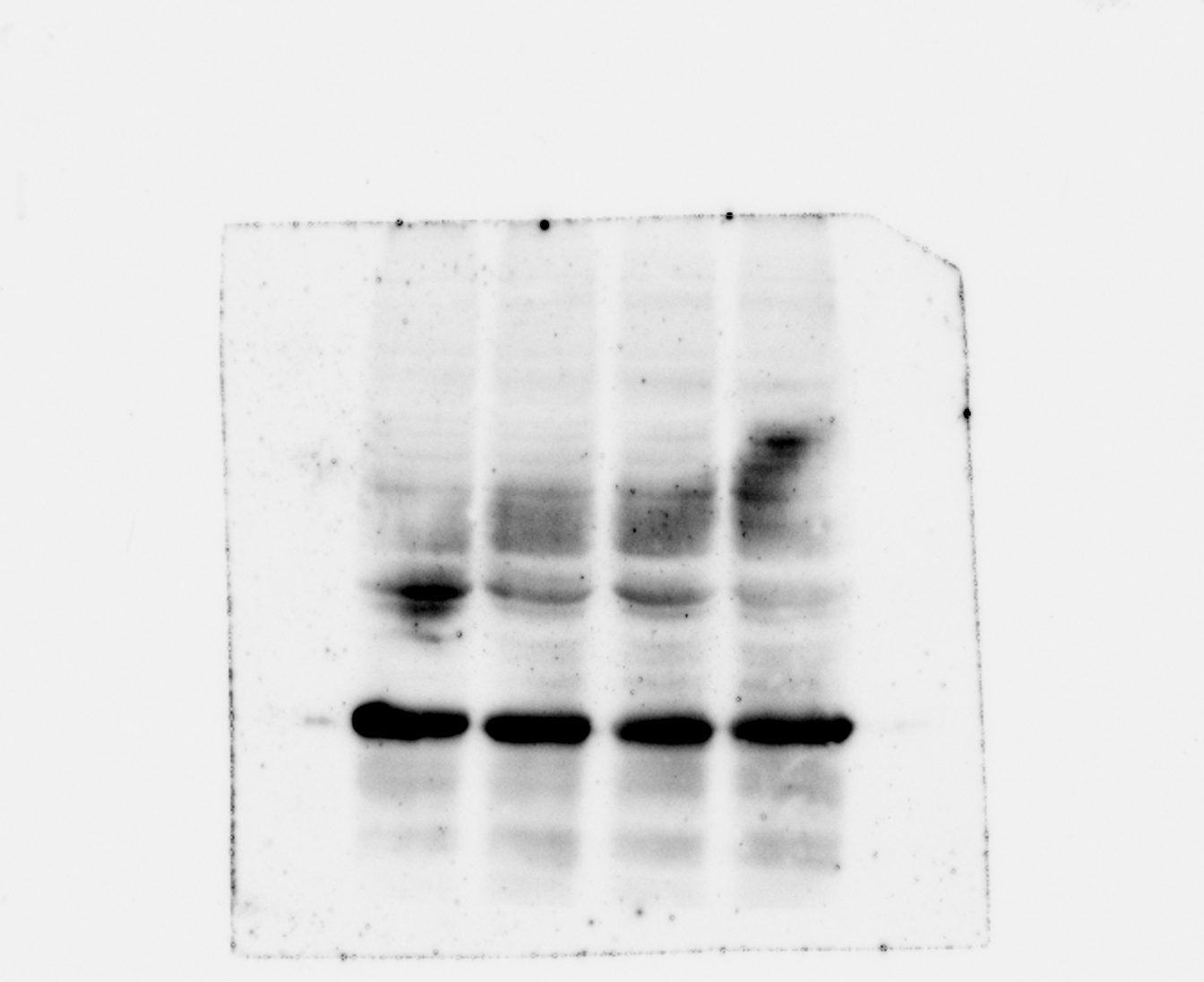

Supplement: Multimedia component 2 [file mmc2.zip › Supplemental_files/Figure 2/Figure 2D/HepG2/GAPDH.Tif]

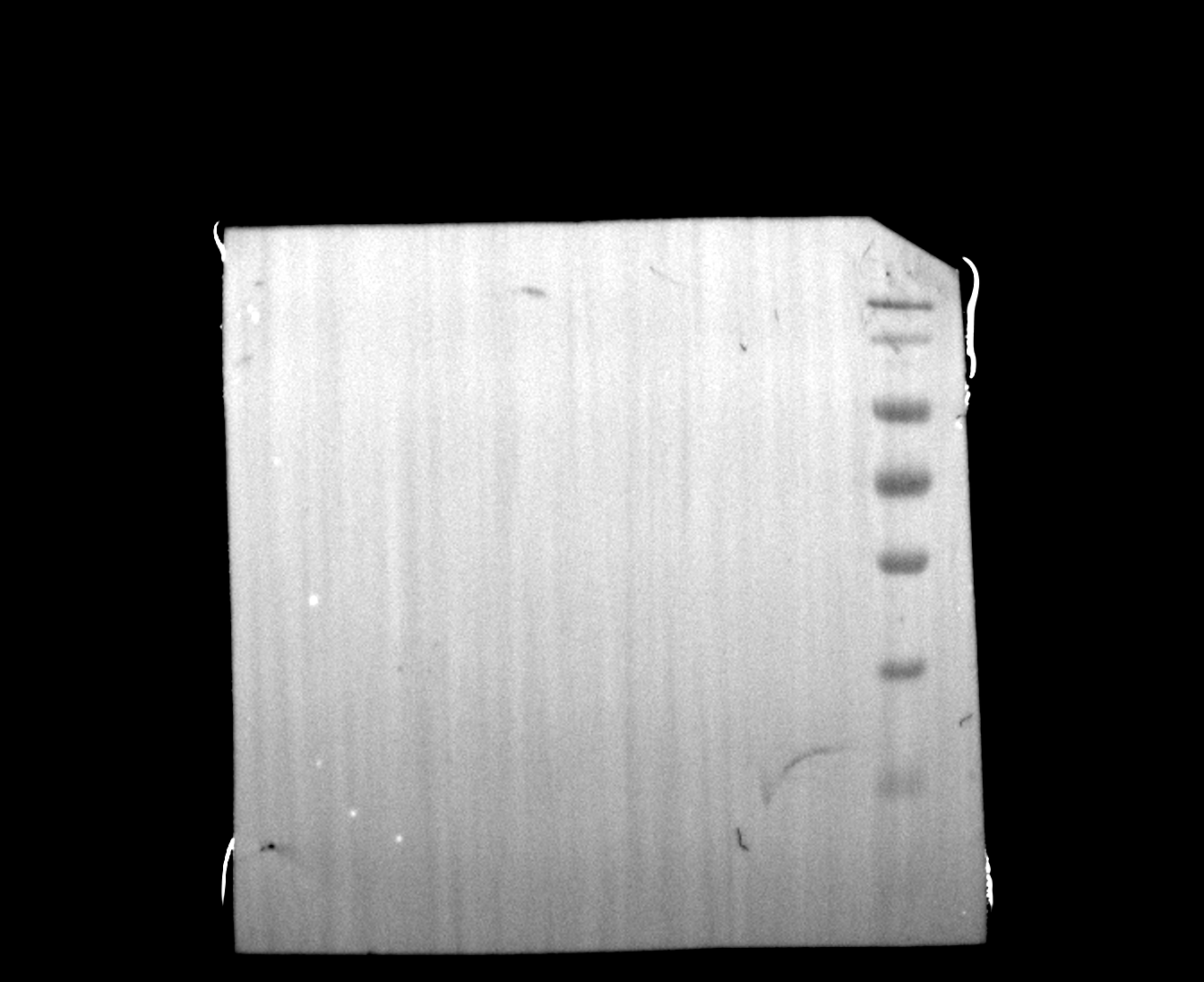

Supplement: Multimedia component 2 [file mmc2.zip › Supplemental_files/Figure 2/Figure 2D/HepG2/GAPDH_marker.Tif]

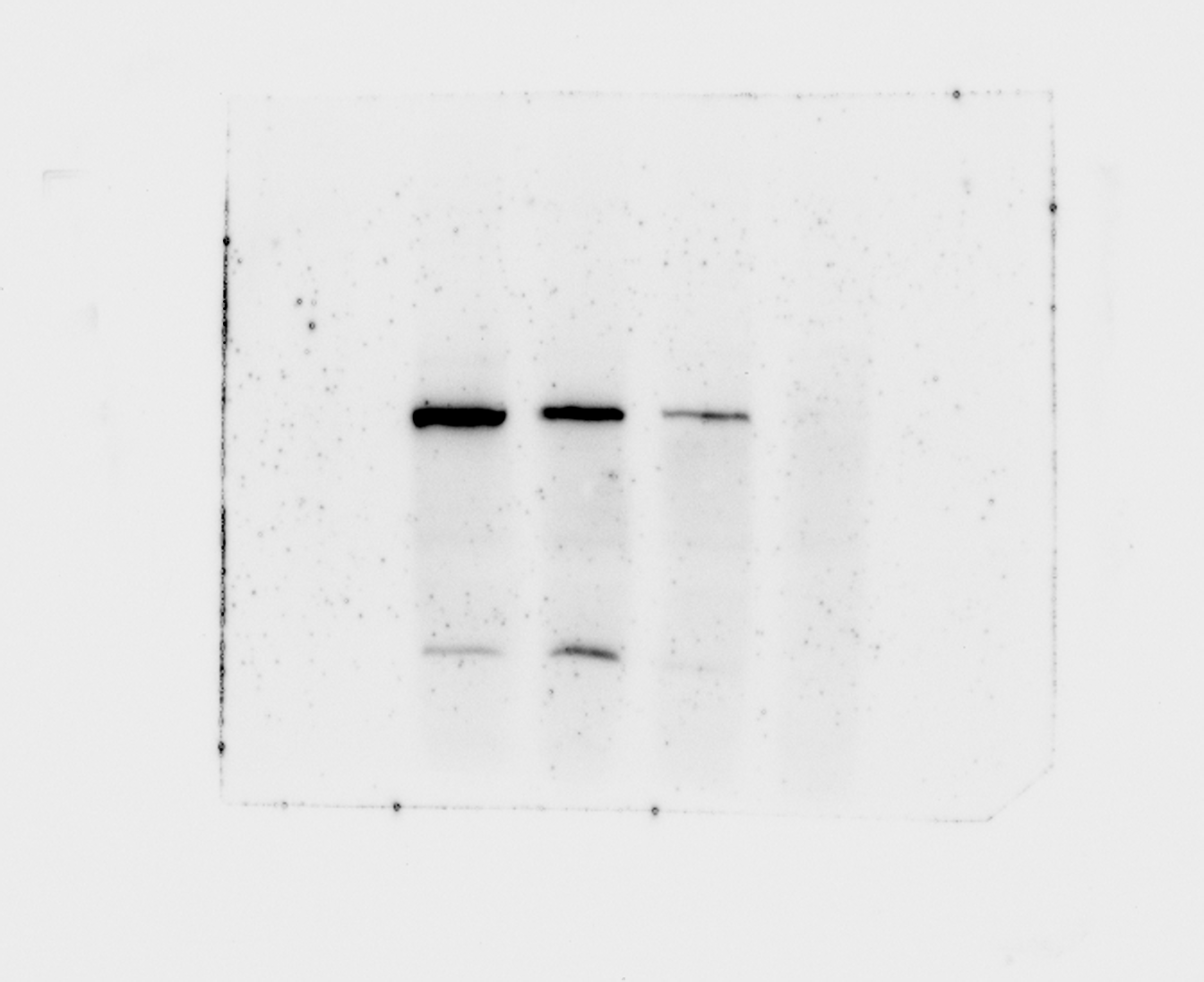

Supplement: Multimedia component 2 [file mmc2.zip › Supplemental_files/Figure 2/Figure 2D/HepG2/N-Cad.Tif]

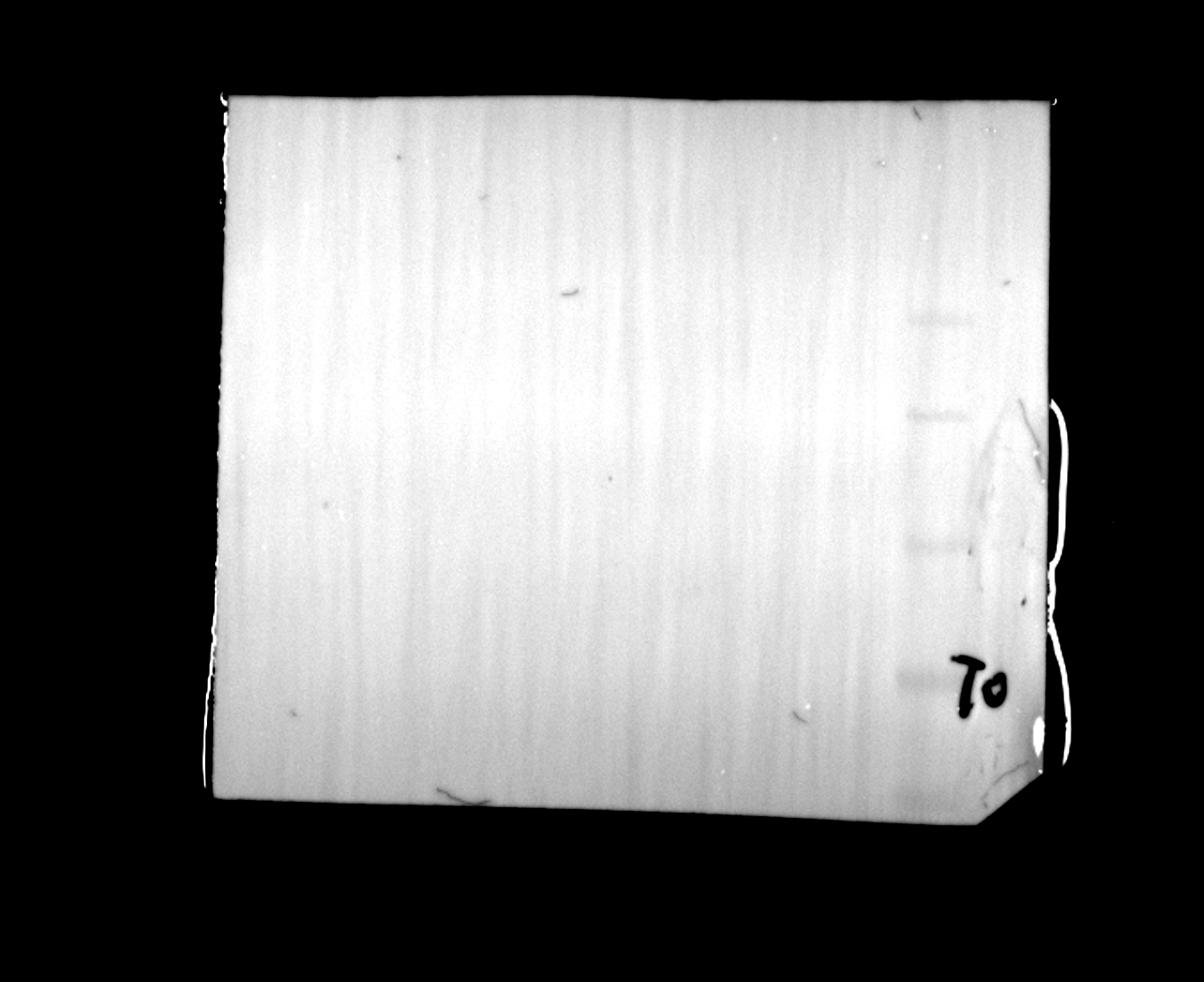

Supplement: Multimedia component 2 [file mmc2.zip › Supplemental_files/Figure 2/Figure 2D/HepG2/N-Cad_marker.Tif]

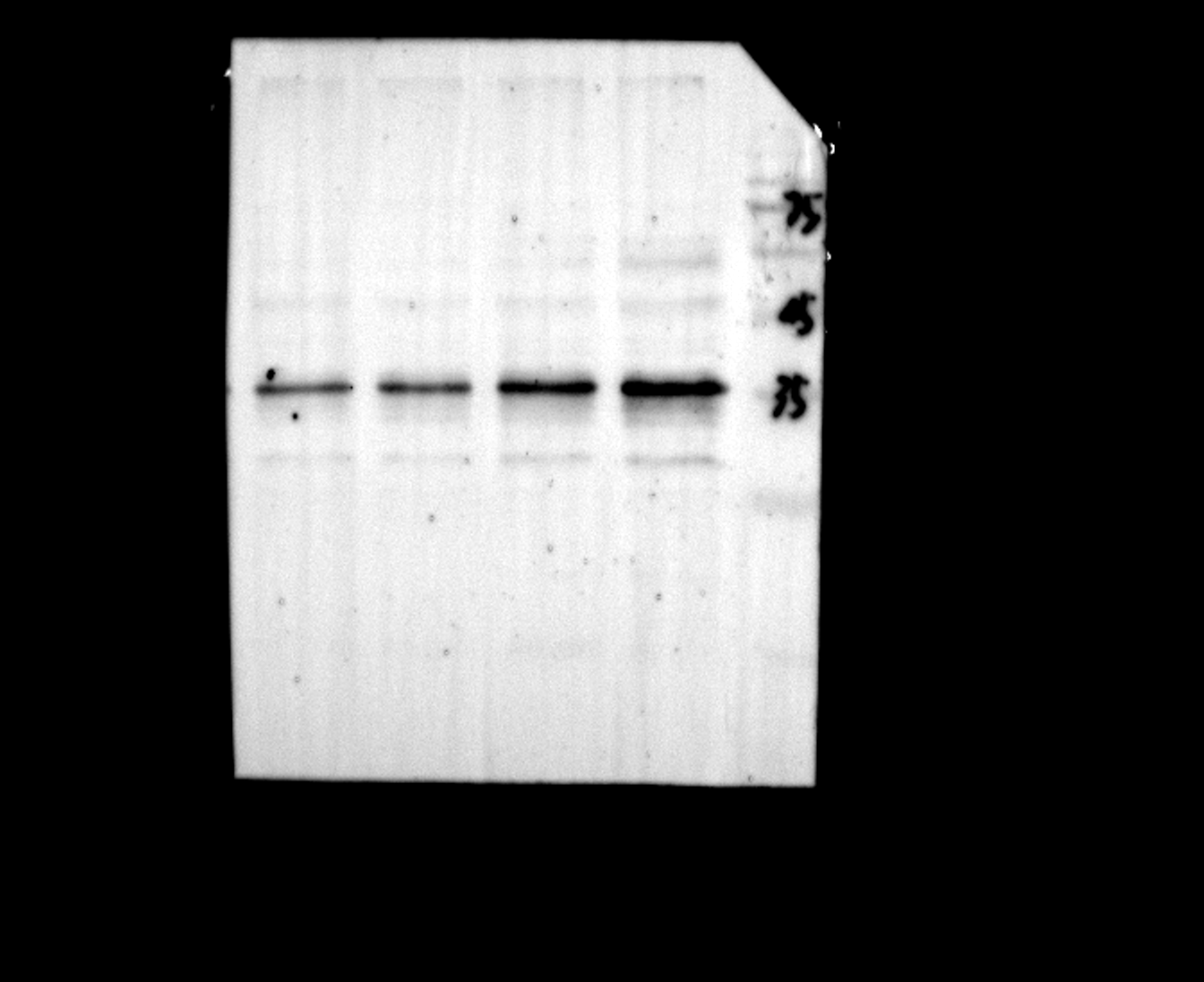

Supplement: Multimedia component 2 [file mmc2.zip › Supplemental_files/Figure 2/Figure 2D/HepG2/SNAI2.Tif]

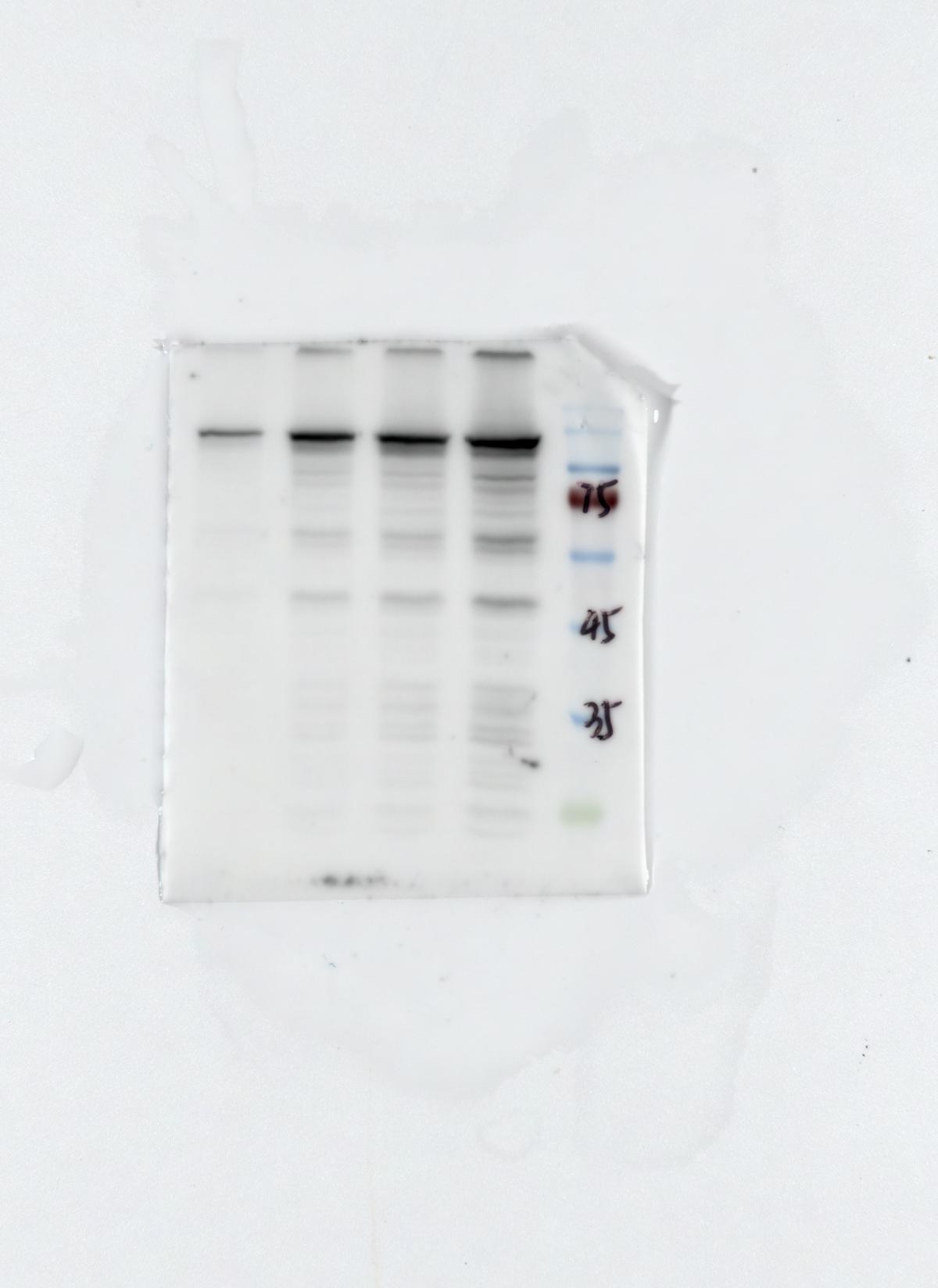

Supplement: Multimedia component 2 [file mmc2.zip › Supplemental_files/Figure 2/Figure 2D/LM3/E-CA.jpg]

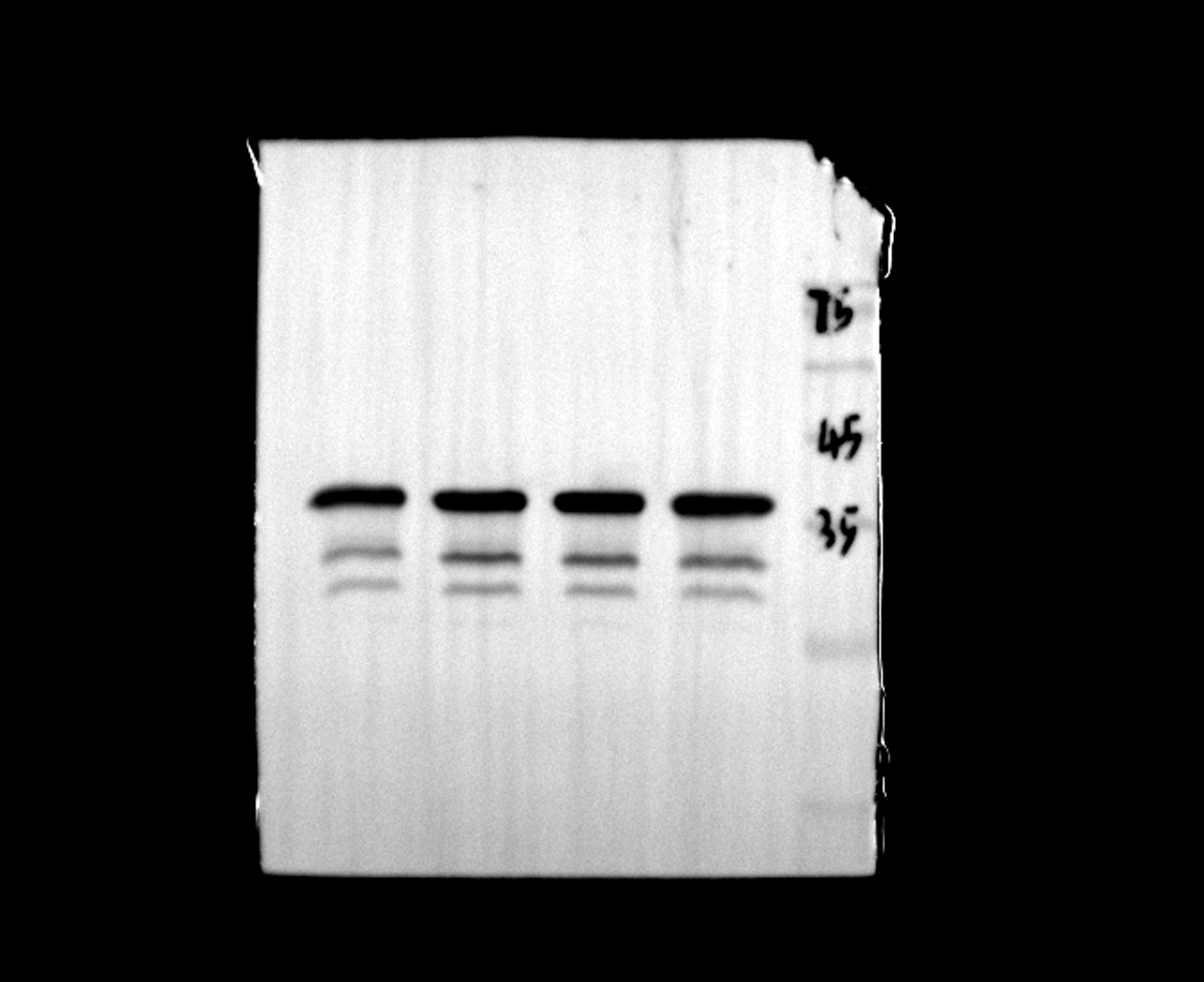

Supplement: Multimedia component 2 [file mmc2.zip › Supplemental_files/Figure 2/Figure 2D/LM3/GAPDH.Tif]

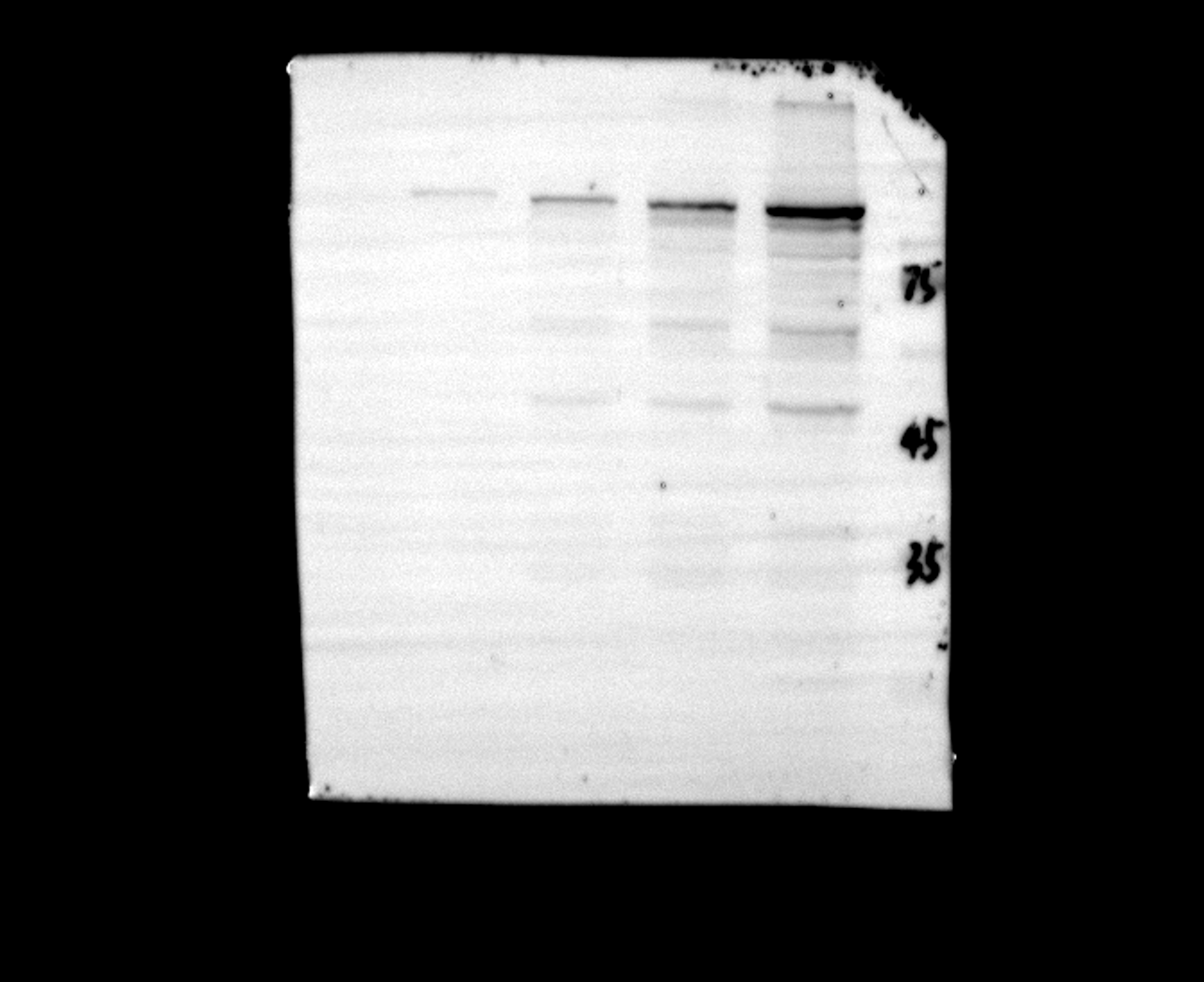

Supplement: Multimedia component 2 [file mmc2.zip › Supplemental_files/Figure 2/Figure 2D/LM3/N-CA.Tif]

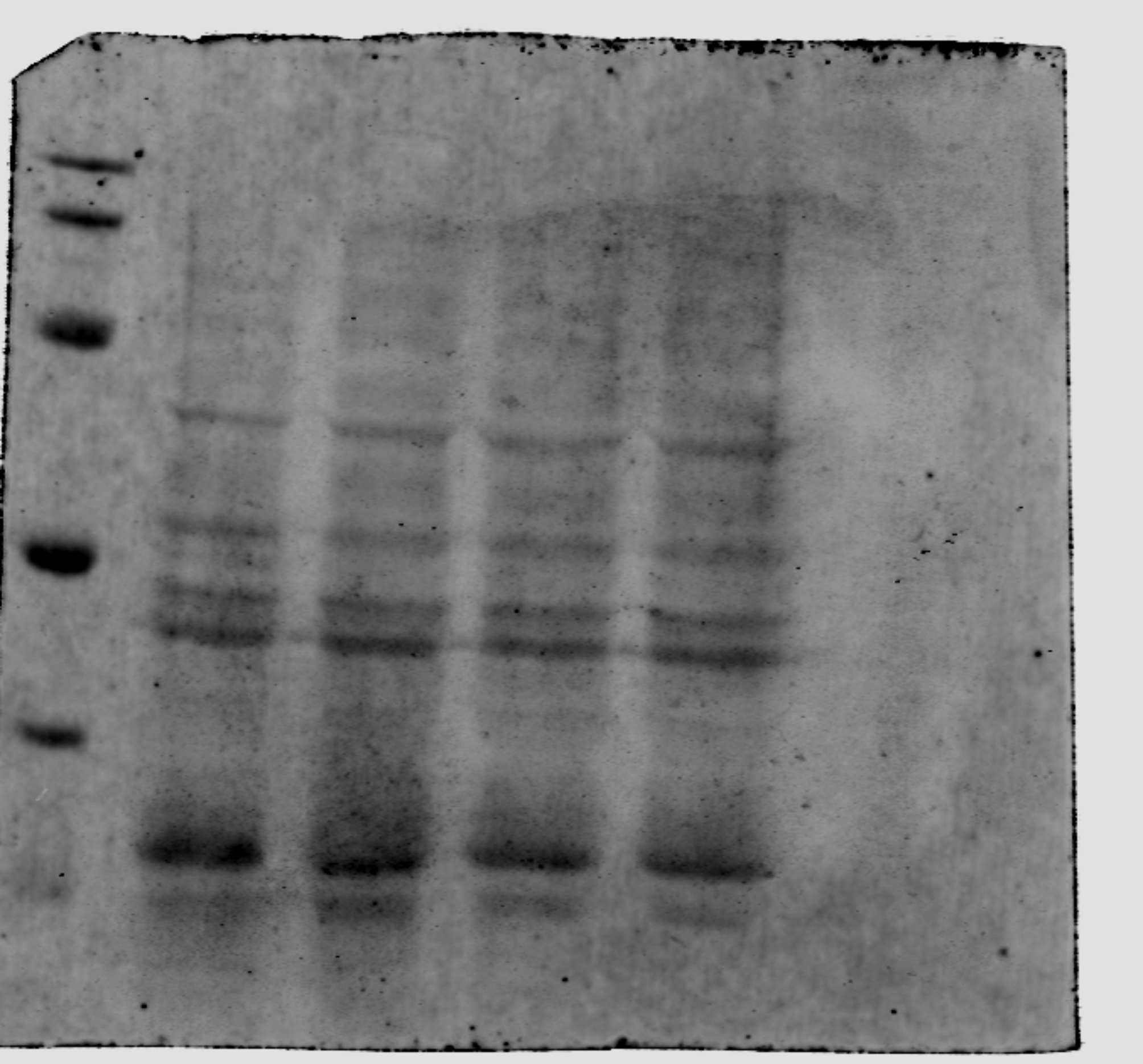

Supplement: Multimedia component 2 [file mmc2.zip › Supplemental_files/Figure 2/Figure 2D/LM3/SNAI2.tif]

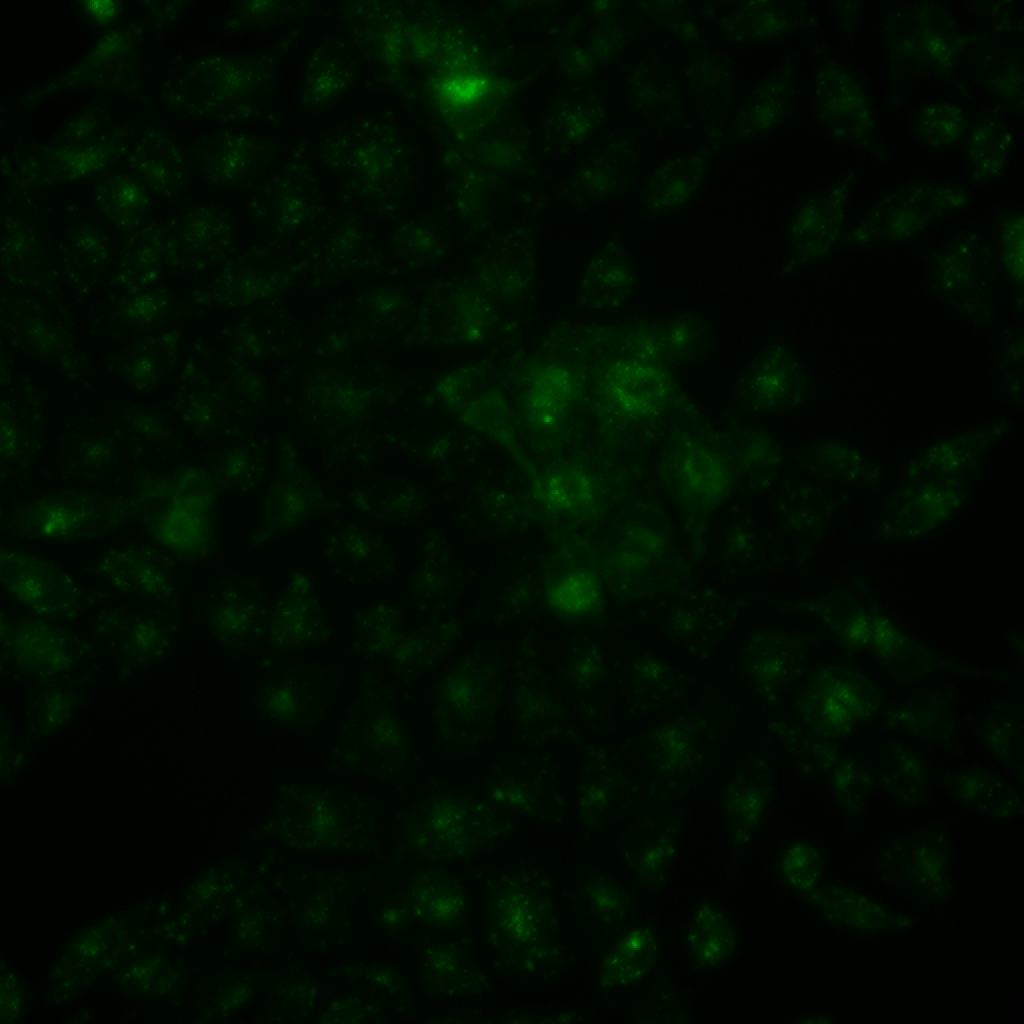

Supplement: Multimedia component 2 [file mmc2.zip › Supplemental_files/Figure 3/Figure 3A/HepG2/0.tif]

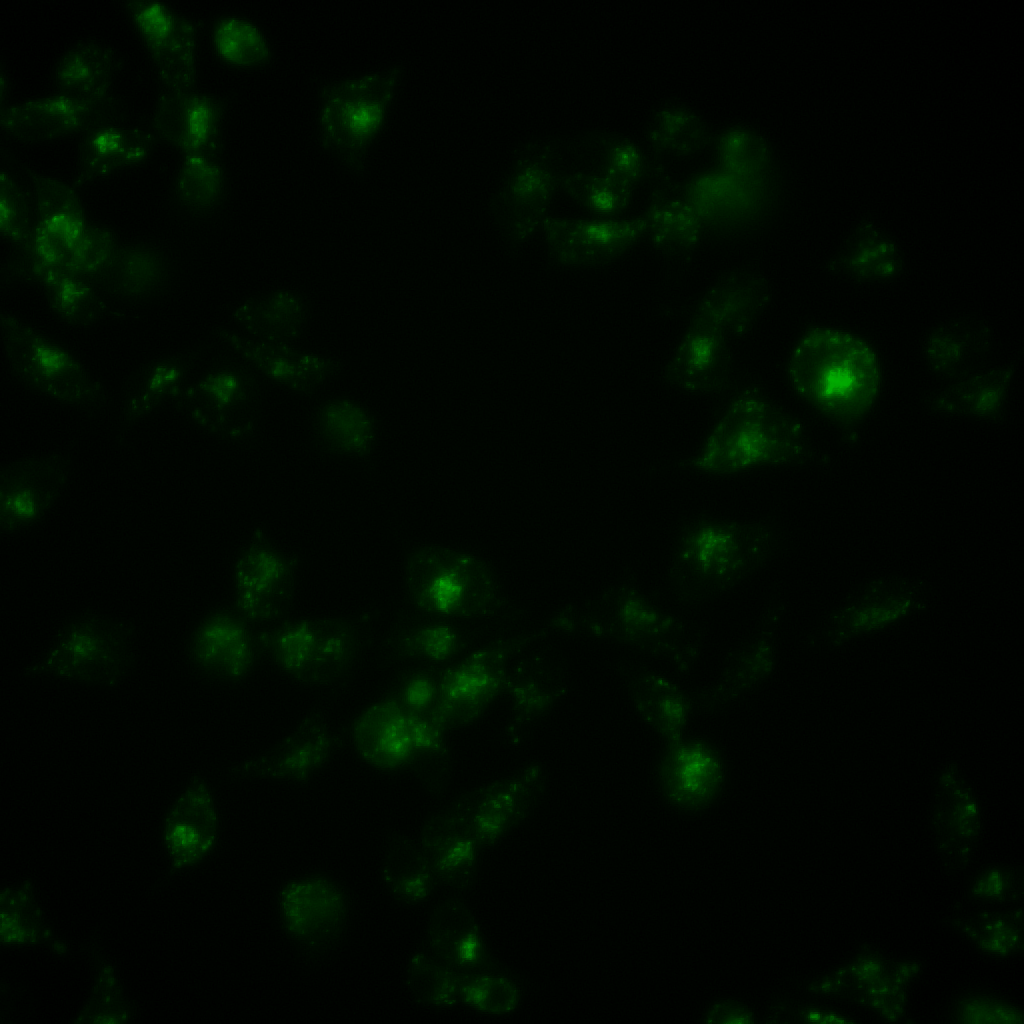

Supplement: Multimedia component 2 [file mmc2.zip › Supplemental_files/Figure 3/Figure 3A/HepG2/200.tif]

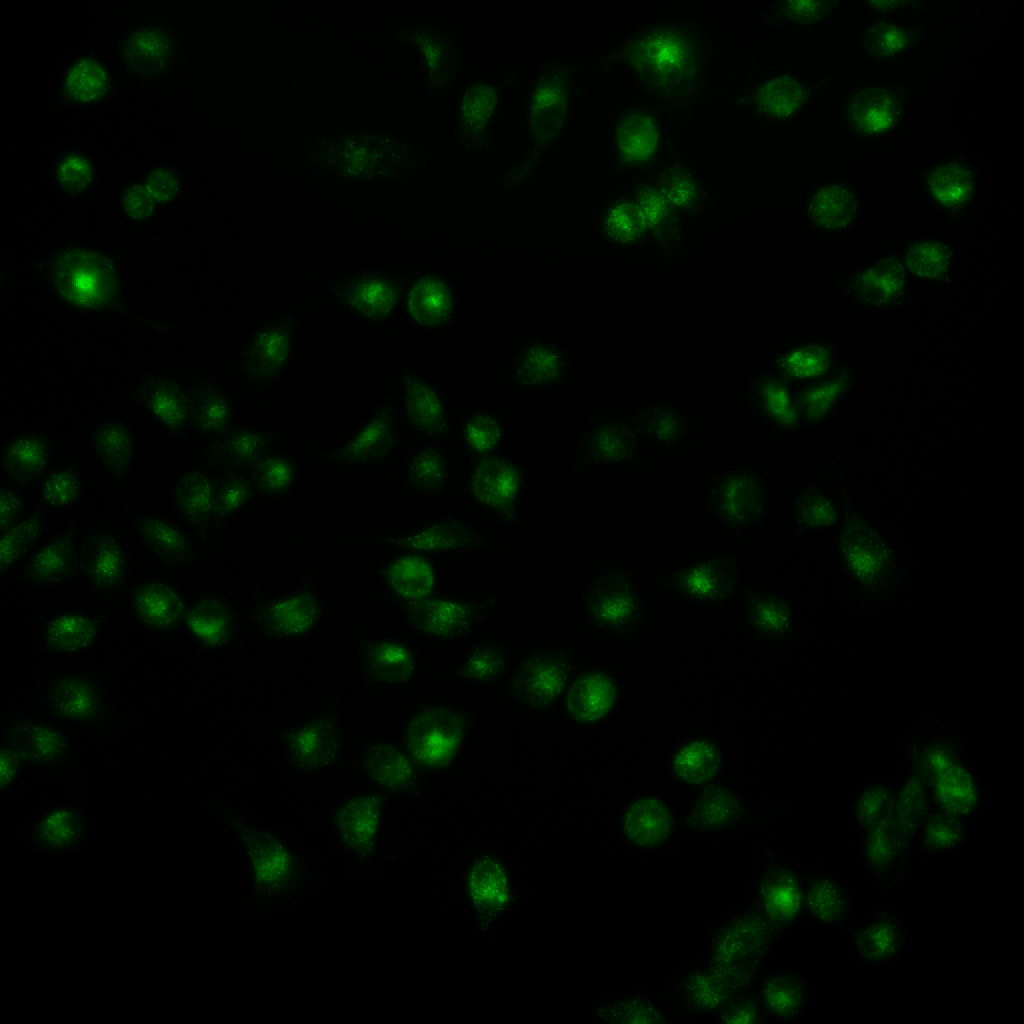

Supplement: Multimedia component 2 [file mmc2.zip › Supplemental_files/Figure 3/Figure 3A/HepG2/450.tif]

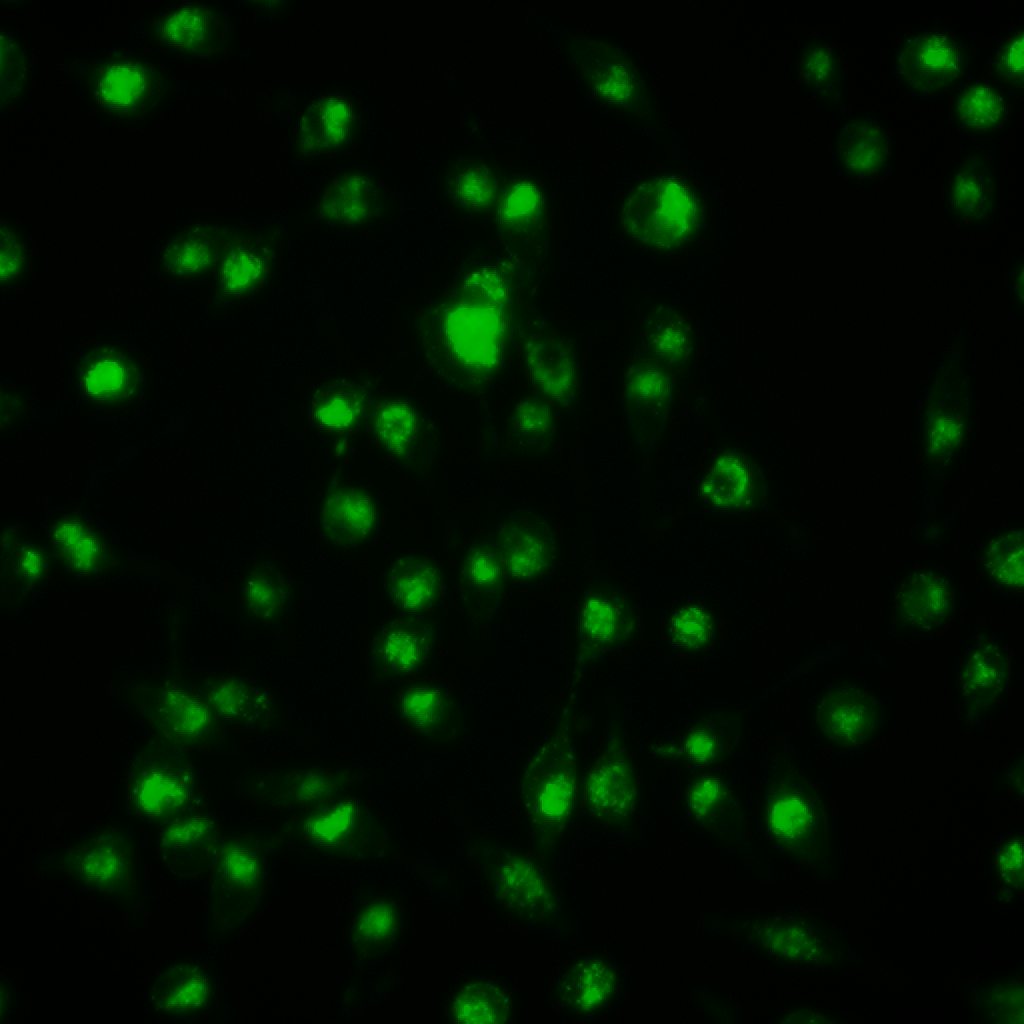

Supplement: Multimedia component 2 [file mmc2.zip › Supplemental_files/Figure 3/Figure 3A/HepG2/600.tif]

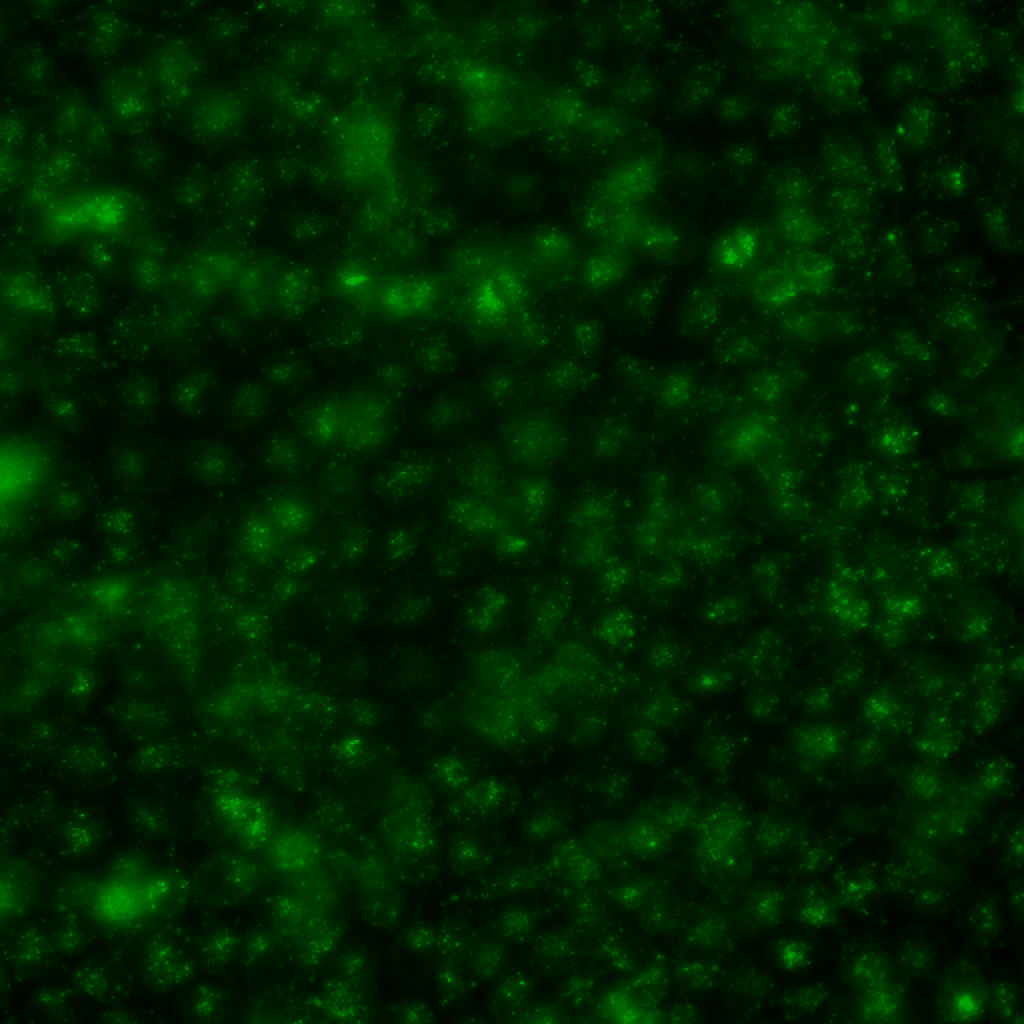

Supplement: Multimedia component 2 [file mmc2.zip › Supplemental_files/Figure 3/Figure 3A/LM3/0.tif]

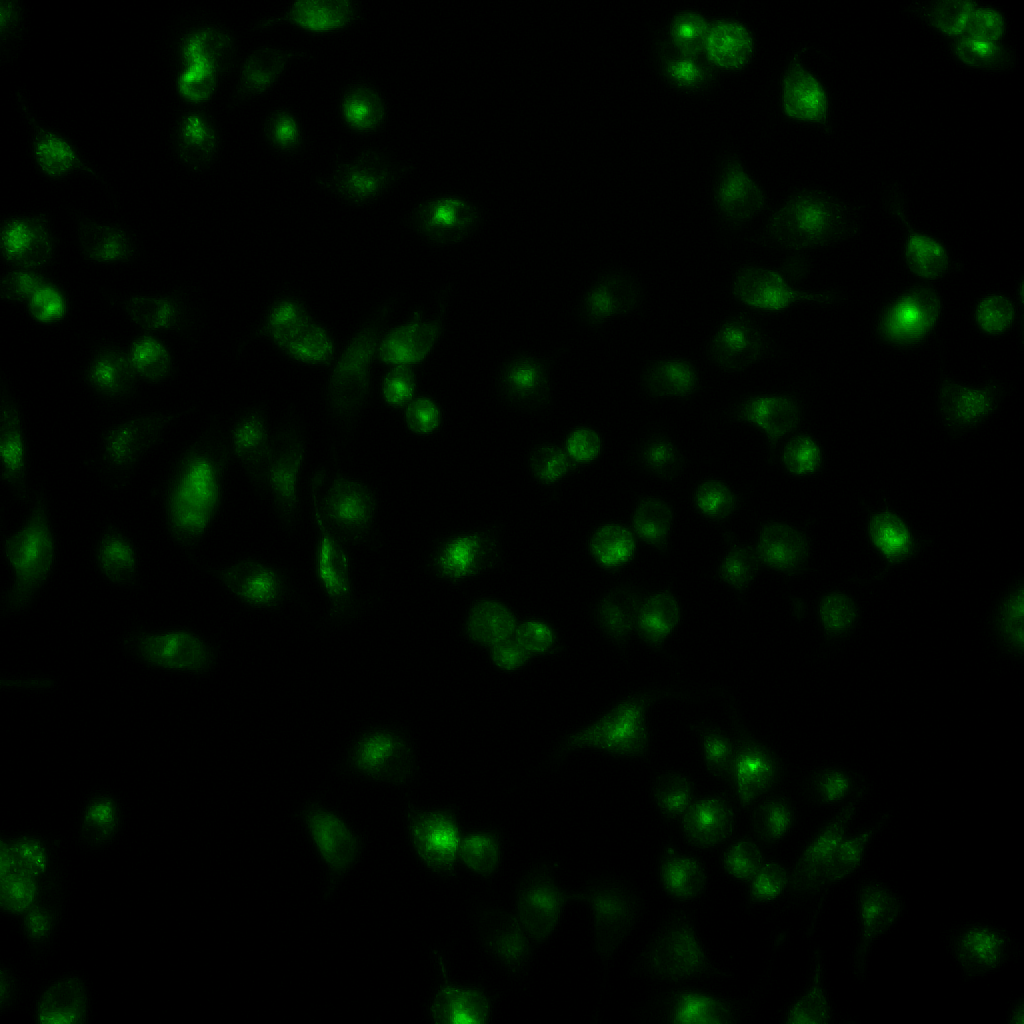

Supplement: Multimedia component 2 [file mmc2.zip › Supplemental_files/Figure 3/Figure 3A/LM3/200.tif]

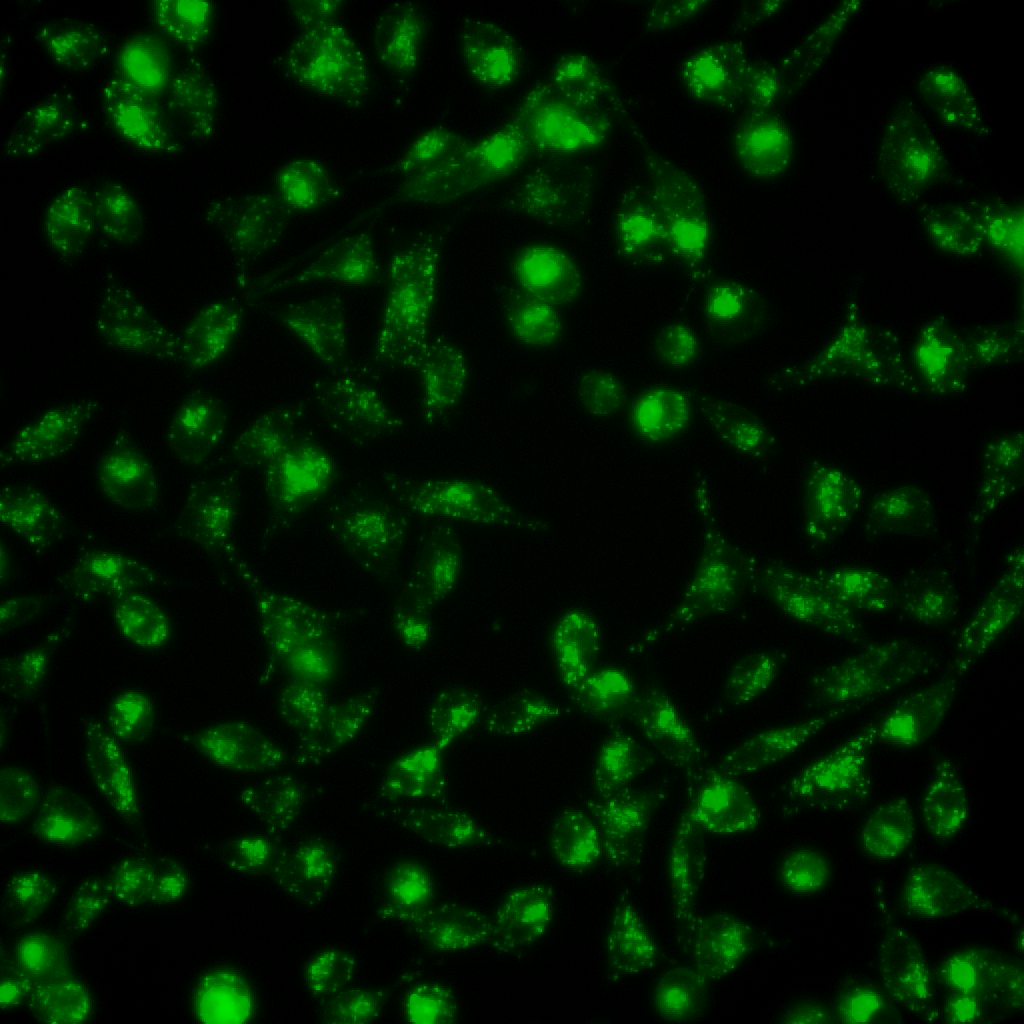

Supplement: Multimedia component 2 [file mmc2.zip › Supplemental_files/Figure 3/Figure 3A/LM3/450 .tif]

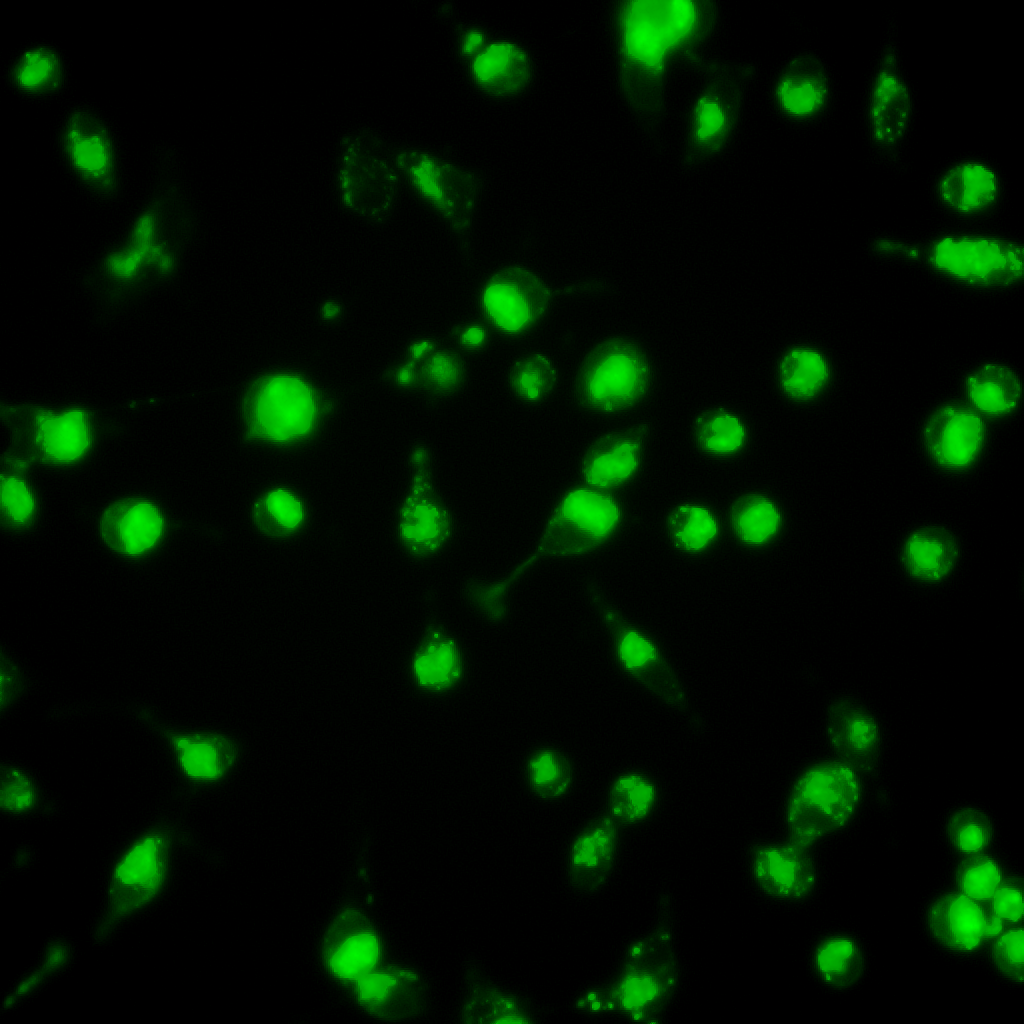

Supplement: Multimedia component 2 [file mmc2.zip › Supplemental_files/Figure 3/Figure 3A/LM3/600.tif]

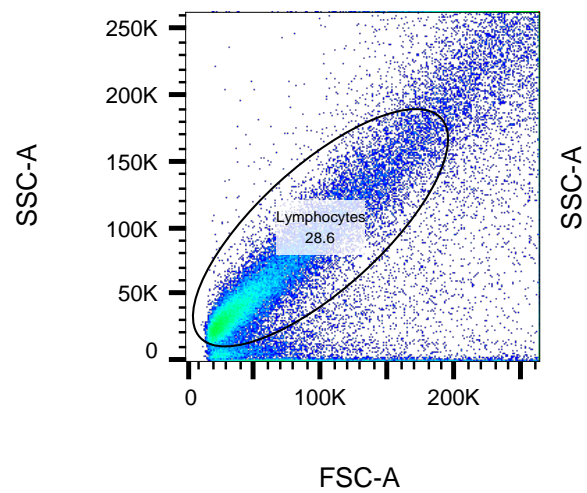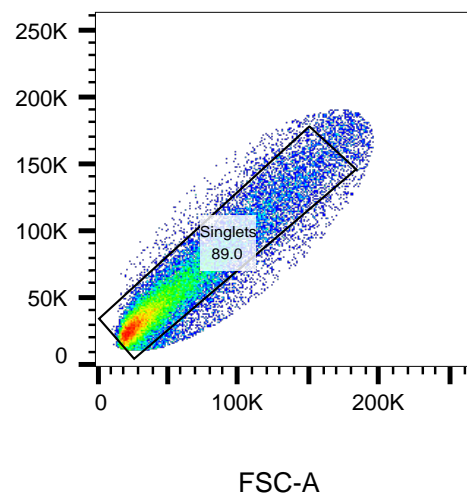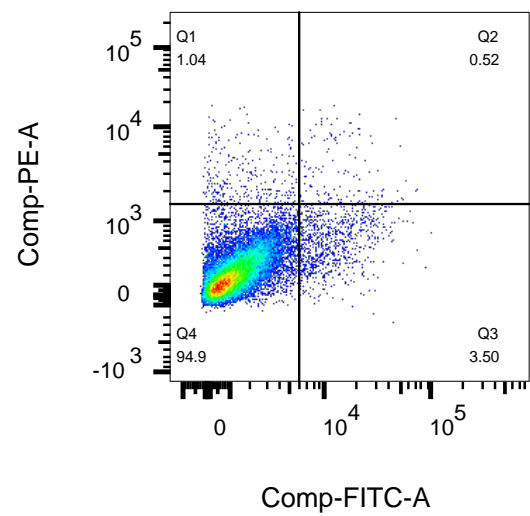

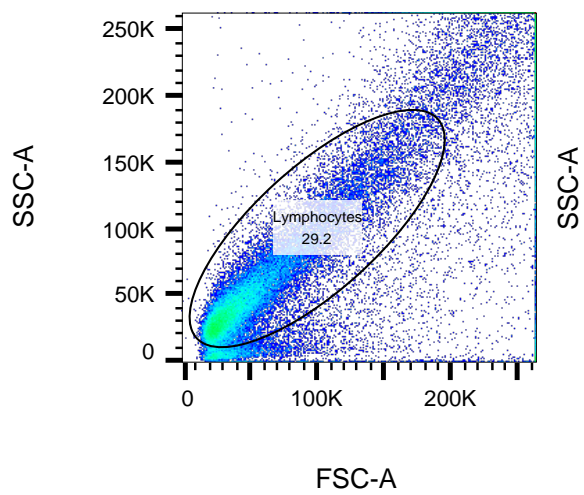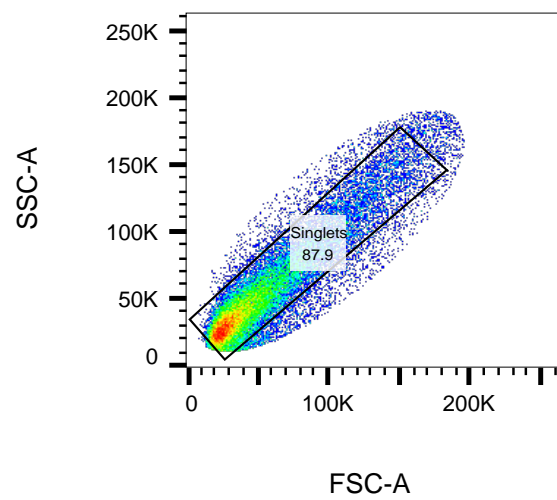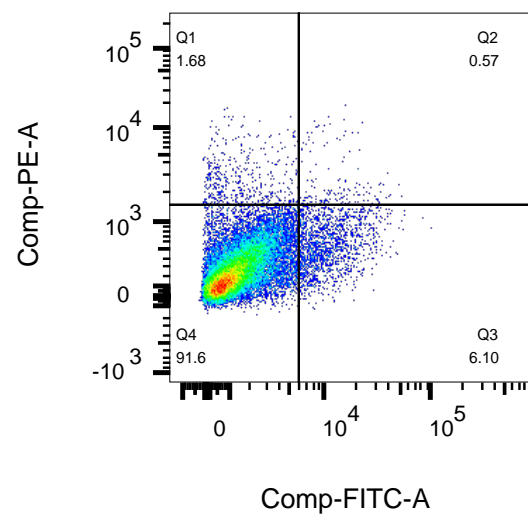

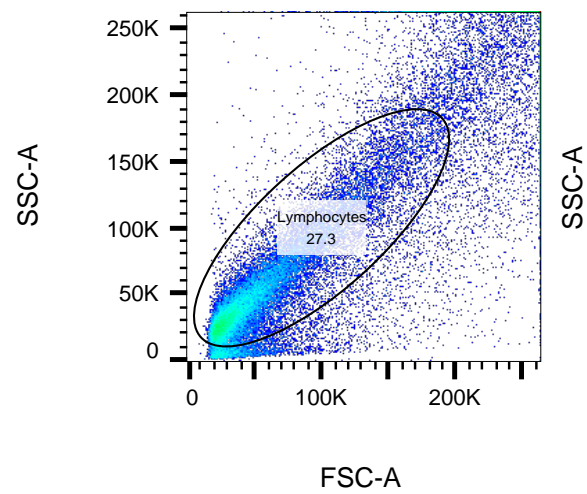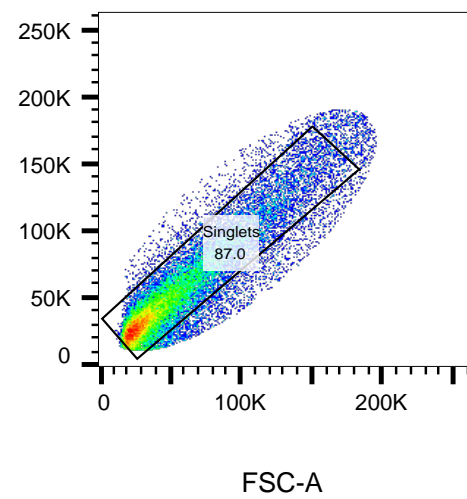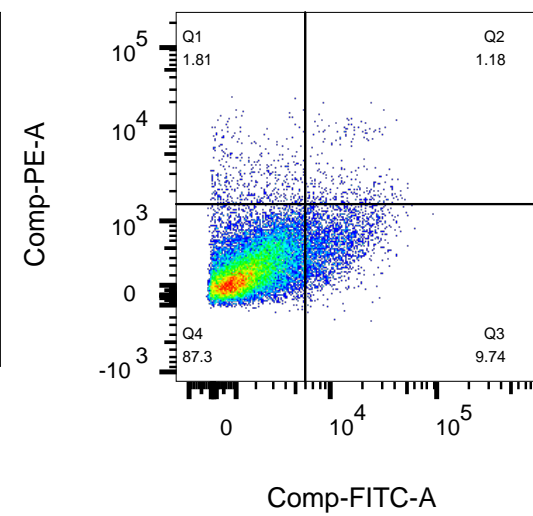

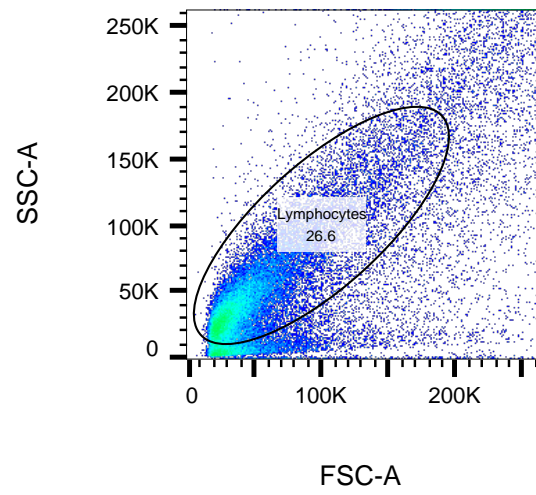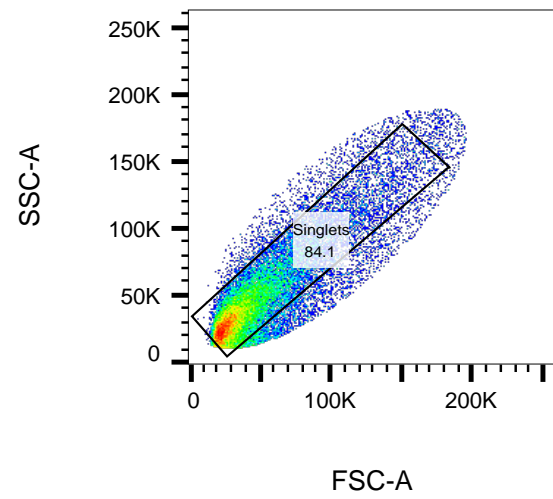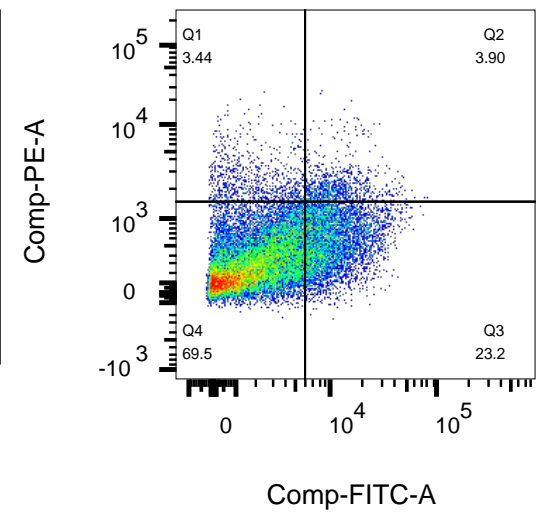

Supplement: Multimedia component 2 [file mmc2.zip › Supplemental_files/Figure 3/Figure 3B/HepG2.pdf]

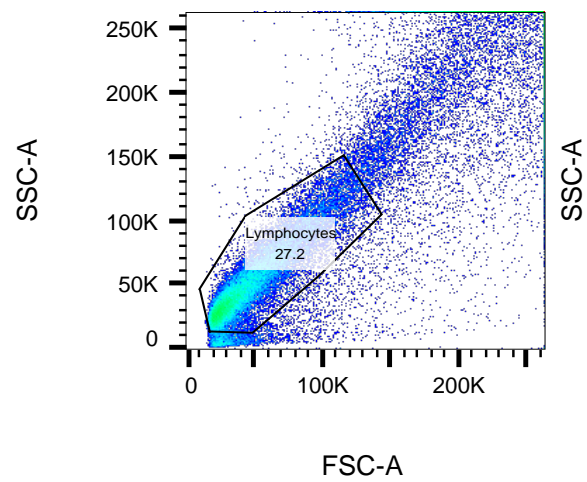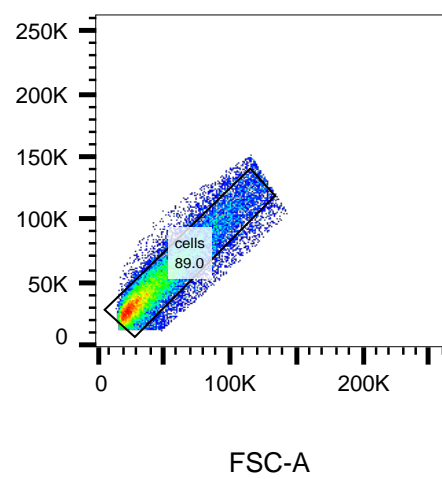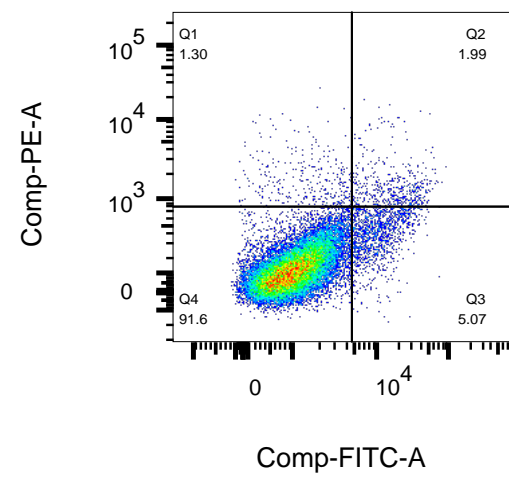

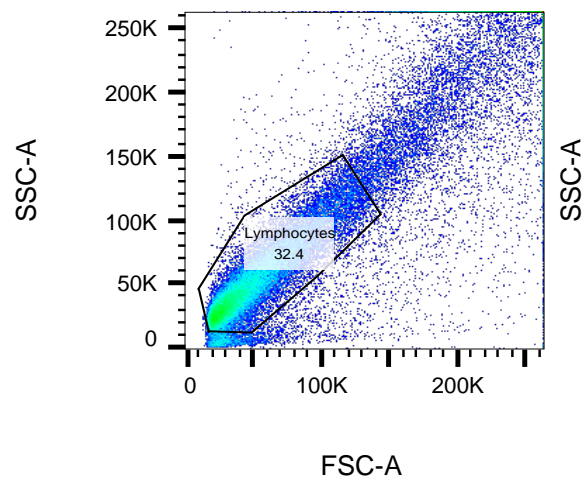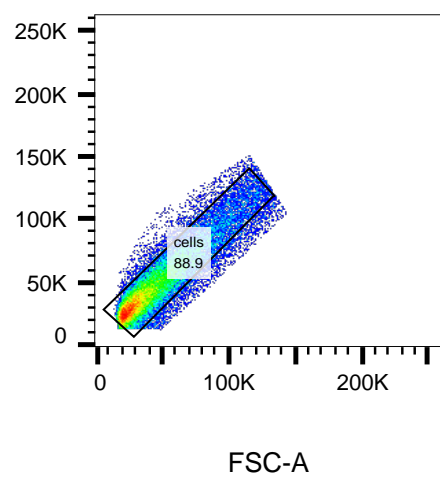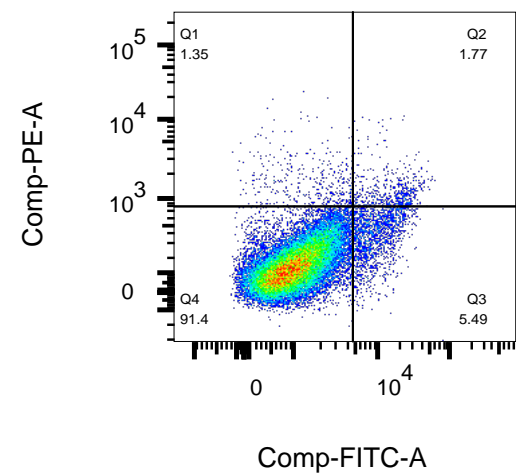

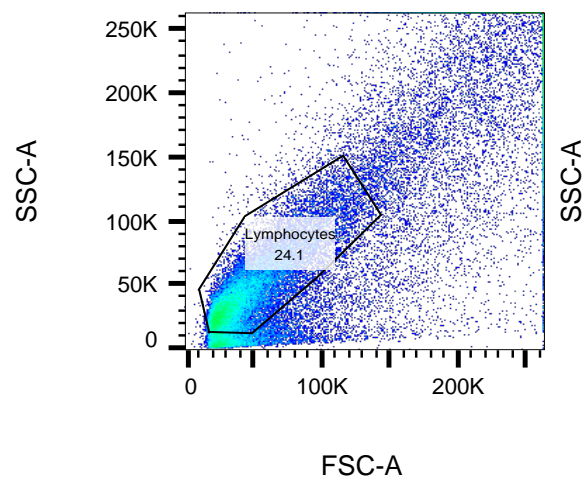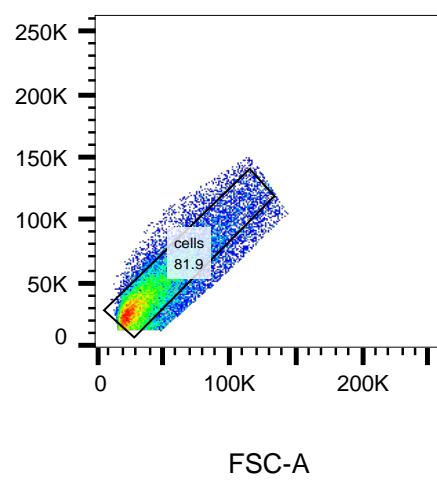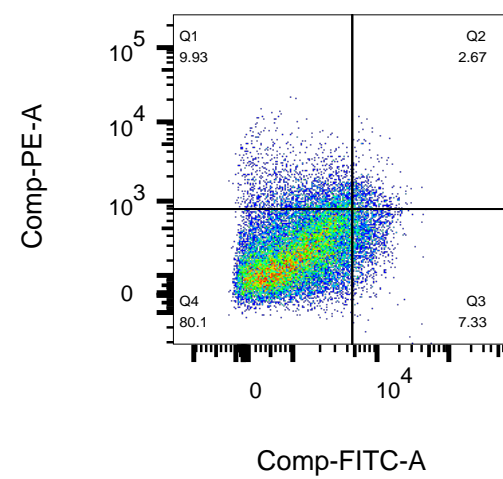

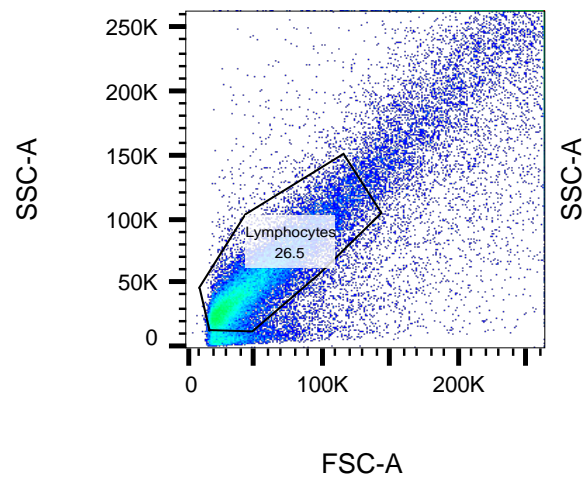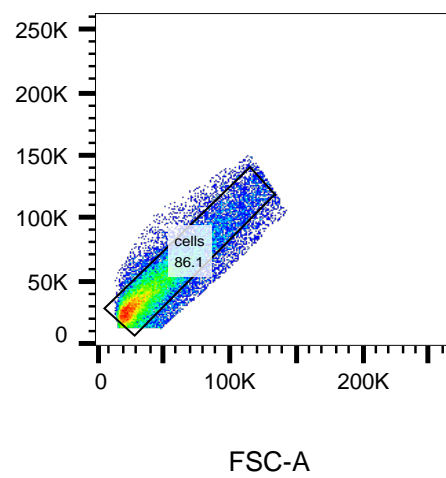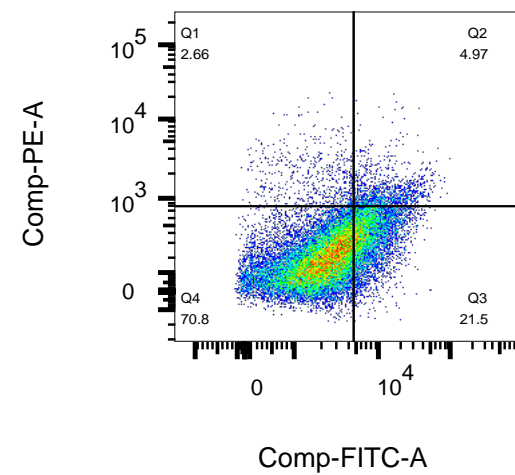

Supplement: Multimedia component 2 [file mmc2.zip › Supplemental_files/Figure 3/Figure 3B/LM3.pdf]

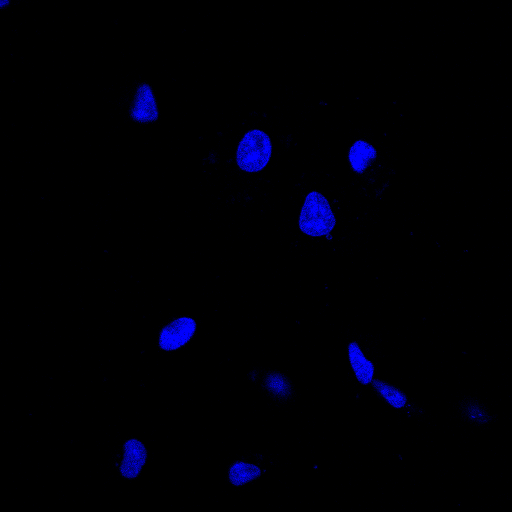

Supplement: Multimedia component 2 [file mmc2.zip › Supplemental_files/Figure 3/Figure 3C/HepG2/0_DAPI.tif]

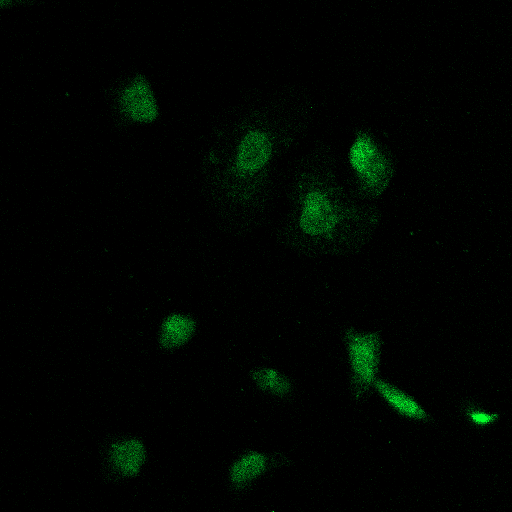

Supplement: Multimedia component 2 [file mmc2.zip › Supplemental_files/Figure 3/Figure 3C/HepG2/0_LC3.tif]

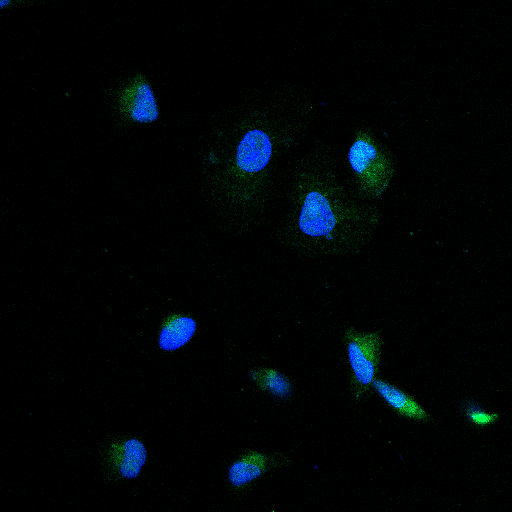

Supplement: Multimedia component 2 [file mmc2.zip › Supplemental_files/Figure 3/Figure 3C/HepG2/0_merge.tif]

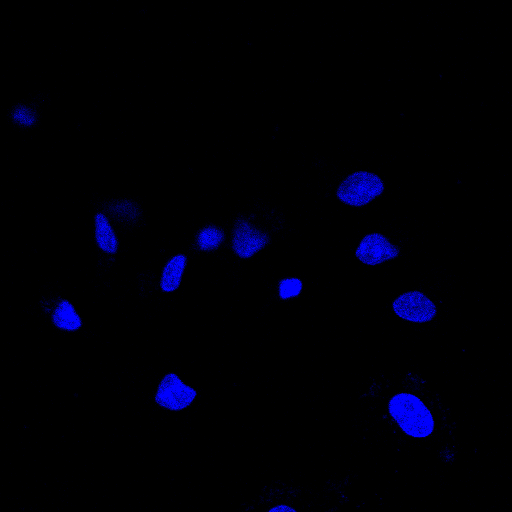

Supplement: Multimedia component 2 [file mmc2.zip › Supplemental_files/Figure 3/Figure 3C/HepG2/450_DAPI.tif]

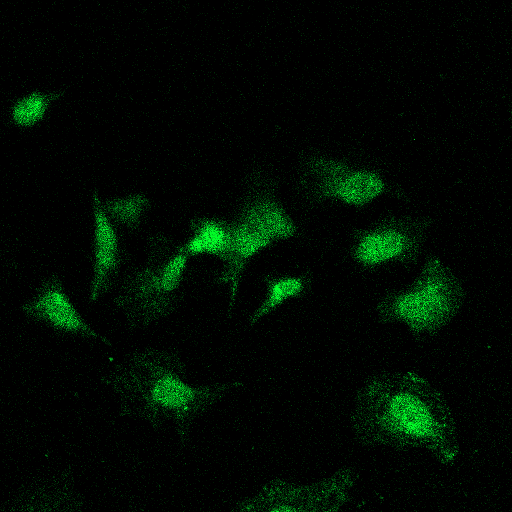

Supplement: Multimedia component 2 [file mmc2.zip › Supplemental_files/Figure 3/Figure 3C/HepG2/450_LC3.tif]

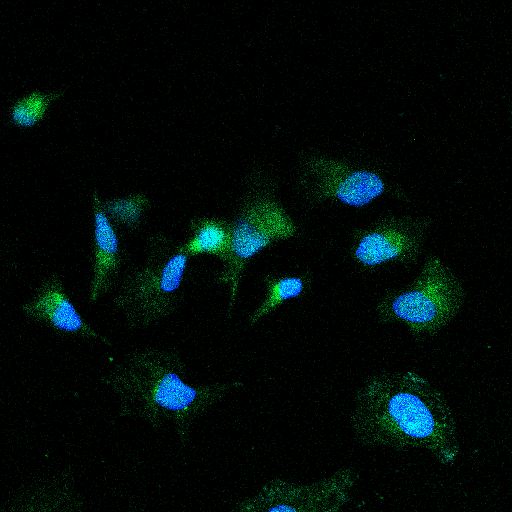

Supplement: Multimedia component 2 [file mmc2.zip › Supplemental_files/Figure 3/Figure 3C/HepG2/450_merge.tif]

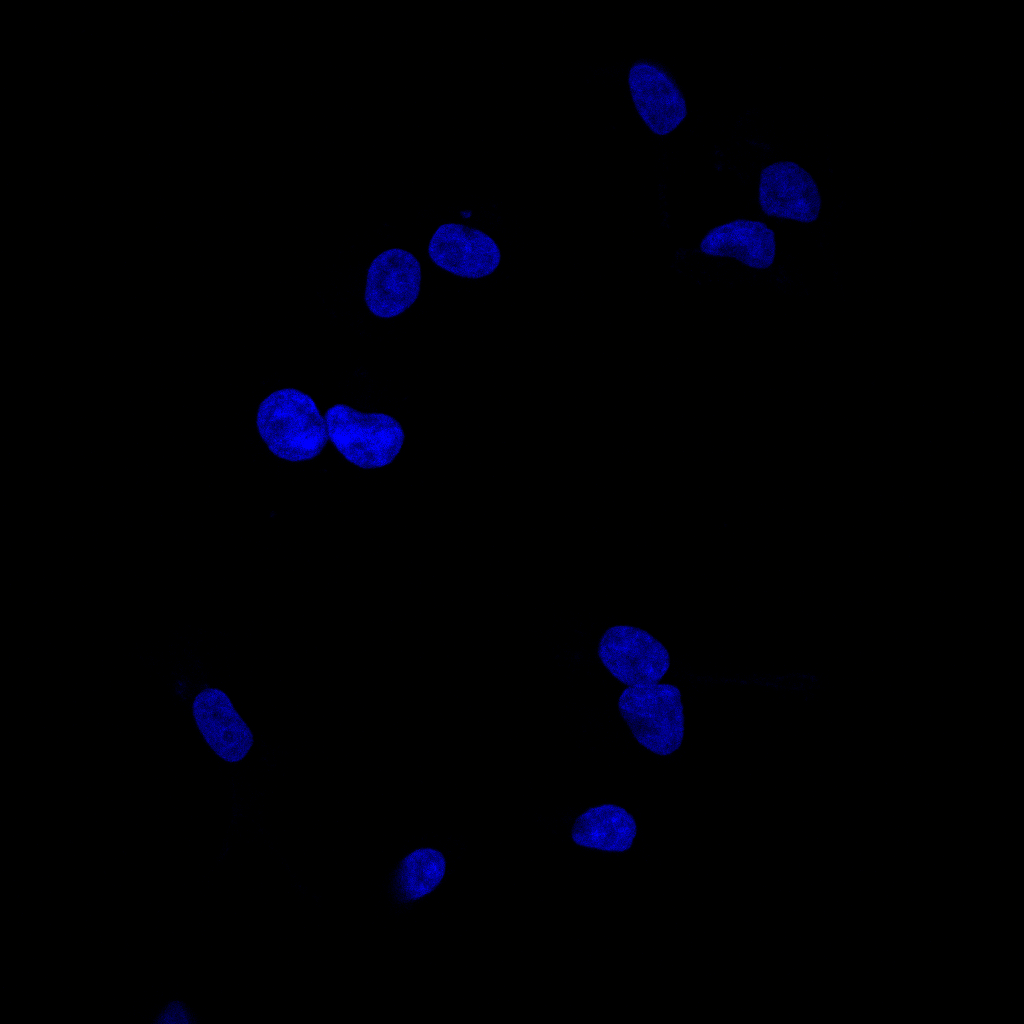

Supplement: Multimedia component 2 [file mmc2.zip › Supplemental_files/Figure 3/Figure 3C/LM3/0_DAPI.tif]

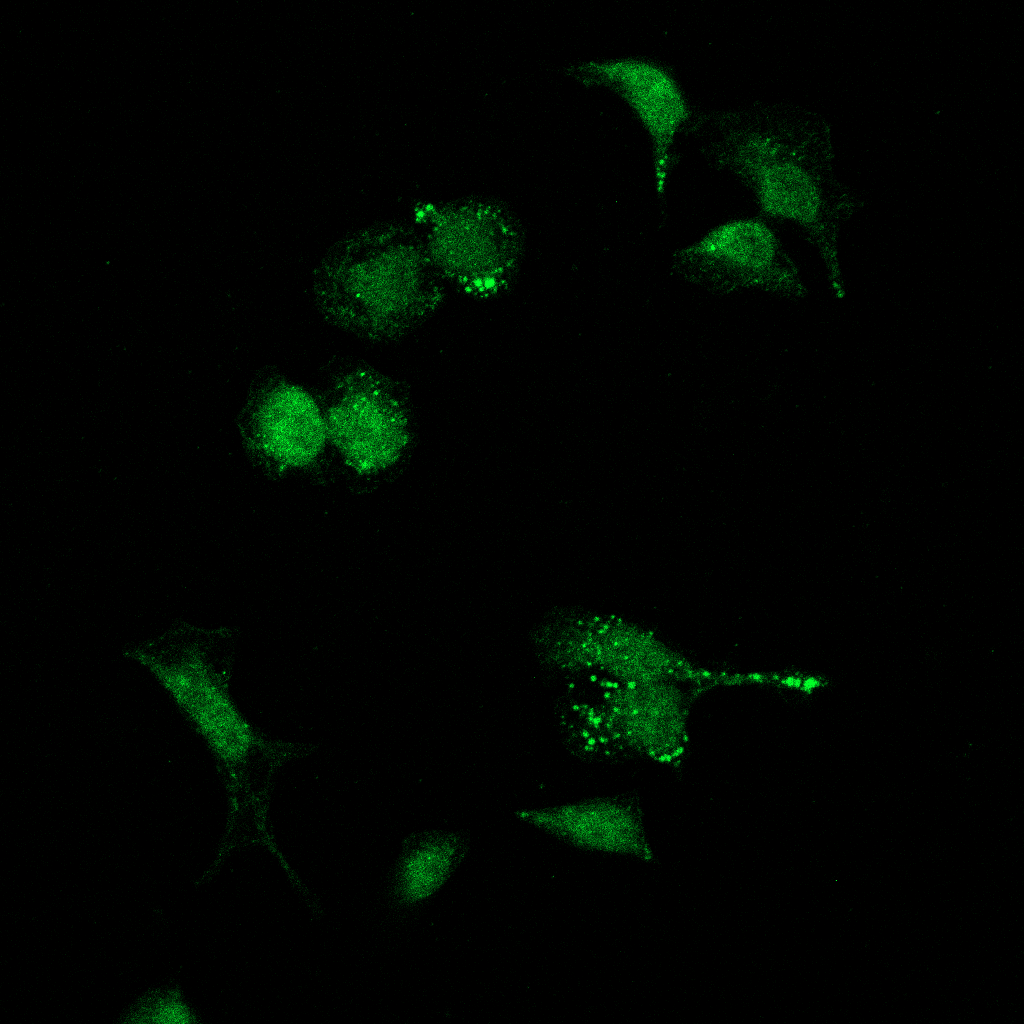

Supplement: Multimedia component 2 [file mmc2.zip › Supplemental_files/Figure 3/Figure 3C/LM3/0_LC3.tif]

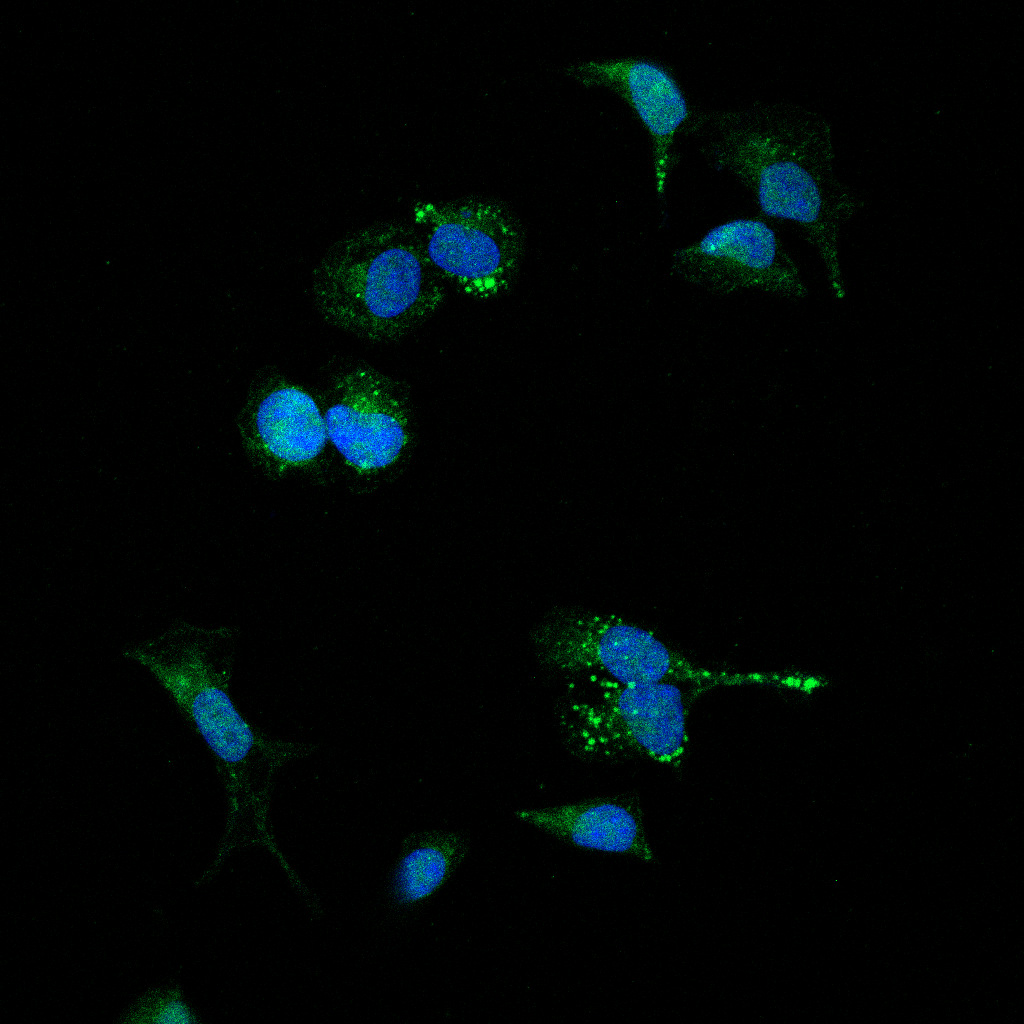

Supplement: Multimedia component 2 [file mmc2.zip › Supplemental_files/Figure 3/Figure 3C/LM3/0_merge.tif]

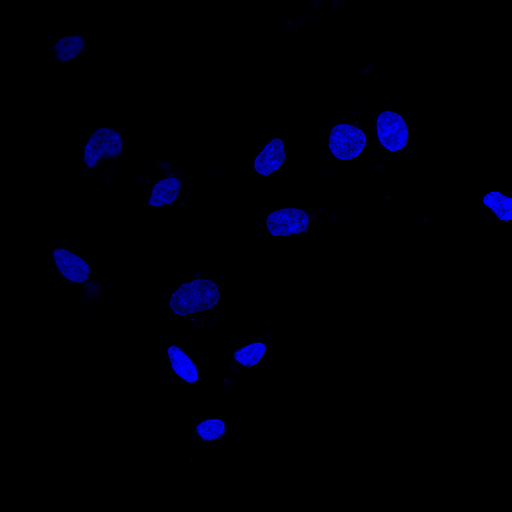

Supplement: Multimedia component 2 [file mmc2.zip › Supplemental_files/Figure 3/Figure 3C/LM3/450_DAPI.tif]

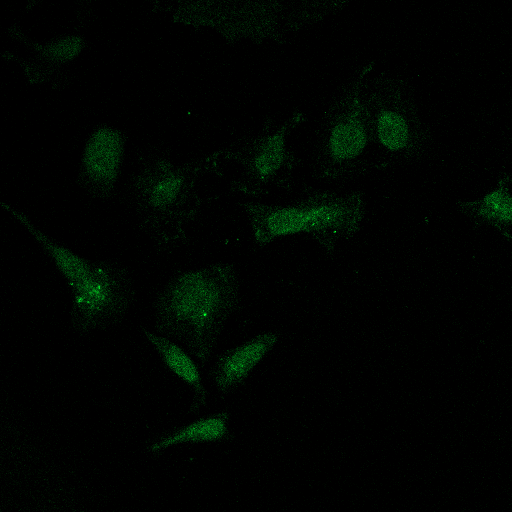

Supplement: Multimedia component 2 [file mmc2.zip › Supplemental_files/Figure 3/Figure 3C/LM3/450_LC3.tif]

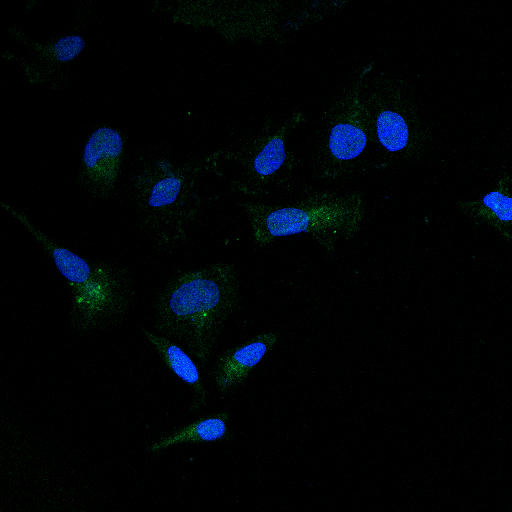

Supplement: Multimedia component 2 [file mmc2.zip › Supplemental_files/Figure 3/Figure 3C/LM3/450_merge.tif]

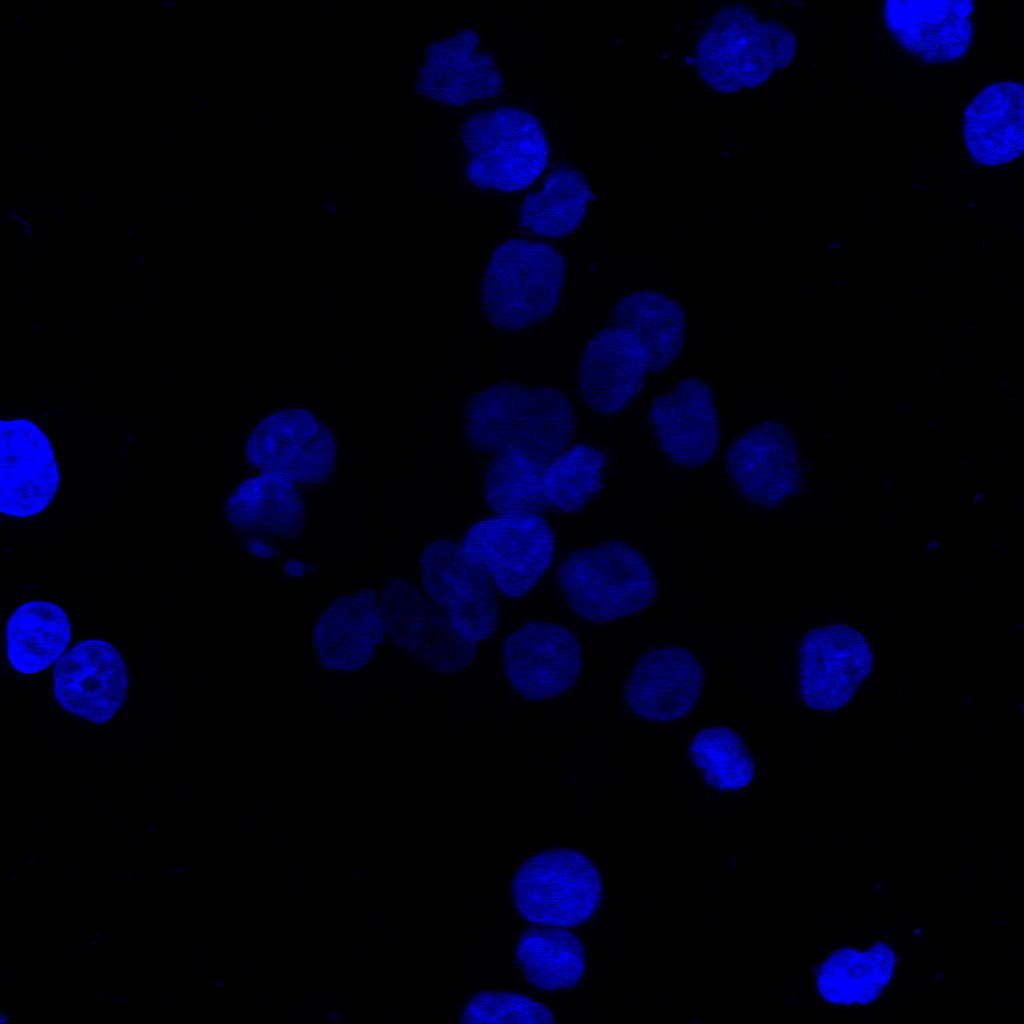

Supplement: Multimedia component 2 [file mmc2.zip › Supplemental_files/Figure 3/Figure 3D/HepG2/0_DAPI.tif]

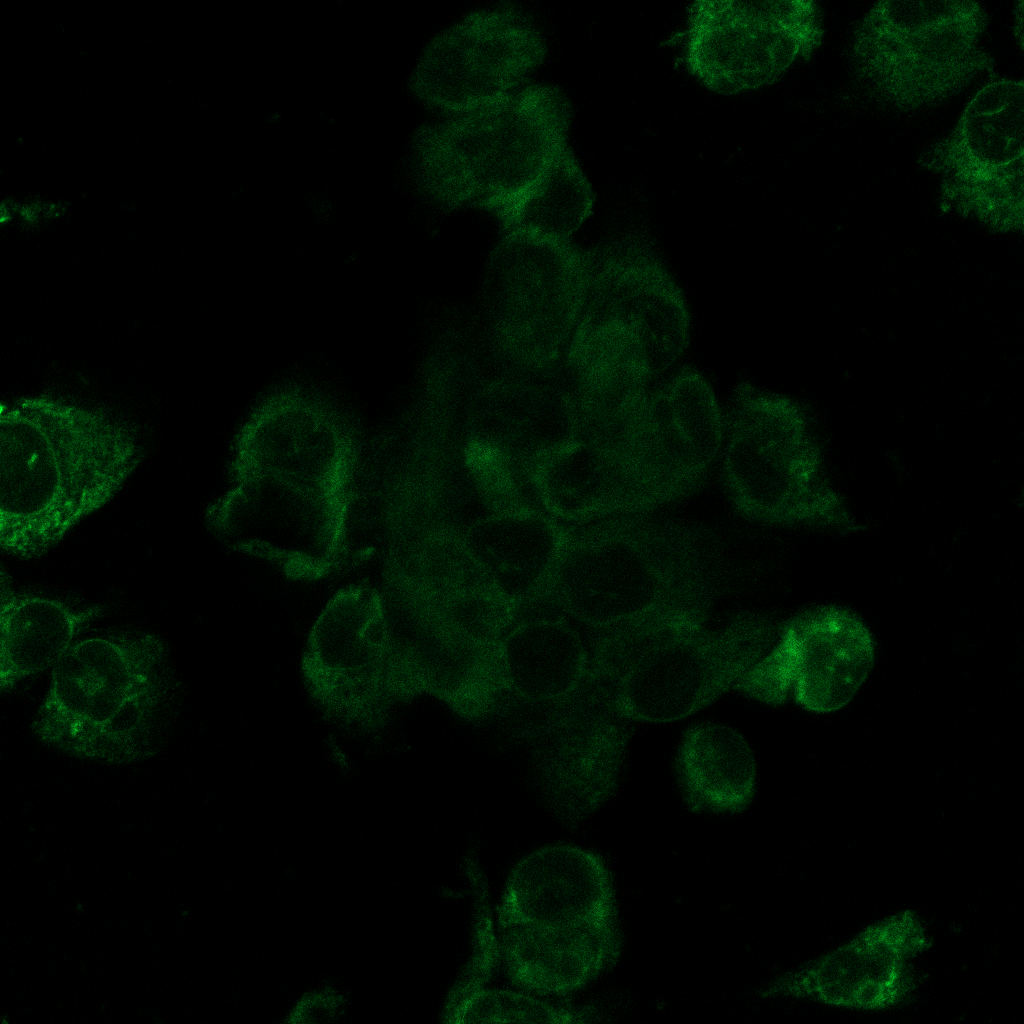

Supplement: Multimedia component 2 [file mmc2.zip › Supplemental_files/Figure 3/Figure 3D/HepG2/0_caspase-3.tif]

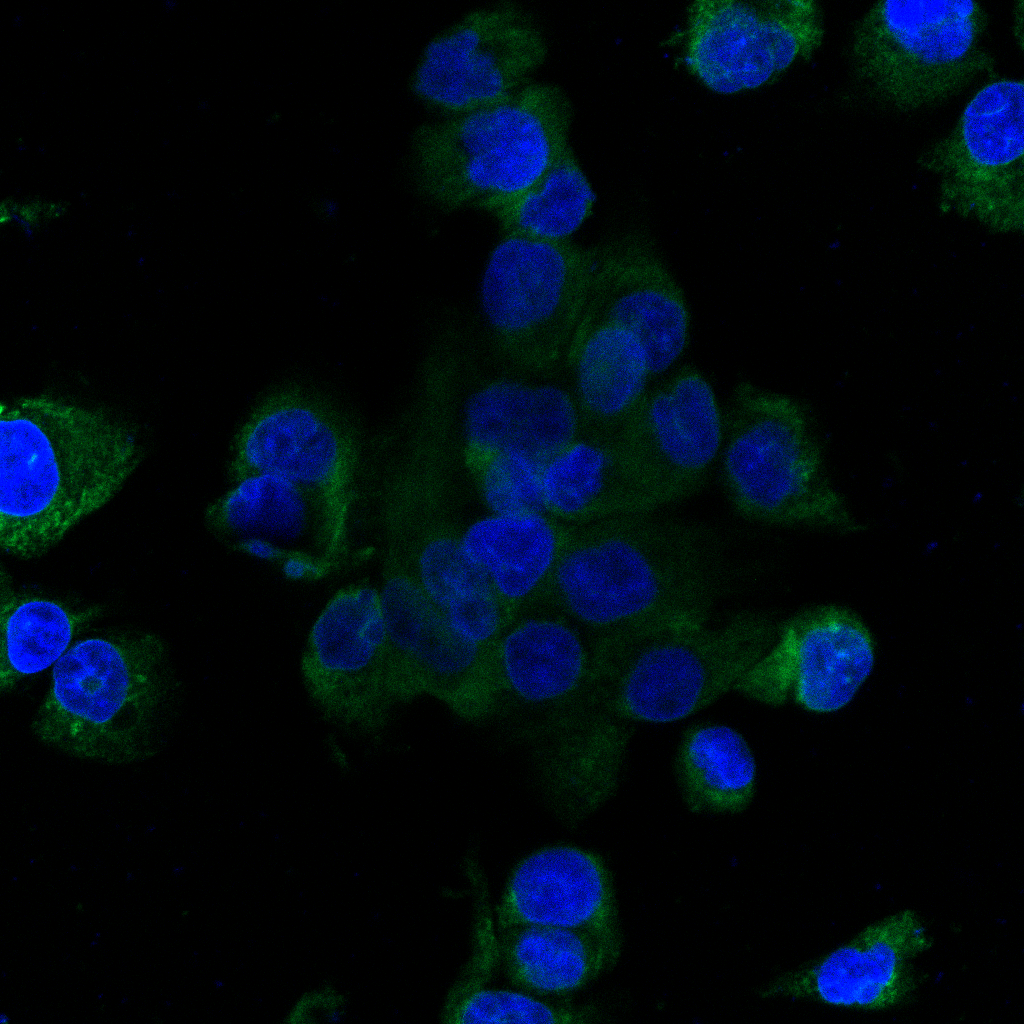

Supplement: Multimedia component 2 [file mmc2.zip › Supplemental_files/Figure 3/Figure 3D/HepG2/0_merge.tif]

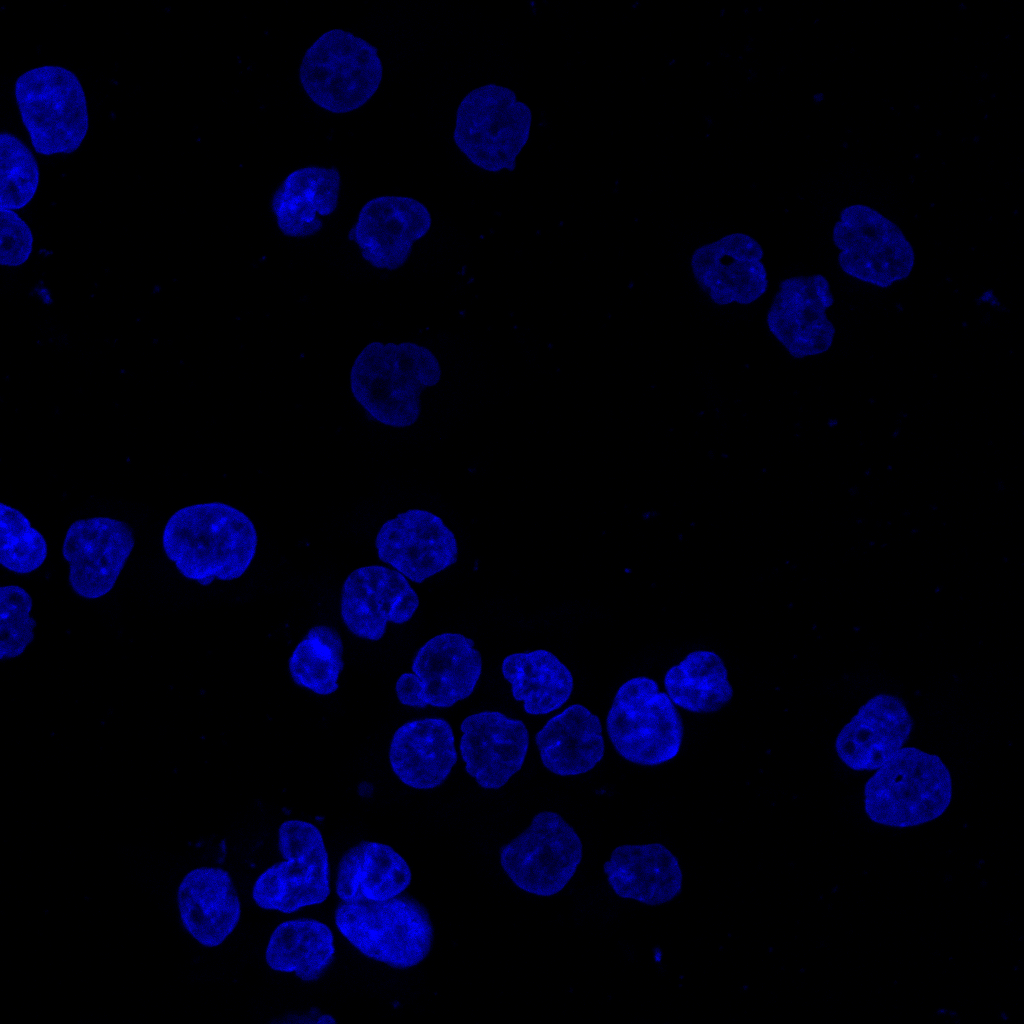

Supplement: Multimedia component 2 [file mmc2.zip › Supplemental_files/Figure 3/Figure 3D/HepG2/450_DAPI.tif]

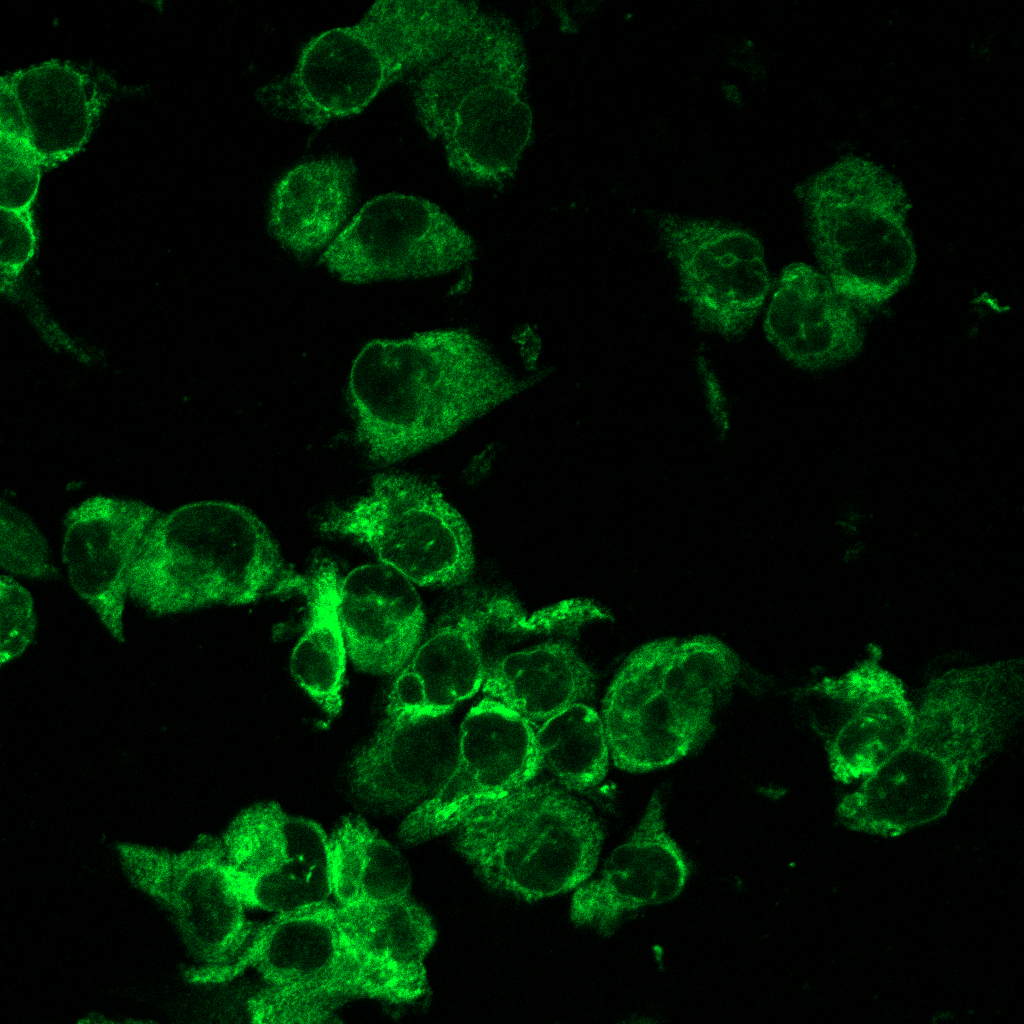

Supplement: Multimedia component 2 [file mmc2.zip › Supplemental_files/Figure 3/Figure 3D/HepG2/450_caspase-3.tif]

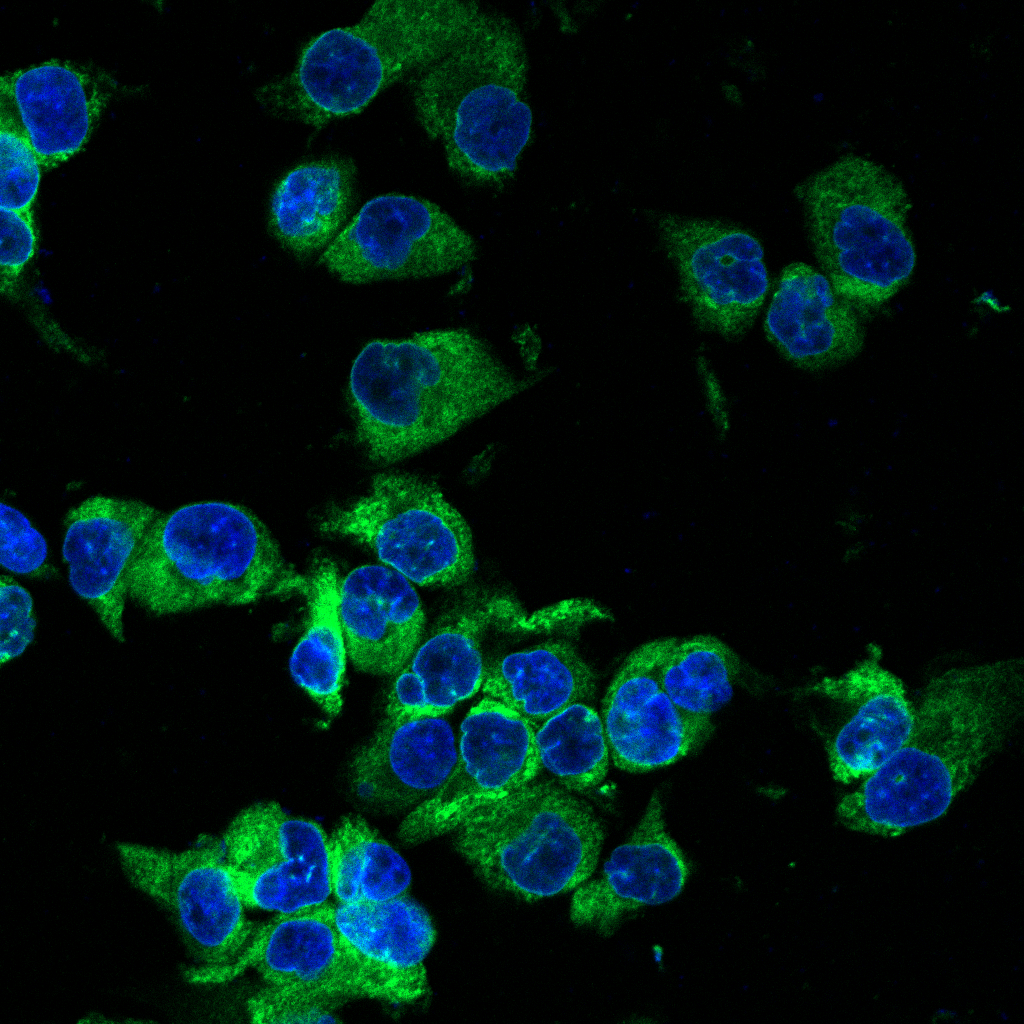

Supplement: Multimedia component 2 [file mmc2.zip › Supplemental_files/Figure 3/Figure 3D/HepG2/450_merge.tif]

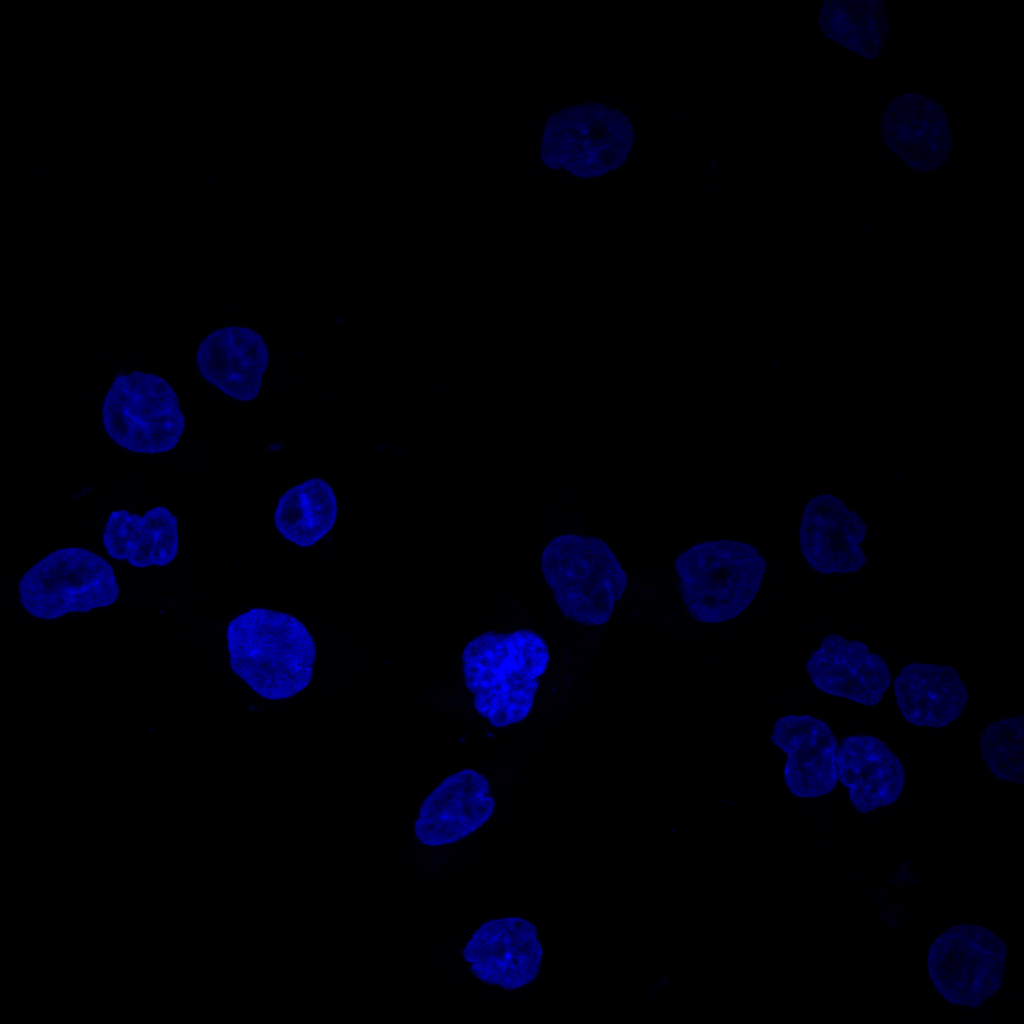

Supplement: Multimedia component 2 [file mmc2.zip › Supplemental_files/Figure 3/Figure 3D/LM3/0_DAPI.tif]

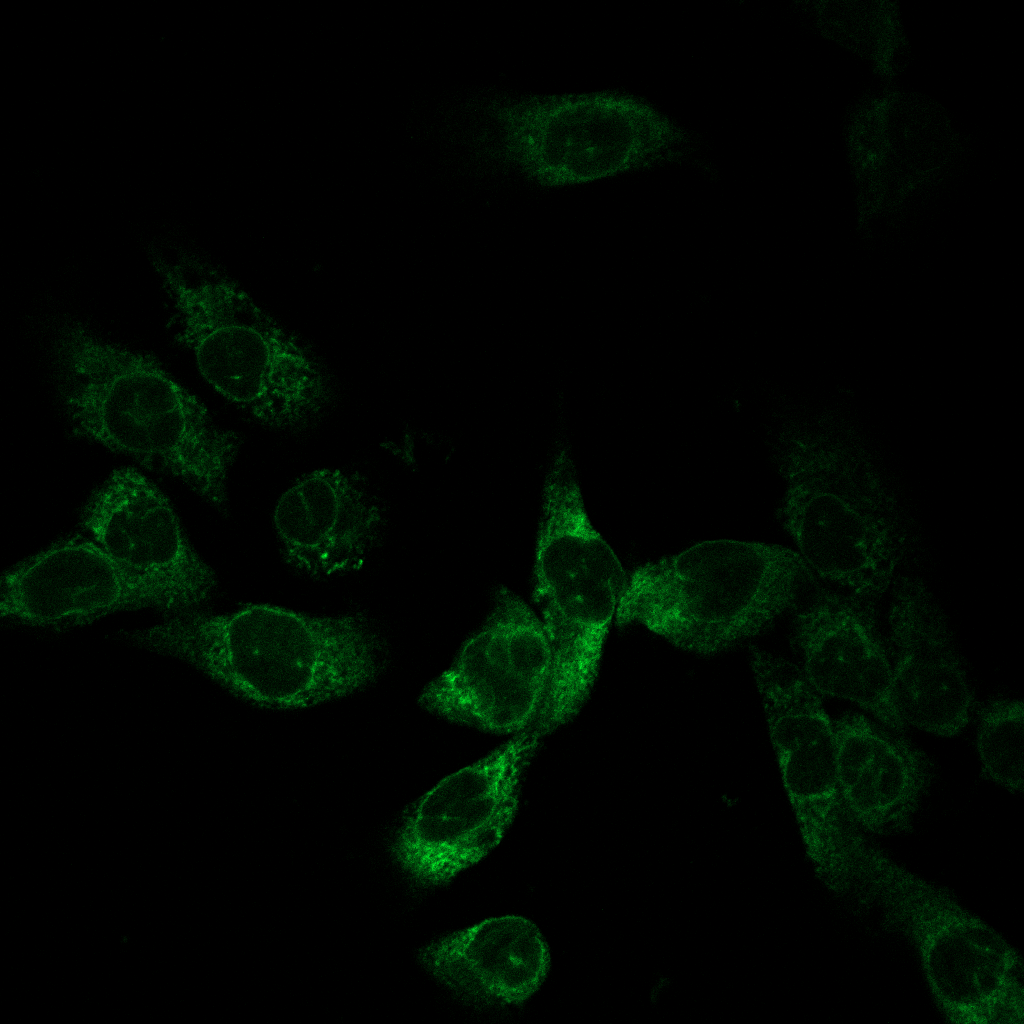

Supplement: Multimedia component 2 [file mmc2.zip › Supplemental_files/Figure 3/Figure 3D/LM3/0_caspase-3.tif]

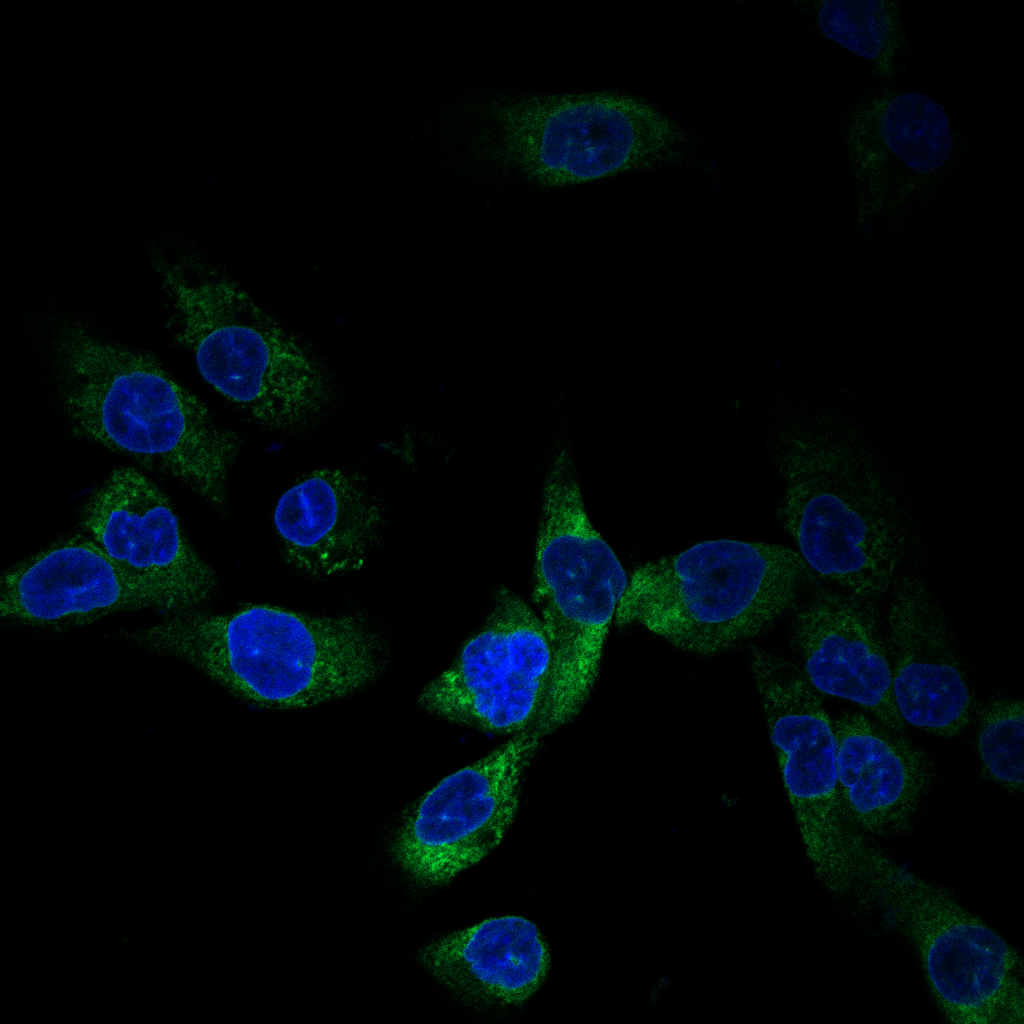

Supplement: Multimedia component 2 [file mmc2.zip › Supplemental_files/Figure 3/Figure 3D/LM3/0_merge.tif]

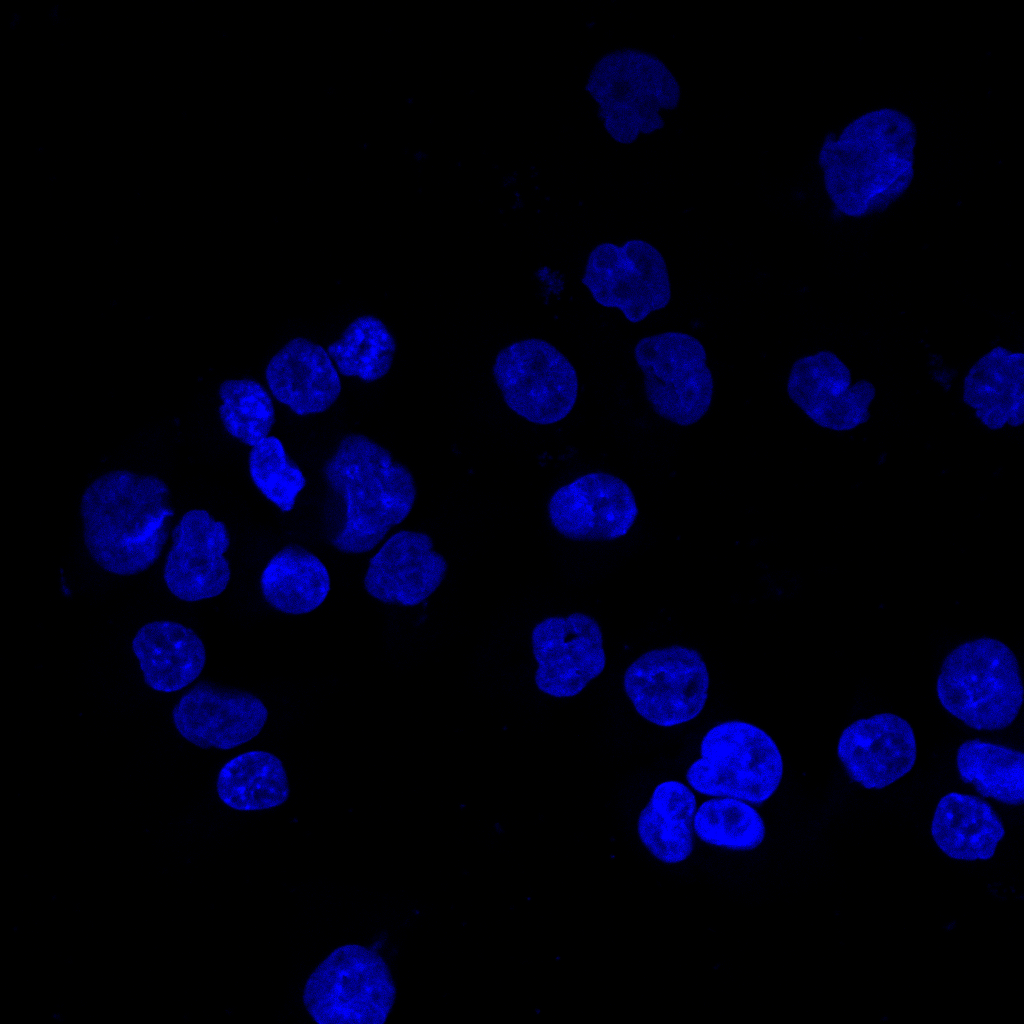

Supplement: Multimedia component 2 [file mmc2.zip › Supplemental_files/Figure 3/Figure 3D/LM3/450_DAPI.tif]

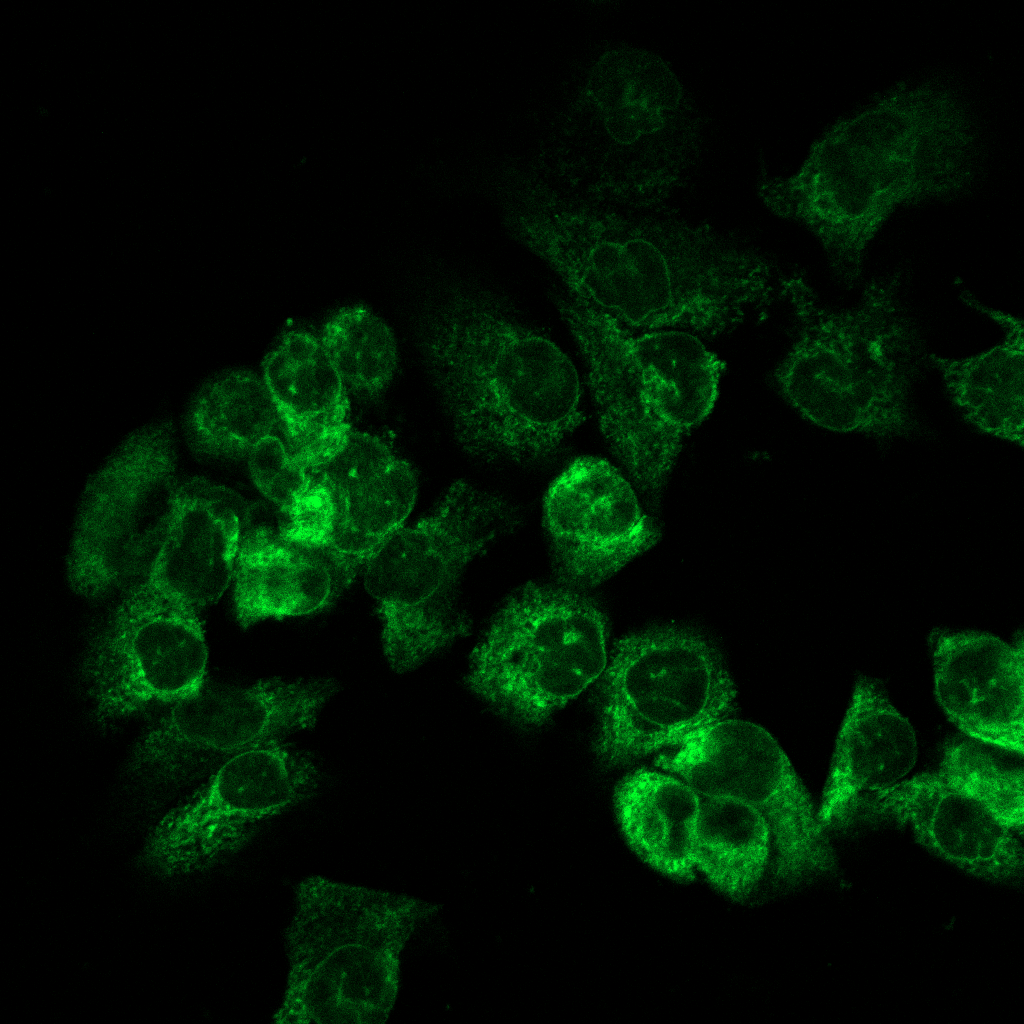

Supplement: Multimedia component 2 [file mmc2.zip › Supplemental_files/Figure 3/Figure 3D/LM3/450_caspase-3.tif]

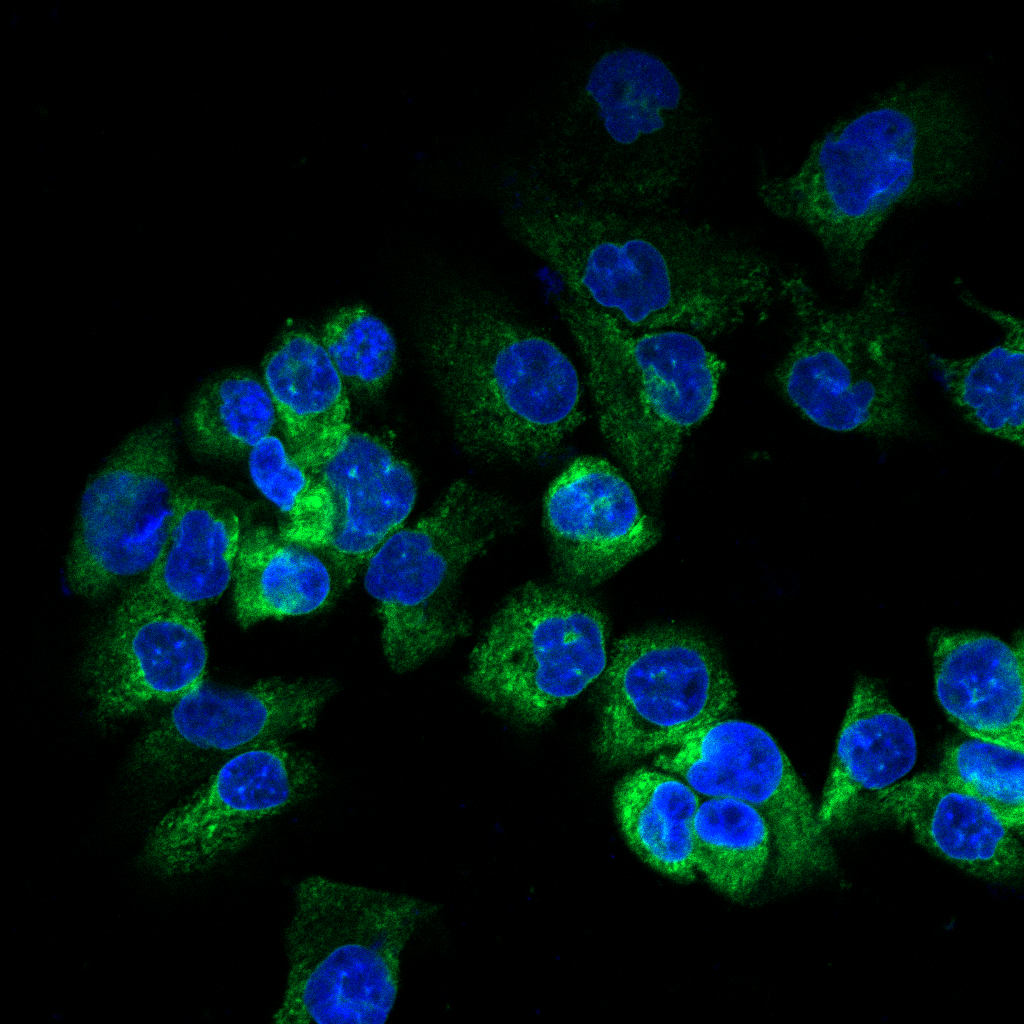

Supplement: Multimedia component 2 [file mmc2.zip › Supplemental_files/Figure 3/Figure 3D/LM3/450_merge.tif]

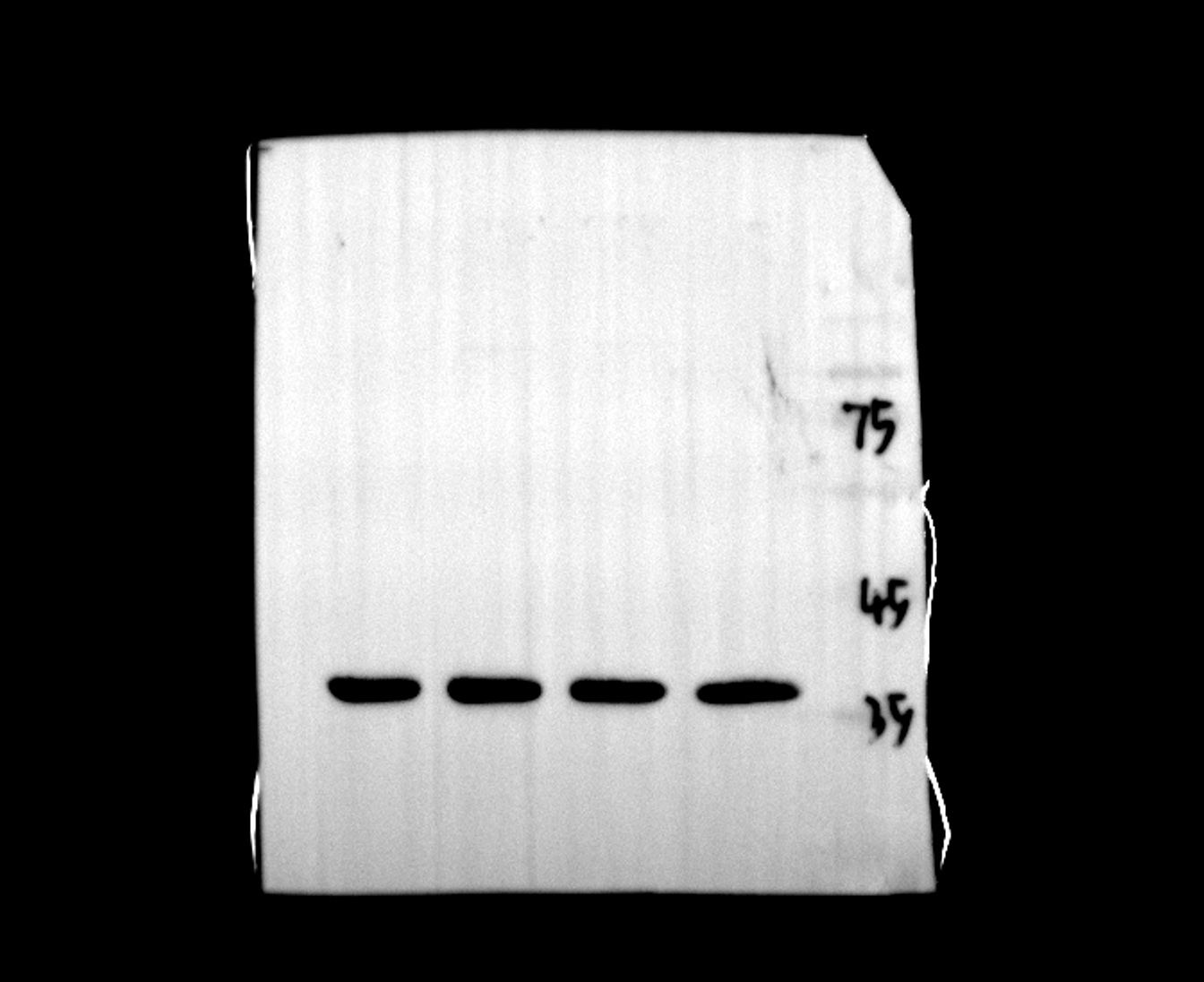

Supplement: Multimedia component 2 [file mmc2.zip › Supplemental_files/Figure 3/Figure 3E/HepG2/GAPDH.Tif]

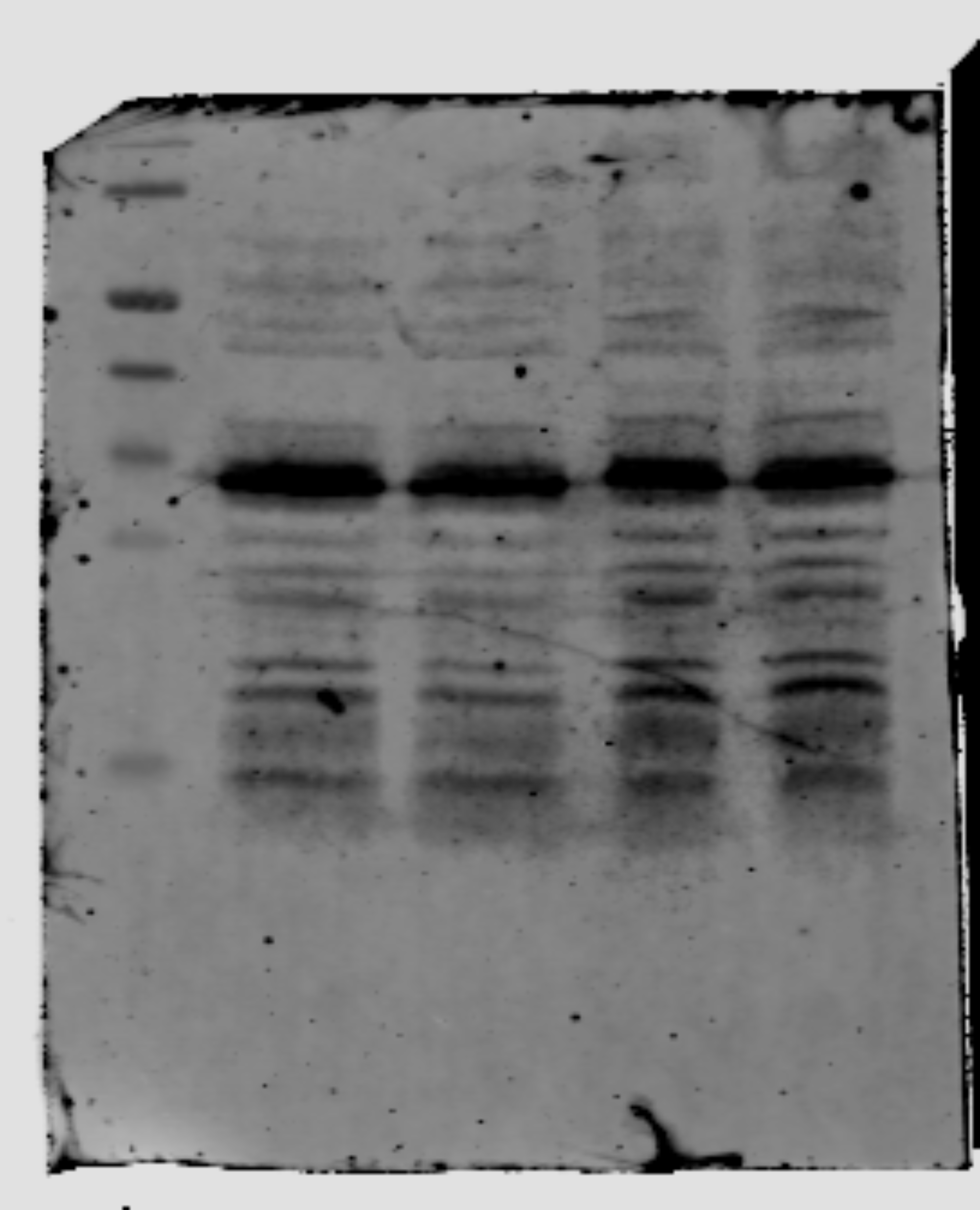

Supplement: Multimedia component 2 [file mmc2.zip › Supplemental_files/Figure 3/Figure 3E/HepG2/LC3I and LC3II.tif]

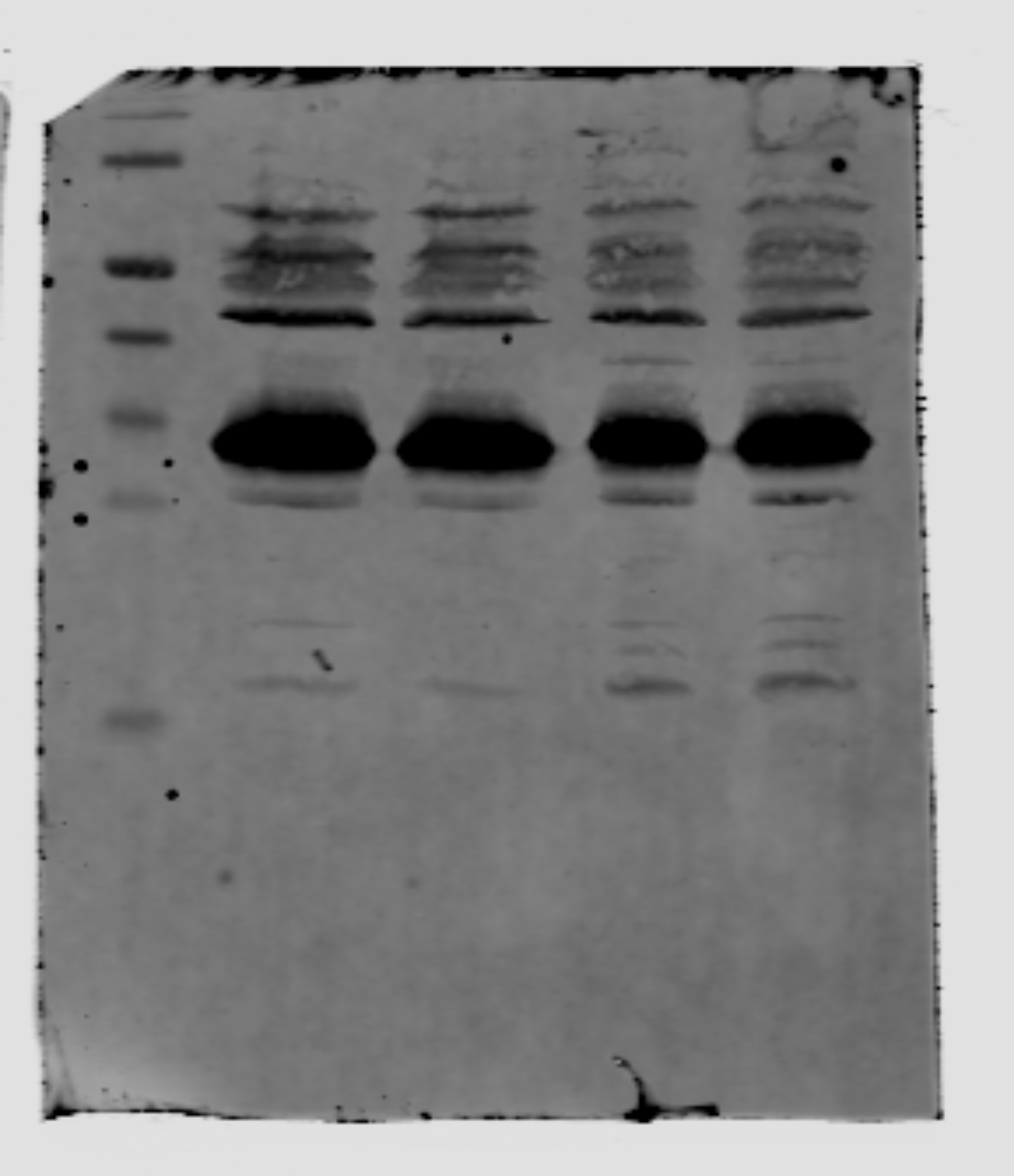

Supplement: Multimedia component 2 [file mmc2.zip › Supplemental_files/Figure 3/Figure 3E/HepG2/cleaved and caspase-3.tif]

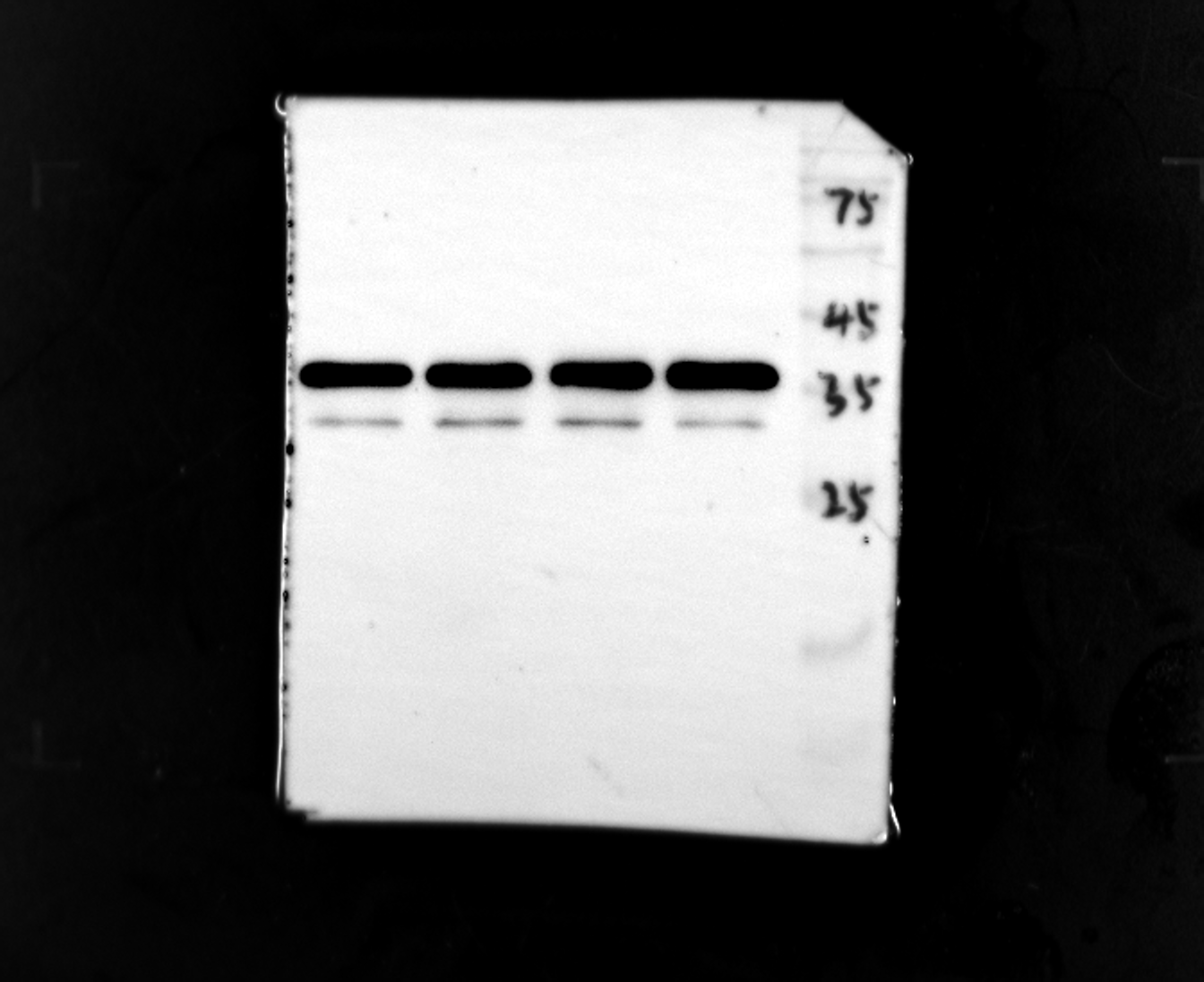

Supplement: Multimedia component 2 [file mmc2.zip › Supplemental_files/Figure 3/Figure 3E/LM3/GAPDH.Tif]

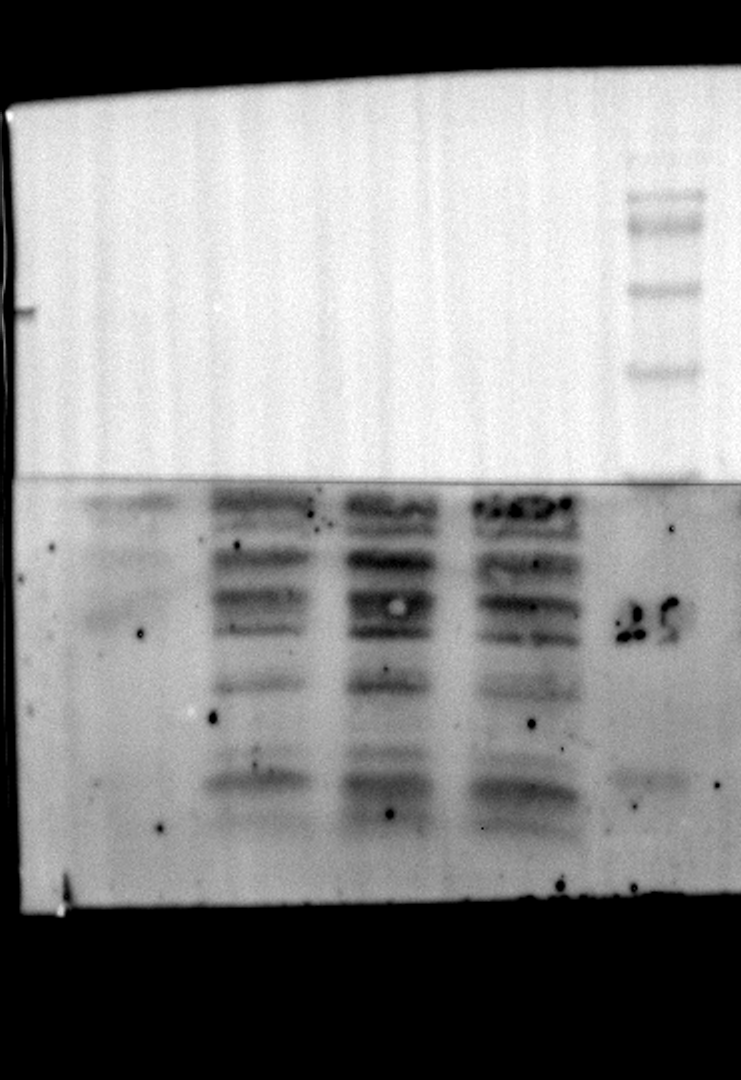

Supplement: Multimedia component 2 [file mmc2.zip › Supplemental_files/Figure 3/Figure 3E/LM3/LC3I and LC3 II.Tif]

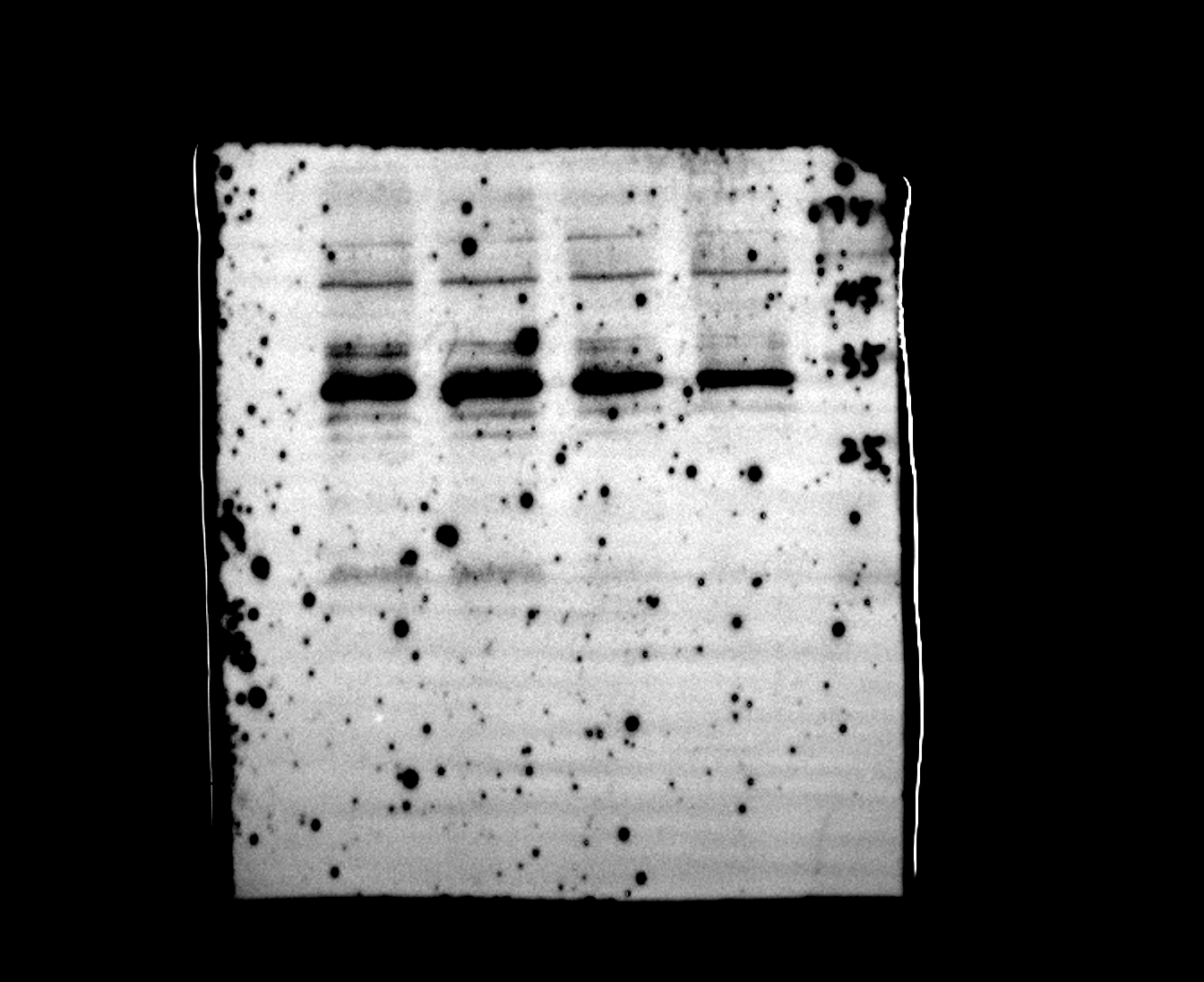

Supplement: Multimedia component 2 [file mmc2.zip › Supplemental_files/Figure 3/Figure 3E/LM3/cleaved and Caspase-3.Tif]

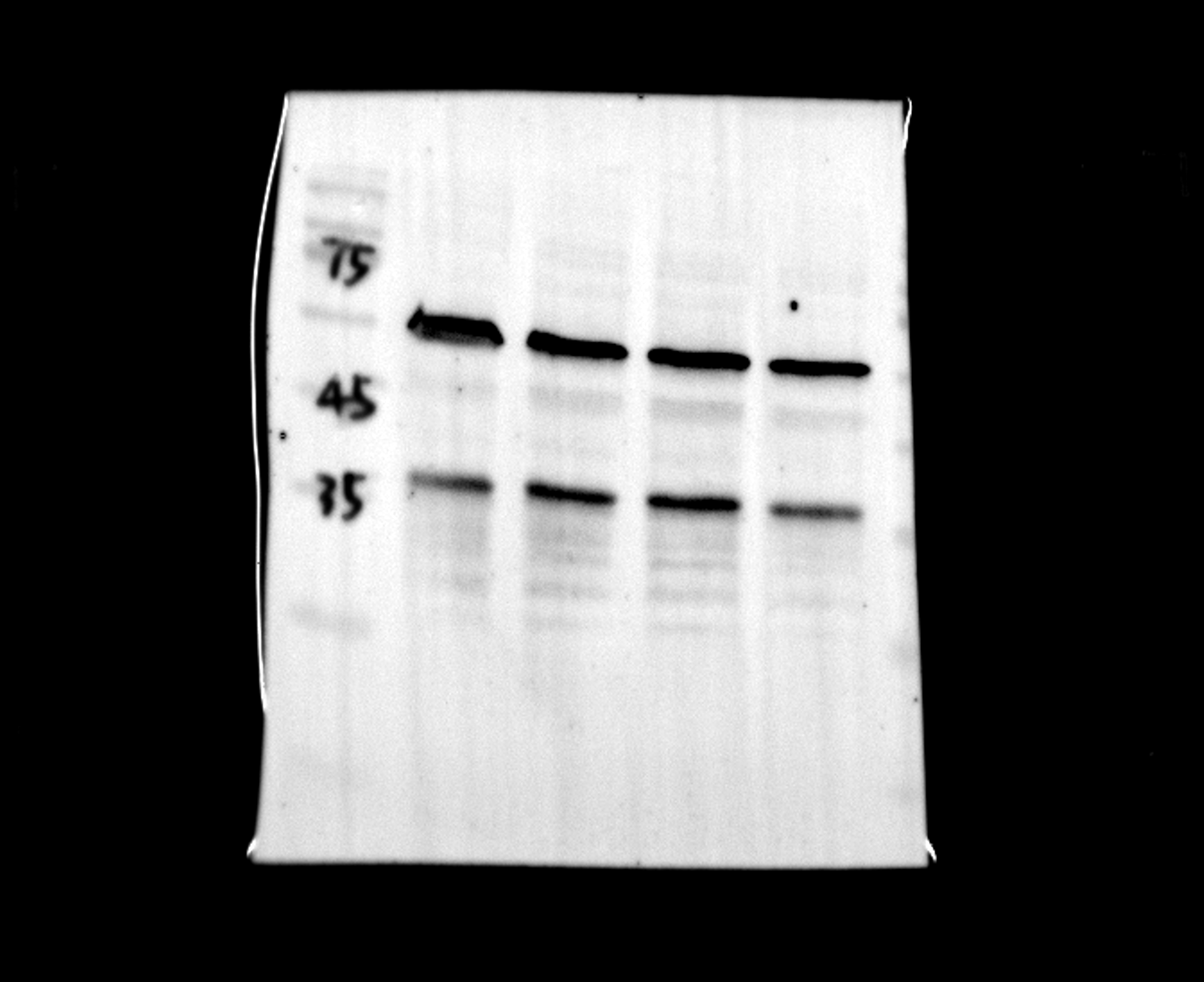

Supplement: Multimedia component 2 [file mmc2.zip › Supplemental_files/Figure 4/Figure 4A/HepG2/AKT.Tif]

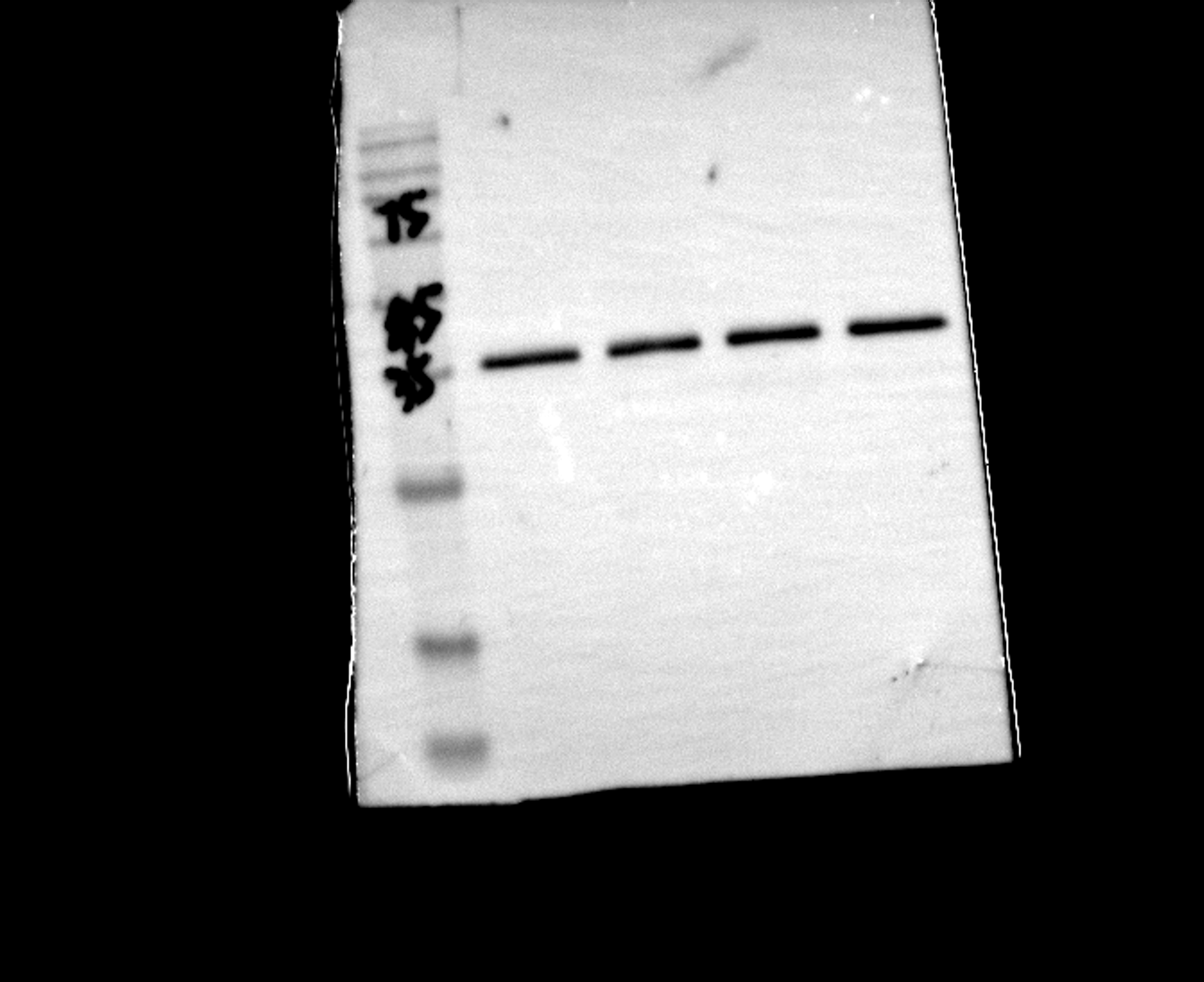

Supplement: Multimedia component 2 [file mmc2.zip › Supplemental_files/Figure 4/Figure 4A/HepG2/GAPDH.Tif]

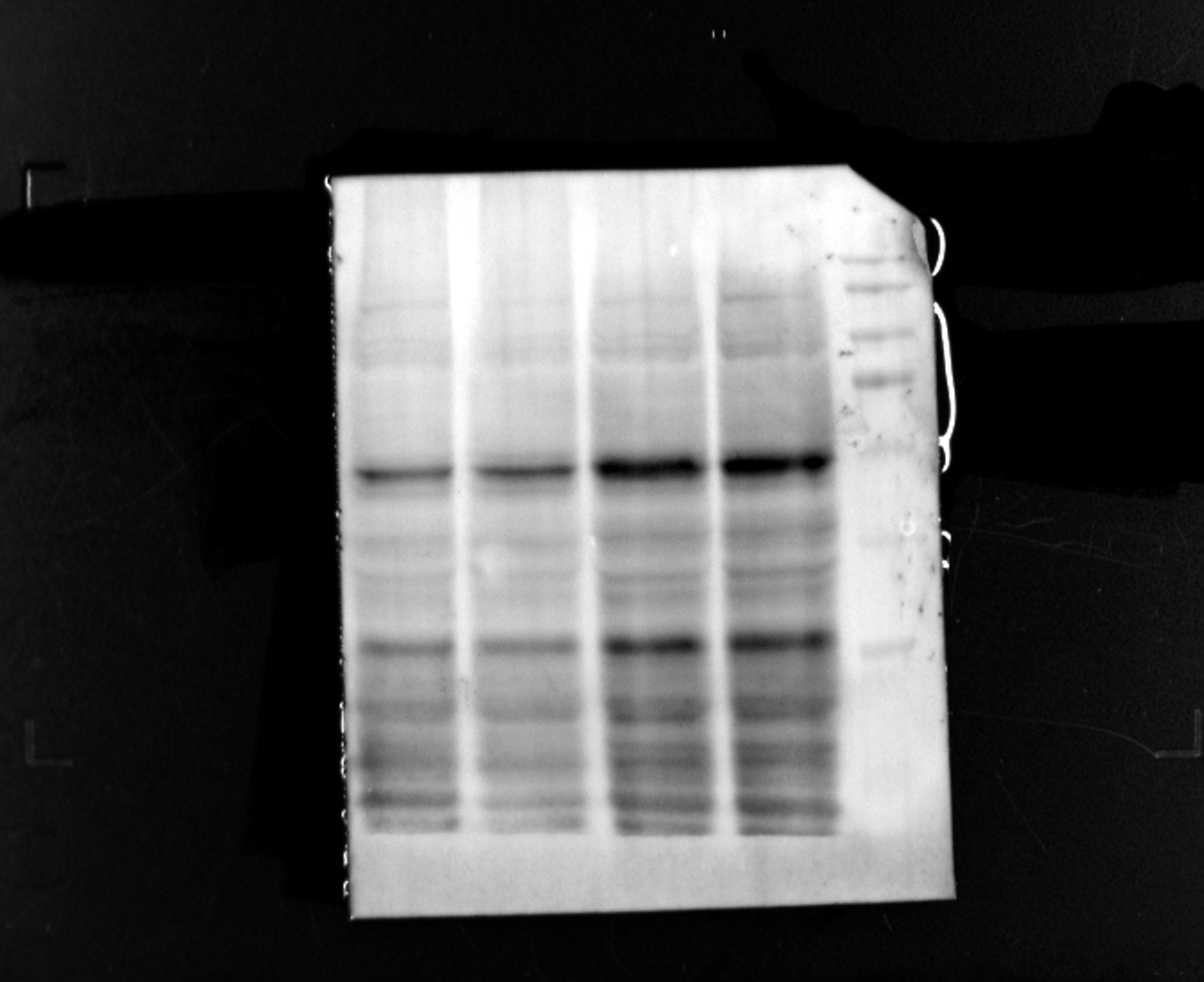

Supplement: Multimedia component 2 [file mmc2.zip › Supplemental_files/Figure 4/Figure 4A/HepG2/P-AKT.Tif]

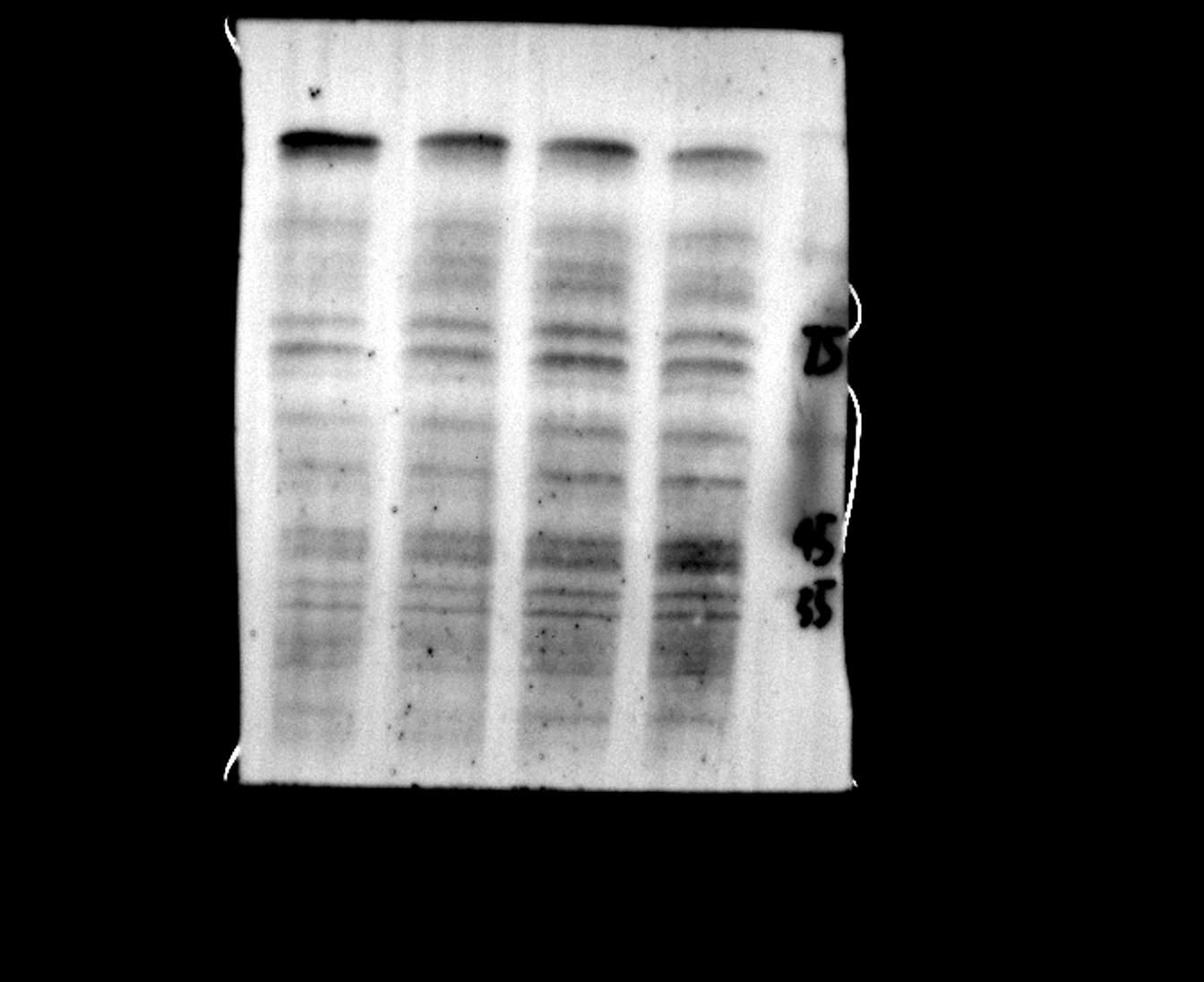

Supplement: Multimedia component 2 [file mmc2.zip › Supplemental_files/Figure 4/Figure 4A/HepG2/mTOR.Tif]

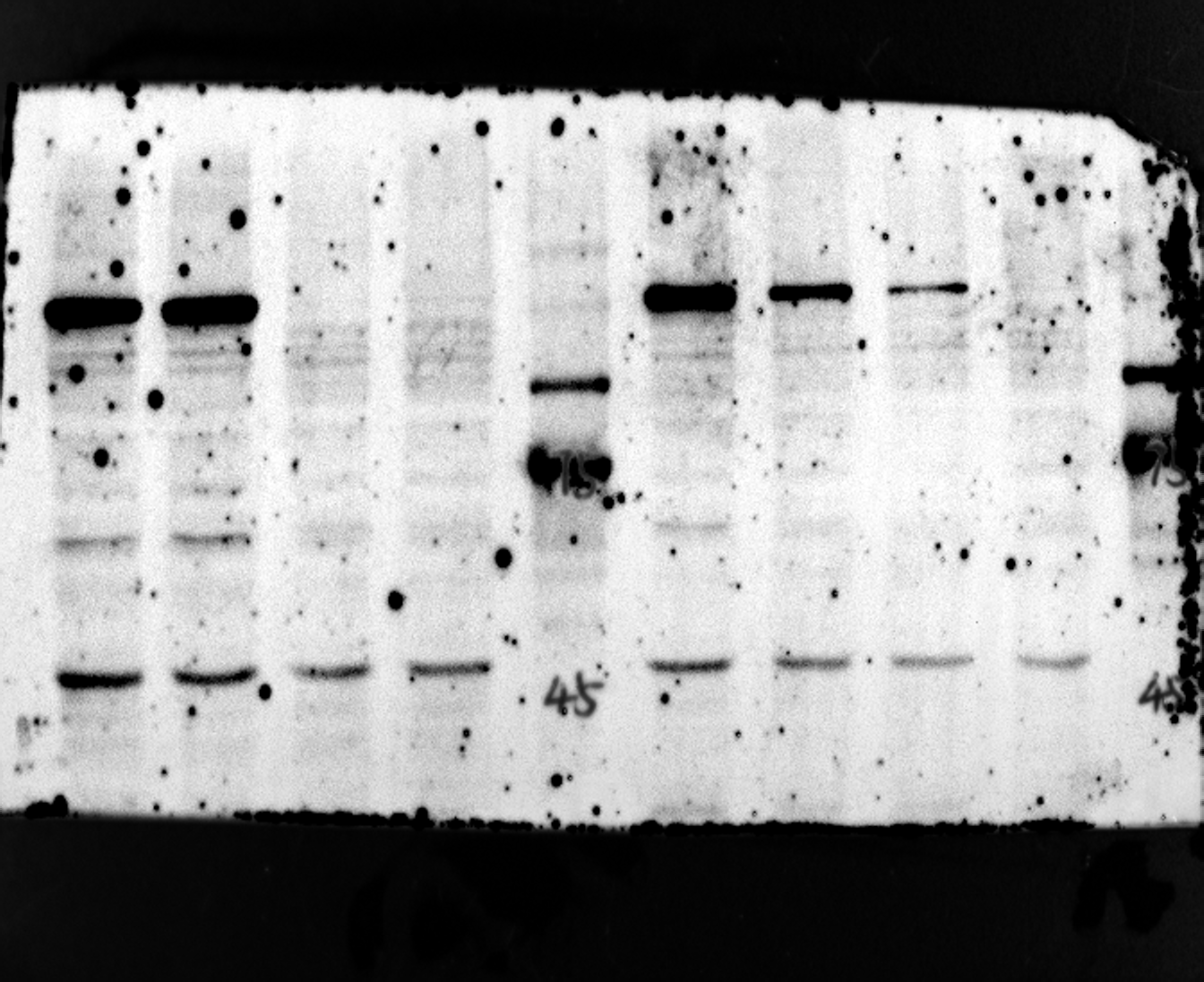

Supplement: Multimedia component 2 [file mmc2.zip › Supplemental_files/Figure 4/Figure 4A/HepG2/p-mTOR(right).Tif]

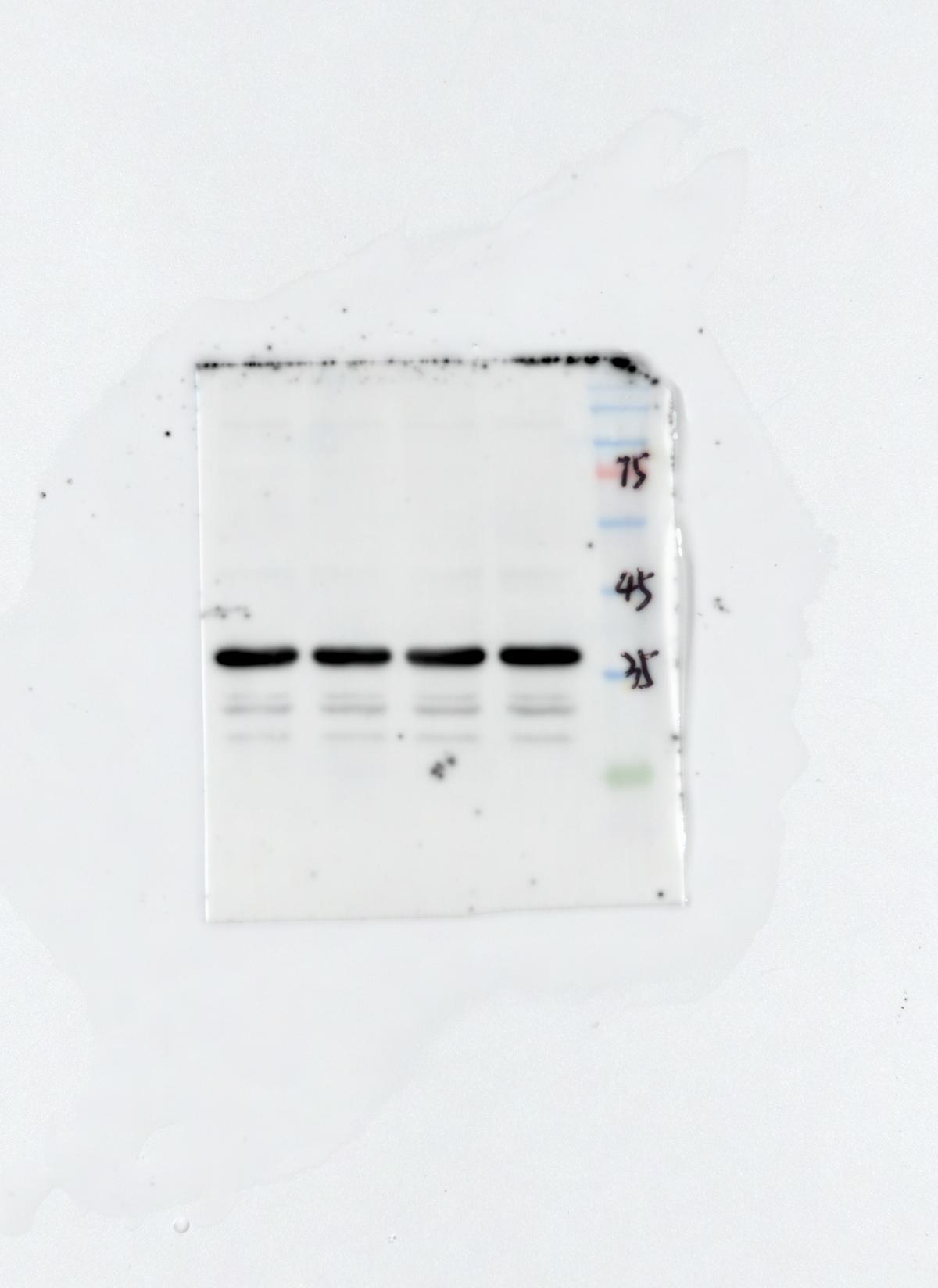

Supplement: Multimedia component 2 [file mmc2.zip › Supplemental_files/Figure 4/Figure 4A/LM3/0730GAPDH2 .jpg]

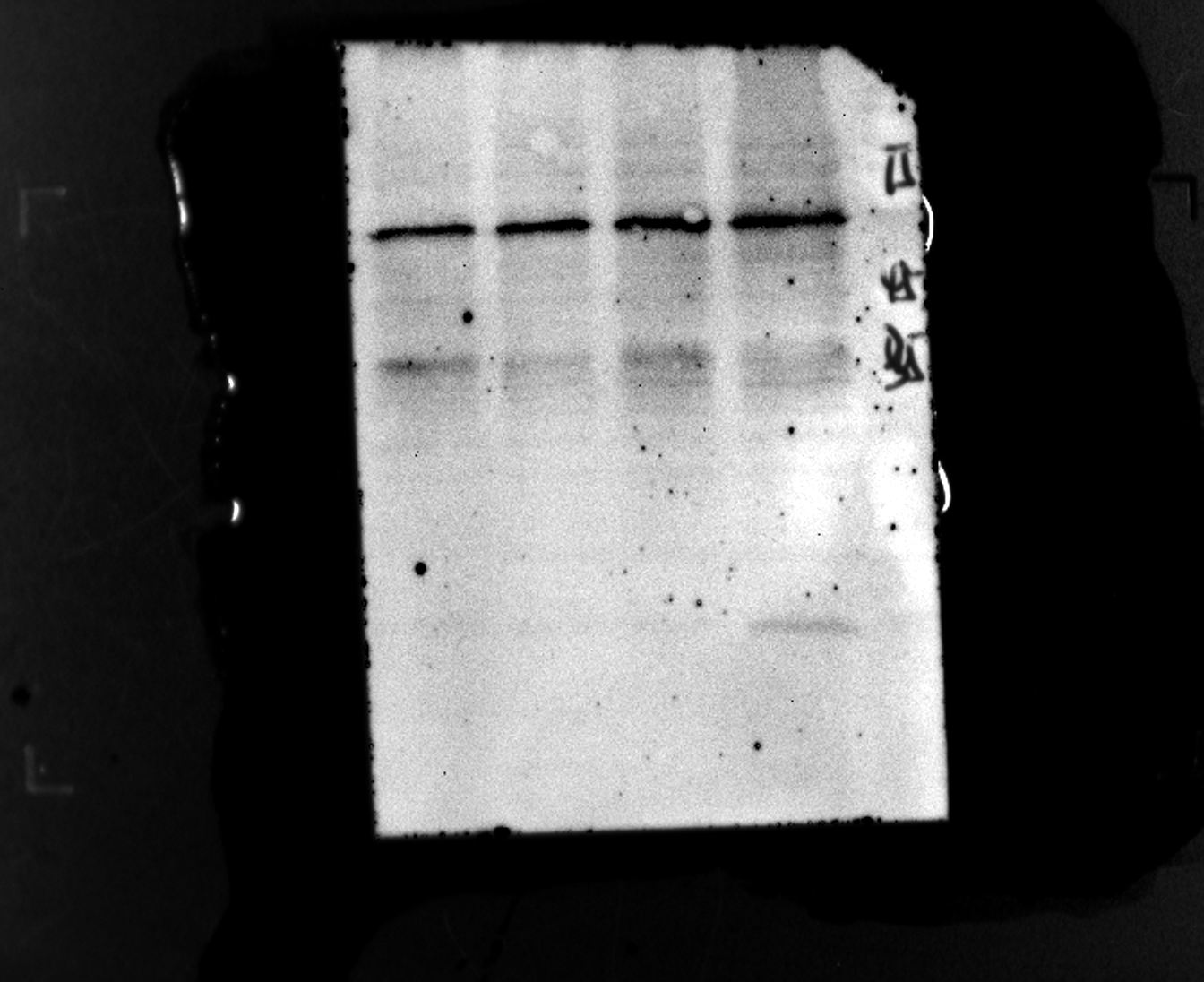

Supplement: Multimedia component 2 [file mmc2.zip › Supplemental_files/Figure 4/Figure 4A/LM3/AKT.Tif]

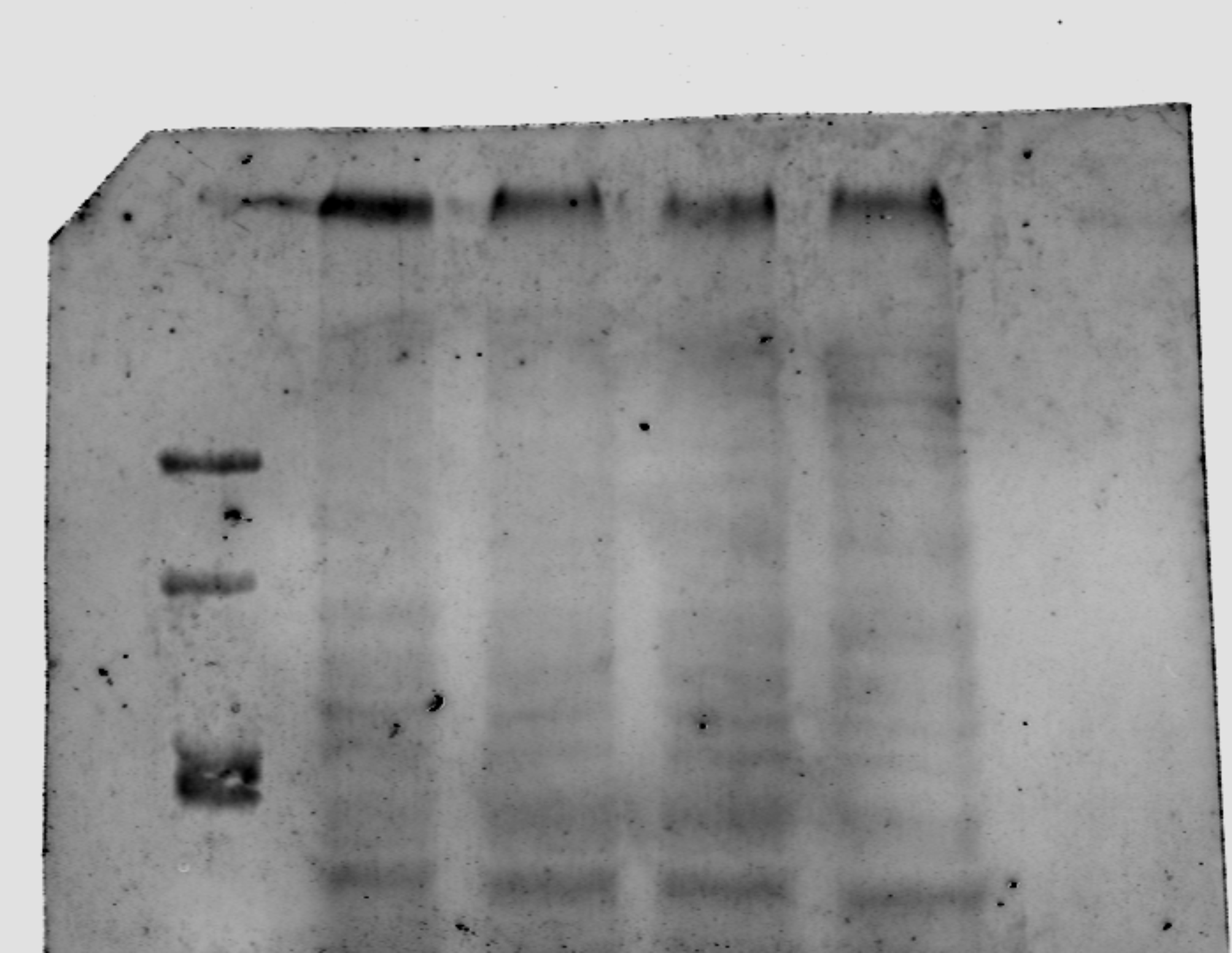

Supplement: Multimedia component 2 [file mmc2.zip › Supplemental_files/Figure 4/Figure 4A/LM3/P-MTOR.tif]

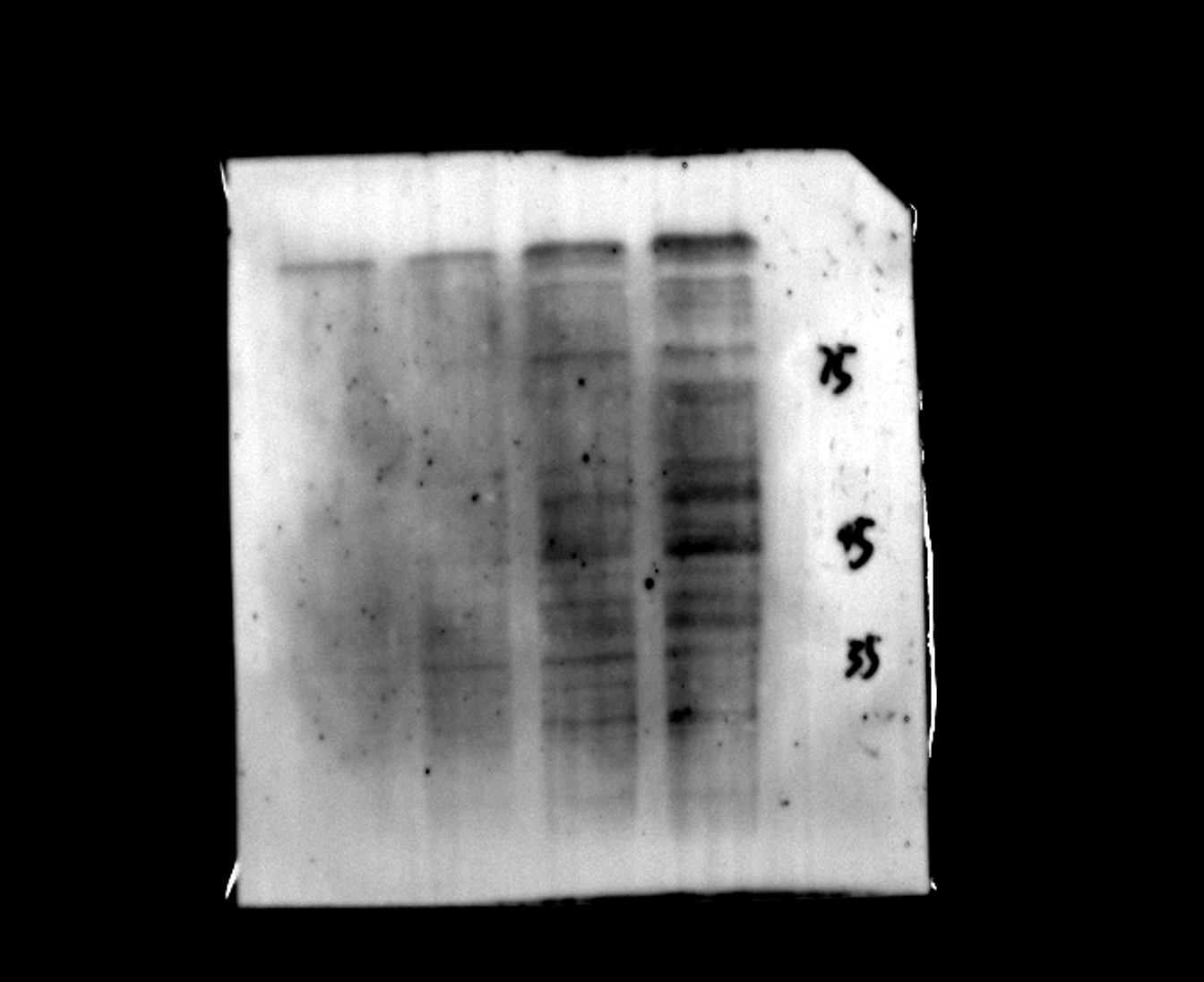

Supplement: Multimedia component 2 [file mmc2.zip › Supplemental_files/Figure 4/Figure 4A/LM3/mTOR.Tif]

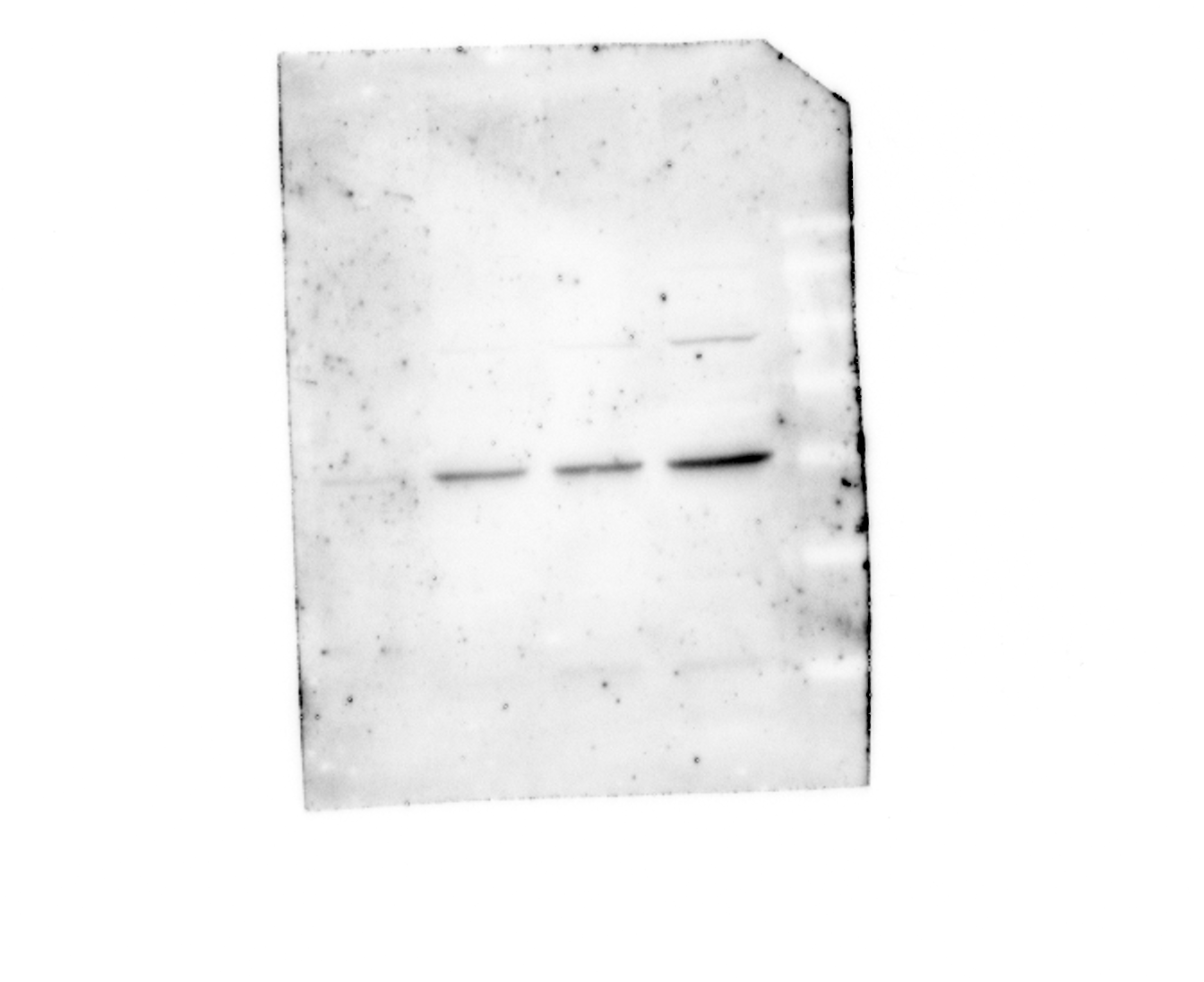

Supplement: Multimedia component 2 [file mmc2.zip › Supplemental_files/Figure 4/Figure 4A/LM3/p-AKT.Tif]
